# Opportunities and new developments for the study of surfaces and interfaces in soft condensed matter at the SIRIUS beamline of Synchrotron SOLEIL

**DOI:** 10.1107/S1600577523008810

**Published:** 2024-01-01

**Authors:** Arnaud Hemmerle, Nicolas Aubert, Thierry Moreno, Patrick Kékicheff, Benoît Heinrich, Sylvie Spagnoli, Michel Goldmann, Gianluca Ciatto, Philippe Fontaine

**Affiliations:** a Synchrotron SOLEIL, L’Orme des Merisiers, Départementale 128, 91190 Saint-Aubin, France; b Institut Charles Sadron, Université de Strasbourg, CNRS UPR22, 67034 Strasbourg, France; c Institut de Physique et Chimie des Matériaux de Strasbourg, Université de Strasbourg, CNRS UMR7504, 67034 Strasbourg, France; d Institut des NanoSciences de Paris, UMR 7588 CNRS, Sorbonne Université, 75252 Paris Cedex 05, France; University of Tokyo, Japan

**Keywords:** beamline, soft condensed matter, grazing-incidence techniques, TXRF, surface science

## Abstract

The SIRIUS beamline of Synchrotron SOLEIL is dedicated to X-ray scattering and spectroscopy of surfaces and interfaces, covering the tender to mid-hard X-ray range (1.1–13 keV). The beamline has hosted a wide range of experiments in the field of soft interfaces and beyond, providing various grazing-incidence techniques such as diffraction and wide-angle scattering (GIXD/GIWAXS), small-angle scattering (GISAXS) and X-ray fluorescence in total reflection (TXRF). SIRIUS also offers specific sample environments tailored for *in situ* complementary experiments on solid and liquid surfaces. Recently, the beamline has added compound refractive lenses associated with a transfocator, allowing for the X-ray beam to be focused down to 10 µm × 10 µm while maintaining a reasonable flux on the sample. This new feature opens up new possibilities for faster GIXD measurements at the liquid–air interface and for measurements on samples with narrow geometries.

## Abstract 2

The main characteristics of the beamline SIRIUS at Synchrotron SOLEIL relevant for soft matter science are presented, as well as recent developments of new sample environments and of X-ray focusing with compound refractive lenses.

## 1. Introduction

Synchrotron-based grazing-incidence X-ray techniques are powerful tools for investigating the structure and dynamics of surfaces and interfaces in soft condensed matter (Daillant, 2009 ▸; Pershan & Schlossman, 2012 ▸; Hexemer & Müller-Buschbaum, 2015 ▸; Narayanan & Konovalov, 2020 ▸). The pioneering work of Kjaer *et al.* (1987 ▸) and Dutta *et al.* (1987 ▸), which first introduced these techniques for the study of organic layers at the air–water interface using grazing-incidence X-ray diffraction (GIXD) in 1987, has paved the way for their further development and application in the study of interfaces in soft matter systems. The establishment of third-generation synchrotron sources has led to the emergence of a diverse and complementary family of techniques, enhancing our ability to investigate interfaces in soft matter systems using grazing-incidence methods.

The grazing-incidence geometry relies on total reflection of X-rays, which occurs when the incident wave angle is below a critical value and propagates from a medium with a higher refractive index to an interface with a medium of lower refractive index, such as at air–liquid or air–solid interfaces (Daillant & Gibaud, 2009 ▸). Using X-rays with energies of typically 10 keV, only a thin interfacial layer of a few nanometres in depth is illuminated, while the bulk condensed phase remains unexposed and does not obscure the signal resulting from the interaction of X-rays with the surface (Fontaine *et al.*, 2004 ▸). The photons that interact with the thin interfacial layer can undergo several events, including elastic scattering, inelastic scattering and absorption (Daillant & Gibaud, 2009 ▸). Elastic scattering results in either diffuse or diffraction patterns if the layer has a degree of organization, while absorption results in the re-emission of a lower-energy photon, in the so-called process of fluorescence.

Several techniques have been developed to provide a comprehensive understanding of air–liquid and air–solid interfaces at different length scales, depending on the detection technique, angular range, collimation, variation of the incidence angle and potentially with chemical sensitivity. At low in-plane angles between the detection direction and incident beam (2θ < 1°), grazing-incidence small-angle X-ray scattering (GISAXS) has been successfully used to unravel the structure of highly organized thin films at the nanoscale, such as, for example, surfaces patterned by the controlled deposition of block copolymers (Aubrit *et al.*, 2018 ▸; Yu *et al.*, 2020 ▸; Demazy *et al.*, 2023 ▸). Moreover, the low divergence and high flux offered by third-generation synchrotron sources have played a crucial role in the increasing popularity of GISAXS measurements at the liquid–air interface (Daillant, 2009 ▸). Thus, the self-assembly of various systems, such as semi-fluorinated alkanes (Fontaine *et al.*, 2005 ▸, 2018 ▸), colloids (Li-Destri *et al.*, 2019 ▸) and metal-organic frameworks (MOFs) (Andrés *et al.*, 2021 ▸) or polyhedra (MOPs) (Tejedor *et al.*, 2022 ▸) at the air–water interface has been investigated using synchrotron-based GISAXS. At wider detection angles (2θ > 1°), the geometry of grazing-incidence wide-angle X-ray scattering (GIWAXS) is preferred on solid thin films with crystalline features of length scales below 10 nm (Rivnay *et al.*, 2012 ▸). This technique has become increasingly popular for understanding structure–function relationships in organic electronics (King *et al.*, 2021 ▸; Galerne *et al.*, 2021 ▸; Labiod *et al.*, 2022 ▸; Talnack *et al.*, 2022 ▸; Swaraj & Hemmerle, 2023 ▸) and perovskite thin films (Filonik *et al.*, 2019 ▸; Steele *et al.*, 2023 ▸). The small interception of the sample with the beam in the geometry used for GISAXS and GIWAXS allows for the preservation of angular and wavevector resolution, enabling rich structural information to be extracted from the measured scattering patterns in a single shot using an area detector and a beamstop. This approach provides several advantages, as it enables the capture of essential features such as crystalline order, grain size and coherence length, among others. In most cases, the image can be acquired in a sufficiently short time (of the order of 1–10 min) to capture dynamic events in so-called *in situ* experiments, where the film or device is subjected to external stimuli such as exposure to a particular solvent, gas or light, a change in relative humidity, or even during operation in the case of *operando* measurements.

Grazing-incidence X-ray diffraction (GIXD) is generally preferred for Langmuir films, *i.e.* molecular layers located at liquid–air interfaces, with scans of the reciprocal space using a collimator in front of a linear detector or an area detector. Because of the significant interception of the beam with the liquid surface under grazing-incidence conditions (of the order of 50 mm × 1 mm), the previous GIWAXS geometry yields poor angular resolution on liquid surfaces, making it unsuitable for obtaining valuable measurements. GIXD provided a major breakthrough in the understanding of Langmuir films in the 1990s (Kaganer *et al.*, 1999 ▸) and has since been widely used to explore various systems, including the self-assembly of molecular rigid rods (Fontaine *et al.*, 2019 ▸), the effects of drugs and pollutants on biomimetic systems (Michel *et al.*, 2015 ▸; Pereira-Leite *et al.*, 2019 ▸; Lopes-de-Campos *et al.*, 2021 ▸; Wójcik *et al.*, 2022 ▸; Matyszewska *et al.*, 2023 ▸), luminescence in 2D molecular crystals (Biswas *et al.*, 2021 ▸) and the structure of graphene oxide films (Bonatout *et al.*, 2017 ▸).

The SIRIUS (Soft Interfaces and Resonant Investigation on Undulator Source) beamline at Synchrotron SOLEIL was designed to provide grazing-incidence scattering and spectroscopy techniques over a unique energy range of 1.1–13 keV covering the tender to mid-hard X-rays domains (Fontaine *et al.*, 2014 ▸; Ciatto *et al.*, 2016 ▸). Since 2013, the beamline has been opened to users belonging to various communities in the fields of physics, chemistry and biology, with scientific interest in diverse topics such as functional materials, cell biology, environmental protection, health, energy storage and production. In the present paper, we discuss the recent updates to the beamline that are particularly relevant for the study of soft condensed matter systems. We first recall the main elements of the beamline layout, including the new compound refractive lenses (CRLs) focusing system. Next, we detail the new GIWAXS/GISAXS environment for studies on solid substrates, the different strategies available to catch diffraction signal from Langmuir films associated with UV–visible spectroscopy, and the recent modifications to the various facilities setups and configurations available on the beamline that are of interest to the soft matter community. Finally, we present a custom electronic notebook fully integrated to the data collection strategy of the beamline.

## 2. Beamline overview

**Figure 1:**
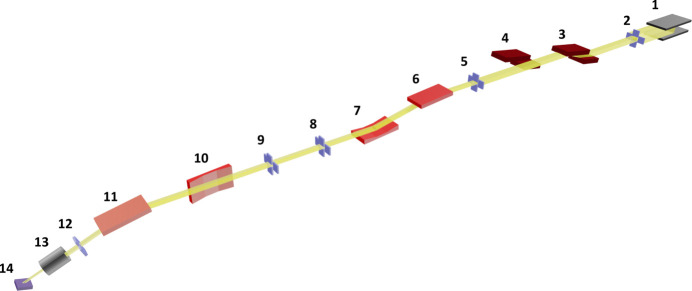
Simplified layout of SIRIUS.

The main components of the beamline are shown in Fig. 1 ▸ with the corresponding positions listed in Table 1 ▸. In the following, we describe only the double-crystal monochromator (DCM), the optical elements and the various endstations that are relevant to the study of soft matter systems in the energy range of ∼6–13 keV with linear polarization. Readers interested in the use of tender X-rays (between 1.1 and 5 keV), variable polarization, the multilayer grating monochromator (MGM) and the absorption spectroscopy capabilities of the SIRIUS beamline and associated optical elements, or in the in-vacuum diffractometer endstation, are referred to previous published papers on the subject (Ciatto *et al.*, 2016 ▸, 2019 ▸).

### 2.1. Undulator

SIRIUS operates a helicoidal Apple-II undulator placed on a straight section, with a magnetic period of 36 mm (HU36) that provides X-rays covering a continuous energy range of at least 1–13 keV with variable polarization (linear horizontal, vertical or circular). This wide energy range is quite rare for an undulator source and required challenging optimization of the magnetic field and electron beam feedback corrections (Briquez *et al.*, 2008 ▸; Kitegi *et al.*, 2010 ▸). The undulator delivers a beam with a size first fixed by a diaphragm of 2.2 mm × 0.8 mm opening at 11.7 m from the source, followed by a pair of water-cooled primary slits (H and V) at 17 m from the source to define the usable beam before the monochromators.

### 2.2. Double-crystal monochromator

The fixed-exit DCM (FMB-Oxford, UK) located at 18.5 m from the source produces a monochromatic beam in the energy range 2–13 keV using a pair of liquid-nitrogen-cooled silicon crystals [111]. Such a large energy range requires a wide set of accessible Bragg angles (from −3 to 85°), which is rare for a cryo-cooled DCM. It is indeed quite difficult to prevent tubes and cables from intercepting the white beam on an angular range of about 90° while maintaining optimal stability and repeatability of the motors against gravity at large Bragg angles. Prior to each experiment, the monochromator energy calibration is checked by X-ray absorption spectroscopy near the absorption edges of nickel (*K*-edge, 8.333 keV) or zinc (*K*-edge, 9.659 keV) foils, ensuring a resolution of less than 5 eV in the 6–13 keV energy range. The control-command system of the DCM was upgraded to a Delta Tau PowerPMAC controller (Engblom *et al.*, 2019 ▸), which allows driving the DCM directly in energy and opens the possibility of continuously scanning over the beamline energy range. This feature also enables quick energy changes during an experiment, facilitating, for example, adaptation of the *q*-range in GISAXS or optimization of fluorescence emission after changing a sample.

### 2.3. Mirrors and slits

The beamline is equipped with a set of four silicon mirrors downstream of the monochromators (DCM and MGM). These mirrors have different tracks coated with materials selected to optimize reflectivity at the chosen energy. For energies above 6 keV, platinum-coated tracks are used systematically. Mirror M1 is located 22 m from the source and reflects the beam downwards by 12 mrad (6 mrad on the mirror), providing harmonic rejection. Mirror M2, located at 26 m, can focus the beam vertically using a mechanical bender and reflects it back to a horizontal direction. Mirror M3, located at 31.2 m from the source, can focus the beam horizontally with the help of mechanical benders as well. Finally, mirror M4, situated at 32.8 m from the source, can be used to deflect the beam downwards to impinge on horizontal samples such as liquid surfaces, with an incidence of up to 0.5° on the mirror (1° on the sample). Vertical and horizontal focusing on the sample is achieved by using M2 and M3 to focus the beam to a spot of 100 µm × 100 µm. Smaller beam sizes can be reached using a pair of slits as secondary source in combination with M2 and M3, with a flux reduction proportional to the closure. Two pairs of collimation (optical) slits, located after the M1 and M2 mirrors, allow us to define the size of the parallel beam in the unfocused directions, and a final set of anti-scattering slits is located just before the sample. These last slits are of prime importance when performing GISAXS at very small angles, for cleaning the beam of the spurious scattering caused by the optical slits upstream. The natural divergence of the beam is 50 µrad in both vertical and horizontal directions, and can be lowered to 10–30 µrad at the sample position with collimation using the aforementioned slits. With such tunable configurations of mirrors and slits, the beam size can already be controlled to vary from millimetres to tens of micrometres with controlled divergence. The beam size can be further improved using our CRLs system, which is described in detail hereafter.

### 2.4. Compound refractive lenses

While the use of the two focusing mirrors M2 and M3 in combination with a significant closure of the secondary slits results in a large loss of flux, beam sizes smaller than 50 µm have been achieved and utilized on the beamline. This has enabled investigations into the structure of confined surfactant solutions in a SFAX (a surface force apparatus combined with X-ray scattering, SAXS and WAXS), despite the low flux. The SFAX, optimized for being operated on SIRIUS, opens the route to establishing the force–distance–structure relationships under controlled nanoconfinement and under stress (Kékicheff *et al.*, 2018 ▸; Kékicheff, 2019 ▸). In one such experiment, a collimated beam with a cross section of around 10 µm was required to match the cross-sectional area of the two opposing immersed surfaces. Although the success in achieving sub-50 µm beam sizes is remarkable, there is a clear need in our user community to maintain high flux while strongly focusing the beam. For instance, we are currently working to increase the level of integration of SFAX to the beamline, which will greatly benefit the combination of a small beam and a high flux. More generally, this would be beneficial to various studies of confinement effects in soft matter systems (*e.g.* nanometric polymer layers, correlations between confined ions, phase transition in colloidal systems, ionic liquids), of heterogeneous samples with complex geometries, and of microfluidic chips with small lateral dimensions.

To focus the X-ray beam onto a small area while minimizing flux loss, we recently designed and acquired a transfocator capable of focusing the beam throughout a large range of energies and distances by inserting a variable number of CRLs into the beam (Vaughan *et al.*, 2011 ▸). The transfocator is designed to work in combination with the M2–M3 mirrors for pre-focalization. To cover a wide energy range of 4 to 12 keV, we chose to use 24 beryllium lenses arranged in five packs, each on a motorized translation with excellent micrometric repeatability. The configuration of the lenses is described in Table 2 ▸. The transfocator was commissioned in 2022 and is now available for users. We describe here its main characteristics and performance in terms of focusing.

**Figure 2:**
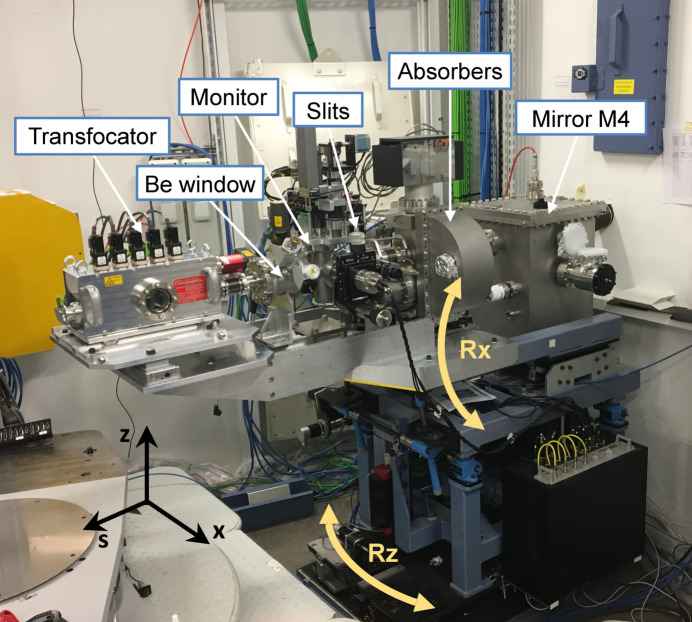
Implantation of the transfocator on the beamline immediately before the diffractometer and sample stage, with the preceding upstream elements.

The lenses are identical and made of IS-50M beryllium with a radius of curvature at the apex of the parabola of *R* = 50 µm, a geometric aperture 2*R*_0_ = 628 µm, a length *L* = 2 mm and a web thickness of *d* = 30 µm. The lenses were machined by RXOPTICS GmbH (Germany), while the pack holder, translations and transfocator vessel were purchased from Cinel Srl (Italy). Figure 2 ▸ depicts the system and its installation on the beamline. IS-50M beryllium has a relatively coarse grain microstructure, but it is suitable for focusing X-rays in the desired energy range. However, its polycrystalline structure may limit its future use with coherent beams when using the transfocator (Lyatun *et al.*, 2020 ▸). The transfocator is mounted on a motorized longitudinal translation (according to the beam) that can be adjusted to shift the focal point when inserting or removing packs. The entire assembly is further mounted on a table than can rotate around a vertical axis (*Rz*) centred on the M3 mirror rotation axis, and a horizontal axis (*Rx*) centred on the M4 mirror rotation (see Fig. 2 ▸). These two rotations enable all elements, including the transfocator, to accommodate the horizontal deviation of the M3 mirror and the vertical deflection of the M4 mirror.

**Figure 3:**
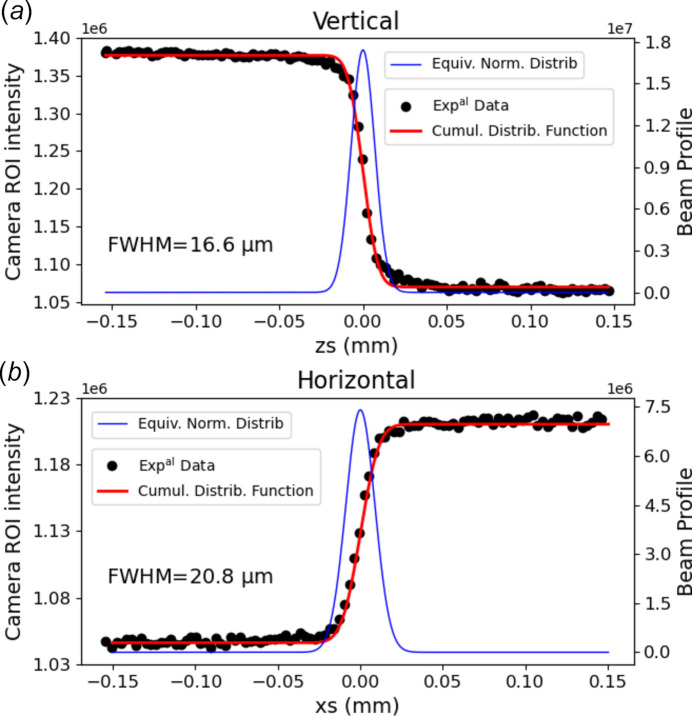
Smallest beam size obtained with the transfocator without closing the secondary slit. Vertical (*a*) and horizontal (*b*) scans of a knife-edge blade fitted by the cumulative distribution function of a normal law (red line) and the associated normal law used to extract the beam size (blue line).

To determine the size of the focused beam, we utilized a knife-edge corner mounted on the six-axis tower of the Newport diffractometer (see Section 2.5). The knife-edge was scanned vertically and horizontally using the motors of the diffractometer, at a fixed energy of 8 keV. The intensity profiles obtained were fitted with the cumulative distribution function of a Gaussian beam shape (González-Cardel *et al.*, 2013 ▸). Figure 3 ▸ shows the curves used to determine the smallest beam size obtained without resorting to the secondary slit as a secondary X-ray source, *i.e.* with an opening of 2 mm × 1.5 mm (H × V) that corresponds to an almost full beam. After optimization of the transfocator alignment (*Rx*, *Rz*) and focalization, we obtained a beam size (full width at half maximum – FWHM) of 21 µm × 17 µm (H × V). Note that the pre-focalization point by the M2 and M3 mirrors should be located within the transfocator to obtain the smallest sizes possible.

As a comparison, we measured the beam size without using the transfocator. First, we removed the lenses and kept the same pre-focalization with M2 and M3. In this case, the beam was considerably larger on the knife-edge at the centre of the diffractometer (300 µm × 150 µm). This is obviously expected since the focal point is located within the transfocator and not on the diffractometer centre. After readjusting the focal point on the knife-edge, still without the transfocator, we obtained a beam as small as 100 µm × 100 µm. To quantify the loss of intensity caused by the transfocator, we replaced the knife-edge corner by an ion chamber (IC PLUS 50; FMB-Oxford, England) filled with nitrogen at ∼1 atm, coupled to a current amplifier DDPCA-300 (FEMTO Messtechnik GmbH, Berlin, Germany) and connected to an ADC ADLink 2005 (ADLINK Technology Inc., Taiwan). Comparing the intensity with and without the transfocator inserted in the beam, we measured a transmission of 28 ± 1% at 8 keV with ten lenses inserted (packs 2 and 5).

We can compare our measurement of the transmission with the theoretical value derived analytically by Lengeler *et al.* (1999 ▸) for a stack of *N* CRLs, using here only the dimensions of the lenses and the linear coefficient of attenuation μ,

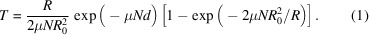

At an energy of 8 keV and for beryllium, we have μ = 2.077 cm^−1^ (Henke *et al.*, 1993 ▸). Thus, we find that the calculated transmission for our CRLs stack is 23%, which is close to our measured value. This agreement validates the efficiency of the chosen optical scheme, which includes a pre-focalization stage that allows the full flux to enter the transfocator through its aperture. Another interesting quantity that can be derived here is the focal length of the transfocator, which can be expressed as *f* = *R*/(2*N*δ), where δ is the real part of the complex X-ray refractive index. For our system, this gives us a focal length of *f* = 0.47 m at 8 keV. We provide in Table 2 ▸ values of the transmissions and focal lengths computed with equation (1) for the different energies. It is anticipated that the insertion of the CRLs may alter the divergence of the beam. Numerical simulations of the beamline conducted by SOLEIL’s optics group have estimated a divergence of 100 µrad in both vertical and horizontal directions. However, it is important to note that this estimate was obtained without considering the collimation of the incident beam with our optical slits. Therefore, the actual divergence of the focused beam should be measured on a case-by-case basis, but with an upper bound of 100 µrad. These values still allow for measurements at grazing incidence (below 1 mrad) without the risk of X-rays exceeding the critical angle.

To conclude, keeping in mind our ultimate goal, we were able to achieve a beam size of 10 µm × 10 µm by closing the secondary slits to 100 µm × 100 µm, with a proportional loss of intensity.

### 2.5. Diffractometer and six-axis tower

The beamline is equipped with two diffractometers: a large seven-circle diffractometer manufactured by Micro-contrôle/Newport (Evry, France) located at a distance of 35 m from the source (Fontaine *et al.*, 2014 ▸; Ciatto *et al.*, 2016 ▸), and a second four-circle in-vacuum diffractometer manufactured by Symetrie (Nîmes, France) located 39 m from the source (Ciatto *et al.*, 2019 ▸). The latter can be removed from the hutch and replaced with an optical table, as described in Section 3.2. The sample stages associated with the in-vacuum diffractometer have been extensively described in previous works, and here we only recall the main features of the six-axis tower stage on the large seven-circle diffractometer. This stage can accommodate different sample environments, including heavy ones up to 200 kg with an off-centre mass. It allows for precise movement of the sample in six degrees of freedom, with a sphere of confusion of all rotations of less than 60 µm. The stage features a precise vertical translation of the sample with negligible backlash and 1 µm repeatability, crucial for liquid surface alignment. In addition, two in-plane translations with a range of 50 mm are present, along with sample rotations provided by two crossed cradles limited to ±10°, and a full rotation along the vertical axis. The resolution and repeatability of the motors for sample positioning are extremely accurate (Ciatto *et al.*, 2016 ▸), which is particularly useful for measurements in complex sample geometries (Kékicheff *et al.*, 2018 ▸; Galerne *et al.*, 2021 ▸).

The diffractometer is equipped with two detector arms: a first arm that can carry heavy detectors and collimation assemblies (up to 50 kg) and allows two rotations, in and out of the plane of the sample; and a second arm consisting of an optical table on which equipment weighing up to 200 kg can be positioned at distances ranging from 1.4 to 3.1 m from the sample, and which can rotate horizontally around the centre of the diffractometer on a marble track. A third static arm was added to the original setup to hold a third detector close to the sample for complementary measurements, usually an X-ray fluorescence detector, an optical camera or a mobile microscope.

### 2.6. Detectors

The main features and uses of the detectors available on SIRIUS are described in Table 3 ▸. Multiple monitors and X-ray beam-position monitors (XBPMs) are placed between the main optical elements of the beamline and used for their alignment or for beamline monitoring. The XBPMs consist of 50 µm-thick (for high energies) and 3 µm-thick (for tender energies) diamond crystals forming a four-quadrants diode (Desjardins *et al.*, 2014 ▸; Ciatto *et al.*, 2016 ▸). The beam position is given by the barycenter of the four currents returned by each diamond after amplification by a LOCuM-4 amplifier (ENZ, Berlin, Germany), and the total intensity by their sum through a TANGO dedicated device. A complementary X-ray camera, developed by the SOLEIL detector group (Bordessoule, 2013 ▸), is used after the endstations to align the beamline and the samples. It can withstand the full intensity of the direct X-ray beam while having a wide enough field of view to capture both direct and reflected beams from surfaces several meters away.

We will only list here the detectors available on the multipurpose diffractometer. Other detectors are available on the beamline but specialized for use in the in-vacuum diffractometer (Ciatto *et al.*, 2019 ▸). A Pilatus3 1M (Dectris, Switzerland) is used in most applications requiring a 2D detector and for GIXD in combination with a Soller collimator (see Section 3.4). The 2D detector UFXC, the result of a recent (ongoing) development by the SOLEIL detector group (Koziol *et al.*, 2018 ▸; Dawiec *et al.*, 2019 ▸), is used instead of the Pilatus3 1M for X-ray reflectivity (XRR), taking advantage of its smaller image and pixel size, and its higher dynamic range. When required, the intensity of the incident beam is measured for normalization by an IC PLUS 50 ion chamber as described in Section 2.4. Two silicon drift detectors (SDDs) coupled to a four-channel xMAP Digital X-ray Processor (XIA, USA) are available to measure X-ray fluorescence (XRF) or X-ray absorption spectroscopy (XAS). A first single-element SDD, XFlash 430M (Bruker, Germany), can be mounted on several of our sample environments due to its thin collimation tube and low weight. A second, larger, four-element SDD, XFlash QUAD 5040 (Bruker, Germany), is used for low fluorescence signals requiring a larger sensitive area, of particular importance for measurements at solid–liquid interfaces (Malloggi *et al.*, 2019 ▸).

## 3. Sample environments and associated scientific examples

Here we describe sample environments available on the SIRIUS beamline that are of interest to the soft matter community, together with a selection of associated scientific examples.

### 3.1. In-helium chamber for GIWAXS and GISAXS on solid samples

Since its opening to users, SIRIUS has offered GIWAXS and GISAXS techniques for solid samples. Prior to 2021, two main sample environments were available depending on the desired temperature range, vacuum needs and compatibility, and potential exposure to solvent vapour: a ‘Baby’ chamber with a beryllium dome and equipped with a high-temperature oven (1000°C) (Ciatto *et al.*, 2016 ▸) or a chamber with Kapton windows on its facade (Fontaine *et al.*, 2014 ▸). However, both setups had a significant portion of the beam travelling in air from the beamline exit window to the chamber and then from the chamber to the detector. This, in addition to the numerous Kapton or beryllium windows traversed, led to a spurious background that had to be carefully accounted for in data analysis (Galerne *et al.*, 2021 ▸).

**Figure 4:**
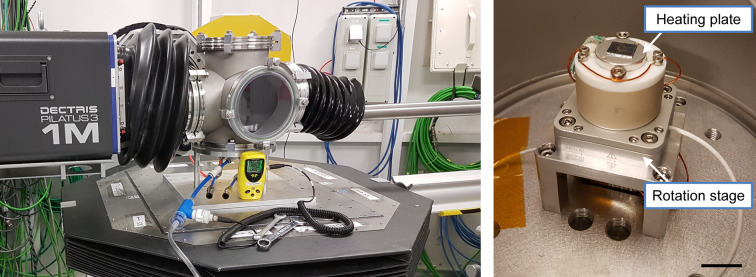
Left: helium chamber in GIWAXS configuration. The chamber is directly connected to the beamline and the Pilatus detector via gas-tight bellows. A 200 µm-thick beryllium window in the inlet tube isolates the setup from the beamline vacuum. An oxygen sensor connected to an outlet of the chamber indicates the percentage of oxygen remaining in the chamber. Right: inside of the helium chamber with the rotation stage and the heating plate in place. The bar is 1 cm.

Following an increasing demand of GIWAXS *in situ* and *operando* measurements on samples with sometimes complex geometries and low diffraction signal, we have developed a custom environmental chamber, initially dedicated to GIWAXS experiments on solid samples. It allows helium to be flushed along the whole beam path with a direct connection to the detector and the beamline using gas-tight plastic bellows, and a single beryllium window upstream to preserve the beamline vacuum (see Fig. 4 ▸).

The new chamber configuration significantly improved the signal-to-noise ratio, making it easier to observe sample features immediately after acquisition and to subtract the background only later on.

**Figure 5:**
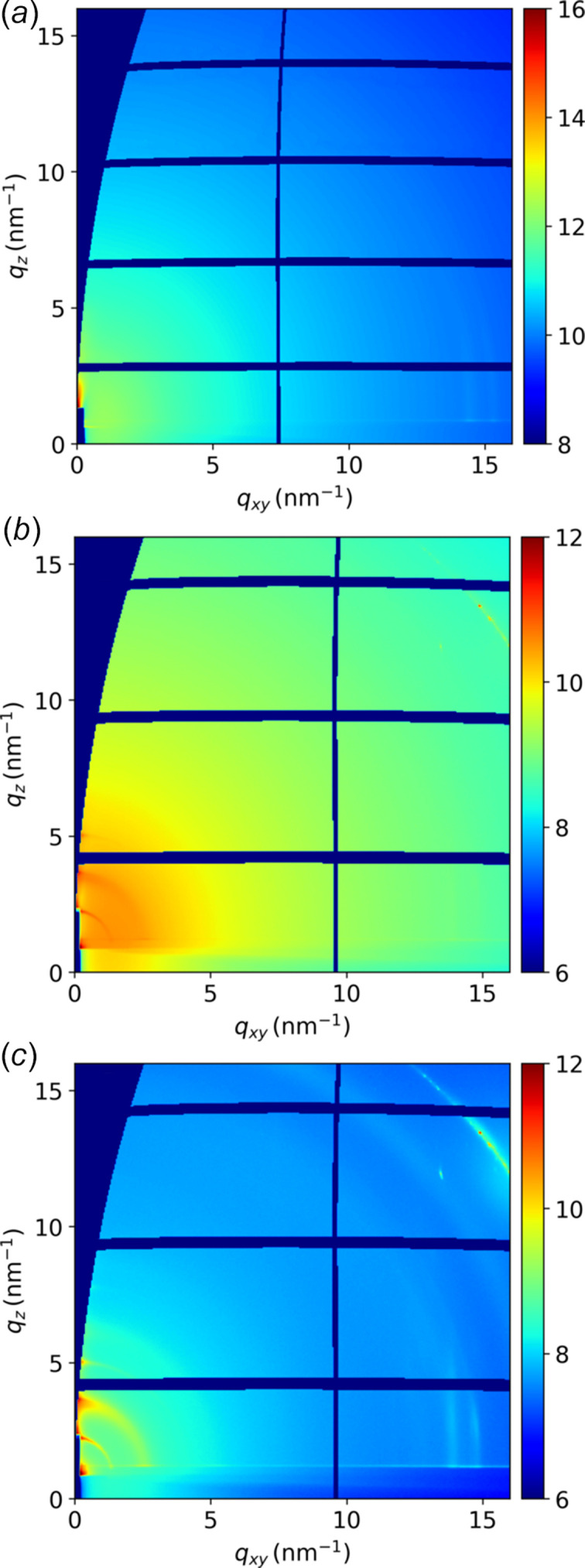
GIWAXS images with a log intensity scale of a conjugated polymer thin film on bare silicon, taken under different conditions: (*a*) without a tube between the exit window and the detector, (*b*) with the new GIWAXS setup but filled with air, and (*c*) with the new GIWAXS setup filled with helium gas.

Figure 5 ▸ shows different GIWAXS patterns of thin films of ∼45 nm thickness of a semiconducting polymer called PF_2_ (Olla *et al.*, 2019 ▸) on bare silicon wafer, taken under various conditions at a beam energy of 10 keV. In Fig. 5 ▸(*a*), the raw images were taken without a tube between the beryllium exit window of the beamline optics and between the sample and the detector, resulting in a few visible rings around 15 nm^−1^ and the clear need for challenging background subtraction, as in particular the low-*q* reflections expected in the 2–5 nm^−1^ range are hidden by the overlapping with the strong air scattering signal. In Fig. 5 ▸(*b*), the raw images were taken with the new setup but filled with air, which revealed more details, especially at low *q* values, due to most of the air scattering on the path between the upstream beryllium window and the sample being captured by the tube. Finally, in Fig. 5 ▸(*c*), the raw images were taken with the new setup filled with helium gas, which revealed many more features without background subtraction. The direct and reflected beam scattering by helium is much lower than with air, making acquisition much more efficient and data analysis easier, particularly when looking for weak diffraction spots and diffuse scattering (Heinrich *et al.*, 2023 ▸).

For GIWAXS measurements, we systematically use the Pilatus3 1M detector with a fixed sample-to-detector distance between 280 mm and 400 mm. Thus, the largest *q*-range accessible in a single image without moving the detector is 0.5–30 nm^−1^, using a beam energy of 12 keV. The diffraction pattern of silver behenate measured in transmission is typically used to calibrate the sample-to-detector distance and to convert the spatial and polar coordinates of the detector into scattering vectors (Nyam-Osor *et al.*, 2012 ▸).

The chamber offers a range of optional features, depending on the experimental requirements. An electrical resistor allows for heating the sample up to 250°C, and the control system can be programmed to implement complex heating patterns synchronized with the scans. Moreover, the sample can be mounted on an SR-4011 rotation stage (SmarAct GmbH, Germany), which provides an angular resolution of less than 2 × 10^−6^ degrees. A microscope (Dino-Lite, France) with magnification ranging from 10× to 140× can be mounted on top or on the side of the substrate, making it especially useful for samples with complex geometries or spatial heterogeneity. XRF measurements can also be taken by connecting one of the SDDs to the side of the chamber with a bellow. Humidity can be controlled and measured within the chamber by flushing the helium in water before entering the vessel, ranging from dry to fully saturated conditions. Finally, when the sample needs to be exposed to hazardous gas or volatile solvents, a small inner chamber with 4.5 µm-thick aluminium windows can be placed inside the main chamber, on top of the rotation and heating stage.

This setup has been commissioned and opened to users in 2020 and is still evolving. For example, a sample changer is currently under development and is expected to be available in 2024. Several GIWAXS studies have already been conducted using this setup, including investigations into the structure change of phthalocyanine-based organic thin-film transistors (OTFTs) (Comeau *et al.*, 2022 ▸; King *et al.*, 2023 ▸), the self-assembly of poly(3-hexylthiophene) (Hicks *et al.*, 2022 ▸) and the structural characterization of titanium oxide nanowires for optoelectronics (Heinrich *et al.*, 2023 ▸).

### 3.2. GISAXS capabilities on SIRIUS

**Figure 6:**
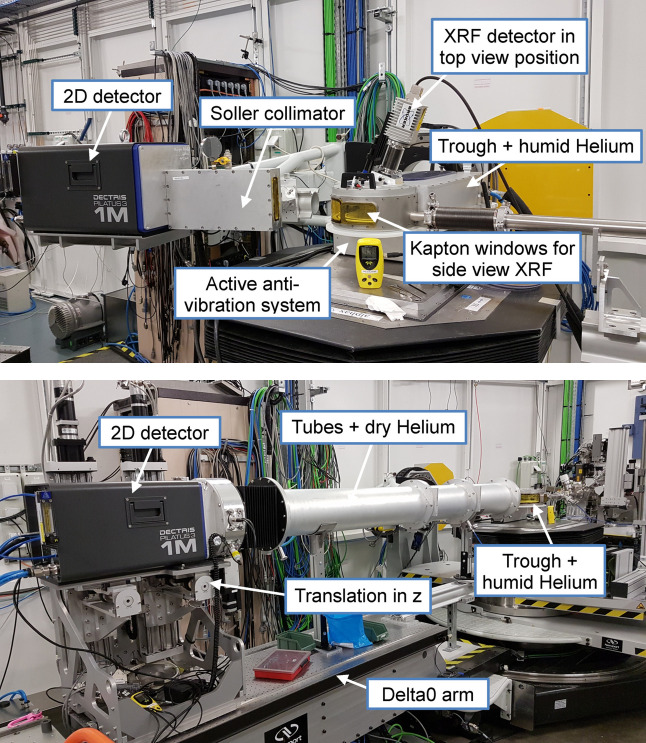
Top: Langmuir trough for experiments at the liquid–air interface mounted on the six-axis tower of the multipurpose diffractometer, enabling combined GIXD and XRF measurements. Bottom: sample deposited on the Langmuir trough with GISAXS tubes in place.

Since opening to users, the capabilities of the beamline for GISAXS experiments have been enhanced for both solid surfaces and air–water interface studies. The sample environment described in Section 3.1 was originally designed and optimized for GIWAXS experiments, but it can also be used in the GISAXS configuration, without any Kapton window connecting the chamber to the series of tubes that are filled with helium and placed between the detector (the Pilatus3 1M) and the sample. We are routinely using a series of beamstops consisting of 2.5 mm-wide stainless steel rods of different heights (30, 40 and 70 mm) with a rectangular cross-section and an engraved V-shape on the side that intercepts the usually 0.5 mm-wide X-ray beams (direct and reflected) and any related spurious scattering at low *q* values. The sample-to-detector distance can be varied between 1.3 m and 4.5 m by varying the dimensions of the tubes (see Fig. 6 ▸), allowing the minimum *q* value that can be reached to go down to 0.01 nm^−1^ using 7 keV X-rays.

This setup has been used to study organic or mixed organic/inorganic layers on solid substrates, as for a recent example to investigate the registration fields for the formation of block copolymer structures using a layer-by-layer approach (Demazy *et al.*, 2023 ▸). On liquid surfaces, the maximum distance combined with the use of an 8 keV X-ray beam has enabled measurement of the organization of MOFs spread as a monolayer at the air–water interface. The measurement revealed a hexagonal structure of the MOFs with the 01 and 11 peaks appearing at 0.0375 nm^−1^ and 0.067 nm^−1^, respectively, corresponding to a parameter of 232 nm (Andrés *et al.*, 2021 ▸).

### 3.3. Langmuir trough for coupling of *in situ* measurements on films at the liquid–air interface

Experiments on Langmuir films, consisting of one or several layers of amphiphilic molecules at the interface between a liquid and a gas, comprise one-third of the proposals performed on SIRIUS. These studies usually complement laboratory measurements at the sub-micrometre scale and are conducted on a dedicated Langmuir trough made of polytetrafluoroethylene (PTFE), which can be mounted on the multipurpose diffractometer (see Fig. 6 ▸). The trough has a total area of 700 cm^2^ and holds a typical subphase volume between 400 and 450 mL. The area per molecule is controlled with a single motorized PTFE barrier with a total compression ratio of 3.8. The surface tension of the liquid–air interface is measured by a Wilhelmy paper plate connected to a Riegler and Kirstein GmbH (Germany) sensor with an accuracy of 0.1 mN m^−1^. The area is controlled by analogue electronics from the same provider, which also enables feedback on the surface pressure. The pressure feedback setpoint can be adjusted locally on the electronics or remotely from the TANGO control system, allowing scripting of layer compression and X-ray scans. Temperature control is achieved by circulating water inside the PTFE trough support, which is connected to an external water bath. This allows for precise temperature regulation within the trough, with the temperature range adjusted so far between 5°C and 45°C. Data acquisition and storage are performed through the TANGO control system of the beamline, either independently or together with X-ray data measurements.

The trough is enclosed in a gas-tight box, flushed with helium that is saturated with water vapour to avoid evaporation of the sub-phase. The helium atmosphere is not only necessary to reduce background scattering at typical working energies of 6–10 keV but is also of crucial importance to reduce beam damage to organic layers. The trough is directly connected to the beamline tube by means of a beryllium window located 800 mm upstream, far enough to avoid wide-angle scattering of the window from reaching the sample or the detector.

This setup has been used in several configurations, such as GIXD, GISAXS, X-ray fluorescence in total reflection (TXRF), and off-specular scattering measurements (sometimes referred to as GIXOS). GIXD is preferably performed with the Pilatus3 1M detector with a Soller collimator to fix and improve the angular resolution (see Section 3.4). Two sets of Soller collimators are available, one with a resolution of 0.025° FWHM when high resolution is required (El Haitami *et al.*, 2018 ▸; Fontaine *et al.*, 2019 ▸), and another of 0.07° FWHM for wider diffraction peaks. These resolutions may slightly change depending on the sample-to-slit distance. TXRF can be measured in parallel with diffraction by placing the single-element SDD above the deposited layer, at a 30° angle with respect to the surface (backscattering geometry) to minimize the contribution of the elastic scattering. The four-element SDD can be used instead, but on a separate arm on the side of the trough. The information provided by X-ray fluorescence in complement to diffraction can be very valuable, in particular to probe the kinetics of absorption/desorption of chemical species at the interface (Andrés *et al.*, 2021 ▸). GIXOS is a very promising and powerful tool for probing both the in-plane and out-of-plane structure of a thin film at the air–water interface. Typically, the incident beam is maintained below the critical angle of total reflection of the interface, and a 2D detector equipped with one or two guard slits captures the scattered signal at an azimuthal angle of the order 0.2° to 1° (Dai *et al.*, 2011 ▸). As in X-ray reflectivity, GIXOS curves exhibit a pronounced dependence on the electron density profile of the interface, offering very valuable insights into the vertical structure of the sample (Mora *et al.*, 2004 ▸; Dai *et al.*, 2011 ▸). Moreover, it provides the potential to monitor dynamic processes in time-resolved experiments (Dai *et al.*, 2013 ▸). Initial experiments utilizing GIXOS were recently conducted on the beamline. However, a significant challenge arose in the precise analysis of the experimental data. This complexity comes from the intrinsic dependence of GIXOS data on the fluctuation spectrum of the interface (Sinha *et al.*, 1988 ▸; Mora *et al.*, 2004 ▸) and the details of the experimental geometry (Dai *et al.*, 2011 ▸). Even the basic task of accessing the electron density profile necessitates meticulous modelling of the GIXOS intensity, rendering it more intricate to employ compared with X-ray reflectivity, and is the subject of current work of the beamline staff to render it more accessible to the user community. Finally, the Langmuir trough can be included in the GISAXS setup (see Section 3.2 and Fig. 6 ▸).

**Figure 7:**
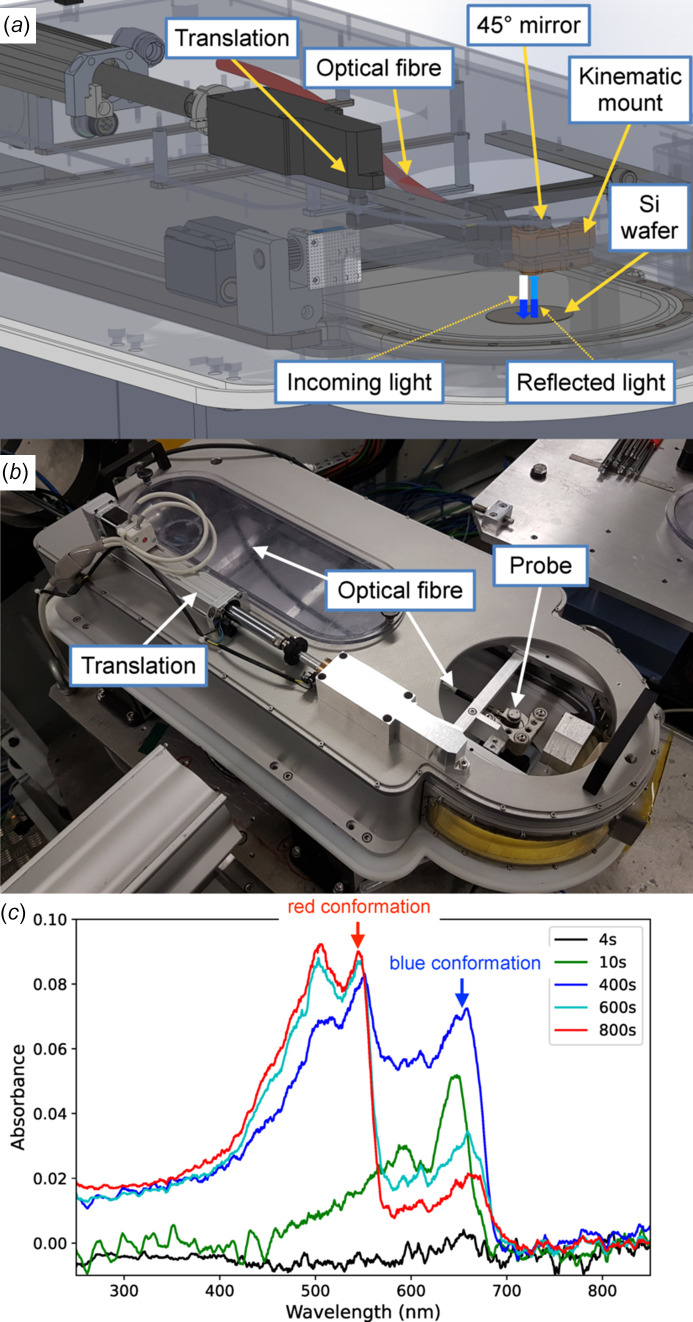
*In situ* UV–visible spectroscopy integrated to the Langmuir trough. (*a*) Schematic diagram, showing the double absorption of the light by the layer after reflection on a Si wafer. (*b*) Annotated picture of the setup. (*c*) Absorbance spectra of a PCDA Langmuir film under continuous UV irradiation as measured with the setup shown in (*a*) and (*b*).

Complementary *in situ* measurements conducted in parallel with the X-ray acquisitions can greatly enhance the understanding of systems studied at the liquid–air interface, as the samples cannot be further characterized with laboratory equipment before or after the beam time. We therefore equipped the trough with a UV–visible spectrometer, in a transmission geometry [see Figs. 7 ▸(*a*) and 7(*b*)]. The setup comprises a bifurcated optical fibre (Thorlabs SAS France, RP20 Reflection Probe, High-OH, SMA Connectors) and an Al-coated mirror (Thorlabs SAS France, SMA905 aluminium reflective collimator) mounted on a kinematic mount (Thorlabs SAS France, Solaris K1) that illuminates the film with a parallel beam of white light. The light source is a DH-2000-BAL from Ocean Insight (Germany–Netherlands) with a halogen and a deuterium lamp providing light between 210 and 1500 nm. The optical fibre is positioned on top of the layer, and the light passes through the interface twice after reflecting on a silicon wafer at the bottom of the trough. The reflected light is directed back into the fibre, which carries it towards a UV–visible spectrometer. Generally, users may bring their own spectrometer if it is compatible with the setup (mainly SMA connectors), and a dedicated spectrometer (Avantes AvaSpec ULS2048XL-EVO) is available on the beamline. The entire setup is mounted on a horizontally translating platform perpendicular to the beam, as shown in Figs. 7 ▸(*a*) and 7(*b*). This allows the apparatus to track any displacement of the trough that may occur when changing the X-ray illuminated area using the diffractometer translation. It also ensures that the area observed by the spectrometer remains focused on the X-ray illuminated region. Additionally, the motorized platform facilitates the cleaning of the whole trough, as it allows the probe to be moved away to access all parts of the trough.

As an example of an ongoing study using the *in situ* UV–visible setup, Fig. 7 ▸(*c*) shows the absorbance spectra of a 10,12-pentacosadyionic acid (PCDA) tri-layer at room temperature at the air–water interface under continuous UV irradiation, measured with an AvaSpec ULS2048XL-USB2 spectrometer (grating UA 200 nm–1160 nm, slit of 25 µm; Avantes, Netherlands). There is currently a significant research effort focused on the development of biological and chemical sensors using diacetylene molecules (Reppy & Pindzola, 2007 ▸; Rego *et al.*, 2021 ▸). These compounds (*R*—C≡C—C≡C—*R*′, where *R* and *R*′ are substituent groups) polymerize under UV irradiation in a topochemical manner, typically resulting in a blue polymer that transitions to a red form under different stimuli such as changes in pH or temperature, as well as during the irradiation. To be suitable for practical applications, polymerization must be stopped before the red form appears. However, the mechanism underlying the blue-to-red transition is not yet fully understood. The objective in this study is to investigate the link between colour and structure by simultaneously measuring the diffraction and UV–visible spectra.

Finally, the microscope described in Section 3.1 can be set in place of the spectrometer, above the liquid surface, to record the morphological transformations of a layer, such as the formation of large domains or a change of colour and opacity, for example in the case of large crystals formation or X-ray radiolysis.

### 3.4. Classic, single-slit and slit-less GIXD

**Figure 8:**
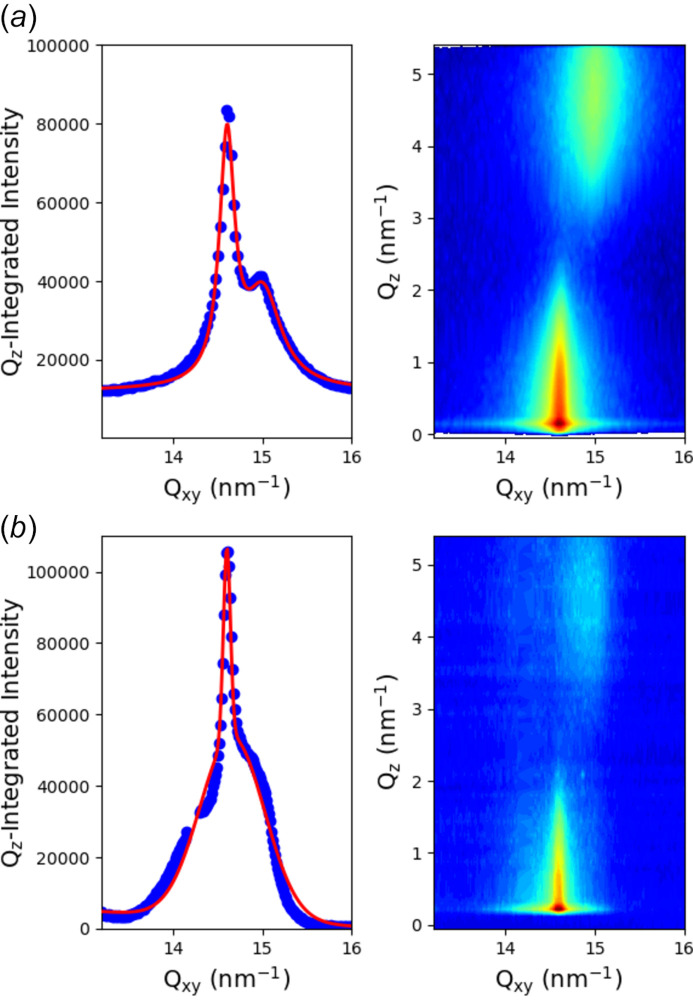
GIXD spectra measured on a Langmuir monolayer of behenic acid compressed at 20 mN m^−1^ (*a*) with the classical setup of a 2D detector with a Soller collimator, (*b*) with the single vertical slit and 2D detector configuration (sample-to-detector distance: 770 mm; sample-to-slit distance: 240 mm; slit horizontal gap: 1 mm). Red lines are fits of the experimental data points by two Lorentz functions in (*a*) and Gaussian functions in (*b*), as discussed in the text, and both with a linear sloping background. The two images do not have the same colour scale due to different binnings in the number of points.

As described in Section 3.3, GIXD at the liquid–air interface is typically measured using the Pilatus3 1M detector with a Soller collimator oriented vertically in front of it (see Fig. 6 ▸). The Pilatus detector is used, in fact, as a 1D detector by integrating 2D images along the horizontal axis at each angle of the diffraction scan. The full diffraction spectrum is obtained by continuously scanning along the 2θ angle in the interface plane and at different discrete values of the out-of-plane angle for the detector. Continuous scanning is preferred to avoid sample perturbation induced by vibrations in step-by-step mode, in particular when probing liquid surfaces. This procedure also presents the advantage of reducing the dead-times induced by the sleep times following each movement in a step-by-step scan. In such a continuous scan, the motor is slowly moved between the two extreme positions of the scanning angular range as the data are recorded on-the-fly. The speed of the motor is determined by the ratio of the angular range and the product of the number of points and counting time, with a correction factor applied to account for acquisition dead-times. A custom *Jupyter Notebook* is used to reduce the data and assemble scans at different values of the out-of-plane angle (see Section 4). This process yields an intensity map *I*(*q*_*xy*_, *q*_*z*_), where *q*_*xy*_ denotes the in-plane component of the scattering vector and *q*_*z*_ its vertical component. The result of such a procedure is given in Figs. 8 ▸(*a*) and 10(*a*).

At a given value of the out-of-plane angle, the time required for a scan between 12 and 16 nm^−1^, which is the typical range for amphiphilic molecules in a monolayer, is about 15 min. For some systems, the scanning time can be reduced to a few minutes by reducing the sampling of *q*_*xy*_ and counting times. However, fast kinetic measurements with a characteristic time scale of less than a minute cannot be performed with this configuration. Nevertheless, some studies require much faster acquisition times, such as the study of the early stages of the X-ray surface radiolysis process (Mukherjee *et al.*, 2015 ▸), as well as when beam damage occurs quickly, such as Langmuir films on divalent salts aqueous solutions (El Haitami *et al.*, 2018 ▸). Even if measurements were successfully performed on the SIRIUS beamline using this former strategy, acquisition times below 1 min for a full spectrum measurement are necessary.

Two different geometries could be implemented on the SIRIUS beamline to enable such quick GIXD acquisition. The first one is based on a concept that we previously introduced on the X-ray liquid surface diffraction beamline (‘difliq’) at LURE, the former French national synchrotron source in Orsay, France (Fontaine *et al.*, 2004 ▸). This setup uses a two-dimensional detector combined with a single vertical slit (horizontal gap) and enables the measurement of the diffraction pattern of a 2D powder monolayer with an equivalent in-plane resolution and a faster acquisition time than the usual setup. Additionally, the resolution versus flux and the *q*_*xy*_-range can be easily adjusted through the choice of the sample–slit–detector distances. This geometry was first introduced by some of the authors (Fontaine *et al.*, 2004 ▸) and has since been successfully implemented on other facilities (Meron *et al.*, 2009 ▸; Banerjee *et al.*, 2015 ▸).

Figure 8 ▸(*a*) shows a diffraction spectrum taken in 15 min with the scanning configuration using the Pilatus detector in combination with the Soller collimator of medium resolution (0.07°) on a behenic acid Langmuir monolayer compressed at 20 mN m^−1^ on pure water. It shows the classical two-peaks pattern of the L2 phase of fatty acids Langmuir monolayers with the 11 peak maximum located out-of-the-plane and the 02 peak maximum located in-plane as expected for next-neighbour tilt of the molecules (Kenn *et al.*, 1991 ▸). In Fig. 8 ▸(*b*), the same pattern was measured using the GIXD geometry with the 2D detector at 770 mm from the sample and a single vertical slit located at 240 mm from the sample, 1.0 mm horizontal gap, with a counting time of 5 min, and is shown here after subtraction of scattering by the surface of pure water. The two spectra showed the same diffraction pattern with one in-plane and one out-of-plane peak as expected, and only differ by the shape of the peaks. Even if the peak positions were roughly identical using the measured parameter values, a slight optimization of the angle of the detector arm with respect to the direct beam position is necessary using the known spectra of behenic acid at 20 mN m^−1^. This adjustment is primarily needed due to the limited resolution in aligning the detector arm with the direct beam, which typically falls within the range of a few tenths of a degree. As we have previously demonstrated, the resolution in the second geometry is controlled by the gap of the horizontal slit, and the sample–slit and sample–detector distances (Fontaine *et al.*, 2004 ▸). According to the theoretical resolution calculation provided in the Appendix of Fontaine *et al.* (2004 ▸), the *q*-resolution is around 0.075 nm^−1^, which is nearly identical to one of the Soller collimator configurations. However, the resolution functions of the two configurations are not identical, which could explain the slight difference observed in the peak shapes. The experimental data points in the latter geometry are fitted using a Gaussian function, which can be rationalized given the different resolution functions of the two configurations. It is worth noting that the main characteristics of the diffraction pattern can already be observed in a few tens of seconds.

**Figure 9:**
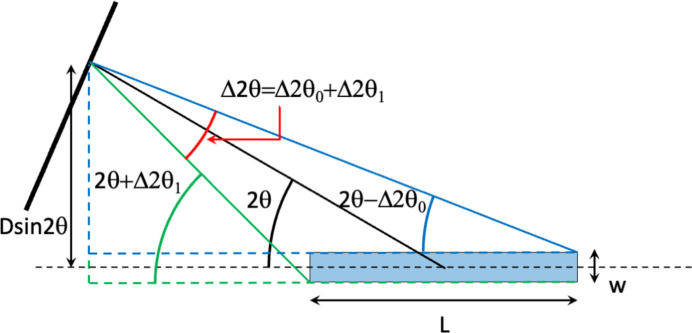
Top view of the GIXD experiment without collimation system before the 2D detector with a footprint of size *L* × *w* and the different angles to determine the resolution of the measurement at a 2θ angle.

Small beams are now more easily available on synchrotron beamlines, thanks to the possibility of focusing X-rays on few-micrometre spots. This trend will be reinforced in the near future with the advent of the so-called diffraction-limited storage rings (DLSRs). Although at first sight such development does not appear particularly interesting for the study of layers at the air–water interface, a new paradigm for the acquisition of the diffraction signal from a liquid surface with a symmetrical and smaller beam is conceivable. Indeed, if the beam size is sufficiently small, we can reach a footprint size for which the illuminated sample dimensions are negligible (at least small) with respect to the sample-to-detector distance. Thus, if we place a 2D detector at a distance *D* from the sample as in Fig. 9 ▸, we can simply express the horizontal angular resolution of one pixel as 

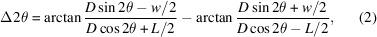

where 2θ is the in-plane detection angle, *w* is the width of the beam, and *L* = 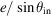 with *e* the vertical thickness of the beam and θ_in_ the incidence angle.

**Figure 10:**
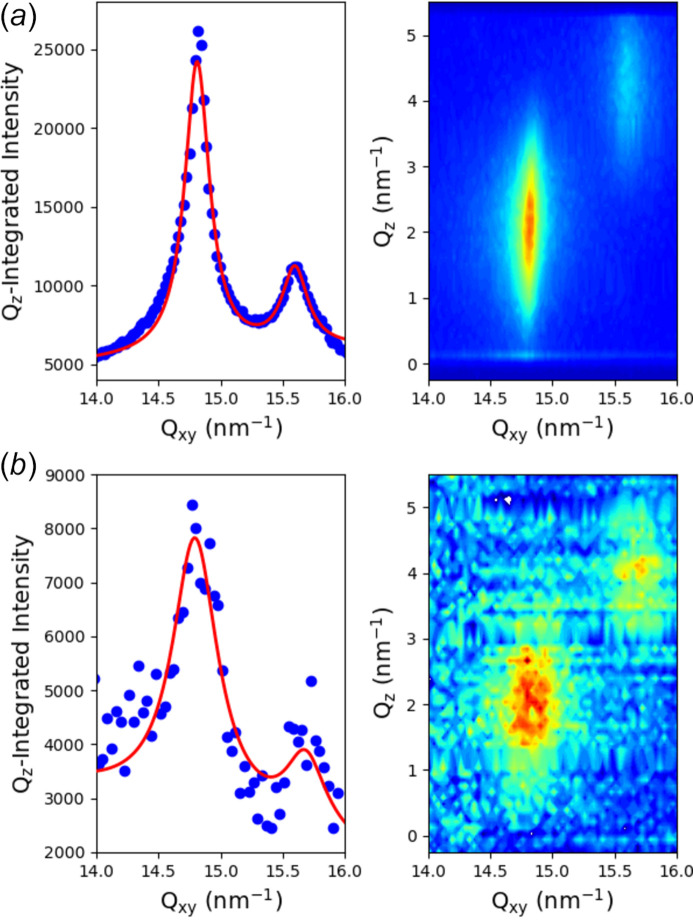
GIXD spectra measured on a Langmuir monolayer of behenic acid compressed at 25 mN m^−1^ (*a*) with the classical setup of a 2D detector with a Soller collimator, and (*b*) without any collimation system before the detector but with a 15 µm × 24 µm (H × V) beam. The detector is located 750 mm from the centre of the sample. The red lines are fits by two Lorentz functions and a linear sloping background.

For a 2D detector at *D* = 750 mm from the sample and a beam of 100 µm × 100 µm, which is the smallest size currently achievable at the SIRIUS beamline (without losing flux or using the transfocator), the angular resolution would be 1.4° at 2θ = 20°, almost two orders of magnitude larger than the best resolution achievable with the Soller collimator (see Section 3.4). However, if we reduce the vertical beam size to 20 µm with the transfocator, the angular resolution becomes 0.26°, still above the resolutions of our two sets of Soller collimators but more reasonable for envisioning a GIXD measurement. We tested this simple geometry at 8 keV on a Langmuir film using the Pilatus3 1M detector at a distance of *D* = 750 mm from the centre of the diffractometer and with a beam focused at 15 µm × 24 µm (H × V) by the transfocator. Figure 10 ▸(*b*) presents the result after 5 min of acquisition. Compared with the pattern in Fig. 10 ▸(*a*), the measured spectrum has similar features and peak positions to those obtained with the other two setups. It displays the characteristic features of the L2′ phase of fatty acid monolayers, with two out-of-plane peaks at *q*_*z*_-positions in a ratio of two, as expected for a next-nearest-neighbour tilt of the molecules (Kenn *et al.*, 1991 ▸). However, in comparison with the spectra measured with the two previous geometries shown in Fig. 8 ▸, the overall quality is lower. The signal-to-background noise is smaller, approximately 1, as opposed to 2.5 for the Soller collimator measurement. The high level of background noise can be attributed to the beam travelling through helium gas before the sample and through air after reflection. This results in spurious scattering that, in this geometry, is not blocked by any mask. For a future development of this geometry, it is imperative to establish a direct connection between the Langmuir trough and the detector, employing a design similar to the one described for GIWAXS in Section 3.1. Besides, the peak widths are larger (0.2 nm^−1^ compared with 0.1 nm^−1^ for the spectra measured with the Soller collimator). Further reducing the beam size would enhance the resolution, *e.g.* at 1 µm × 1 µm the resolution reaches 0.01°.

Nevertheless, this latest geometry using a small beam for GIXD raises some questions for future use besides the high density of photons that could induce sample degradation. While for conventional amphiphilic molecules such as fatty acids, alcohol or lipids the 2D powder distribution of the samples is usually satisfying when considering the beam size (and more precisely the footprint), new studies in the field of layers at the air–water interface have revealed inhomogeneities of similar or larger sizes than the beam’s footprint. These features can typically be observed with Brewster angle microscopy, in systems such as behenic acid monolayers on cadmium salt solutions (Cantin *et al.*, 2004 ▸) and monolayers of DPPC and cholesterol mixture in contact with digitonin (Wojciechowski *et al.*, 2016 ▸), for example. Thus, when using this new approach for GIXD on such samples, these inhomogeneities will have to be taken into account during analysis. Nevertheless, this geometry could offer a means to address the structural determination of the domains. On forthcoming DLSRs, where the beam is anticipated to be more coherent, the crystal sizes or structural correlation lengths may be of the same order of magnitude as the beam’s coherence length. As such, this may therefore pose a challenge in future experimental measurements and analysis, but it could also provide novel opportunities.

In this context, the SIRIUS beamline gives access to a range of detection setups and beam shapes allowing to vary the diffraction data collection at the air–water interface and tailor our approach to the needs of the systems and of the users. While the two first geometries described here, with a Soller collimator or a single slit, can be immediately used depending on their respective advantages, the third one can be seen as a prospective option, especially with the development of small beam capabilities on beamlines.

## 4. *JupyLabBook*: a customized electronic notebook

**Figure 11:**
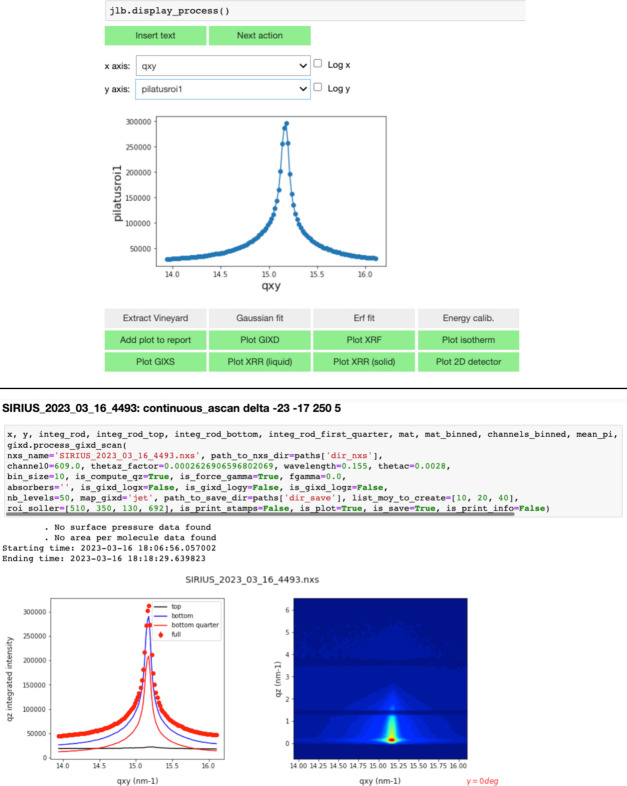
Screenshots of *JupyLabBook*. After selecting the scan, the user can view the data included in the Nexus file and decide how the data should be processed. Top: quick visualization of a GIXD scan and widgets for selection of an appropriate data treatment. Bottom: notebook cell after full data extraction and conversion to *q*-space.

Since mid-2020, SIRIUS users have had access to a custom electronic notebook. This interactive notebook, called *JupyLabBook*, is based on a *Jupyter Notebook* core (https://jupyter.org/) enriched with several widgets (see Fig. 11 ▸). Designed as a low-code tool, users unfamiliar with programming can use it without writing a single line of code, after setting up of the notebook by the beamline staff, while an expert user can easily enrich it with Python code. The notebook allows users to quickly reduce and visualize data once a scan is complete, and to display the results in a clear and structured manner with headings and comments. Most of the experimental techniques present on the beamline can now be processed in the notebook, such as GIXD, GIWAXS, GISAXS, XRF, XRR and Langmuir isotherms. The alignment process and all the calibration parameters are reported at the beginning of the notebook file, and used for data reduction throughout the experiment.

*JupyLabBook* converts diffraction scans into reciprocal space, represented as a TIFF image and raw ASCII files, and fluorescence scans into individual energy-intensity spectra in the form of text files, and spectrograms as images. X-ray reflectivity data on solids can also be extracted as ASCII files using the notebook by integrating the 2D detector, performing proper background subtraction and normalizing by the incident beam. The notebook is linked to the log files of SPyC, an IPython-based control and acquisition system developed at SOLEIL within the TANGO framework, enabling automatic transfer of most of the information related to the scan. For users who prefer not to display graphs and figures for each scan, the notebook allows importing only the sequence of commands typed into SPyC, similar to what was previously recorded in paper notebooks. Once the experiment is complete, the notebook can be converted into a single PDF file that includes all the relevant information.

The open-source code of *JupyLabBook* is hosted on the SOLEIL GitLab repository (https://gitlab.synchrotron-soleil.fr/sirius-beamline/notebooks/JupyLabBook) and version 3.0.2 is provided here in the supporting information. While it was specifically developed for the SIRIUS beamline, the code has been structured in a way that allows for potential adaptation to other beamlines, but without support of SIRIUS staff.

## 5. Conclusion

Since its opening to users in 2013, the SIRIUS beamline has hosted a wide range of experiments relevant to the soft condensed matter community. Researchers have investigated a variety of systems, including, among others, the behaviour of amphiphilic molecules, polymers, colloids and electrolytes, at liquid or solid interfaces. The results of these experiments are of potential interest to fields such as functional materials, energy storage and biology. In this article, we have recalled the main characteristics of the SIRIUS beamline and presented a selection of recent developments that illustrates the growing need in the community for fast experiments with beams of tunable sizes, in combination with a number of *in situ* techniques. In this context, the SIRIUS beamline complements other similar facilities in terms of energy range and experimental techniques. These include I07 at Diamond (UK) (Nicklin *et al.*, 2016 ▸), P03 and P08 at Petra III (Germany) (Buffet *et al.*, 2012 ▸; Shen *et al.*, 2022 ▸), ChemMat-Cars at APS (USA) (Lin *et al.*, 2003 ▸) and ID10 at ESRF (France) (Smilgies *et al.*, 2005 ▸; Jankowski *et al.*, 2023 ▸), all of which we share many users with. In our future projects, we plan to improve our soft matter sample environment by integrating complementary characterization techniques alongside X-ray measurements. For example, we intend to incorporate a Brewster angle microscope into our Langmuir trough, and investigate the application of small beams and X-ray beam coherence to soft matter systems using our transfocator. Furthermore, we aim to take advantage of the capabilities of the SIRIUS beamline for X-ray absorption spectroscopy and resonant or anomalous scattering techniques applied to soft interfaces, including the tender X-ray energies (1–4 keV). To achieve these goals, we could utilize the MGM monochromator and the windowless in-vacuum diffractometer for low-energy resonant GISAXS and GIWAXS studies, as well as absorption spectroscopy at the sulfur *K*-edge (2473 eV), for example. This would constitute a significant advancement in the exploration of organic materials, particularly in multicomponent systems, as demonstrated by the work of Coric *et al.* (2018 ▸) on resonant GISAXS work on P3HT:PCBM [poly(3-hexylthiophene-2,5-diyl):phenyl-C61-butyric acid methyl ester].

## Supplementary Material

Source code of JupyLabBook v3.0.2, a custom electronic notebook based on Jupyter Notebook. DOI: 10.1107/S1600577523008810/ay5620sup1.zip

## Figures and Tables

**Table 1:** Positions of the components displayed in Fig. 1 ▸

Index	Distance from source (m)	Component
1	0	HU36 undulator
2	17.00	Primary slits
3	18.50	Double-crystal monochromator
4	20.00	Multilayer grating monochromator
5	21.10	Secondary source slits
6	22.00	Mirror M1 (harmonic rejection)
7	26.00	Mirror M2 (vertical focusing)
8–9	27.50–30.00	Optical slits
10	31.20	Mirror M3 (horizontal focusing)
11	32.80	Mirror M4 (deflection)
12	33.70	Anti-scattering slits
13	34.50	Compound refractive lenses
14	35.00	Seven-circle diffractometer
–	0.28–4.50	Position of area detector (relative to a sample on the seven-circle diffractometer)

**Table 2:** Optimized configuration of the CRLs used with the SIRIUS transfocator, as a function of beam energy *N* is the total number of CRLs inserted in the beam. *T* and *f* are the transmissions and the focal lengths of the transfocator.

Energy	Pack 1	Pack 2	Pack 3	Pack 4	Pack 5			
(keV)	2 CRLs	4 CRLs	6 CRLs	6 CRLs	6 CRLs	*N*	*T*	*f* (m)
4	Yes	No	No	No	No	2	14%	0.58
5	No	Yes	No	No	No	4	14%	0.46
6	No	No	No	No	Yes	6	17%	0.44
7	Yes	No	No	No	Yes	8	20%	0.45
8	No	Yes	No	No	Yes	10	23%	0.47
9	No	No	Yes	No	Yes	12	25%	0.50
10	Yes	No	Yes	Yes	Yes	14	28%	0.52
11	No	Yes	Yes	Yes	No	16	30%	0.55
12	No	Yes	Yes	Yes	Yes	22	26%	0.48

**Table 3:** Main features and uses of the detectors available on the multipurpose seven-circle diffractometer

Detector	Characteristics	Use
Ion chamber	Filled with N_2_ or Ar	Intensity normalization
X-ray camera	Spatial resolution: 35.5 µm	Beamline and sample alignment
	Detection area: 50 mm × 40 mm	
Pilatus3 1M	2D detector with hybrid-pixel technology	GISAXS, GIWAXS, GIXD, off-specular reflectivity
	Pixel size: 172 µm × 172 µm	
	Number of pixels: 981 × 1043	
	Linear response: up to ∼10^5^ photons pixel^−1^ s^−1^	
UFXC	2D detector with hybrid-pixel technology	XRR
	Pixel size: 75 µm × 75 µm	
	Number of pixels: 256 × 257	
	Linear response: up to ∼10^6^ photons pixel^−1^ s^−1^	
XFlash 430M	One-element silicon drift detector	XRF, TXRF, XAS
	Energy resolution: 133 eV	
	Sensitive surface: 30 mm^2^	
XFlash QUAD 5040	Four-element silicon drift detector	XRF (mostly solid–liquid interface), XAS
	Energy resolution: 135 eV	
	Sensitive surface: 4 × 10 mm^2^	
