# Supplementary material for: Opportunities and new developments for the study of surfaces and interfaces in soft condensed matter at the SIRIUS beamline of Synchrotron SOLEIL
Source: J Synchrotron Radiat. 2024 Jan 1;31(Pt 1):162–76. doi: 10.1107/S1600577523008810 (PMC10833424; doi:10.1107/S1600577523008810)
Supplement: Supplementary file 1 [file s-31-00162-sup1.zip › JupyLabBook-v3.0.2/docs/JupyLabBook_User_Manual.pdf]

Référence : EXP-SIRIUS-MANUAL-P-JupyLabBook_User_Manual

P

JUPYLABBOOK: USER MANUAL

Date de diffusion	Rédacteur	Vérificateur	Approbateur	Modifications
2020/07/13	A. Hemmerle (Beamline Scientist)			Creation for release of v1.0
2023/02/24	AH			Update for v3.0.2
**Destinataires**	SIRIUS Users.			

PUBLIC

La version électronique fait foi.

TABLE OF CONTENTS

1. *Introduction* 4
1.1. What is JupyLabBook? 4
1.2. What is not JupyLabBook? 4
2. *Installation & Start up* 5
2.1. Install Jupyter 5
2.2. Set up crontab 5
2.3. Download the latest version of JupyLabBook 5
2.4. Run JupyLabBook 5
2.5. Set up and run the first cell 6
3. *Panel "Action"* 6
3.1. Fill Form 6
3.2. Calibration theta z 6
3.1. Calibration XRR 6
3.2. Export To PDF 7
3.3. Insert text 7
3.4. Insert script 7
3.5. Insert image 7
3.6. Insert positions 7
3.7. Insert commands 8
3.8. Refresh 8
3.9. Process scan(s) 8
3.10. Save/Load params 9
4. *Panel "Process "* 10
4.1. Next action 10
4.2. Extract Vineyard 10
4.3. Calib. energy 11
4.4. Fit with gaussian/erf 11
4.5. Add plot to report 11
4.6. Plot GIXD 12
4.6.1. Parameters 12

4.6.2. Formula 12
4.6.3. Data output 13

4.7. Plot XRF 14
4.7.1. Parameters 14
4.7.2. Identify peaks 14
4.7.3. Data output 15

4.8. Plot isotherm 16
4.8.1. Parameters 16
4.8.2. Data output 16

4.9. Plot 2D detector 17
4.9.1. Parameters 17
4.9.2. Data output 17

4.10. Plot GIXS 17
4.10.1. Parameters 18
4.10.2. Formula 19
4.10.3. Data output 20

4.11. Plot XRR (liquid) 21
4.11.1. Parameters 21
4.11.2. Formula 22
4.11.3. Data output 22

4.12. Plot XRR (solid) 23
4.12.1. Parameters 23
4.12.2. Formula 24
4.12.3. Data output 24

5. Miscellaneous 25

5.1. Get the scan selection panel back 25

5.2. The notebook has crashed 25

5.3. Modify a text cell 25

5.4. The last scan is missing in the scan list 25

5.5. Delete a cell 25

5.6. Save/copy the notebook 26

5.7. It is impossible to save the notebook 26

5.8. Example 26

1. INTRODUCTION

1.1. WHAT IS JUPYLABBOOK?

JupyLabBook is an interactive lab book to be used on SIRIUS. It is meant to be as close as possible as the paper-like lab book that you would use on the beamline (except for the ruler and the tape).

The user can easily plot raw data, do data reduction, and write sample description without typing a single line of code. A PDF with a linear presentation of the notebook can be generated at any time of the experiment.

Expert users can define their own functions to perform more involved data analysis or presentation of the data, if needed.

The development of JupyLabBook is tracked on GitLab:
https://gitlab.synchrotron-soleil.fr/sirius-beamline/notebooks/JupyLabBook

1.2. WHAT IS NOT JUPYLABBOOK?

JupyLabBook was not designed to be a notebook for **analysis,** but for **data reduction** only. Think of it as the traditional lab book, that should remain untouched after the end of the experiment. Notebooks for specific analysis (GIXS, GIXD, XRF ...) can be provided as well on demand.

JupyLabBook is not made of paper: it can crash, it can be corrupted, it can be deleted by mistake ... In the present state of development, we advise users to keep a written paper-like lab book in parallel with any type of digital notebook. Especially for all the info concerning the samples measured, that cannot be retrieved afterwards.

2. INSTALLATION & START UP

2.1. INSTALL JUPYTER

JupyLabBook works within the framework of the Project Jupyter (https://jupyter.org/). It only requires a proper install of Jupyter, and can therefore work on Windows, Linux, or MacOS. For installing Jupyter on your work station, refer directly to Jupyter website.

Few recommendations:
- Use Python >= 3.8 (see requirements.txt)
- If used on Windows, it requires the installation of MikTex >= 2.9.7 for rendering the notebook as a PDF.
- The packages *lmfit* and *ipysheet* are required and not necessarily provided within the anaconda distribution.

2.2. SET UP CRONTAB

An automatic copy of the logs and scripts should be set up from srv4 to the Ruche. First create a folder `logs/` and a folder `scripts/` in the user's folder on the Ruche.

Then edit the crontab file in a terminal on srv4:

- Type `crontab -e` (works like vi)
- Add two lines (or modify if already there):
  - `*/1 * * * * cp -u /home/experiences/sirius/com-sirius/log/spyc/_*.log /nfs/pathToUser/logs/`
  - `*/1 * * * * cp -u /home/pathToUser/*py /nfs/pathToUser/scripts/`

This will copy the files every minute (only if the files are modified).

2.3. DOWNLOAD THE LATEST VERSION OF JUPYLABBOOK

To download the latest version of JupyLabBook:

- Go to https://gitlab.synchrotron-soleil.fr/sirius-beamline/notebooks/JupyLabBook
- Click Repository/Tags
- Download the latest tagged version and extract the zip file
- Rename the file JupyLabBook.ipynb with experiment name: User_PropNumber.ipynb (for ex.: Doe_20200712.ipynb)

2.4. RUN JUPYLABBOOK

Start Jupyter Notebook in a folder from where you can access your working folder.

Contrary to its name, JupyLabBook cannot be used with Jupyter Lab. It has to be used with Jupyter Notebook.

Find and open the .ipynb file, which should now have the name User_PropNumber.ipynb

2.5. Set up and run the first cell

A few thing must be modified in the first code cell:

- Change the value of `'notebook' : 'JupyLabBook.ipynb'` with your notebook name.
- Change the value of `dir_save` with the full path to your working directory (where the data reduction will be saved).
- Change the value of `dir_user` with the full path to your recording directory (where the nexus files are saved).

Run the first two cells. Check that there is no error message.

If everything worked well, a "Choose action" panel with the nexus files should appear at the bottom of the notebook.

3. Panel "Action"

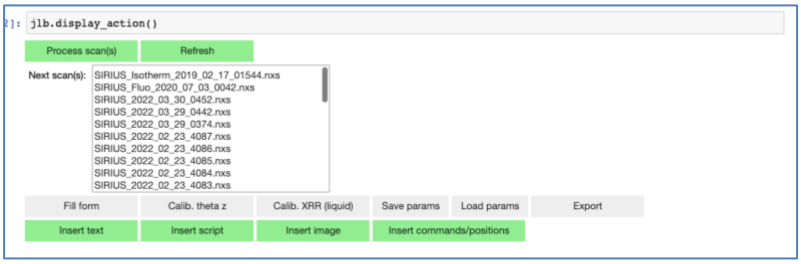

Users can click on the green buttons without asking the staff.

3.1. Fill Form

Load an empty form to be filled at the beginning of the experiment.

When the form is filled, click on "Print form".
The different text cells can be modified afterwards by double-clicking on them.

3.2. Calibration theta z

Do the qz calibration for GIXD.

A code cell is generated with default values.
Change the corresponding values and re-run the cell.
The value of the calibration in radian per channel is stored, and will be automatically used in GIXD.

3.1. Calibration XRR

Do the calibration for XRR.

A code cell is generated with default values.

Change the corresponding values and run the cell.
Help you doing the calibration of m4pitch, zs, c10tablepitch, gamma.

3.2. Export To PDF

Export the notebook into a PDF stored in the same folder as your notebook and convert all the logs from your logs directory into a human-readable format.

New logs are saved in a folder `readable_logs/` in your save directory.
The original logs are kept intact.

3.3. Insert text

Insert formatted text into the notebook.

Write down the text into the box and choose its format (simple text, title, subtitle, or subsubtitle).
When you are done, click on "Insert ".

3.4. Insert script

Insert a script in the notebook.

Choose the script you want to print. If you want an automatic numbering of the scans, put the index of the first scan in the text cell. If not, leave it empty. Click to insert the script.

The script can be modified after import by double clicking on the text cell.

NB: Save the scripts with a different file name each time you modify them.

3.5. Insert image

Insert an image in the notebook.

Removed since v3.0.2

Choose the image you want to insert in the notebook. It will also appear in the PDF.

Note that inserting large images will increase the memory used by the notebook, and thus lower its stability/responsiveness.

3.6. Insert positions

Get the positions in the logs and insert them in a text cell.

This function searches in the logs all the *wm* commands. First select the log file, then the *wm* you want to print.

The text cell can be edited by double-clicking on it.

3.7. Insert commands

Print a series of commands extracted from the logs.

The index of each scan is added after the command. In case of a script, the index of each scan ran by the script is added.

First select the log file, then select one or multiple commands and click on *Insert commands* below the selection window.

To select multiple commands: keep shift pressed while clicking to select a range of commands, keep ctrl pressed while clicking to select the commands individually (cmd on MacOS).

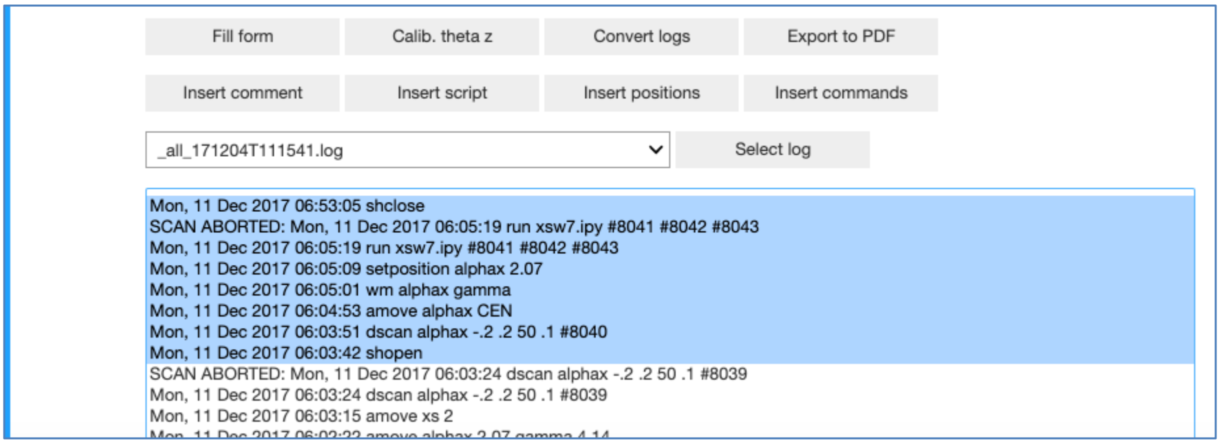

Insert commands with multiple scans selected by keeping shift pressed while clicking.

3.8. Refresh

Refresh the list of nexus files.

Click on *Refresh* if your scan is finished but does not appear in the list.

It does not refresh the list of scripts/logs. These files are automatically updated every minute.

3.9. Process scan(s)

Open the panel Process.

First select a scan in the scan selection window. The command which led to this scan is extracted from the logs and printed below the window for your information. Use it to confirm that the selected scan is the right one, and click on *Process scan(s).*

This will delete the panel "Action", print the command which led to the scan as a subtitle, and open a panel "Process".

It is possible to select multiple scans and apply the same treatment on each of them.

To select multiple scans: keep shift pressed while clicking to select a range of commands, keep ctrl pressed while clicking to select the commands individually (cmd on MacOS).

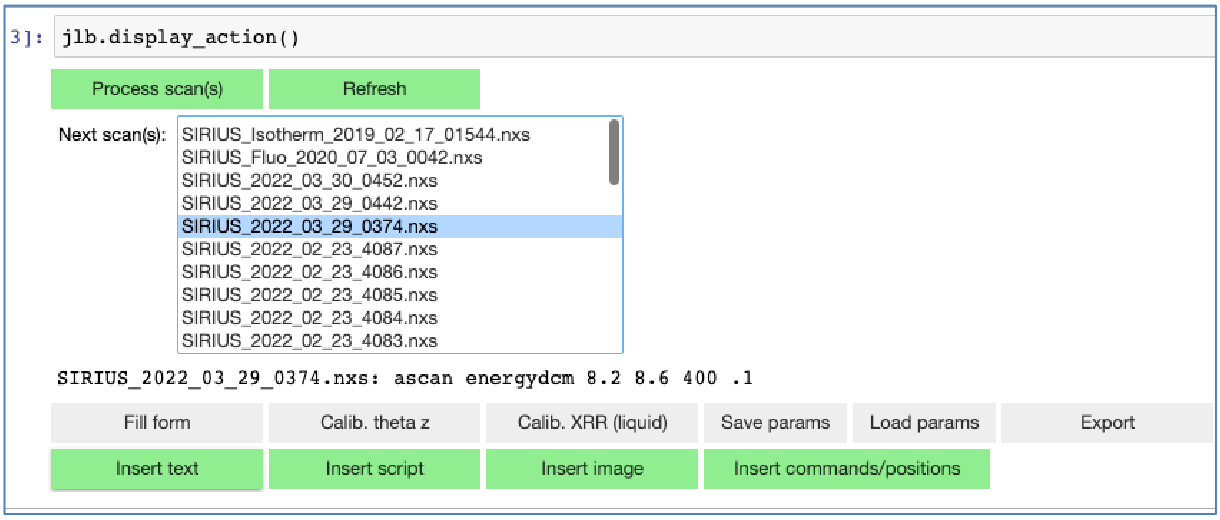

Scan selection window. When the scan is selected, click on Treat scan(s).

3.10. Save/Load params

Save/load the state of the notebook.

When a notebook is too large and you want to create a new one to continue, you can transfer the state of the variable *expt* by saving it in the current notebook and loading it into the new one, via the creation of a json file.

4. Panel "Process "

This panel allows you to choose which type of data reduction you want to apply on the selected scan.

You can have a look on the 1D data contained in the scan by selecting sensors for x and y.

NB: If you want to have this plot in the final PDF, you need to click on *Add plot to report.*

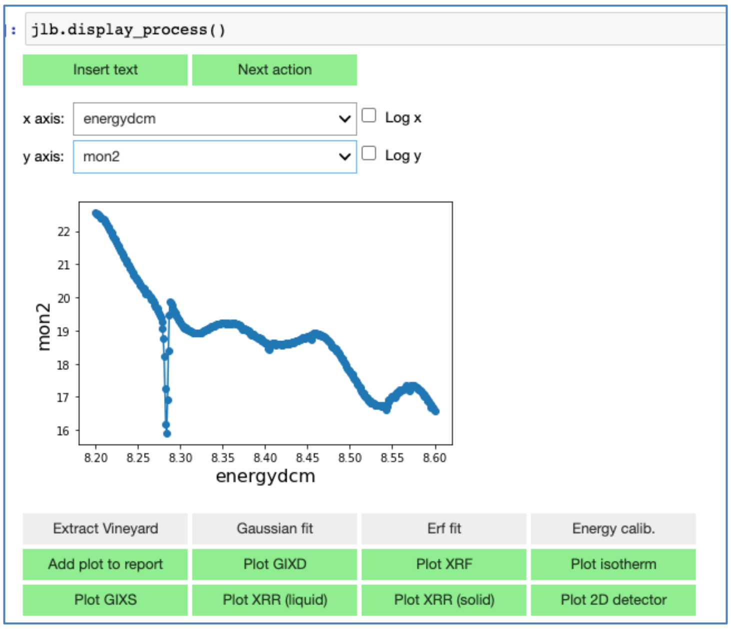

Example of a panel "Process". The plot will appear in the PDF only if you click on Add plot to report.

4.1. Next action

Bring back the panel "Action".

Click on Next action if there is something wrong or if you do not want to extract the current scan.

Careful, the subtitle with the scan number and its command will stay and needs to be manually erased.

4.2. Extract Vineyard

Extract the position of the Vineyard from a GIXD scan.

The value of the Vineyard is automatically stored in the variable `jlb.expt.channel0` and will appear as the default value in the GIXD panel.

Usually needs to be done once per experiment only.

4.3. CALIB. ENERGY

Help the calibration in energy of the DCM after a scan in energy through a foil of a standard element for XAS.

Helper to fine tune the value of the Bragg angle of the DCM, with comparison to XAS standards.

4.4. FIT WITH GAUSSIAN/ERF

Fit the 1D plot displayed with a gaussian/erf function.

4.5. ADD PLOT TO REPORT

Add the 1D plot currently displayed in the PDF report.

4.6. PLOT GIXD

Open a panel to plot a GIXD (Grazing Incidence X-ray Diffraction) scan.

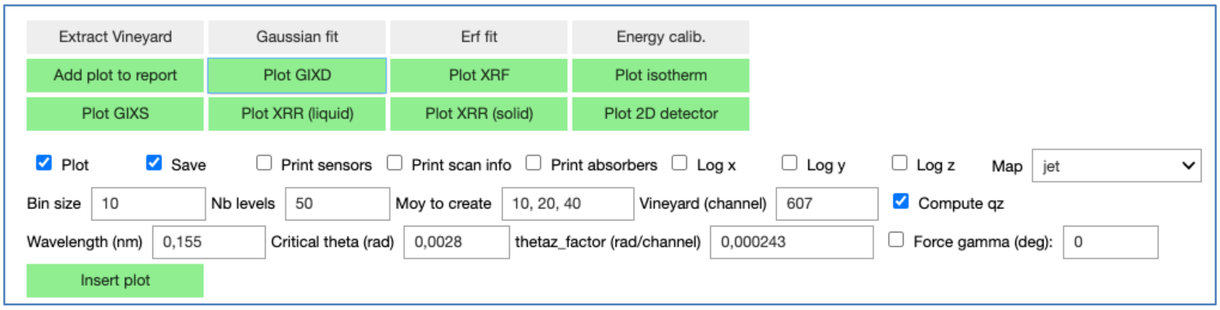

Panel Plot GIXD

4.6.1. PARAMETERS

- Print sensors: print the list of sensors in the nexus file.
- Print scan info: print several contextual info from the scan.
- Print absorbers: print the absorber used.
- log x/log y: log on the x and y axis of the integrated profile.
- log z: log on the image.
- map: colormap of the image.
- binsize: size in pixels of the vertical binning (along qz), for the image display only.
- nblevels: number of color levels for the image display.
- moy to create: binsize to be saved.
- Vineyard: channel of the Vineyard (automatically filled with *Extract Vineyard*).
- Compute qz: switch from pixels to qz in the vertical direction.
- wavelength: use 12398/E(eV)/10 to have the wavelength in nm.
- thetac: critical angle in rad.
- thetazfactor: factor for conversion from channel to radian in the vertical direction (automatically filled by *Calibration Theta z*).
- Force gamma: Tick to enforce the value of gamma **if the sensor is in the sensor list but is not correct/relevant** (for example if you are in a config for XRR); and give the corresponding value.

Works with the Pilatus detector only.

4.6.2. FORMULA

Classical approximations of small incident and exit in-plane angles are made when computing the q_{xy} -q_z map. Maps without these approximations can be obtained using another notebook available on demand.

4.6.2.1. q_{xy}

q_{xy} is extracted from the scan, and is computed with the formula:

$$q_{xy} = 4\pi/\lambda \sin(\delta/2)$$

where δ is the in-plane angle of the detector (classical 2θ in diffraction).
Note that an approximation of small incident and exit in-plane angles has been made (negligible q_x).

4.6.2.2. q_z

q_z is computed using:

$$q_z = 2\pi/\lambda \sin(\theta_z)$$

with $\theta_z = \theta_c + \gamma + (channel0 - channel) * \theta_{z,factor}$,where γ is the out-of-plane angle of the detector.
Note that an approximation of small incident angle has been made (negligible θ_i). Note also that within this approximation $q_z = 0$ is defined at the horizon of the sample and not on the direct beam, contrary to classical GISAXS or GIWAXS experiments.

4.6.3. DATA OUTPUT

Files are generated in the working directory.

- XXX_1D.mat: each line corresponds to a position of delta. It is the matrix corresponding to the image displayed.
- XXX_1D.dat: each line corresponds to a position of delta. It contains the value of each sensor, and integration along qz:
  - QzIntegrated: over the whole detector,
  - QzIntegratedTop: over its top half,
  - QzIntegratedBottom: over its bottom half,
  - QzIntegratedBottomQuarter: over its bottom quarter.

Binned data:

- XXX_1D.matNN: binning of the matrix, with NN the number of points per bin.
- XXX_1D_qz.datNN: to convert bin number to qz in XXX_1D.matNN.
- XXX_1D.moyNN: a more convenient way to represent the binned matrices with a 3 columns (qxy, qz, intensity) display.

4.7. PLOT XRF

Open a panel to plot an XRF (X-Ray Fluorescence) scan.

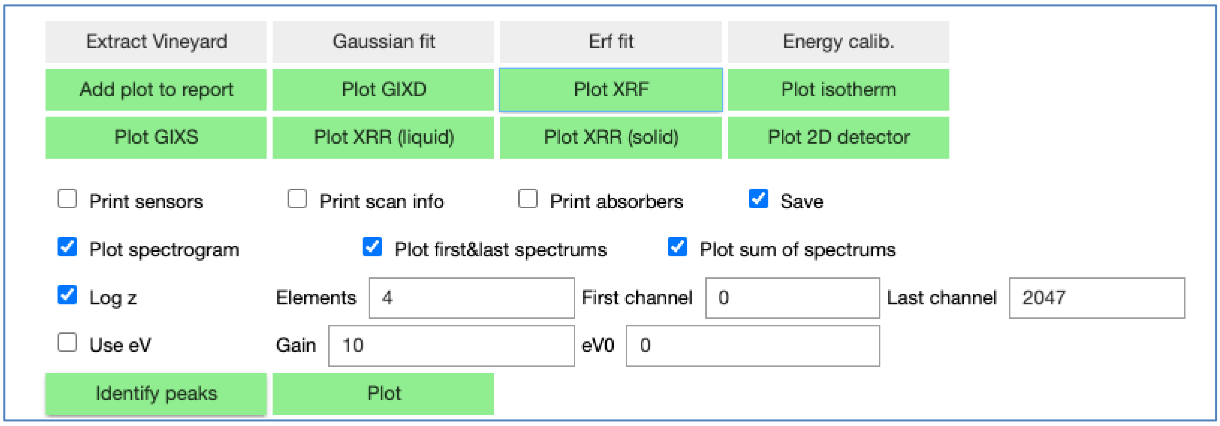

Panel Plot XRF

4.7.1. PARAMETERS

- Print sensors: print the list of sensors in the nexus file.
- Print scan info: print several contextual info from the scan.
- Print absorbers: print the absorber used.
- Plot spectrogram: plot a spectrogram (spectrums as a function of time).
- Plot first&last spectrums: plot the first and last spectrum of the scan.
- Plot sum of spectrums: plot the sum of the spectrums (i.e. a time integration).
- logz: log on the image and on the plots.
- Elements: the index of the element (i.e. the part of the detector) to be used. For the single-element detector use '4' (by convention). For the four-elements detector use '0,1,2,3' or any combination of these numbers.
- First/Last channel: define the range of channels to be used in the plots.
- Use eV: use eV instead of channels with the formula $Gain * channel + eV0$.

4.7.2. IDENTIFY PEAKS

Click on *Identify peaks* to add positions of peaks on the plots. This will open a spreadsheet with 3 columns:

- Name: the name of the peak.
- Position: the position of the peak (in eVs or channels depending on your choice in the corresponding tick box).
- Use?(y/n): allows you to keep a peak in the list without using it (useful if you alternate between different samples).

Once you are done, click on *Validate peaks.* Your current selection of peaks will be displayed. You can continue to modify the list and click on *Validate peaks* anytime you want to check it on the plot.

Click then on *Plot* to add the series of plots to the report.

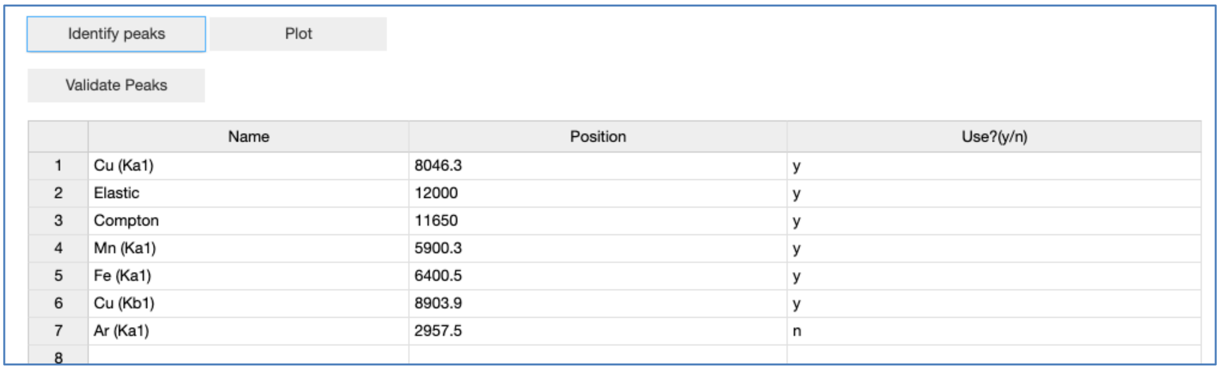

	Name	Position	Use?(y/n)
1	Cu (Ka1)	8046.3	y
2	Elastic	12000	y
3	Compton	11650	y
4	Mn (Ka1)	5900.3	y
5	Fe (Ka1)	6400.5	y
6	Cu (Kb1)	8903.9	y
7	Ar (Ka1)	2957.5	n
8			

Panel Identify peaks

NB: You have to click on *Validate peaks* to have the peaks on the final plots.

The list of peaks is saved for the next scans as well when you click on *Plot* (i.e. you will not have to enter the list of peaks for every scan, just play with the y/n option).

4.7.3. DATA OUTPUT

Files are generated in the working directory.

- XXX_fluospectrumNN.mat: raw data file converted to a matrix, with NN the index of the detector element. Each line is one data point, each column a channel. For example, if you ran a "*tscan 300 30"* the matrix will contain 301 lines (each being an integration over 30 s), and 2048 columns (corresponding to the 2048 channels of the detector).
- XXX.dat: each line corresponds to a data point and each column the value of a sensor (see the header of the file).

4.8. PLOT ISOTHERM

Open a panel to plot an isotherm.

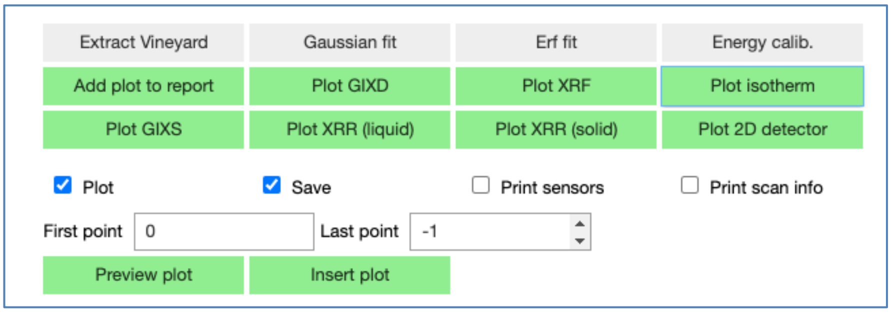

Panel Plot Isotherm

4.8.1. PARAMETERS

- Print sensors: print the list of sensors in the nexus file.
- Print scan info: print several contextual info from the scan.
- First/last point: starting/last point for the plot. Last point = -1 means that it will take the data up to the final point.

4.8.2. DATA OUTPUT

Files are generated in the working directory.

- XXX.dat: each line corresponds to a data point and each column the value of a sensor (see the header of the file).

4.9. Plot 2D detector

Open a panel to plot the time-integrated images from the Pilatus or the UFXC.

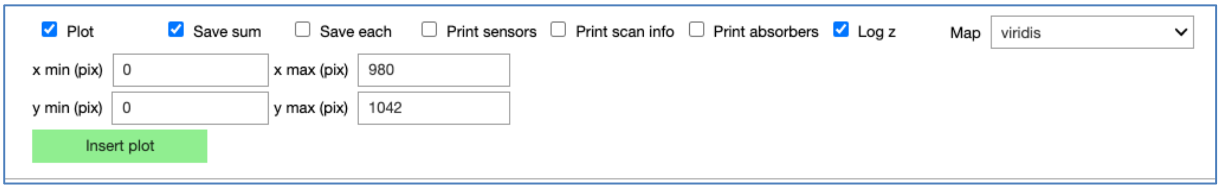

Panel Plot 2D detector

4.9.1. Parameters

- Save sum: save the sum of all images in the scan as a tiff file.
- Save each: save all individual images as tiff files.
- Print sensors: print the list of sensors in the nexus file.
- Print scan info: print several contextual info from the scan.
- Print absorbers: print the absorber used.
- logz: log on the image and on the plots.
- map: choice of the colormap.
- x min/max: x-range in pixels for the plot of the integration along the vertical axis (bottom left plot).
- y min/max: y-range in pixels for the plot of the integration along the horizontal axis (top left profile plot).

Works with Pilatus and UFXC detectors.

4.9.2. Data output

Files are generated in the working directory.

- XXX_ DETECTOR _sum.mat: the image saved as a matrix. Each pixel with the value 0 is replaced by the value -2 (easier to filter out the dead zones of the detector in the analysis).
- XXX_DETECTOR_sum.tiff: the same as the mat file, but saved as a tiff.
- XXX_integrated_x.dat: horizontal integration of **the whole detector** as a function of y (each line is a pixel in the vertical direction).
- XXX_integrated_y.dat: vertical integration of **the whole detector** as a function of x (each line is a pixel in the horizontal direction).
- XXX.dat: each line corresponds to a data point and each column the value of a sensor (see the header of the file).

4.10. Plot GIXS

Open a panel to plot a GIXS (Grazing Incidence X-ray Scattering) scan.

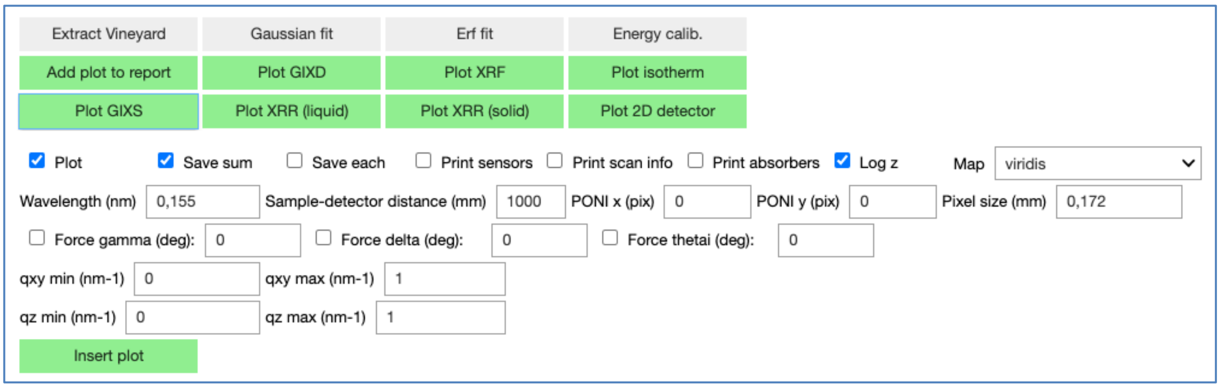

Panel Plot GIXS

4.10.1. Parameters

- Save sum: save the sum of all images in the scan as a tiff file.
- Save each: save all individual images as tiff files.
- Print sensors: print the list of sensors in the nexus file.
- Print scan info: print several contextual info from the scan.
- Print absorbers: print the absorber used.
- logz: log on the image and on the plots.
- map: choice of the colormap.
- Pixel size: pixel size in mm.
- wavelength: use 12398/E(eV)/10 to have the wavelength in nm.
- distance: distance from the detector to the center of the sample in mm.
- thetai: incident angle on the sample in rad. **thetah should be equal to 0 to avoid problems with the sign of thetai.**
- PONIx/PONIy: Coordinates of the Point Of Normal Incidence in pixels. Measured on the direct beam at delta=0 and gamma=0.
- Force gamma/delta/thetai: Tick to enforce the value of gamma or delta or thetai **if the sensors are in the sensor list but are not correct/relevant** (for example if the detector is on the delta0 platform); and give the corresponding value.
- qxy min/max: qyx-range in nm-1 for the plot of the integration along the vertical axis (bottom left plot).
- qz min/max: qz-range in nm-1 for the plot of the integration along the horizontal axis (top left profile plot).

Works with the Pilatus detector only.

4.10.2. FORMULA

4.10.2.1. QXY

qxy is computed with the formula:

$$q_{xy} = 4\pi/\lambda \sin(2\theta/2),$$

using, for each pixel of horizontal coordinate x:

$$2\theta = \tan^{-1}(pixel\_size * (x - pos\_direct\_x)/D),$$

with pos_direct_x the position of the direct beam on the rotated detector:

$$pos\_direct\_x = PONIx - D/pixel\_size * \tan(\delta),$$

where δ is the in-plane angle of the detector and D the distance detector-sample.

4.10.2.2. QZ

qz is computed with the formula:

$$q_z = 2\pi/\lambda \ (\sin(\alpha_f) + \sin(\alpha_i)),$$

using, for each pixel of vertical coordinate y:

$$\alpha_f = \tan^{-1}(pixel\_size * (pos\_direct\_y - y)/D) - \alpha_i,$$

with pos_direct_y the position of the direct beam on the rotated detector:

$$pos\_direct\_y = PONIy - distance/pixel\_size * \tan(\gamma),$$

where γ is the out-of-plane angle of the detector.

Careful, here $q_z = 0$ is placed on the direct beam ($\alpha_f = -\alpha_i$). Some users are used to another convention with $q_z = 0$ at the horizon ($\alpha_f = 0$).

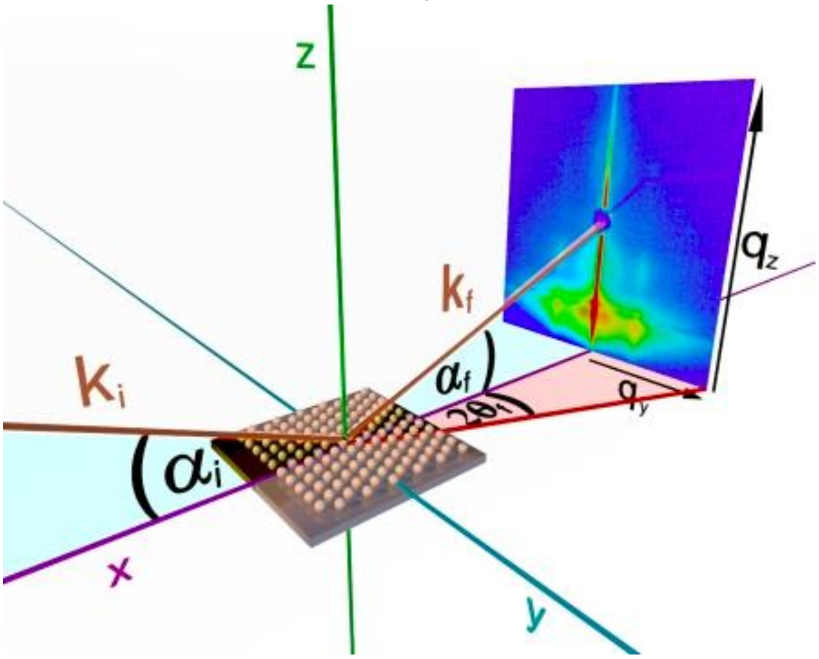

Geometry for GIXS. Image from Prof. Andreas Meyer, U.Hamburg. See http://gisaxs.com/files/Strzalka.pdf as well.

4.10.3. DATA OUTPUT

Files are generated in the working directory.

- XXX_pilatus_sum.mat: the image saved as a matrix. Each pixel with the value 0 is replaced by the value -2 (easier to filter out the dead zones of the detector in the analysis).
- XXX_pilatus_sum.tiff: the same as the mat file, but saved as a tiff.
- XXX_integrated_qxy.dat: horizontal integration of **the whole detector** as a function of qz (in nm-1).
- XXX_integrated_qz.dat: vertical integration of **the whole detector** as a function of qxy (in nm-1).
- XXX.dat: each line corresponds to a data point and each column the value of a sensor (see the header of the file).

4.11. PLOT XRR (LIQUID)

Open a panel to plot a XRR (X-Ray Reflectivity) scan, on a liquid.

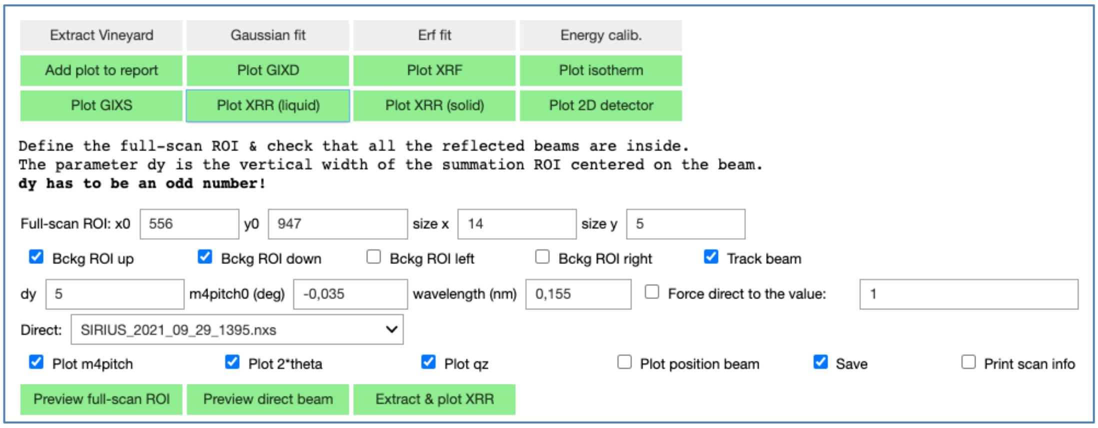

Panel Plot XRR (liquid)

4.11.1. PARAMETERS

- Plot m4pitch: plot the reflectivity as a function of m4pitch.
- Plot qz: plot the reflectivity as a function of qz.
- Plot position beam: plot the vertical position of the reflected beam on the 2D detector.
- Print scan info: print several contextual info from the scan.
- ROI x0/y0: upper left corner of the full-scan ROI containing all the reflected beams on the 2D detector.
- sizex/sizey: size of the full-scan ROI in pixels.
- Bckg ROI up/down/left/right: define the background ROIs. The average of the given ROIs is used for background subtraction.
- dy: vertical size of the summation ROI in pixels. This ROI is centered on the beam and the sum of its pixels gives the intensity. **It has to be an even number, to have the ROI correctly centered on the beam.**
- Track beam: track the vertical position of the beam, useful it moves on the detector. When the beam is not tracked the summation ROI is the full-scan ROI.
- m4pitch0: value of m4pitch when aligned with the incident beam (in deg).
- wavelength: use 12398/E(eV)/10 to have the wavelength in nm.
- Force direct to value: force the intensity of the direct beam to the given value, for normalization.
- Direct: nexus file containing the direct beam, if any. If the direct does not exist, leave it with any file and click on "Force direct to the value:"
- Preview full-scan ROI: Show the given full-scan ROI on the 2D detector.
- Preview direct beam: Show the direct scan on the 2D detector, with a zoom of height dy on the beam.
- Extract & Plot XRR: extract & plot the XRR when all parameters are given.

Works with Pilatus and UFXC detectors.

4.11.2. FORMULA

4.11.2.1. QZ

qz is computed with the formula:

$$q_z = 4\pi/\lambda * \sin(\theta),$$

with θ the incident angle on the water surface:

$$\theta = 2 * |m4\_pitch - m4\_pitch0| * \pi/180.$$

4.11.3. DATA OUTPUT

Files are generated in the working directory.

- XXX_XRR.dat: the XRR with a clear header.

4.12. PLOT XRR (SOLID)

Open a panel to plot a XRR (X-Ray Reflectivity) scan, on a solid.

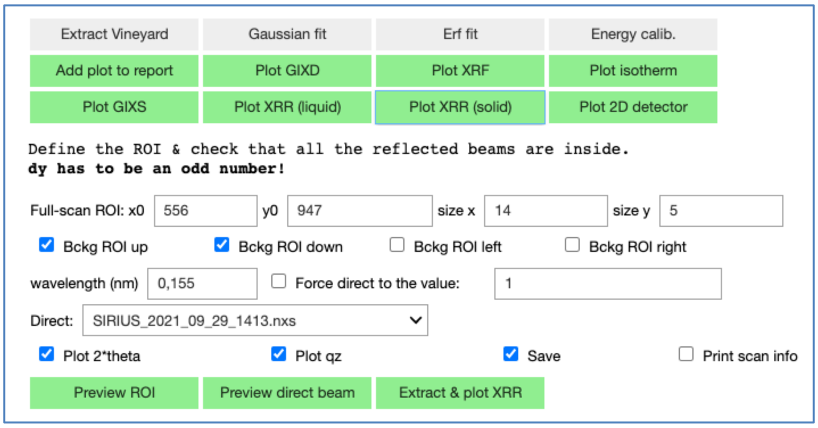

Panel Plot XRR (solid)

4.12.1. PARAMETERS

- Plot 2*theta: plot the reflectivity as a function of gamma (=2*theta).
- Plot qz: plot the reflectivity as a function of qz.
- Print scan info: print several contextual info from the scan.
- Full-scan ROI x0/y0: upper left corner of the summation ROI containing all the reflected beams on the 2D detector.
- sizex/sizey: size of the summation ROI in pixels. **sizey has to be an even number, to have the ROI correctly centered on the beam.**
- Bckg ROI up/down/left/right: define the background ROIs. The average of the given ROIs is used for background subtraction.
- wavelength: use 12398/E(eV)/10 to have the wavelength in nm.
- Force direct to value: force the intensity of the direct beam to the given value, for normalization.
- Direct: nexus file containing the direct beam, if any. If the direct does not exist, leave it with any file and click on "Force direct to the value:"
- Preview ROI: Show the given summation ROI on the 2D detector.
- Show direct beam: Show the direct scan on the 2D detector, with a zoom of height sizey on the beam.
- Extract & Plot XRR: extract & plot the XRR when all parameters are given.

Works with Pilatus and UFXC detectors.

4.12.2. FORMULA

4.12.2.1. QZ

qz is computed with the formula:

$$q_z = 4\pi/\lambda * \sin(\theta),$$

with θ the incident angle on the solid surface.

4.12.3. DATA OUTPUT

Files are generated in the working directory.

- XXX_XRR.dat: the XRR with a clear header.

5. Miscellaneous

5.1. Get the scan selection panel back

If you have deleted by mistake the scan selection panel, or if you are simply lost and do not know how to continue filling the notebook:
- Select the first code cell of the notebook (click inside),
- Execute it.
A new panel will be created at the very bottom of the notebook.

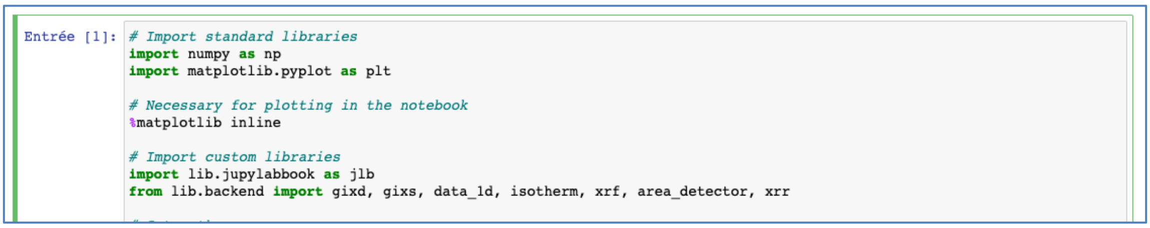

```
Entrée [1]: # Import standard libraries
import numpy as np
import matplotlib.pyplot as plt

# Necessary for plotting in the notebook
%matplotlib inline

# Import custom libraries
import lib.jupylabbook as jlb
from lib.backend import gixd, gixs, data_1d, isotherm, xrf, area_detector, xrr
```

If you are lost, select the first code cell and click on execute.

5.2. The notebook has crashed

If the notebook has crashed (weird code output when executing a cell), or if the notebook was closed by mistake, you need to restart the kernel. Click on the refresh button in the tool bar, or in the menu *Kernel* choose *Restart*. Then execute the first code cell at the top of the notebook.

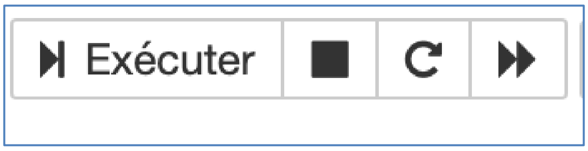

Click on the Restart Kernel button in the tool bar if the notebook has crashed (the refresh-like button)

Unfortunately all the info put into the panel boxes will be reset to default values. You will have to fill each of them back again.

5.3. Modify a text cell

To modify a text cell double-click on it. Re-execute it when done.
See the section "Insert comment" for more details.

5.4. The last scan is missing in the scan list

Wait for the scan to be finished. Click on *Refresh* in the panel *Action.*

5.5. Delete a cell

The easiest way to delete a cell is to select it and click on the scissors in the tool bar. **Be sure that you have selected the right cell!**

It happens frequently that the user deletes the wrong cell by mistake. If doing so, try to paste back the cell by clicking on Paste in the toolbar.

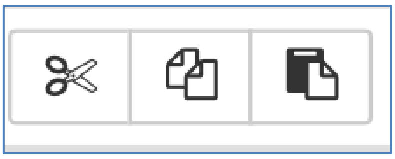

Delete a cell by selecting it and clicking on the scissors. If you want to put it back, click on Paste (extreme right button).

5.6. SAVE/COPY THE NOTEBOOK

Save regularly the notebook by clicking on *Save* in the toolbar.

It is also good to make a hard copy of the notebook every day, in case a mistake happens. To do so, copy/paste directly the .ipynb file in the file explorer.

If the notebook is bigger than ~50 Mo it is good to create a new one to continue. Use *Save/Load state* to export the state of the variable *expt* into the new notebook.

5.7. IT IS IMPOSSIBLE TO SAVE THE NOTEBOOK

If it is impossible to save the notebook (check the elapsed time since the last save in the top bar), and if there is an error in red "'_xsrf' argument missing from post":
- **do not close the current notebook!**
- start a new Jupyter session, independent of the current one.
- open a new notebook (File/new notebook), and the issue is magically gone; you can again save the notebook.

See as well: https://stackoverflow.com/questions/55014094/jupyter-notebook-not-saving-xsrf-argument-missing-from-post

5.8. EXAMPLE

An example of what the final PDF can look like is available is the folder *Example*.
