# Supplementary material for: Opportunities and new developments for the study of surfaces and interfaces in soft condensed matter at the SIRIUS beamline of Synchrotron SOLEIL
Source: J Synchrotron Radiat. 2024 Jan 1;31(Pt 1):162–76. doi: 10.1107/S1600577523008810 (PMC10833424; doi:10.1107/S1600577523008810)
Supplement: Supplementary file 1 [file s-31-00162-sup1.zip › JupyLabBook-v3.0.2/docs/XRF/PyMca_User_Manual.pdf]

Référence : EXP-SIRIUS-MANUAL-P-PyMca_User_Manual

P

PyMca: User Manual

Date de diffusion	Rédacteur	Vérificateur	Approbateur	Modifications
2022/02/11	A. Hemmerle (Beamline Scientist)			Creation. PyMca version = 5.6.7
2022/02/18	AH			Précisions ajoutées.
**Destinataires**	SIRIUS Users.			

PUBLIC

La version électronique fait foi.

Synchrotron SOLEIL – Société Civile au capital de 12.000 €
439 684 903 R.C.S. EVRY – NAF 7219Z – SIRET 439 684 903 00016
L'Orme des Merisiers – Saint-Aubin – BP 48 – 91192 GIF-S/-YVETTE Cedex
www.synchrotron-soleil.fr

TABLE OF CONTENTS

1. Introduction 3
1.1. Objective of the manual 3
1.2. Differences with JupyFluo 3
2. Open a nexus file with XRF data in PyMca 4
3. View the XRF sum spectrum 5
4. Adjust parameters in the fitting configuration 6
4.1. Open the fitting window 6
4.2. Optional: Load a config file 6
4.3. Calibration Channel-keVs 6
4.4. Configure the fit parameters 10
5. Run a fit and iteratively adjust parameters to reach a good spectral fit 13
5.1. Finding missing peaks 13
5.2. Reaching a good fit 14
5.2.1. Find the detector noise 15
5.2.2. Improving the fit quality 16
5.2.3. Save the configuration file 17
6. Batch fitting 18
7. Check the results 19
8. Another example 20
8.1. Parameters 20
8.2. Results 21
8.3. Optional: removing the scattering peaks 22

1. INTRODUCTION

1.1. OBJECTIVE OF THE MANUAL

This manual aims at explaining the different steps to fit XRF curves obtained at the beamline SIRIUS (SOLEIL synchrotron) with the software PyMca, developed at the ESRF by V. Armando Solé.

Stand-alone versions of PyMca can be downloaded here:
https://sourceforge.net/projects/pymca/
With some documentation here:
- http://www.silx.org/doc/PyMca/latest/index.html
- https://www.maxiv.lu.se/accelerators-beamlines/beamlines/nanomax/after-beamtime/pymca-xrf-imaging-guide/ (parts of this manual are based on this guide).

This manual explains all the different steps to start analyzing your XRF data from scratch. Most of the steps presented here can be skipped if you already have a configuration file from a previous experiment done in similar conditions.

This manual does not aim at getting a quantitative measurement of the concentration of each element contained in your sample. Meaning that, you will have the evolution with time of each element, but not the absolute concentration. Getting the absolute values is theoretically possible but requires a very accurate description of your setup in the configuration file, and a calibration with a reference sample in similar conditions.

The typical data analysis procedure consists of these steps:

A. Open a nexus file with XRF data in PyMca.
B. View the XRF sum spectrum.
C. Adjust parameters in the fitting configuration or load a fitting configuration template file.
D. Run a fit and iteratively adjust parameters to reach a good spectral fit.
E. Save the optimized configuration to a new configuration file.
F. Run a *Batch fitting* to analyze each individual XRF curve within the scan.
G. Check the results.

1.2. DIFFERENCES WITH JUPYFLUO

A notebook for fitting XRF curves, named JupyFluo, is also available here:
https://gitlab.synchrotron-soleil.fr/sirius-beamline/notebooks/tutorial_pymca
This notebook uses custom-defined functions for peaks and spectrums, with more flexibility than PyMca when the Rayleigh peak and its associated Compton peak are difficult to fit. JupyFluo is easily customable, but PyMca is way more complete and allows for modeling of more complex systems.
Quantitative comparisons between the two approaches have been done on regular XRF scans obtained at SIRIUS, showing almost no differences in the results.

2. Open a nexus file with XRF data in PyMca

An XRF scan will generate a nexus file containing each individual spectrum along with other data. Typical file name for XRF nexus files on SIRIUS are shaped as *SIRIUS_Fluo_YYYY_MM_DD_NUMBER.nxs*.

- Click on *Open/Data Source*.
- Go to your folder with the nxs files.
- Change *Files of type* to HDF5 Files.
- Select and open the nxs file.

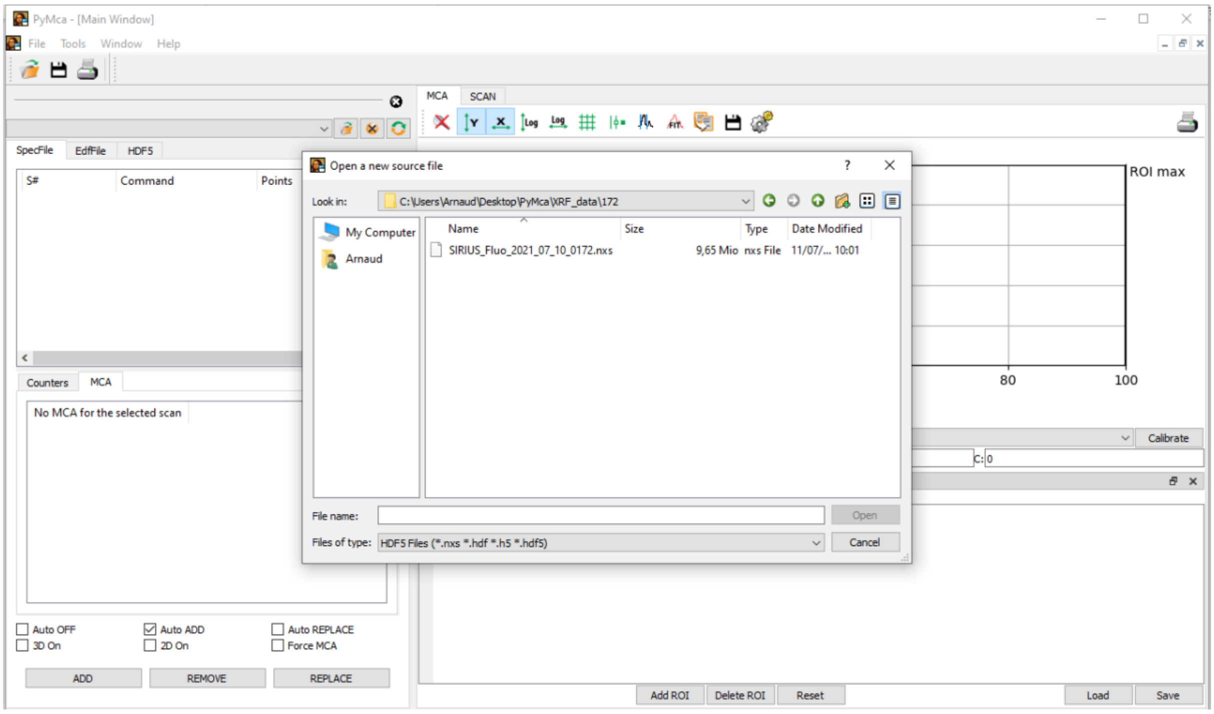

3. VIEW THE XRF SUM SPECTRUM

You have the file structure displayed in the HDF5 window.

- Click on *scan*, then on *scan_data*.
- It will display the list of sensors measured in the scan. Look for the one with the shape Nx2048, N being the number of points in the scan. In this example, you would have to select *data_12*.
- Right-click on it and click on *Show as 1D Stack*.
- A window suggests reshaping the data. Click on *Dismiss*.
- NB: If you are using the 4-elements SDD, each element will provide a *data* subentry with the shape Nx2048. Select any of the element (careful, the 4th element of the Brucker detector has a larger resolution than the 3 others).

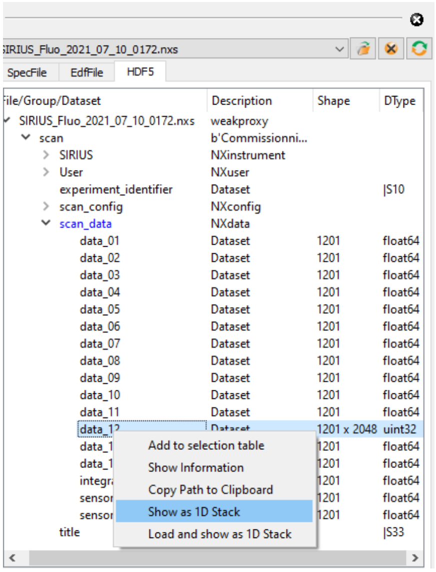

4. ADJUST PARAMETERS IN THE FITTING CONFIGURATION

4.1. OPEN THE FITTING WINDOW

- Click on the icon *Fit*.
- Choose *Advanced*.
- A window opens, saying that there are no peaks defined. Click *OK*.

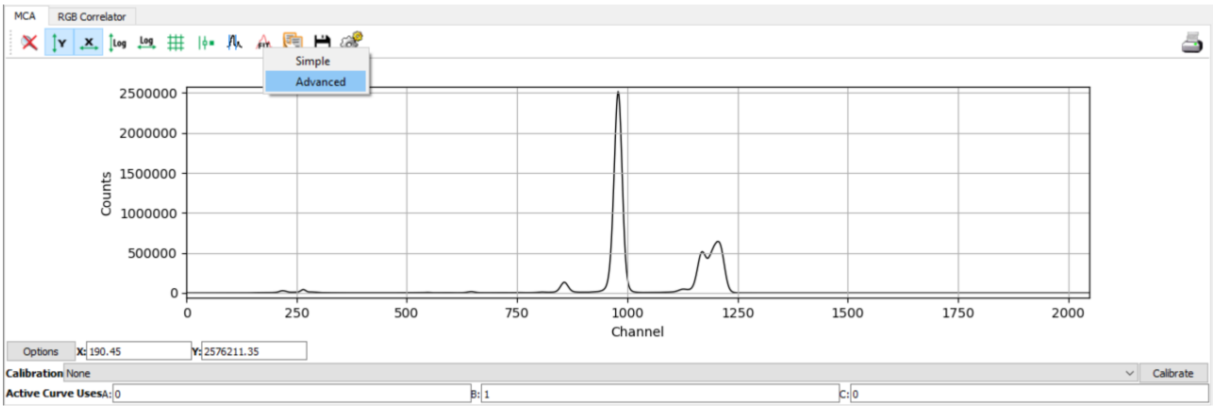

4.2. OPTIONAL: LOAD A CONFIG FILE

If you already have a config file, from the beamline staff or from a previous experiment:

- Click on *Configure.*
- In the new window, click on *Load*.
- Select your config file.

4.3. CALIBRATION CHANNEL-KEVS

If you already have a configuration file, you can skip this part.

The aim is to find the relation between the channels and the energy (in keVs). PyMca can offer a 2nd order calibration with the relation *keVs = A+B*channel+C*channel^2.* However, you should aim for a linear calibration (C=0). **On SIRIUS the calibration is always close to B=0.0099-0.01,** meaning that 1 channel is close to 9.9-10 eVs.

Before starting, you should know beforehand the emission energies of some of the elements present in your sample. To do so, you can use tabulated values (e.g. the xraydatalib), an external software (e.g. Hephaestus), or the tools present in PyMca.

Click on the icon *Log* to switch the y-axis to log scale.

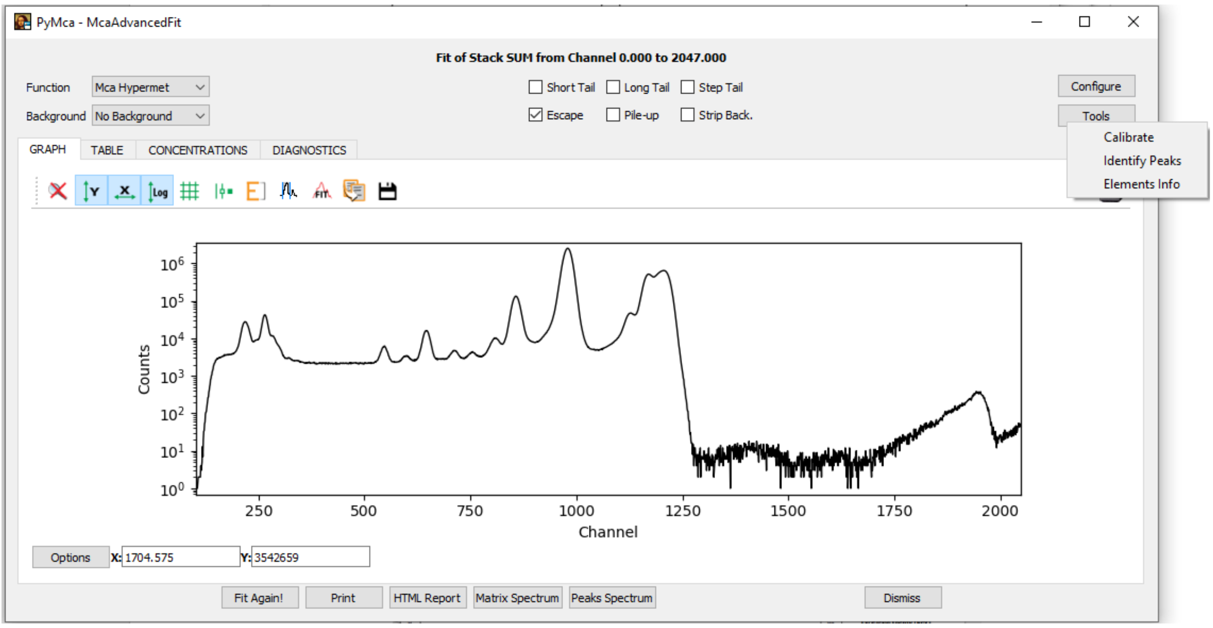

Identify Peaks:

- Click on *Tools/Identify Peaks* in the McaAdvancedFit window.
- You can type an energy and check to which element it may correspond.

Elements info:

- Click on *Tools/Elements info* in the McaAdvancedFit window.
- You can choose an element and see its emission lines.

Here, for example, we know that the sample contains Cl and Au. We should at least see the lines at 2.62 keV for Cl (channel 260, line KL), and 9.71 keV for Au (line L3M5, channel 970).

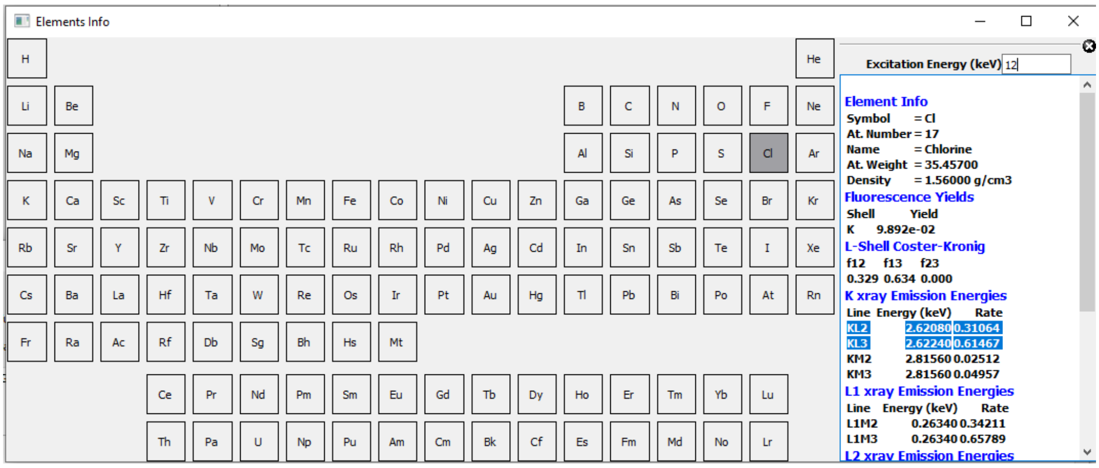

There is also a single peak around channel 545 (i.e. close to 5.4 keV). This could correspond to the KL line of Cr, and another one at channel 640 which would be the KL line of Fe.

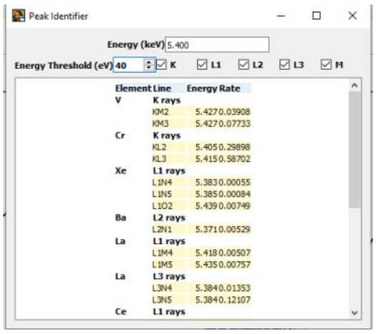

It is dangerous to use the position of the scattering peak, as it may appear shifted due of its Compton foot.

We are now ready for the calibration:

- Click on *Tools/Calibrate* to open the MCA Calibration Widget.
- Check that *Order: 1st* is selected.
- Click on the icon *Clear peak table and search peaks.*

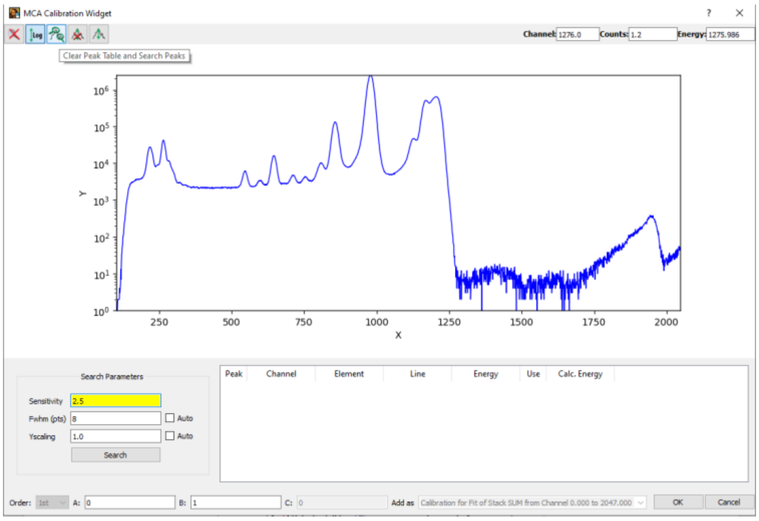

It detects the position of peaks that we will use for the calibration. Click on one the peak you have identified before. Here for example, we click on the peak at channel 265, that we identified as the KL line of Cl. Choose the right element/line and click on *OK*.

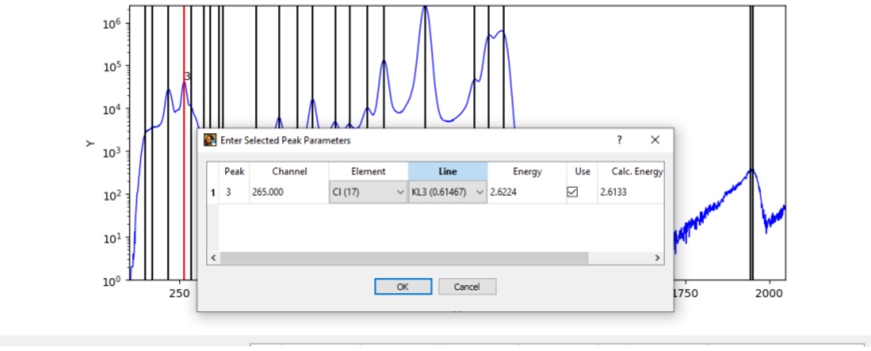

Click on the peaks you have identified and associate them with their corresponding emission line. The calibration parameters A and B are displayed at the bottom of the window.

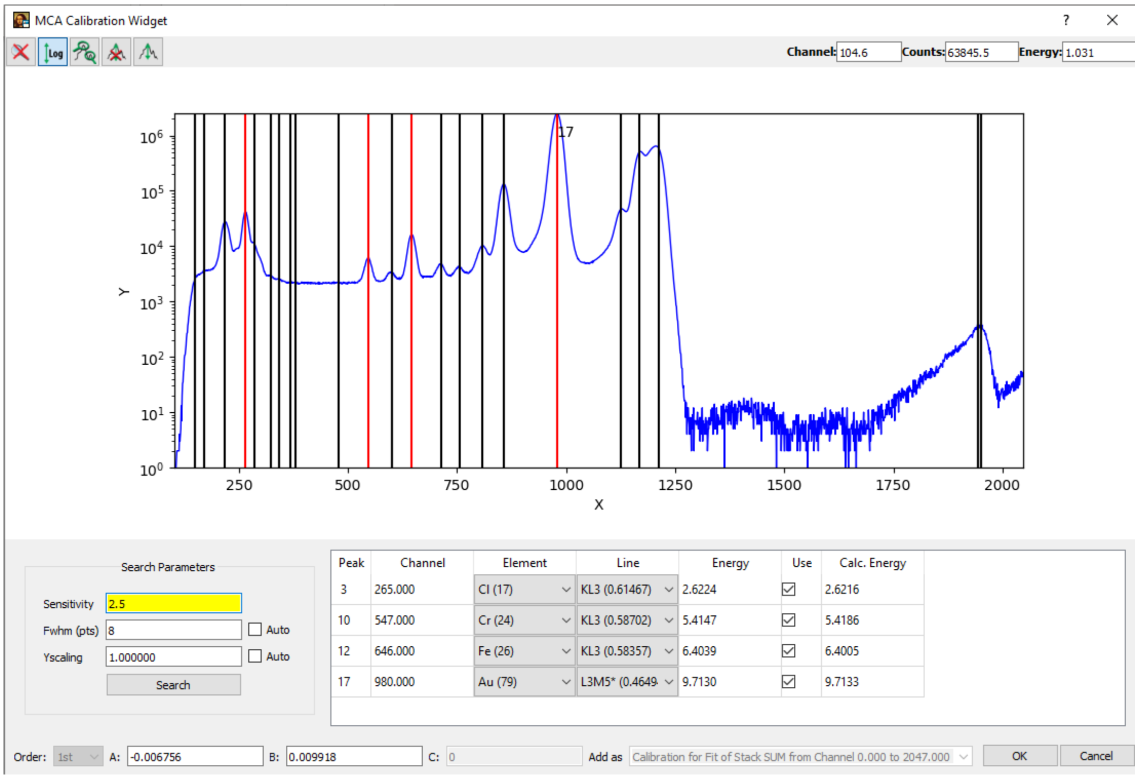

Click on *OK.* You can now switch between channels and keVs in McaAdvancedFit window by clicking on the icon *Toggle Energy Axis.*

4.4. CONFIGURE THE FIT PARAMETERS

If you already have a configuration file, you can skip this part.

Starting from now you will have to go back and forth between the windows McaAdvancedFit and MCA Fit Parameters.

Click on *Configure.* This opens the window MCA Fit Parameters.

Tab. FIT

- Set the *Continuum type* to *Constant.*
- Click on *Limit fitting Region to:* and choose a range of channels so that it starts just before the first peak of interest, and stops right after the high-energy foot of the scattering peak. Here, for example, we choose the range 180-1240.
- Check that *Escape peaks* and *Scattering peaks* are ticked on (and the other parameters ticked off).

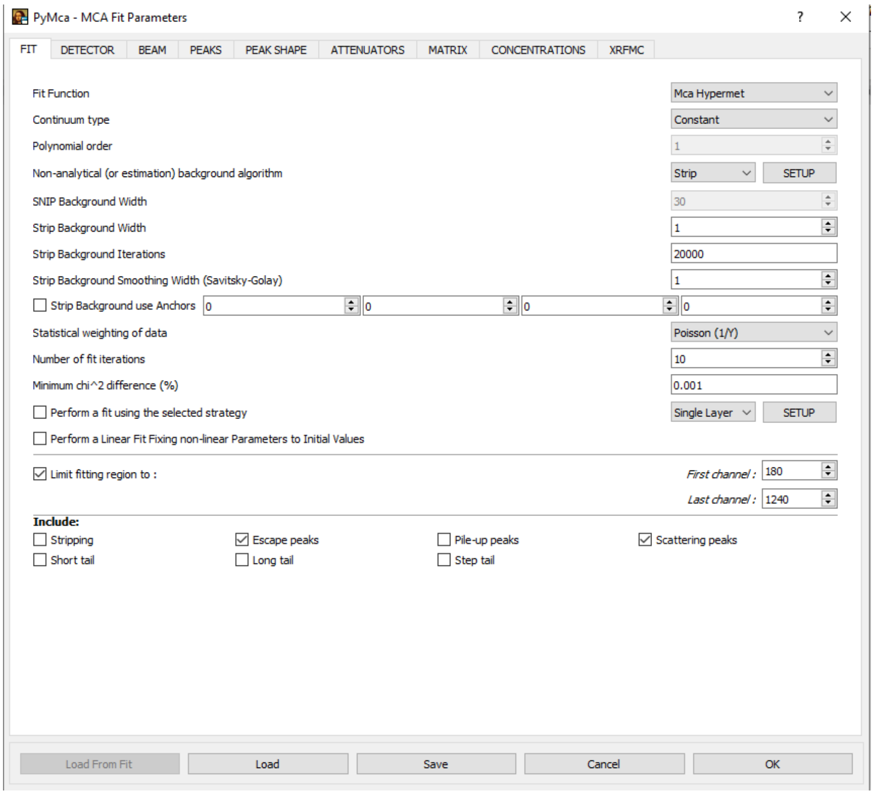

Tab. DETECTOR

Fix the values of *Spectrometer zero, Spectrometer gain, Fano factor.*

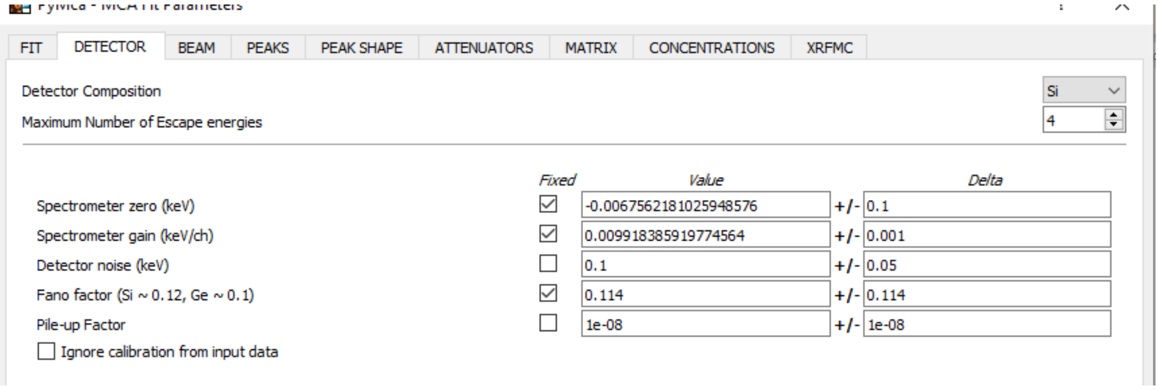

Tab. BEAM

Click on *Energy 0*, put the energy of the scattering peak (also known as the elastic peak) in keV, and a weight of 1.

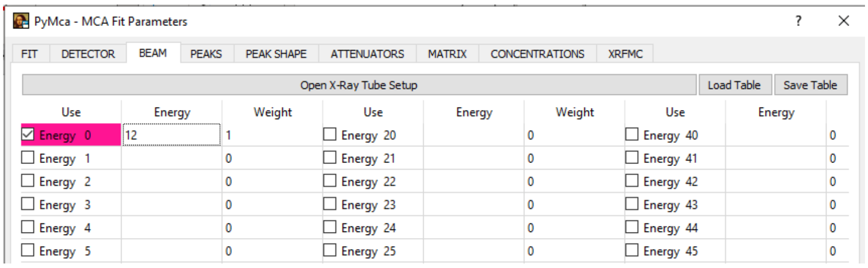

Tab. PEAKS

This is where you select the peaks corresponding to the elements contained in your sample.

- Click on the element.
- Click on the line(s). If you choose *K,* for example, all the K lines will be included in the fit, with a tabulated ratio between the lines. If you choose the lines individually by clicking on *Ka* and *Kb*, they will be fitted independently. Most of the time we select *K* and/or *L* and/or *M* but not the individual lines.
- Here we select the K lines of Cl, Cr and Fe as we already know that these elements are present; and the L and M lines of Au.

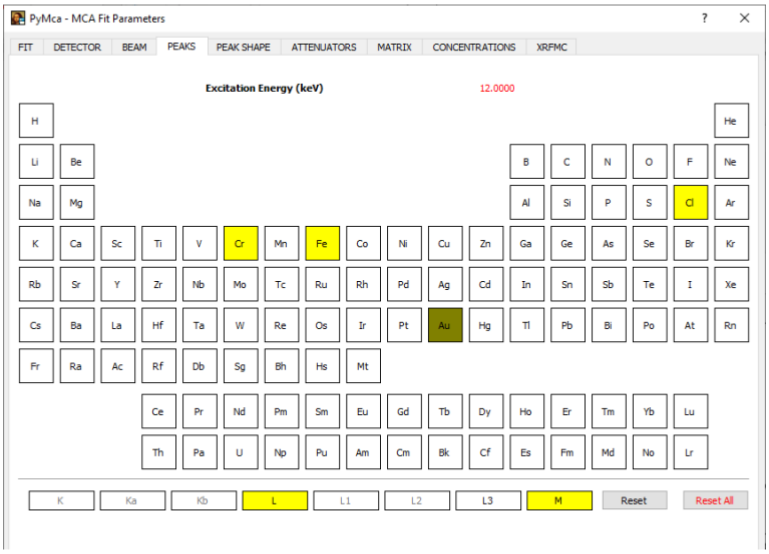

Tab. MATRIX

Set here the incident and detector angles in degrees. The scattering angle will have an influence on the position of the Compton peak, and you may adjust the value of the detector angle later to improve the fit.

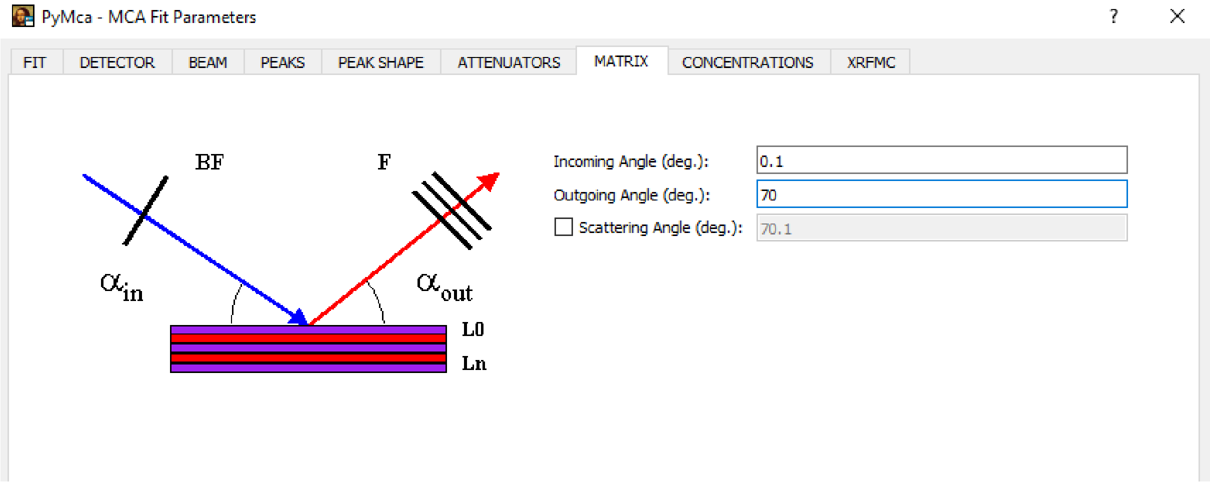

Click on *OK* to go back to the McaAdvancedFit window.

5. Run a fit and iteratively adjust parameters to reach a good spectral fit

Switch on the *Energy* axis (icon E) and click on *Fit Again!*

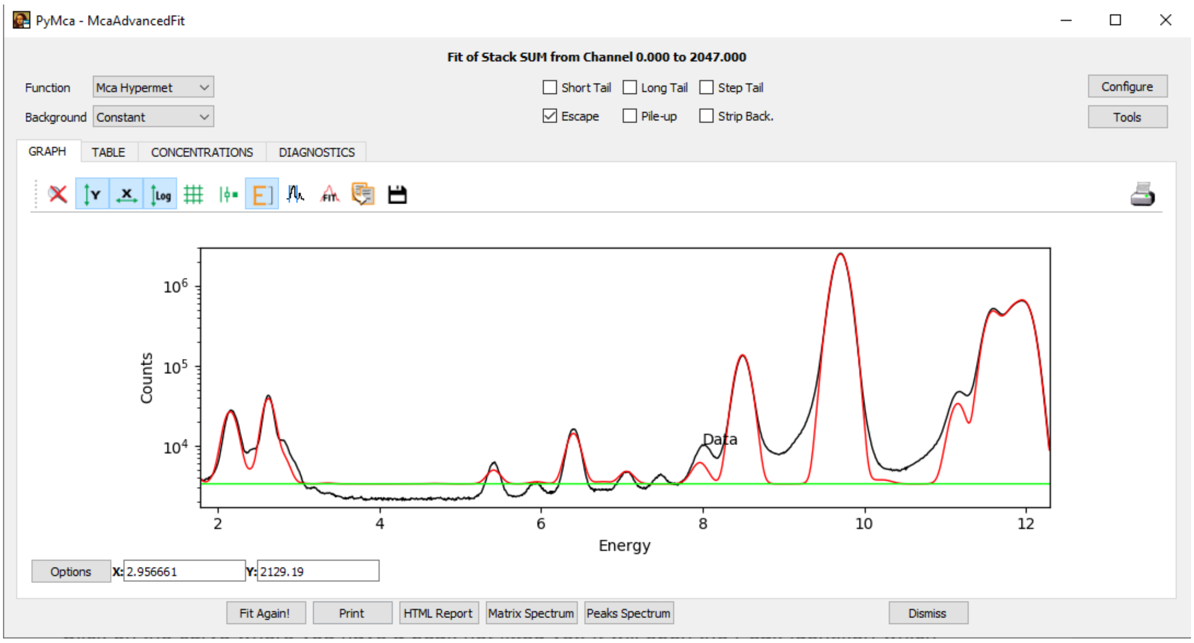

Your fit is probably not very good yet. Here we see first that some peaks are missing.

5.1. Finding missing peaks

Click on the curve where you have a peak not fitted yet. It will open the *Peak Identifier*, which will help you by giving you hints on what are the possible peaks located at this energy. Aim for the peaks with the highest rates.

Then, go back to the *Configure/Peaks* tab and add the corresponding peak.

For example here, the missing peak is most likely Cu (coming from the sample environment).

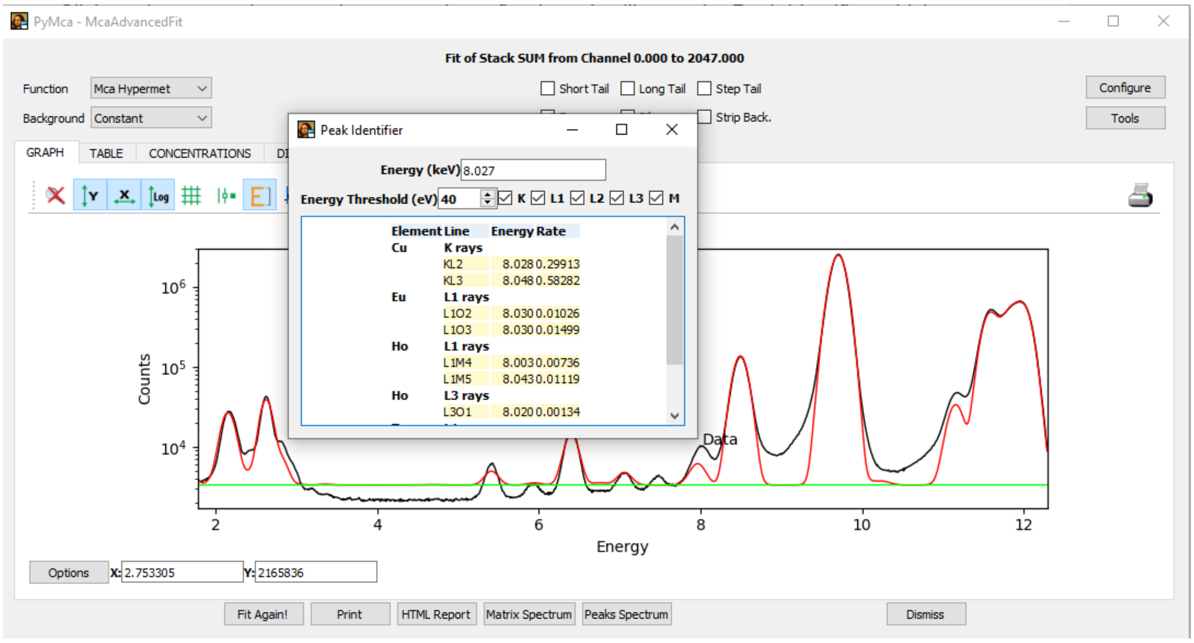

After some effort, you should be able to identify most of the peaks.

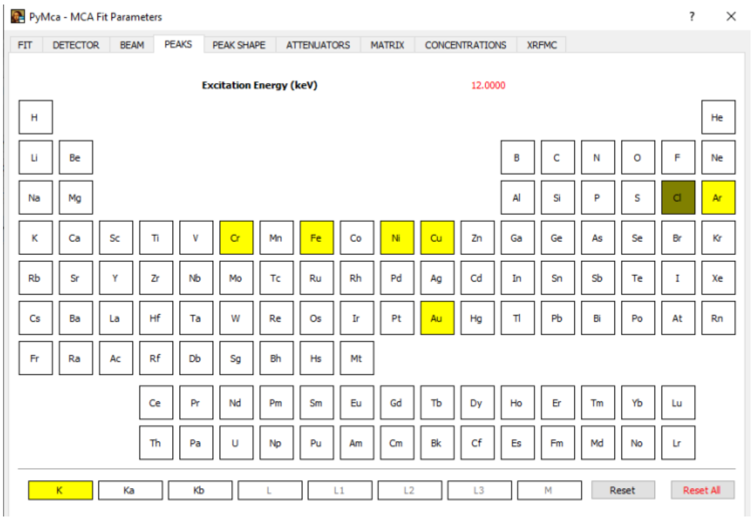

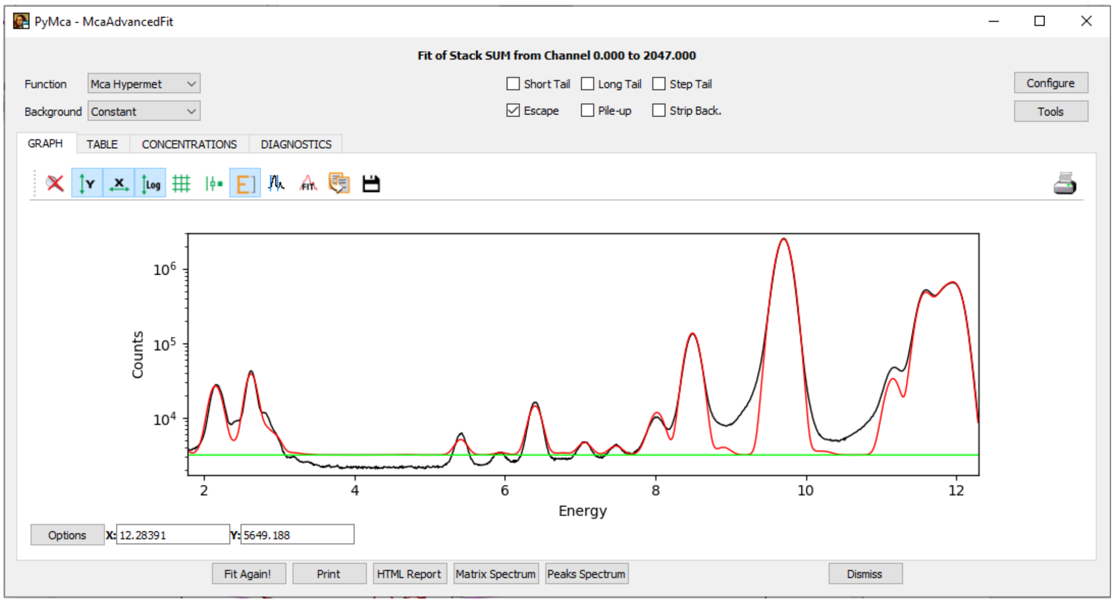

In this example, all the peaks have been identified but:

- The shape of the peaks is not adjusted yet.
- The background is not fitted well.

5.2. Reaching a good fit

If you already have a configuration file and the fit looks good for your purpose, you can skip this part.

The quality of the final fit you should reach depends on what quantities you would like to extract. Keep also in mind that:

- You are working in log scale, and deviations from the data may account for only a few percent of the total area of the peaks.
- At this step, you are mostly looking for good initial guesses for the batch fitting which will follow.
- The way to reach a good fit may depend on your sample (i.e. there is no magical recipe for every situation).

5.2.1. FIND THE DETECTOR NOISE

We will determine the detector resolution on a series of individual peaks (ideally more than one). In this example we will focus on the sub-range between channels 500 and 770.

- Click on *Configure* and go to the tab *FIT.* Set the range of channels and click on *Stripping*, *Short Tail* and *Step Tail.*

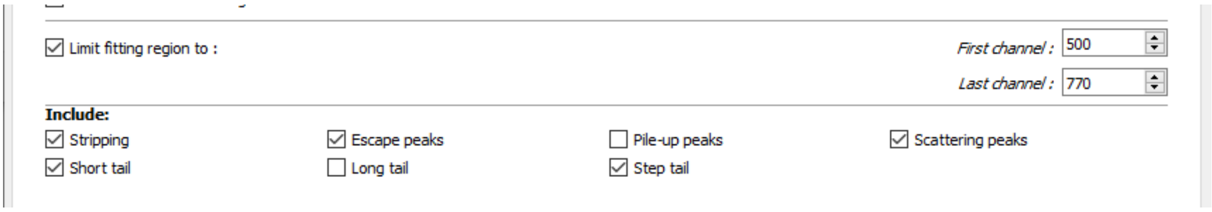

- Click on *OK* and *Fit again.* Here, even if the Cr is not fitted well, the other peaks are good for determining the resolution.

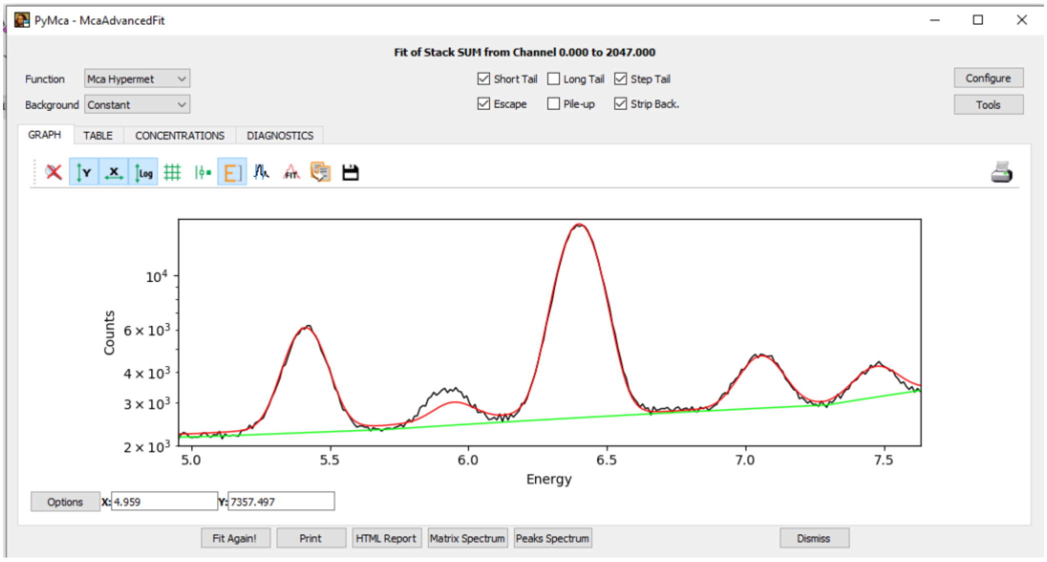

- Go to *Configure.* Click on *Load from fit.* A window appears, click on *OK.*
- In the tab *DETECTOR* you can see that the detector noise has been updated. Click on *Fixed* to fix this value.

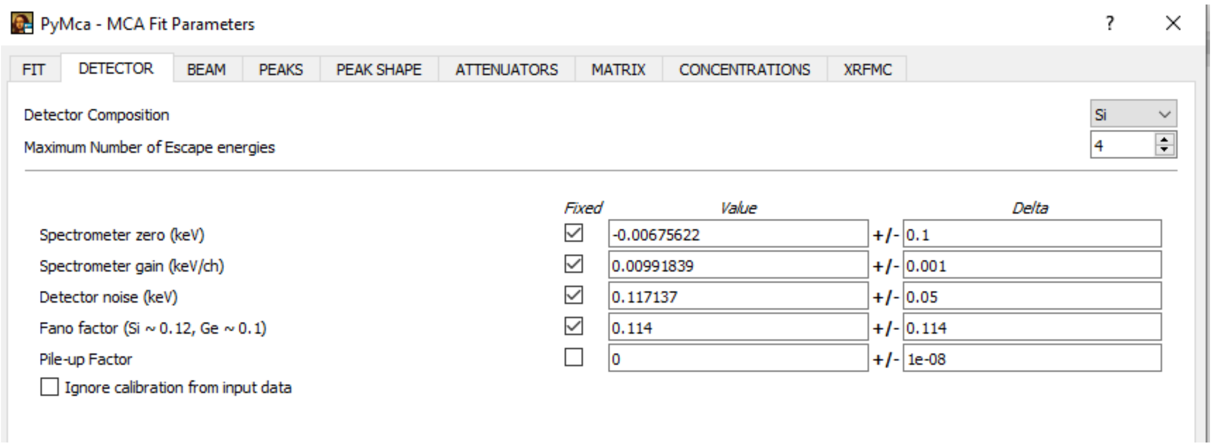

- Go to the tab *FIT.* Set back the original range of channels.

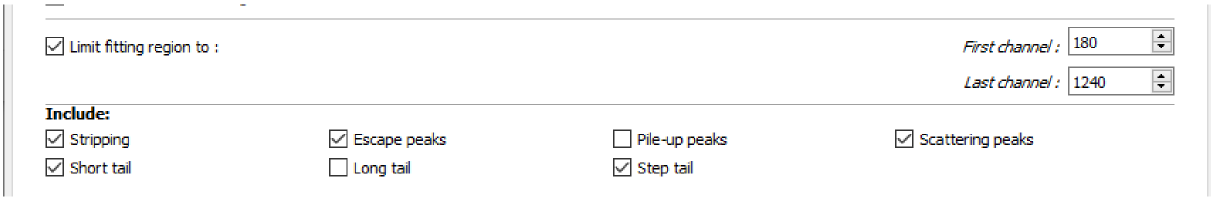

5.2.2. Improving the fit quality

- Click on *OK* and do another fit. Click on *Pile-up* and check if it improves the fit. If it does, keep it ticked. If there is an error or no change at all, set up the initial value of the pile-up factor (*Configure/DETECTOR)* to 1e-8, not fixed, and fit again.

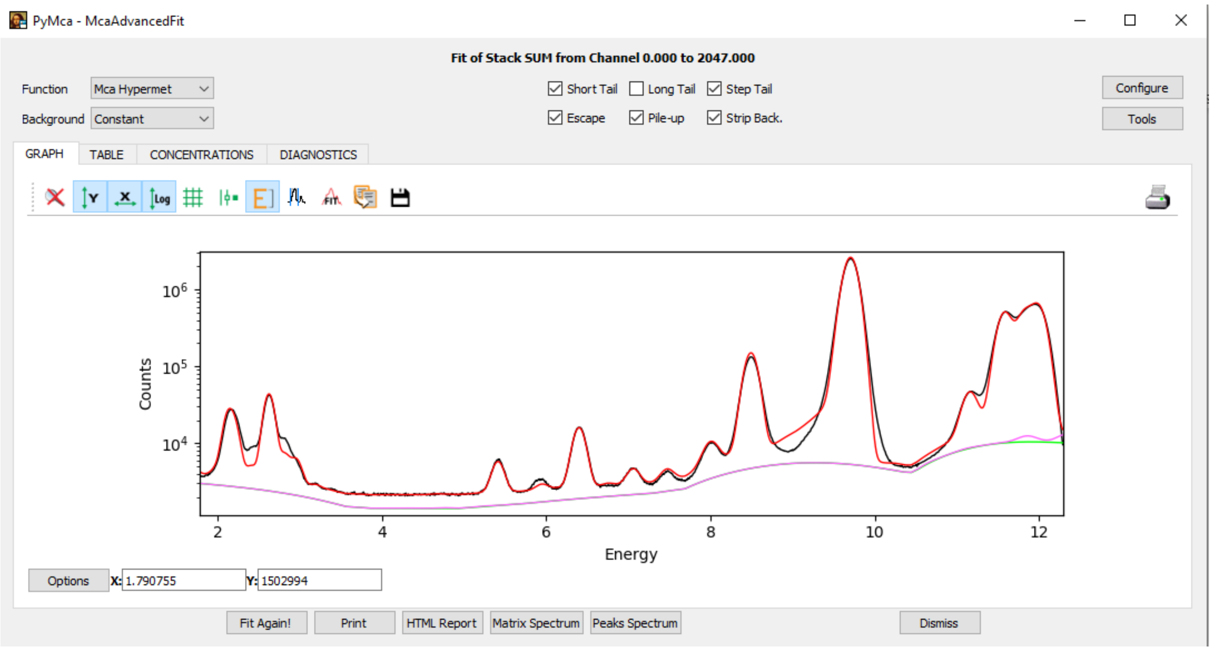

- In the present example, the fit can be improved in the region of 2-3 keV. Use with caution, but you can decorrelate the KL peaks from the Cl family and similarly add KL peaks for S.

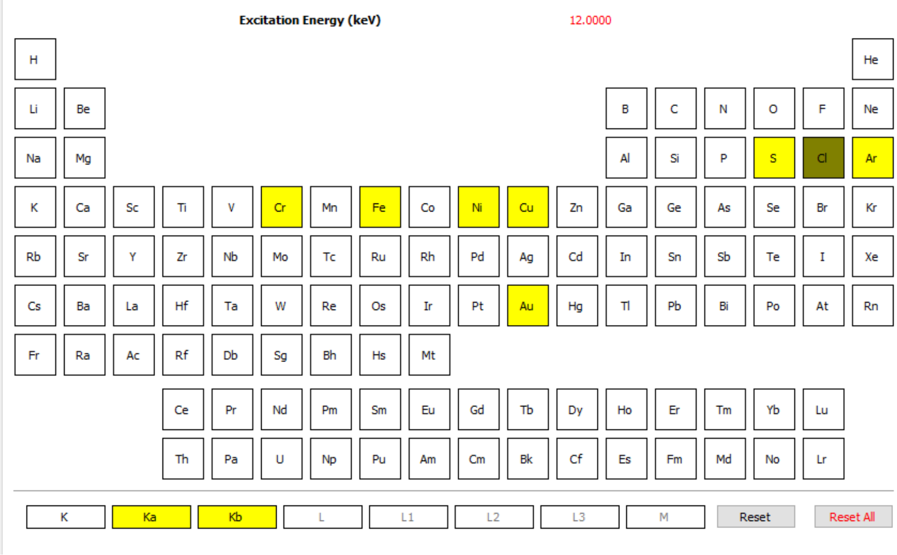

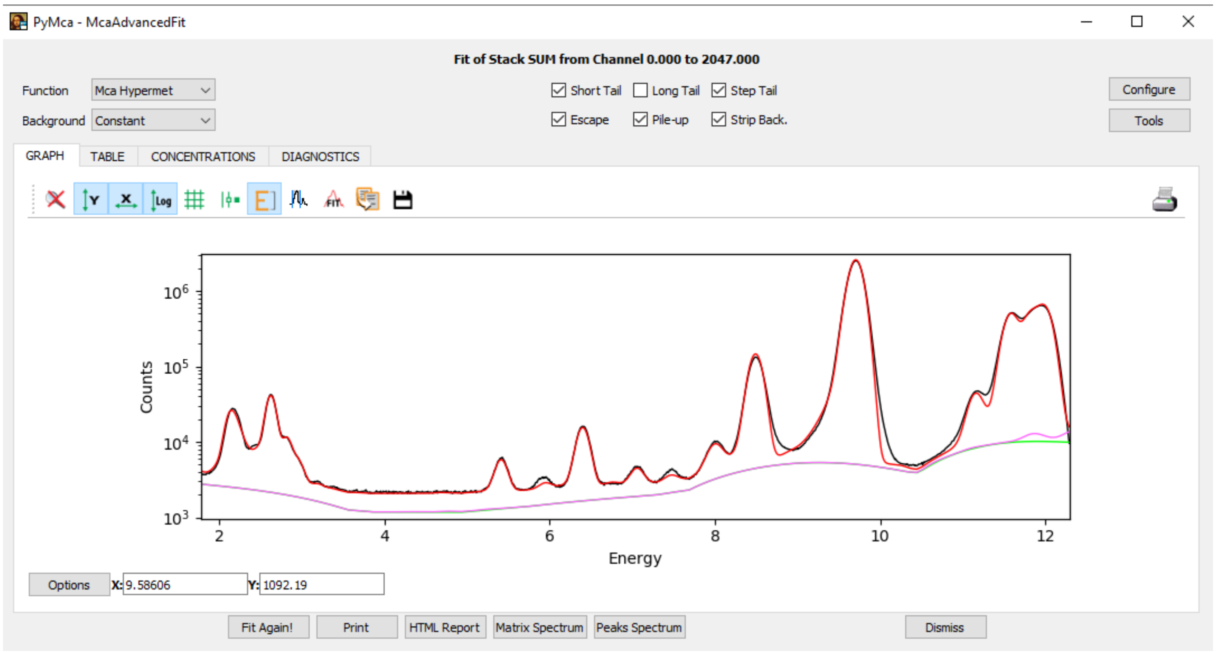

- If you do not expect large variations of the overall intensity of your scan, it is better to fix the peak parameters (the results will be less noisy). In *DETECTOR,* fix the Pile-up factor, and in *PEAK SHAPE* fix *Short Tail Area, Short Tail Slope* and *Step Height.*

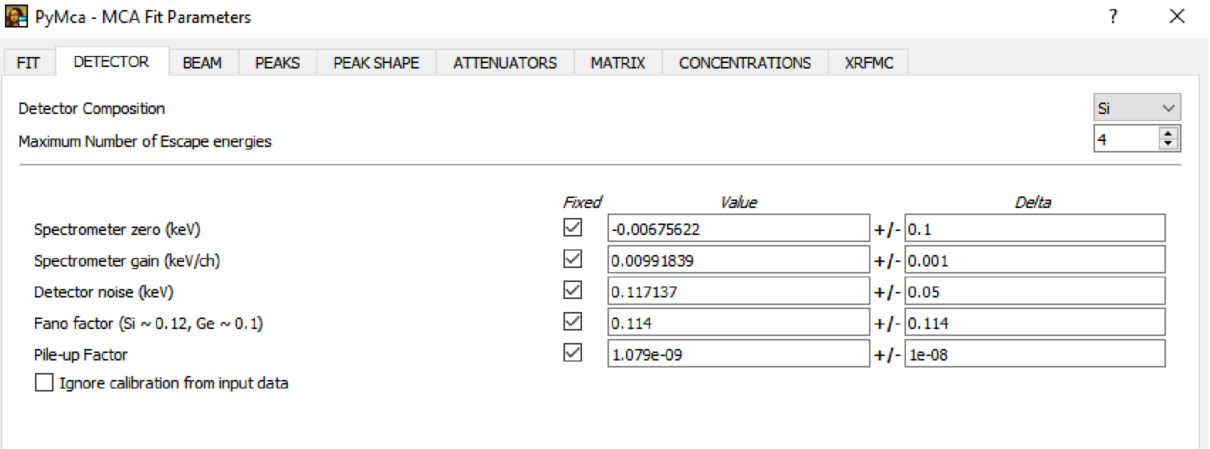

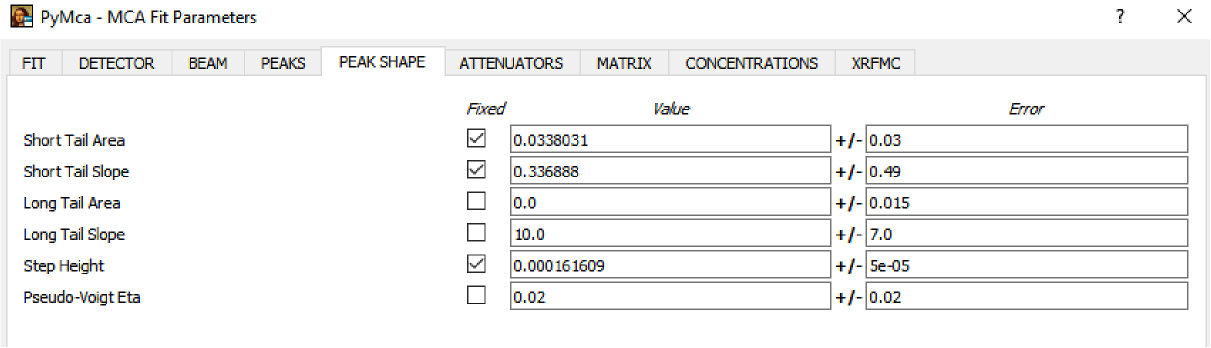

5.2.3. SAVE THE CONFIGURATION FILE

Very important, save the configuration file, which will be used for the batch fitting. Click on *Configure/Save.*

6. BATCH FITTING

- Go back to PyMca main window and select *Tools/Batch Fitting.*

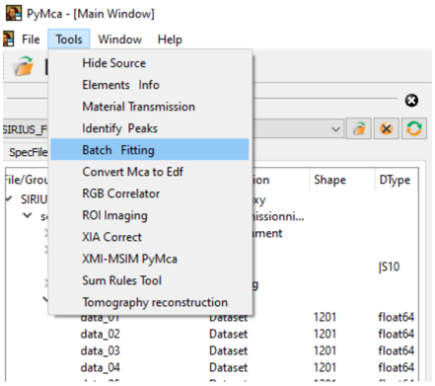

- *Browse* and select the nexus file (switch the file type to HDF5).
- Select the data set containing the XRF data (see Part 2). Tick the box *Signals*.

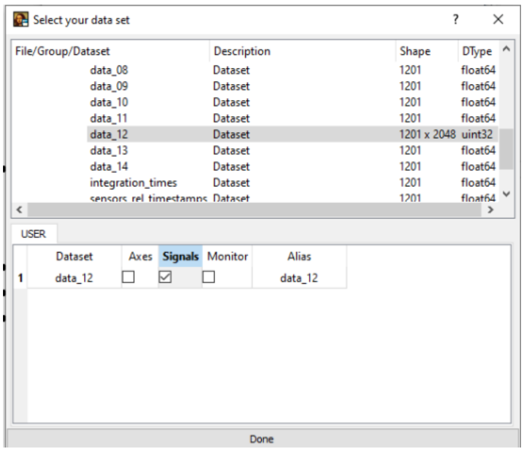

- If you are using the 4-elements detector, click on each data set you want to use and click their *Signals* boxes. The analysis will be done on the sum of each element.
- Add the configuration file, created in the previous part.
- Select the folder where the results will be stored.
- Tick *Generate Report/Overwrite/Table in Report/HDF5/CSV.* You can also use several processors. Then click on *Start.*

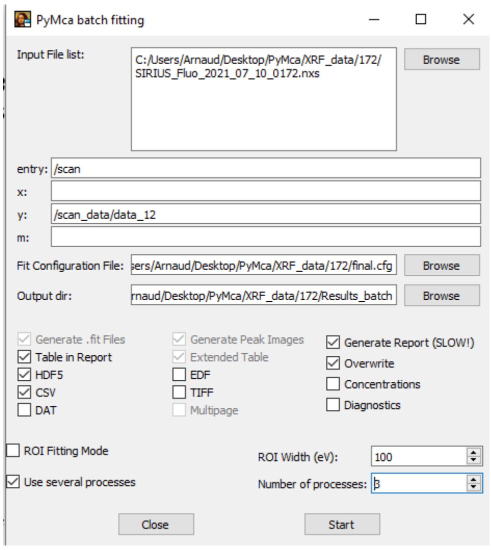

7. CHECK THE RESULTS

The results will be saved in various formats. This represents in the end a lot of files, you may want to delete or compress individual results from the folders FIT and HTML when the fit is done. If you keep the config file, you will be able to redo the exact same fit later if needed.

Folder FIT

Text files with details for each fit performed (params and curves).

Folder HTML

The file *index.html*, from which you can check each individual fit with the results displayed as a Table. Generating the HTML makes the fit VERY slow, and takes a lot of place.

Folder IMAGES

The config file used for your fit, and a csv file with all the results (area of each peak as a function of the spectrum index). You can use Excel to directly look at the csv file.

FILENAME.h5

A HDF5 file with the results, that you can open with PyMca (same way you would open a Nexus file).

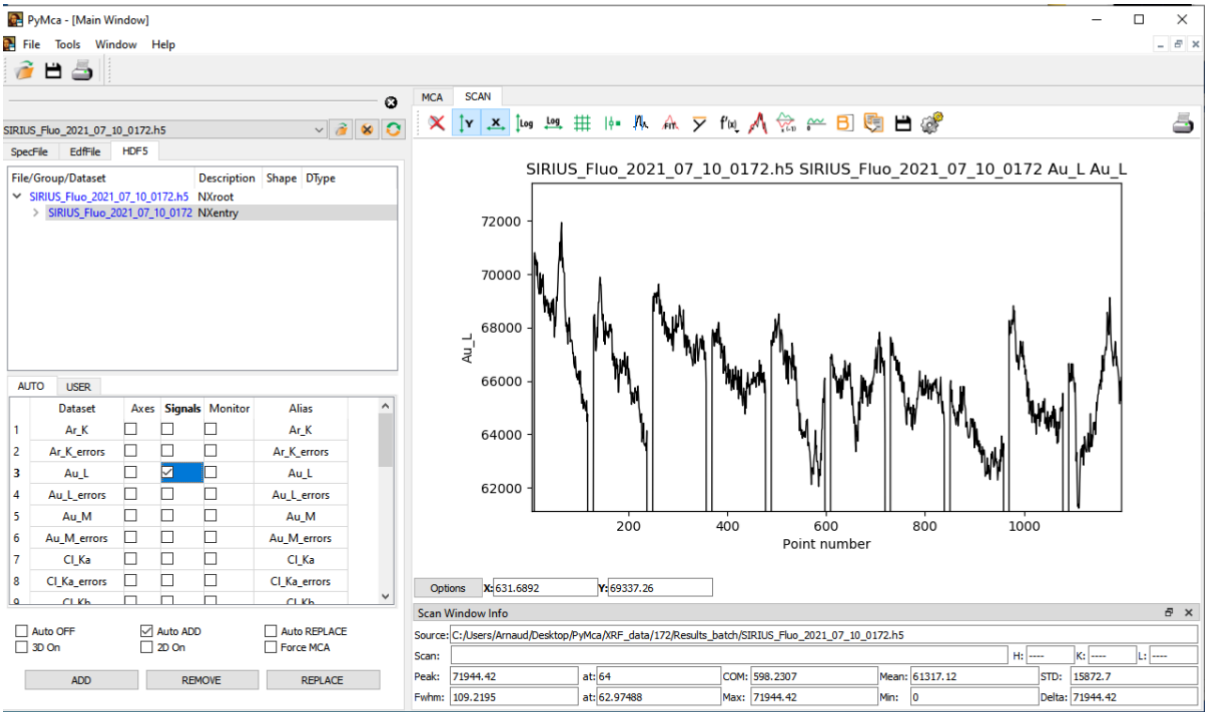

8. ANOTHER EXAMPLE

8.1. PARAMETERS

We describe another example, which requires a bit more work to reach a good fit. The main difficulty here is the large Compton foot that might be due to the geometry of the experiment, or to a fluorescence intensity too high for the acquisition card (ratio ICR/OCR > 1).

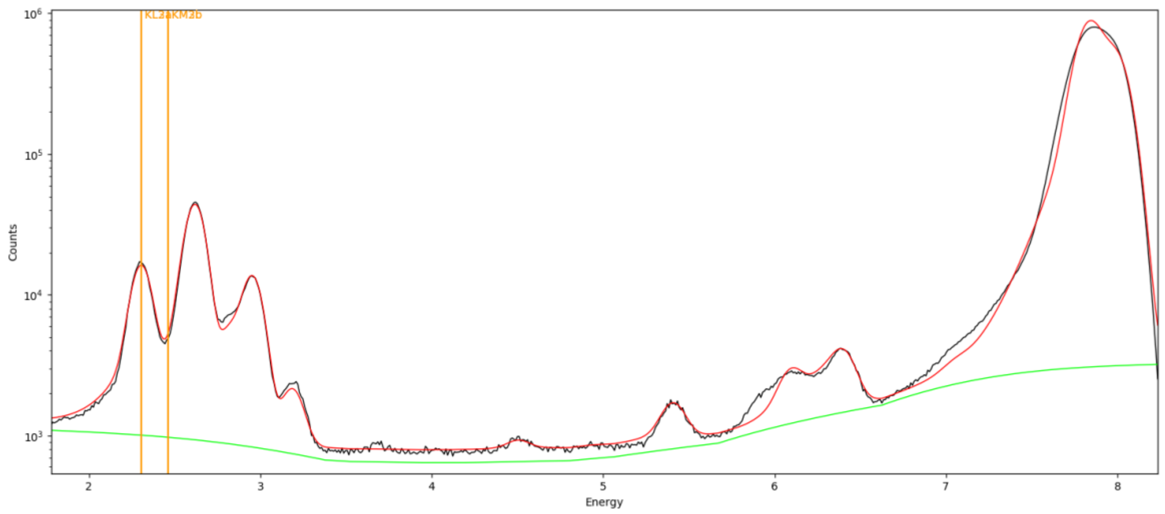

We manage to get a reasonable fit of the scattering peaks by setting the angle of the detector to 108 deg. We still see that the escape peak of the Compton foot around 6 keV is not fitted well, and no elemental peak should be extracted from this part of the curve.

Here are the parameters used. We split the two K lines of S to improve the quality of the fit, with minor impact on the final result.

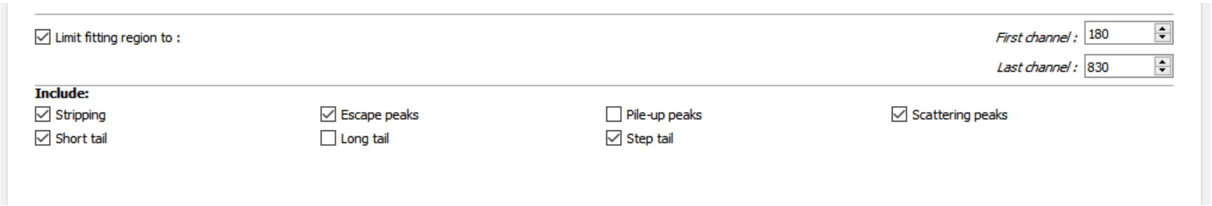

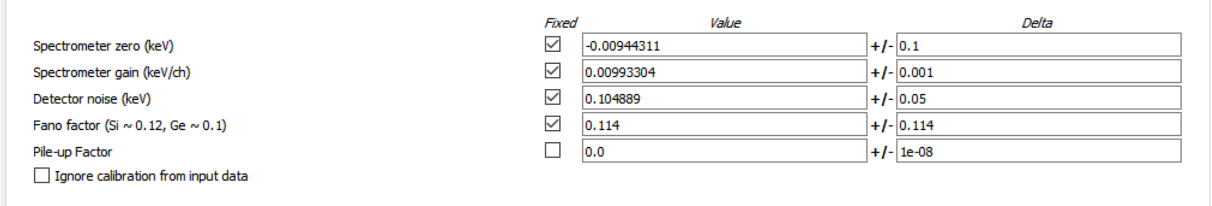

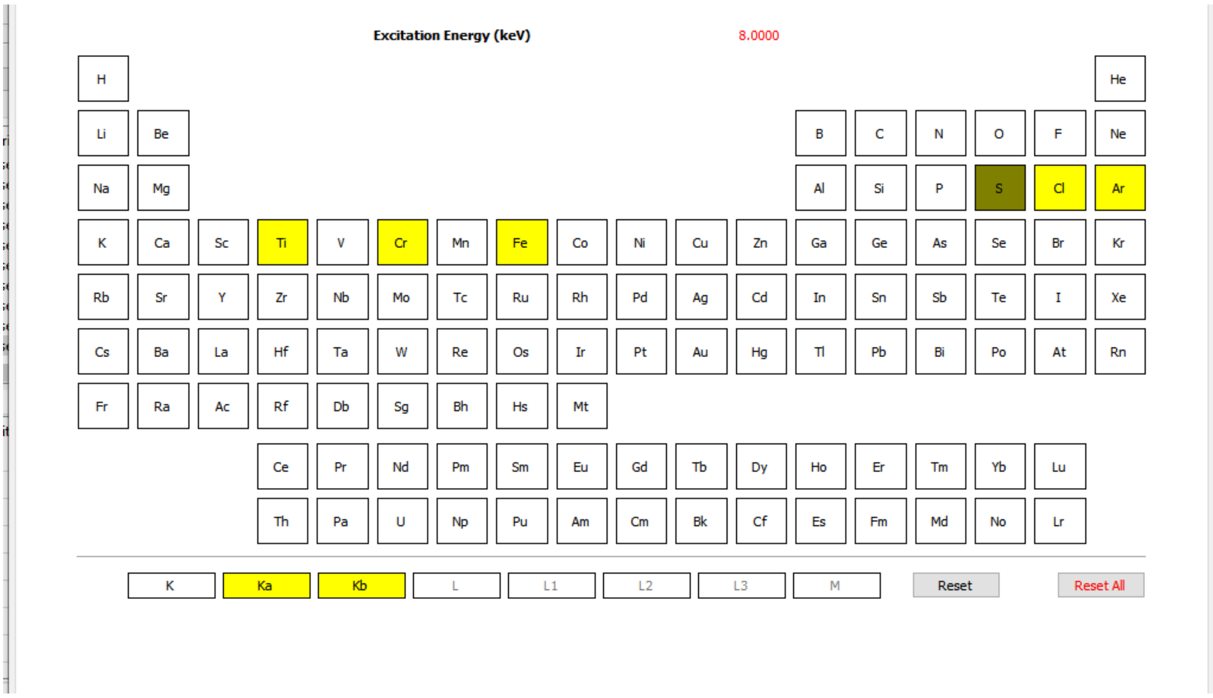

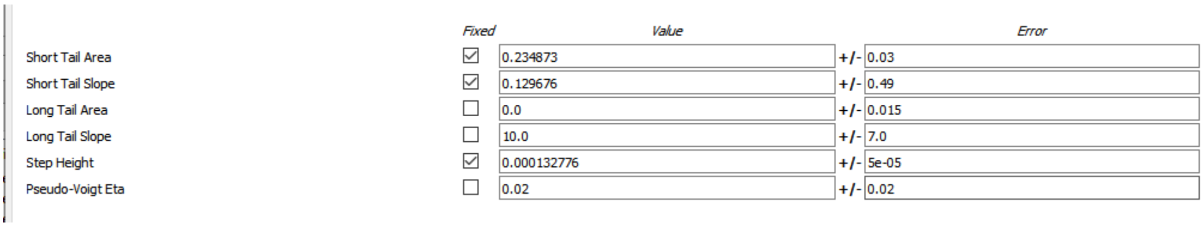

8.2. Results

Here is, for example, the result on the S Ka line obtained after batch fitting.

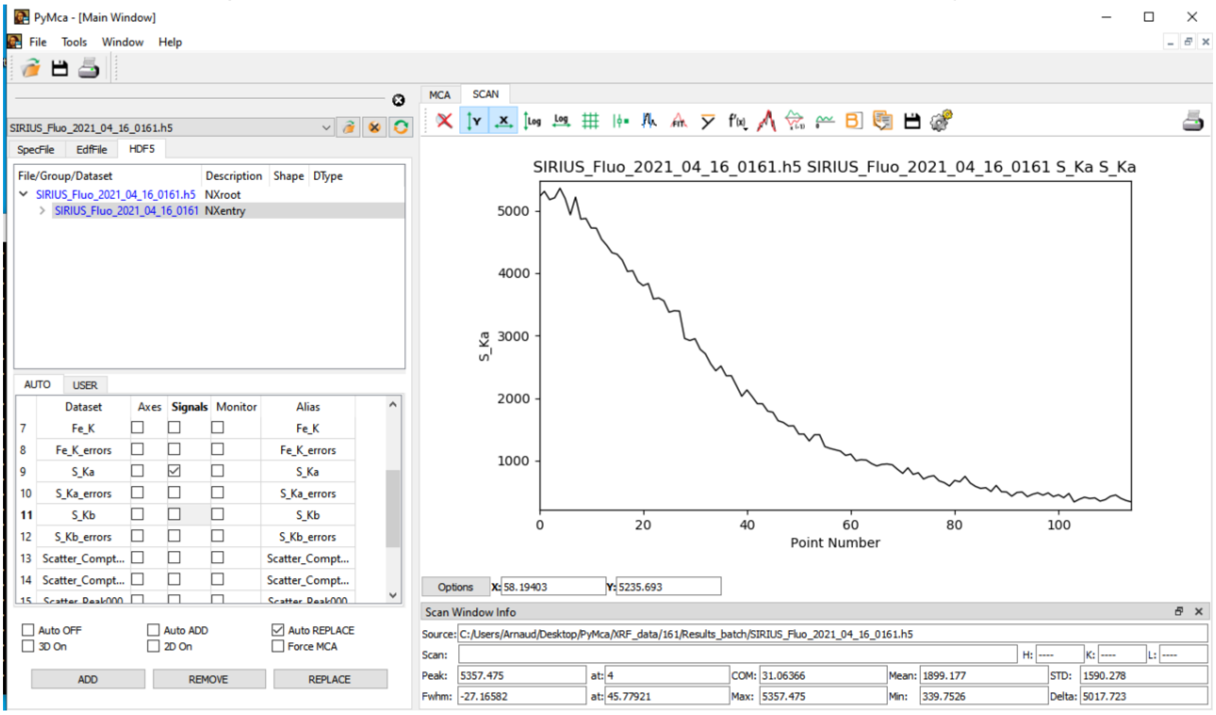

8.3. OPTIONAL: REMOVING THE SCATTERING PEAKS

PyMca explicitly does not aim at fitting well the scattering peaks, especially when they have this shape. The best in such a case is to restrain the range of the fit to exclude these peaks. Keeping bad fitted scattering peaks may affect the fit of other peaks as well.
The time-evolution of the elastic peak can be later obtained via a ROI summation, if it is needed for rescaling the peak areas afterwhile, for example.
