# Supplementary material for: Opportunities and new developments for the study of surfaces and interfaces in soft condensed matter at the SIRIUS beamline of Synchrotron SOLEIL
Source: J Synchrotron Radiat. 2024 Jan 1;31(Pt 1):162–76. doi: 10.1107/S1600577523008810 (PMC10833424; doi:10.1107/S1600577523008810)
Supplement: Supplementary file 1 [file s-31-00162-sup1.zip › JupyLabBook-v3.0.2/docs/XRF/comparison_jupyfluo_pymca.html]

comparison\_jupyfluo\_pymca


# Comparion JupyFluo and PyMca¶

The aim of this notebook is to compare quantitavely some results from fits usinf JupyFluo and PyMca. The conclusion is that the differences between the two approaches are negligible.

In [1]:

```
%%latex
\tableofcontents
\newpage
```

\tableofcontents
\newpage

In [2]:

```
# RUN THIS CELL TO START

__version__ = '0.6.1'
print("JupyFluo version: %s"%__version__)
print("More info on: %s"%"https://gitlab.com/soleil-data-treatment/soleil-beamlines/soleil-beamline-sirius/JupyFluo")

import matplotlib.pyplot as plt
%matplotlib inline
import numpy as np

# import custom libraries
from lib.frontend.experiment import Experiment
from lib.frontend.myWidgets import myWidgets
from lib.extraction import PyNexus as PN
import lib.frontend.main as FE

# To get vector plots in the PDF
import matplotlib_inline.backend_inline
matplotlib_inline.backend_inline.set_matplotlib_formats('png', 'pdf')

# Expand the result cells
from IPython.display import Javascript, display
display(Javascript('IPython.OutputArea.prototype._should_scroll = function(lines) {return false;}'))

# Name of the notebook (necessary for saving in pdf)
notebook_name = 'comparison_jupyfluo_pymca.ipynb'

# Directory where the data will be saved
save_dir = "save/"

# Directory where the nexus files are 
files_dir = "../files/"

# Logs depth level (default = 0, extensive = 1)
logs_lvl = 0

# Create the objects Experiment and myWidgets
expt = Experiment(notebook_name, save_dir, files_dir, logs_lvl) 
wid = myWidgets()

# Check the files and create the interactive cell
expt.check_and_init()
```

```
JupyFluo version: 0.6.1
More info on: https://gitlab.com/soleil-data-treatment/soleil-beamlines/soleil-beamline-sirius/JupyFluo
```

```
Results will be saved in the directory:
save/
Scans (nexus files) should be in the directory:
../files/
```

# SIRIUS\_Fluo\_2020\_02\_16\_02289¶

## Fit with JupyFluo¶

### Session ID: 20220207\_203848¶

Fit results for file `SIRIUS_Fluo_2020_02_16_02289.nxs`  
Parameters and results saved in:  
`save/SIRIUS_Fluo_2020_02_16_02289/20220207_203848/`

**Extraction parameters**  
Spectrum interval = [0, 1405]  
Channel interval = [140, 1250]  
SDD elements used = [4]

**Fit parameters**  
List of fitted parameters: ['sl', 'ct']  
beam energy = 12000; min. strength = 0.01  
**Params for conversion to eVs**  
gain = 9.9279; eV0 = -18.4341  
**Params for linear background**  
slope = 0.001; constant = 2.163  
Fit the background on the subset [4000 eV, 6000 eV]  
**Params for elastic peaks**  
noise = 0.1051; tail fraction (low energy side) = 0  
tail width (low energy side) = 0; shelf fraction = 0.6993  
**Params for Compton peaks**  
broadening factor = 1; tail fraction (low energy side) = 0.1089; tail fraction (high energy side) = 0  
tail width (low energy side) = 5; tail width (high energy side) = 0

In [3]:

```
fit_index=1000
path_to_result_folder='save/SIRIUS_Fluo_2020_02_16_02289/20220207_203848/'
expt.plot_all_fit_results(fit_index, path_to_result_folder)
```

## Comparison with PyMca¶

### Curves¶

In [4]:

```
file_curve_pymca = '../2289/Results_batch/FIT/SIRIUS_Fluo_2020_02_16_02289.nxs_FITDIR/SIRIUS_Fluo_2020_02_16_02289.nxs_1.0000.1000.fit'
with open(file_curve_pymca) as a_file:
    for line in a_file:
        if 'energy = [' in line:
            energy_pymca = [float(x)*1000. for x in line.split('energy = ')[1][2:-3].split(' ')]
        if 'ydata = [' in line:
            ydata_pymca = [float(x) for x in line.split('ydata = ')[1][2:-3].split(' ')]
        if 'yfit = [' in line:
            yfit_pymca = [float(x) for x in line.split('yfit = ')[1][2:-3].split(' ')]
            

file_curve_jupy = 'save/SIRIUS_Fluo_2020_02_16_02289/20220207_203848/fit_curves/spectrum_1000.csv'
curve_jupy = np.genfromtxt(file_curve_jupy, delimiter =';', names = True)

energy_jupy = curve_jupy['eVs']
ydata_jupy = curve_jupy['data']
yfit_jupy = curve_jupy['fit']
```

In [5]:

```
fig = plt.figure(figsize=(12,6))
plt.plot(energy_pymca, ydata_pymca, 'k.')
plt.plot(energy_pymca, yfit_pymca, 'r-', lw = 3, label = 'fit pymca')
plt.plot(energy_jupy, yfit_jupy, 'b-', lw = 3, label = 'fit jupyfluo')

#plt.plot(x, y_jupy*ratio, label = 'jupyfluo')
plt.yscale('log')
plt.ylim(1,1e4)
plt.legend()
plt.show()
```

### Fit results¶

In [6]:

```
data_pymca = np.genfromtxt('../2289/Results_batch/IMAGES/SIRIUS_Fluo_2020_02_16_02289.csv',
                     delimiter =';', names = True)[0:1406]
print('header pymca:\n',data_pymca.dtype.names)
data_jupy = np.genfromtxt('save/SIRIUS_Fluo_2020_02_16_02289/20220207_203848/fit_results.csv',
                           delimiter =';', names = True)
print('')
print('header jupyfluo:\n',data_jupy.dtype.names)
```

```
header pymca:
 ('row', 'column', 'S_Kb', 'Cl_Ka', 'Cl_Kb', 'Ar_K', 'Cr_K', 'Fe_K', 'Ni_K', 'Cu_K', 'Au_L', 'Au_M', 'Scatter_Peak000', 'Scatter_Compton000', 'sS_Kb', 'sCl_Ka', 'sCl_Kb', 'sAr_K', 'sCr_K', 'sFe_K', 'sNi_K', 'sCu_K', 'sAu_L', 'sAu_M', 'sScatter_Peak000', 'sScatter_Compton000', 'chisq')

header jupyfluo:
 ('ys', 'zs', 'xs', 'surfacepressure', 'areapermolecule', 'area', 'fluo01', 'fluo02', 'fluo03', 'fluo04', 'fluoicr04', 'fluoocr04', 'integration_time', 'sensorsRelTimestamps', 'sensorsTimestamps', 'spectrum_index', 'area_Elastic_El', 'stderr_area_Elastic_El', 'area_Compton_Co', 'stderr_area_Compton_Co', 'pos_peak_Compton_Co', 'stderr_pos_peak_Compton_Co', 'area_EscAu_AuL3M5Esc', 'stderr_area_EscAu_AuL3M5Esc', 'area_Cl_K', 'stderr_area_Cl_K', 'area_Fe_K', 'stderr_area_Fe_K', 'area_Cu_K', 'stderr_area_Cu_K', 'area_Au_L', 'stderr_area_Au_L', 'area_Au_M', 'stderr_area_Au_M', 'area_Cr_K', 'stderr_area_Cr_K', 'area_Pt_L', 'stderr_area_Pt_L', 'area_Ar_K', 'stderr_area_Ar_K', 'area_S_K', 'stderr_area_S_K', 'sl', 'stderr_sl', 'ct', 'stderr_ct')
```

#### Area Au¶

In [7]:

```
x = data_pymca['column']
y_pymca = data_pymca['Au_L']
y_jupy = data_jupy['area_Au_L']

# Average ratio to scale the two curves
ratio = np.nanmean(y_pymca/y_jupy)

fig = plt.figure(figsize=(12,6))
plt.plot(x, y_pymca, label = 'pymca')
plt.plot(x, y_jupy*ratio, label = 'jupyfluo')
plt.yscale('log')
plt.ylim(2e5,4.5e5)
plt.legend()
plt.show()
```

#### Area S¶

In [8]:

```
x = data_pymca['column']
y_pymca = data_pymca['S_Kb']
y_jupy = data_jupy['area_S_K']

# Average ratio to scale the two curves
ratio = np.nanmean(y_pymca/y_jupy)

fig = plt.figure(figsize=(12,6))
plt.plot(x, y_pymca, label = 'pymca')
plt.plot(x, y_jupy*ratio, label = 'jupyfluo')
plt.yscale('log')
plt.ylim(1e2,3e2)
plt.legend()
plt.show()
```

#### Area El¶

In [9]:

```
x = data_pymca['column']
y_pymca = data_pymca['Scatter_Peak000']
y_jupy = data_jupy['area_Elastic_El']

# Average ratio to scale the two curves
ratio = np.nanmean(y_pymca/y_jupy)

fig = plt.figure(figsize=(12,6))
plt.plot(x, y_pymca, label = 'pymca')
plt.plot(x, y_jupy*ratio, label = 'jupyfluo')
plt.yscale('log')
plt.ylim(4e3,1e5)
plt.legend()
plt.show()
```

#### Ratio Area Au / Area El¶

In [10]:

```
x = data_pymca['column']
y_pymca = data_pymca['Au_L']/data_pymca['Scatter_Peak000']
y_jupy = data_jupy['area_Au_L']/data_jupy['area_Elastic_El']

# Average ratio to scale the two curves
ratio = np.nanmean(y_pymca/y_jupy)

fig = plt.figure(figsize=(12,6))
plt.plot(x, y_pymca, label = 'pymca')
plt.plot(x, y_jupy*ratio, label = 'jupyfluo')
plt.yscale('log')
#plt.ylim(4e3,1e5)
plt.legend()
plt.show()
```

```
<ipython-input-10-e854a09a84d6>:2: RuntimeWarning: invalid value encountered in true_divide
  y_pymca = data_pymca['Au_L']/data_pymca['Scatter_Peak000']
```

# SIRIUS\_Fluo\_2021\_07\_10\_0172¶

## Fit with JupyFluo¶

### Session ID: 20220208\_203103¶

Fit results for file `SIRIUS_Fluo_2021_07_10_0172.nxs`  
Parameters and results saved in:  
`save/SIRIUS_Fluo_2021_07_10_0172/20220208_203103/`

**Extraction parameters**  
Spectrum interval = [0, 1201]  
Channel interval = [150, 1250]  
SDD elements used = [4]

**Fit parameters**  
List of fitted parameters: ['ct']  
beam energy = 12000; min. strength = 0.01  
**Params for conversion to eVs**  
gain = 9.928; eV0 = -19.6691  
**Params for linear background**  
slope = 0; constant = 1.5411  
Fit the background on the subset [3800 eV, 4100 eV]  
**Params for elastic peaks**  
noise = 0.1353; tail fraction (low energy side) = 0.05  
tail width (low energy side) = 1.3054; shelf fraction = 1  
**Params for Compton peaks**  
broadening factor = 1; tail fraction (low energy side) = 0.2647; tail fraction (high energy side) = 0  
tail width (low energy side) = 3; tail width (high energy side) = 0

In [11]:

```
fit_index=95
path_to_result_folder='save/SIRIUS_Fluo_2021_07_10_0172/20220208_203103/'
expt.plot_all_fit_results(fit_index, path_to_result_folder)
```

## Comparison with PyMca¶

### Curves¶

In [12]:

```
file_curve_pymca = '../172/Results_batch/FIT/SIRIUS_Fluo_2021_07_10_0172.nxs_FITDIR/SIRIUS_Fluo_2021_07_10_0172.nxs_1.0000.0095.fit'
with open(file_curve_pymca) as a_file:
    for line in a_file:
        if 'energy = [' in line:
            energy_pymca = [float(x)*1000. for x in line.split('energy = ')[1][2:-3].split(' ')]
        if 'ydata = [' in line:
            ydata_pymca = [float(x) for x in line.split('ydata = ')[1][2:-3].split(' ')]
        if 'yfit = [' in line:
            yfit_pymca = [float(x) for x in line.split('yfit = ')[1][2:-3].split(' ')]
            

file_curve_jupy = 'save/SIRIUS_Fluo_2021_07_10_0172/20220208_203103/fit_curves/spectrum_95.csv'
curve_jupy = np.genfromtxt(file_curve_jupy, delimiter =';', names = True)

energy_jupy = curve_jupy['eVs']
ydata_jupy = curve_jupy['data']
yfit_jupy = curve_jupy['fit']
```

In [13]:

```
fig = plt.figure(figsize=(12,6))
plt.plot(energy_pymca, ydata_pymca, 'k.')
plt.plot(energy_pymca, yfit_pymca, 'r-', lw = 3, label = 'fit pymca')
plt.plot(energy_jupy, yfit_jupy, 'b-', lw = 3, label = 'fit jupyfluo')

#plt.plot(x, y_jupy*ratio, label = 'jupyfluo')
plt.yscale('log')
plt.ylim(1,1e4)
plt.legend()
plt.show()
```

### Fit results¶

In [14]:

```
data_pymca = np.genfromtxt('../172/Results_batch/IMAGES/SIRIUS_Fluo_2021_07_10_0172.csv',
                     delimiter =';', names = True)
print('header pymca:\n',data_pymca.dtype.names)
data_jupy = np.genfromtxt('save/SIRIUS_Fluo_2021_07_10_0172/20220208_203103/fit_results.csv',
                           delimiter =';', names = True)
print('')
print('header jupyfluo:\n',data_jupy.dtype.names)
```

```
header pymca:
 ('row', 'column', 'S_Ka', 'S_Kb', 'Cl_Ka', 'Cl_Kb', 'Ar_K', 'Cr_K', 'Fe_K', 'Ni_K', 'Cu_K', 'Au_L', 'Au_M', 'Scatter_Peak000', 'Scatter_Compton000', 'sS_Ka', 'sS_Kb', 'sCl_Ka', 'sCl_Kb', 'sAr_K', 'sCr_K', 'sFe_K', 'sNi_K', 'sCu_K', 'sAu_L', 'sAu_M', 'sScatter_Peak000', 'sScatter_Compton000', 'chisq')

header jupyfluo:
 ('ys', 'zs', 'xs', 'surfacepressure', 'areapermolecule', 'fluo00', 'fluo01', 'fluo02', 'fluo03', 'fluoicr04', 'fluoocr04', 'qxy', 'm4pitch', 'integration_time', 'sensorsRelTimestamps', 'sensorsTimestamps', 'spectrum_index', 'area_Elastic_El', 'stderr_area_Elastic_El', 'area_Compton_Co', 'stderr_area_Compton_Co', 'pos_peak_Compton_Co', 'stderr_pos_peak_Compton_Co', 'area_EscEl_EscEl', 'stderr_area_EscEl_EscEl', 'pos_peak_EscEl_EscEl', 'stderr_pos_peak_EscEl_EscEl', 'area_EscAuL3M5_EscAuL3M5', 'stderr_area_EscAuL3M5_EscAuL3M5', 'area_Cl_K', 'stderr_area_Cl_K', 'area_Au_L', 'stderr_area_Au_L', 'area_Au_M', 'stderr_area_Au_M', 'area_Ar_K', 'stderr_area_Ar_K', 'area_Fe_K', 'stderr_area_Fe_K', 'area_Cr_K', 'stderr_area_Cr_K', 'area_Cu_K', 'stderr_area_Cu_K', 'area_S_K', 'stderr_area_S_K', 'ct', 'stderr_ct')
```

#### Area Au¶

In [15]:

```
x = data_pymca['column']
y_pymca = data_pymca['Au_L']
y_jupy = data_jupy['area_Au_L']

# Average ratio to scale the two curves
ratio = np.nanmean(y_pymca/y_jupy)

fig = plt.figure(figsize=(12,6))
plt.plot(x, y_pymca, label = 'pymca')
plt.plot(x, y_jupy*ratio, label = 'jupyfluo')
plt.yscale('log')
plt.ylim(6e4,8e4)
plt.legend()
plt.show()
```

#### Area Cl¶

In [16]:

```
x = data_pymca['column']
y_pymca = data_pymca['Cl_Ka']
y_jupy = data_jupy['area_Cl_K']

# Average ratio to scale the two curves
ratio = 145.

fig = plt.figure(figsize=(12,6))
plt.plot(x, y_pymca, label = 'pymca')
plt.plot(x, y_jupy*ratio, label = 'jupyfluo')
plt.yscale('log')
plt.ylim(4e2,1e3)
plt.legend()
plt.show()
```

#### Area El¶

In [17]:

```
x = data_pymca['column']
y_pymca = data_pymca['Scatter_Peak000']
y_jupy = data_jupy['area_Elastic_El']

# Average ratio to scale the two curves
ratio = np.nanmean(y_pymca/y_jupy)

fig = plt.figure(figsize=(12,6))
plt.plot(x, y_pymca, label = 'pymca')
plt.plot(x, y_jupy*ratio, label = 'jupyfluo')
plt.yscale('log')
plt.ylim(9e3,1.5e4)
plt.legend()
plt.show()
```

#### Ratio Area Au / Area El¶

In [18]:

```
x = data_pymca['column']
y_pymca = data_pymca['Au_L']/data_pymca['Scatter_Peak000']
y_jupy = data_jupy['area_Au_L']/data_jupy['area_Elastic_El']

# Average ratio to scale the two curves
ratio = np.nanmean(y_pymca/y_jupy)

fig = plt.figure(figsize=(12,6))
plt.plot(x, y_pymca, label = 'pymca')
plt.plot(x, y_jupy*ratio, label = 'jupyfluo')
plt.yscale('log')
plt.ylim(5,10)
plt.legend()
plt.show()
```

```
<ipython-input-18-27cf83ad59b6>:2: RuntimeWarning: invalid value encountered in true_divide
  y_pymca = data_pymca['Au_L']/data_pymca['Scatter_Peak000']
```

### Comparison with ROI (from PyMca)¶

In [19]:

```
data_pymca = np.genfromtxt('../172/Results_ROI/IMAGES/SIRIUS_Fluo_2021_07_10_0172_0100eVROI.csv',
                     delimiter =';', names = True)
print('header pymca:\n',data_pymca.dtype.names)
data_jupy = np.genfromtxt('save/SIRIUS_Fluo_2021_07_10_0172/20220208_203103/fit_results.csv',
                           delimiter =';', names = True)
print('')
print('header jupyfluo:\n',data_jupy.dtype.names)
```

```
header pymca:
 ('row', 'column', 'S_Ka_KL3a', 'S_Kb_KM3b', 'Cl_Ka_KL3a', 'Cl_Kb_KM3b', 'Ar_K_KL3', 'Ar_K_KM3', 'Cr_K_KL3', 'Cr_K_KM3', 'Fe_K_KL3', 'Fe_K_KM3', 'Ni_K_KL3', 'Ni_K_KM3', 'Cu_K_KL3', 'Cu_K_KM3', 'Au_L_L3M5', 'Au_L_L3N5', 'Au_L_L3M1', 'Au_L_L3O45', 'Au_M_M5N7', 'Au_M_M4N2', 'Au_M_M3N5', 'Au_M_M3N1', 'Au_M_M2N4')

header jupyfluo:
 ('ys', 'zs', 'xs', 'surfacepressure', 'areapermolecule', 'fluo00', 'fluo01', 'fluo02', 'fluo03', 'fluoicr04', 'fluoocr04', 'qxy', 'm4pitch', 'integration_time', 'sensorsRelTimestamps', 'sensorsTimestamps', 'spectrum_index', 'area_Elastic_El', 'stderr_area_Elastic_El', 'area_Compton_Co', 'stderr_area_Compton_Co', 'pos_peak_Compton_Co', 'stderr_pos_peak_Compton_Co', 'area_EscEl_EscEl', 'stderr_area_EscEl_EscEl', 'pos_peak_EscEl_EscEl', 'stderr_pos_peak_EscEl_EscEl', 'area_EscAuL3M5_EscAuL3M5', 'stderr_area_EscAuL3M5_EscAuL3M5', 'area_Cl_K', 'stderr_area_Cl_K', 'area_Au_L', 'stderr_area_Au_L', 'area_Au_M', 'stderr_area_Au_M', 'area_Ar_K', 'stderr_area_Ar_K', 'area_Fe_K', 'stderr_area_Fe_K', 'area_Cr_K', 'stderr_area_Cr_K', 'area_Cu_K', 'stderr_area_Cu_K', 'area_S_K', 'stderr_area_S_K', 'ct', 'stderr_ct')
```

#### Area Au¶

In [20]:

```
x = data_pymca['column']
y_pymca = data_pymca['Au_L_L3M5']
y_jupy = data_jupy['area_Au_L']

# Average ratio to scale the two curves
ratio = np.nanmean(y_pymca/y_jupy)

fig = plt.figure(figsize=(12,6))
plt.plot(x, y_pymca, label = 'pymca ROI')
plt.plot(x, y_jupy*ratio, label = 'jupyfluo')
plt.yscale('log')
plt.ylim(2e4,2.5e4)
plt.legend()
plt.show()
```

# SIRIUS\_Fluo\_2021\_04\_16\_0161¶

## Fit with JupyFluo¶

### Session ID: 20220215\_115348¶

Fit results for file `SIRIUS_Fluo_2021_04_16_0161.nxs`  
Parameters and results saved in:  
`save/SIRIUS_Fluo_2021_04_16_0161/20220215_115348/`

**Extraction parameters**  
Spectrum interval = [0, 114]  
Channel interval = [180, 830]  
SDD elements used = [4]

**Fit parameters**  
List of fitted parameters: ['ct']  
beam energy = 8000; min. strength = 0.01  
**Params for conversion to eVs**  
gain = 9.933; eV0 = -9.443  
**Params for linear background**  
slope = 0; constant = 9.6843  
Fit the background on the subset [4000 eV, 4600 eV]  
**Params for elastic peaks**  
noise = 0.1084; tail fraction (low energy side) = 0  
tail width (low energy side) = 0; shelf fraction = 1.8687  
**Params for Compton peaks**  
broadening factor = 1.437; tail fraction (low energy side) = 0.1342; tail fraction (high energy side) = 0  
tail width (low energy side) = 3.1664; tail width (high energy side) = 0

In [21]:

```
fit_index=10
path_to_result_folder='save/SIRIUS_Fluo_2021_04_16_0161/20220215_115348/'
expt.plot_all_fit_results(fit_index, path_to_result_folder)
```

## Comparison with PyMca¶

### Curves¶

In [22]:

```
file_curve_pymca = '../161/Results_batch/FIT/SIRIUS_Fluo_2021_04_16_0161.nxs_FITDIR/SIRIUS_Fluo_2021_04_16_0161.nxs_1.0000.0010.fit'
with open(file_curve_pymca) as a_file:
    for line in a_file:
        if 'energy = [' in line:
            energy_pymca = [float(x)*1000. for x in line.split('energy = ')[1][2:-3].split(' ')]
        if 'ydata = [' in line:
            ydata_pymca = [float(x) for x in line.split('ydata = ')[1][2:-3].split(' ')]
        if 'yfit = [' in line:
            yfit_pymca = [float(x) for x in line.split('yfit = ')[1][2:-3].split(' ')]
            

file_curve_jupy = 'save/SIRIUS_Fluo_2021_04_16_0161/20220215_115348/fit_curves/spectrum_10.csv'
curve_jupy = np.genfromtxt(file_curve_jupy, delimiter =';', names = True)

energy_jupy = curve_jupy['eVs']
ydata_jupy = curve_jupy['data']
yfit_jupy = curve_jupy['fit']
```

In [23]:

```
fig = plt.figure(figsize=(12,6))
plt.plot(energy_pymca, ydata_pymca, 'k.')
plt.plot(energy_pymca, yfit_pymca, 'r-', lw = 3, label = 'fit pymca')
plt.plot(energy_jupy, yfit_jupy, 'b-', lw = 3, label = 'fit jupyfluo')

#plt.plot(x, y_jupy*ratio, label = 'jupyfluo')
plt.yscale('log')
plt.ylim(1,1.5e4)
plt.legend()
plt.show()
```

### Fit results¶

In [24]:

```
data_pymca = np.genfromtxt('../161/Results_batch/IMAGES/SIRIUS_Fluo_2021_04_16_0161.csv',
                     delimiter =';', names = True)
print('header pymca:\n',data_pymca.dtype.names)
data_jupy = np.genfromtxt('save/SIRIUS_Fluo_2021_04_16_0161/20220215_115348/fit_results.csv',
                           delimiter =';', names = True)
print('')
print('header jupyfluo:\n',data_jupy.dtype.names)
```

```
header pymca:
 ('row', 'column', 'S_Ka', 'S_Kb', 'Cl_K', 'Ar_K', 'Ti_K', 'Cr_K', 'Fe_K', 'Scatter_Peak000', 'Scatter_Compton000', 'sS_Ka', 'sS_Kb', 'sCl_K', 'sAr_K', 'sTi_K', 'sCr_K', 'sFe_K', 'sScatter_Peak000', 'sScatter_Compton000', 'chisq')

header jupyfluo:
 ('ys', 'zs', 'xs', 'surfacepressure', 'areapermolecule', 'fluo00', 'fluo01', 'fluo02', 'fluo03', 'fluoicr04', 'fluoocr04', 'qxy', 'm4pitch', 'integration_time', 'sensorsRelTimestamps', 'sensorsTimestamps', 'spectrum_index', 'area_Elastic_El', 'stderr_area_Elastic_El', 'area_Compton_Co', 'stderr_area_Compton_Co', 'pos_peak_Compton_Co', 'stderr_pos_peak_Compton_Co', 'area_S_K', 'stderr_area_S_K', 'area_Cl_K', 'stderr_area_Cl_K', 'area_Ar_K', 'stderr_area_Ar_K', 'area_Ti_K', 'stderr_area_Ti_K', 'area_Cr_K', 'stderr_area_Cr_K', 'area_Fe_K', 'stderr_area_Fe_K', 'area_EscCo_EscCo', 'stderr_area_EscCo_EscCo', 'pos_peak_EscCo_EscCo', 'stderr_pos_peak_EscCo_EscCo', 'area_EscEl_EscEl', 'stderr_area_EscEl_EscEl', 'pos_peak_EscEl_EscEl', 'stderr_pos_peak_EscEl_EscEl', 'ct', 'stderr_ct')
```

#### Area S¶

In [25]:

```
x = data_pymca['column']
y_pymca = data_pymca['S_Ka']
y_jupy = data_jupy['area_S_K']

# Average ratio to scale the two curves
ratio = np.nanmean(y_pymca/y_jupy)

fig = plt.figure(figsize=(12,6))
plt.plot(x, y_pymca, label = 'pymca')
plt.plot(x, y_jupy*ratio, label = 'jupyfluo')
plt.yscale('log')
plt.legend()
plt.show()
```

#### Area Cl¶

In [26]:

```
x = data_pymca['column']
y_pymca = data_pymca['Cl_K']
y_jupy = data_jupy['area_Cl_K']

# Average ratio to scale the two curves
ratio = np.nanmean(y_pymca/y_jupy)

fig = plt.figure(figsize=(12,6))
plt.plot(x, y_pymca, label = 'pymca')
plt.plot(x, y_jupy*ratio, label = 'jupyfluo')
plt.yscale('log')
#plt.ylim(1e2,3e2)
plt.legend()
plt.show()
```

#### Area El¶

In [27]:

```
x = data_pymca['column']
y_pymca = data_pymca['Scatter_Peak000']
y_jupy = data_jupy['area_Elastic_El']

# Average ratio to scale the two curves
ratio = np.nanmean(y_pymca/y_jupy)

fig = plt.figure(figsize=(12,6))
plt.plot(x, y_pymca, label = 'pymca')
plt.plot(x, y_jupy*ratio, label = 'jupyfluo')
plt.yscale('log')
#plt.ylim(4e3,1e5)
plt.legend()
plt.show()
```

#### Area Compton¶

In [28]:

```
x = data_pymca['column']
y_pymca = data_pymca['Scatter_Compton000']
y_jupy = data_jupy['area_Compton_Co']

# Average ratio to scale the two curves
ratio = np.nanmean(y_pymca/y_jupy)

fig = plt.figure(figsize=(12,6))
plt.plot(x, y_pymca, label = 'pymca')
plt.plot(x, y_jupy*ratio, label = 'jupyfluo')
plt.yscale('log')
#plt.ylim(4e3,1e5)
plt.legend()
plt.show()
```

# Playing around with the hypermet function of PyMca¶

In [29]:

```
from silx.math.fit import sum_ahypermet
import matplotlib.pyplot as plt
%matplotlib inline
import numpy as np

x = np.arange(0,10,0.01)
area1 = 1.
position1 = 5
fwhm1 = 0.1
st_area_r1 = 1.
st_slope_r1 = 1.
lt_area_r1 = 10.
lt_slope_r1 = 10.
step_height_r1 = 0.
params = (area1, position1, fwhm1, st_area_r1, st_slope_r1, lt_area_r1, lt_slope_r1, step_height_r1)
peaks = sum_ahypermet(x, params, gaussian_term=True, st_term=True, lt_term=True, step_term=True)\
        + 1.

plt.yscale('log')
plt.xlim(1,6)
plt.plot(x, peaks)
```

Out[29]:

```
[<matplotlib.lines.Line2D at 0x7fa6beaf8d30>]
```
