# Supplementary material for: Opportunities and new developments for the study of surfaces and interfaces in soft condensed matter at the SIRIUS beamline of Synchrotron SOLEIL
Source: J Synchrotron Radiat. 2024 Jan 1;31(Pt 1):162–76. doi: 10.1107/S1600577523008810 (PMC10833424; doi:10.1107/S1600577523008810)
Supplement: Supplementary file 1 [file s-31-00162-sup1.zip › JupyLabBook-v3.0.2/docs/XRR_liquid/Howto_batch_XRR_liquid.html]

Howto\_batch\_XRR\_liquid


# Batch XRR liquid¶

We show here how to batch a series of XRR using the library `XRR_liquid.py` (JupyLabBook >= v2.10.5).

Each step is described in details in the notebook `Howto_data_reduction_XRR_liquid.ipynb`.

## User inputs¶

In [1]:

```
# Put the path to the folder containing /lib
# Here we start from JupyLabBook/docs/XRR/ and we want to go back to JupyLabBook/
import os
import numpy as np
os.chdir("../../")

# Import the library
from lib.extraction import XRR_liquid as XRR_liquid

# Directory where the nexus files are
recording_dir = '/nfs/ruche/sirius-soleil/com-sirius/commissionning/2021/week15/'

# To get the list of XRRs
from glob import glob
list_files = np.array(glob(recording_dir+"*files*"))
list_files.sort()
display([file.split('/')[-1].split('_XRR_files.dat')[0]+'.nxs' for file in list_files])

# Directory where the data will be saved (the directory must exist)
working_dir = ''

# List of XRR to treat (give the name of the first nexus scan)
list_first_XRR_filename = ['SIRIUS_2021_04_14_4254.nxs',
                           'SIRIUS_2021_04_14_4346.nxs',
                           'SIRIUS_2021_04_14_4390.nxs']


# List of corresponding direct scans
list_direct_filename = ['SIRIUS_2021_04_14_4253.nxs',
                        'SIRIUS_2021_04_14_4345.nxs',
                        'SIRIUS_2021_04_14_4389.nxs']

# Full scan ROI
ROIx0=560
ROIy0=905
ROIsizex=21
ROIsizey=40

# Height of the summation ROI
# Always use an odd number!
summation_ROIsizey = 5

# Value of m4pitch0 (in deg)
m4pitch0 = -0.0375

# lambda (in nm)
wavelength = 0.155

# Define ROIs for background
is_bckg_up = True
is_bckg_down = True
is_bckg_left = False
is_bckg_right = False

# Track the vetical position of the beam?
is_tracking = True
```

```
['SIRIUS_2021_04_13_4084.nxs',
 'SIRIUS_2021_04_13_4088.nxs',
 'SIRIUS_2021_04_13_4092.nxs',
 'SIRIUS_2021_04_13_4097.nxs',
 'SIRIUS_2021_04_13_4110.nxs',
 'SIRIUS_2021_04_13_4147.nxs',
 'SIRIUS_2021_04_14_4191.nxs',
 'SIRIUS_2021_04_14_4208.nxs',
 'SIRIUS_2021_04_14_4218.nxs',
 'SIRIUS_2021_04_14_4241.nxs',
 'SIRIUS_2021_04_14_4246.nxs',
 'SIRIUS_2021_04_14_4249.nxs',
 'SIRIUS_2021_04_14_4254.nxs',
 'SIRIUS_2021_04_14_4298.nxs',
 'SIRIUS_2021_04_14_4346.nxs',
 'SIRIUS_2021_04_14_4390.nxs',
 'SIRIUS_2021_04_15_4435.nxs']
```

## Loop over the files¶

In [3]:

```
for i in range(len(list_first_XRR_filename)):
    
    nxs_filename = list_first_XRR_filename[i]
    direct_nxs_filename = list_direct_filename[i]
    
    print('Treating XRR %s'%nxs_filename)

    m4pitch, theta, qz, bckg_R_up, bckg_R_down, bckg_R_left, bckg_R_right, bckg_R, err_R, R = \
    XRR_liquid.Treat(nxs_filename, recording_dir, \
    direct_nxs_filename, ROIx0, ROIy0, ROIsizex, ROIsizey, \
    summation_ROIsizey, m4pitch0, wavelength, force_direct=False, fdirect=1.0, \
    is_bckg_up=is_bckg_up, is_bckg_down=is_bckg_down,
    is_bckg_left=is_bckg_left, is_bckg_right=is_bckg_right, is_tracking=is_tracking,
    working_dir=working_dir, plot_XRR_m4pitch=False, plot_XRR_qz=False, plot_pos_y=False, save=True, verbose=False)
    
    print('')
```

```
Treating XRR SIRIUS_2021_04_14_4254.nxs
Direct extracted from SIRIUS_2021_04_14_4253.nxs: direct=2.40925e+12
                              
Treating XRR SIRIUS_2021_04_14_4346.nxs
Direct extracted from SIRIUS_2021_04_14_4345.nxs: direct=1.07108e+12
                              
Treating XRR SIRIUS_2021_04_14_4390.nxs
Direct extracted from SIRIUS_2021_04_14_4389.nxs: direct=2.38013e+12
```

## Plot a specific result¶

In [5]:

```
import numpy as np

for i in range(len(list_first_XRR_filename)):
    nxs_filename = list_first_XRR_filename[i]

    data_filename = working_dir+nxs_filename[:-4]+'_XRR.dat'

    [m4pitch, theta, qz, pos_y, bckg_R_up, bckg_R_down, bckg_R_left, bckg_R_right, bckg_R, err_R, R] =\
    np.loadtxt(data_filename, delimiter = '\t').transpose()

    XRR_liquid.Plot(m4pitch, theta, qz, pos_y, bckg_R_up, bckg_R_down, bckg_R_left, bckg_R_right, bckg_R, err_R, R,\
             nxs_filename, plot_XRR_m4pitch=False, plot_XRR_qz=True, plot_pos_y=True)
```

In [ ]:

```

```
