# Supplementary material for: Opportunities and new developments for the study of surfaces and interfaces in soft condensed matter at the SIRIUS beamline of Synchrotron SOLEIL
Source: J Synchrotron Radiat. 2024 Jan 1;31(Pt 1):162–76. doi: 10.1107/S1600577523008810 (PMC10833424; doi:10.1107/S1600577523008810)
Supplement: Supplementary file 1 [file s-31-00162-sup1.zip › JupyLabBook-v3.0.2/docs/XRR_liquid/Howto_data_reduction_XRR_liquid.html]

Howto\_data\_reduction\_XRR\_liquid


# Description of the data reduction process for XRR on a liquid surface¶

This notebook explains the different steps performed to extract the X-ray reflectivity in JupyLabBook >= v2.10.5 with the library `XRR_liquid.py`.

# Conditions¶

The conditions for the examples treated here are:

- A 2D detector (Pilatus or UFXC) is on the delta-gamma arm.
- A time scan of the direct beam has been performed.
- The ionization chamber has been mounted before the sample.
- A file `FIRSTSCAN_XRR_files.dat` exists in your recording folder, which contains the list of scans for one XRR curve.
- The direct scan and each scan of the XRR have a companion file `SCAN_gains.dat`, wich contains the voltage of the ionization chamber for each of its gains.

# Extraction of the intensity of the reflected beam¶

Each point of the XRR curve results from a time scan on the detector. We describe here how the reflected beam is located on the 2D images, and how its raw intensity is extracted.

## User-defined full scan ROI¶

The user defines the ROI (Region Of Interest) in which **all the reflected beams of a XRR curve** falls. It means that the user should check that this ROI includes the reflected beams of each scan of the XRR.

In [1]:

```
# User defined full scan ROI
ROIx0=560
ROIy0=905
ROIsizex=21
ROIsizey=40
```

### Control on one scan¶

We check that the ROI is right for one scan, i.e. one data angle of the XRR curve.  
We use the library `DetectorSum.py`to extract the 2D images from the nexus file of a scan. All the images within a same time scan are summed.

In [2]:

```
# Put the path to the folder containing /lib
# Here we start from JupyLabBook/docs/XRR/ and we want to go back to JupyLabBook/
import os
os.chdir("../../")

from lib.extraction import DetectorSum as DetectorSum
import numpy as np
import matplotlib.pyplot as plt
import matplotlib.colors as colors

recording_dir = 'not_in_git/recording/XRR_liquid_pilatus/'
nxs_filename = 'SIRIUS_2021_04_14_4256.nxs'


# Extract the sum of all images in the scan
_, image, _, _, _, _, _, _  =DetectorSum.Extract(nxs_filename, recording_dir,
                                                 show_data_stamps=False, verbose=False)

# Replace the intensity of the dead zones with a value of 0
image=np.where(image<0., 0., image)
```

```

```

In [3]:

```
# Extract the full scan ROI
# Full image: ROI = [0, 0, 981, 1043]
ROI = [ROIx0, ROIy0, ROIsizex, ROIsizey]

#Apply the ROI
image_ROI = image[ROI[1]:ROI[1]+ROI[3], ROI[0]:ROI[0]+ROI[2]]

# Show the ROI image (summed over the time scan)
#fig, ax0  = plt.subplots(figsize=(15,20))
fig, ax0  = plt.subplots(figsize=(15,4))

im = ax0.pcolormesh(image_ROI, cmap = 'jet', shading = 'auto', rasterized=True)

fig.colorbar(im, ax=ax0)
ax0.set(xlabel = 'horizontal pixel (x)', ylabel ='vertical pixel (y)')
ax0.invert_yaxis()
plt.show()
```

### Control on all the scans¶

We check on every scan of the XRR that the reflected beam falls in the full scan ROI.

In [4]:

```
# The nxs_filename should be the first XRR, with a companion file XXX_XRR_files.dat
nxs_filename = 'SIRIUS_2021_04_14_4254.nxs'
original_nxs_filename = nxs_filename

files_path = recording_dir+nxs_filename[:-4]+'_XRR_files.dat'
file_list = np.genfromtxt(files_path,dtype='U')

for file in file_list:
    
    _, image, _, _, _, _, _, _ = DetectorSum.Extract(file+'.nxs', recording_dir,
                                                     show_data_stamps=False, verbose=False)
  
    # Replace the intensity of the dead zones with a value of 0
    image=np.where(image<0., 0., image)

    # Apply the ROI
    image_ROI = image[ROI[1]:ROI[1]+ROI[3], ROI[0]:ROI[0]+ROI[2]]

    # Show the full image integrated over the scan
    fig, ax0  = plt.subplots(figsize=(15,3))
    #fig, ax0  = plt.subplots(figsize=(15,15))
    im = ax0.pcolormesh(image_ROI, norm = colors.LogNorm(),
                        cmap = 'jet', shading = 'auto', rasterized=True)

    ax0.invert_yaxis()
    plt.show()
```

```

```

```

```

```

```

```

```

```

```

```

```

```

```

```

```

```

```

```

```

```

```

```

```

```

```

```

```

```

```

```

```

```

```

```

```

```

```

```

```

```

```

```

```

```

```

```

```

```

```

```

```

```

```

```

```

```

```

```

```

```

```

```

```

```

```

```

```

```

```

```

```

```

```

```

```

```

```

```

```

```

```

```

```

## Finding the vertical position of the reflected beam¶

It may happen that the reflected beam moves vertically on the Pilatus from one scan to another, within the full scan ROI.  
For proper measurement of the intensity we need to center the summation ROI on the reflected beam.  
We find the reflected beam following this routine:

- Take the position of the pixel with the highest intensity within the full scan ROI, after integration along the horizontal direction.
- If the position of the maximum if too far from the prediction of a linear interpolation of the previous positions, we take the prediction instead.
- Perform a rocking curve aroung this position, the reflected beam is located at the maximum of the rocking curve.

The user defines the height of the summation ROI, **which will be summed to extract the intensity of the reflected beam**. Careful, the larger the ROI, the more background you add to the signal. **Also, the height has to be an odd number, to have the beam centered.**

NB: it does not seem reasonable to fit the beam with a gaussian or a step function, as its width is usually of only one or two pixels on the Pilatus detector (pixel size of 172 microns).

In [5]:

```
# User defined height of the summation ROI
# Always use an odd number!
summation_ROIsizey = 7
```

### Extract the raw intensity of the reflected beam¶

For each scan of the XRR we then extract the raw intensity of the reflected beam by summing over the summation ROI.  
We normalize each point by the integration time and the number of points in the scan.

In [6]:

```
from lib.extraction.common import PyNexus as PN

I_raw_refl = np.array([])
pos_y_list = np.array([])

# height of the summation ROI
dy = summation_ROIsizey

# width of the summation ROI
dx = ROIsizex

#for file in file_list:
for file in file_list:    
    
    _, image, _, _, _, _, _, _ = DetectorSum.Extract(file+'.nxs', recording_dir,
                                                     show_data_stamps=False, verbose=False)
    
    # Replace the intensity of the dead zones with a value of 0
    image=np.where(image<0., 0., image)
    
    # Apply the full scan ROI
    image_ROI = image[ROI[1]:ROI[1]+ROI[3], ROI[0]:ROI[0]+ROI[2]]

    # Integrate along the horizontal axis and find the maximum
    integrated_x = image_ROI.sum(axis=1)
    pos_max_y = np.argmax(integrated_x)+ROI[1]
    
    print(file+'\nPosition maximum = %s'%pos_max_y) 
    
    # Prediction of the pos_y based on linear interpolation of previous ones
    if len(pos_y_list)>5:
        a, b = np.polyfit(np.arange(len(pos_y_list)), pos_y_list, 1)
        pos_pred_y = int(a*np.arange(len(pos_y_list)+1)[-1]+b)
        print('Position given by the linear interpolation = %s'%pos_pred_y)
        
        # If there is a shift of more than 2 pixels with the prediction, take the prediction
        if np.abs(pos_pred_y-pos_max_y)>2:
            pos_y0 = pos_pred_y
        else:
            pos_y0 = pos_max_y
            
            
        # Do a rocking curve and find the max
        print('Chosen position for performing a rocking curve = %s'%pos_y0)    
        summation_ROI0 = image[pos_y0-dy//2 : pos_y0+dy//2+1,
                               ROI[0] : ROI[0]+ROI[2]]

        # Move to the max of the rocking curve
        pos_max_rc = np.argmax(summation_ROI0.sum(axis=1))
        pos_y_rc = pos_y0-dy//2+pos_max_rc
            
        print('Position given by the rocking curve = %s'%pos_y_rc)

        # If there is a shift of more than 2 pixels with the prediction, take the prediction
        if np.abs(pos_pred_y-pos_y_rc)>2:
            pos_y = pos_pred_y
            print('Take position from linear interpolation.')
        else:
            pos_y = pos_y_rc
            print('Take position from rocking curve.')
        
        # Plot the rocking curve
        fig, ax  = plt.subplots(figsize=(15,4))
        plt.plot(pos_max_rc, summation_ROI0.sum(axis=1)[pos_max_rc], 'gd',
                 label = 'Max rocking curve')
        plt.plot(pos_pred_y-pos_y0+dy//2, summation_ROI0.sum(axis=1)[pos_pred_y-pos_y0+dy//2], 'ro',
                 label = 'Pred. interp.')
        plt.plot(pos_y-pos_y0+dy//2, summation_ROI0.sum(axis=1)[pos_y-pos_y0+dy//2], 'kx',
                     label = 'Chosen position')
        plt.plot(summation_ROI0.sum(axis=1), 'b.-')
        plt.legend()
        plt.show()                 
            
    else:
        print('Take the position of the maximum for the first points.')
        pos_y = pos_max_y
                       
    print('')
    
    summation_ROI = image[pos_y-dy//2 : pos_y+dy//2+1,
                       ROI[0] : ROI[0]+ROI[2]]
    
    pos_y_list = np.append(pos_y_list, int(pos_y))
    
    # Extract info from nexus file
    nexus = PN.PyNexusFile(recording_dir+file+'.nxs', fast=True)
    stamps0D, data0D = nexus.extractData("0D")
    nbpts=int(nexus.get_nbpts())
    sensor_list = [stamps0D[i][0] if stamps0D[i][1]== None else stamps0D[i][1] for i in range(len(stamps0D))]

    integration_timeArg = sensor_list.index('integration_time')
    integration_time = np.mean(data0D[integration_timeArg])
            
    # Sum the ROI and normalize with the integration time and the number of images
    I_raw_refl = np.append(I_raw_refl,summation_ROI.sum(axis=0).sum(axis=0)/integration_time/nbpts)
```

```
SIRIUS_2021_04_14_4254        
Position maximum = 926
Take the position of the maximum for the first points.

SIRIUS_2021_04_14_4255        
Position maximum = 926
Take the position of the maximum for the first points.

SIRIUS_2021_04_14_4256        
Position maximum = 926
Take the position of the maximum for the first points.

SIRIUS_2021_04_14_4257        
Position maximum = 926
Take the position of the maximum for the first points.

SIRIUS_2021_04_14_4258        
Position maximum = 926
Take the position of the maximum for the first points.

SIRIUS_2021_04_14_4259        
Position maximum = 925
Take the position of the maximum for the first points.

SIRIUS_2021_04_14_4260        
Position maximum = 925
Position given by the linear interpolation = 925
Chosen position for performing a rocking curve = 925
Position given by the rocking curve = 925
Take position from rocking curve.
```

```
SIRIUS_2021_04_14_4261        
Position maximum = 925
Position given by the linear interpolation = 924
Chosen position for performing a rocking curve = 925
Position given by the rocking curve = 925
Take position from rocking curve.
```

```
SIRIUS_2021_04_14_4262        
Position maximum = 925
Position given by the linear interpolation = 924
Chosen position for performing a rocking curve = 925
Position given by the rocking curve = 925
Take position from rocking curve.
```

```
SIRIUS_2021_04_14_4263        
Position maximum = 925
Position given by the linear interpolation = 924
Chosen position for performing a rocking curve = 925
Position given by the rocking curve = 925
Take position from rocking curve.
```

```
SIRIUS_2021_04_14_4264        
Position maximum = 925
Position given by the linear interpolation = 924
Chosen position for performing a rocking curve = 925
Position given by the rocking curve = 925
Take position from rocking curve.
```

```
SIRIUS_2021_04_14_4265        
Position maximum = 925
Position given by the linear interpolation = 924
Chosen position for performing a rocking curve = 925
Position given by the rocking curve = 925
Take position from rocking curve.
```

```
SIRIUS_2021_04_14_4266        
Position maximum = 925
Position given by the linear interpolation = 924
Chosen position for performing a rocking curve = 925
Position given by the rocking curve = 925
Take position from rocking curve.
```

```
SIRIUS_2021_04_14_4267        
Position maximum = 925
Position given by the linear interpolation = 924
Chosen position for performing a rocking curve = 925
Position given by the rocking curve = 925
Take position from rocking curve.
```

```
SIRIUS_2021_04_14_4268        
Position maximum = 925
Position given by the linear interpolation = 924
Chosen position for performing a rocking curve = 925
Position given by the rocking curve = 925
Take position from rocking curve.
```

```
SIRIUS_2021_04_14_4269        
Position maximum = 925
Position given by the linear interpolation = 924
Chosen position for performing a rocking curve = 925
Position given by the rocking curve = 925
Take position from rocking curve.
```

```
SIRIUS_2021_04_14_4270        
Position maximum = 925
Position given by the linear interpolation = 924
Chosen position for performing a rocking curve = 925
Position given by the rocking curve = 925
Take position from rocking curve.
```

```
SIRIUS_2021_04_14_4271        
Position maximum = 925
Position given by the linear interpolation = 924
Chosen position for performing a rocking curve = 925
Position given by the rocking curve = 925
Take position from rocking curve.
```

```
SIRIUS_2021_04_14_4272        
Position maximum = 925
Position given by the linear interpolation = 924
Chosen position for performing a rocking curve = 925
Position given by the rocking curve = 925
Take position from rocking curve.
```

```
SIRIUS_2021_04_14_4273        
Position maximum = 924
Position given by the linear interpolation = 924
Chosen position for performing a rocking curve = 924
Position given by the rocking curve = 924
Take position from rocking curve.
```

```
SIRIUS_2021_04_14_4274        
Position maximum = 925
Position given by the linear interpolation = 924
Chosen position for performing a rocking curve = 925
Position given by the rocking curve = 925
Take position from rocking curve.
```

```
SIRIUS_2021_04_14_4275        
Position maximum = 924
Position given by the linear interpolation = 924
Chosen position for performing a rocking curve = 924
Position given by the rocking curve = 924
Take position from rocking curve.
```

```
SIRIUS_2021_04_14_4276        
Position maximum = 924
Position given by the linear interpolation = 924
Chosen position for performing a rocking curve = 924
Position given by the rocking curve = 924
Take position from rocking curve.
```

```
SIRIUS_2021_04_14_4277        
Position maximum = 924
Position given by the linear interpolation = 924
Chosen position for performing a rocking curve = 924
Position given by the rocking curve = 924
Take position from rocking curve.
```

```
SIRIUS_2021_04_14_4278        
Position maximum = 924
Position given by the linear interpolation = 924
Chosen position for performing a rocking curve = 924
Position given by the rocking curve = 924
Take position from rocking curve.
```

```
SIRIUS_2021_04_14_4279        
Position maximum = 924
Position given by the linear interpolation = 924
Chosen position for performing a rocking curve = 924
Position given by the rocking curve = 924
Take position from rocking curve.
```

```
SIRIUS_2021_04_14_4280        
Position maximum = 924
Position given by the linear interpolation = 923
Chosen position for performing a rocking curve = 924
Position given by the rocking curve = 924
Take position from rocking curve.
```

```
SIRIUS_2021_04_14_4281        
Position maximum = 924
Position given by the linear interpolation = 923
Chosen position for performing a rocking curve = 924
Position given by the rocking curve = 924
Take position from rocking curve.
```

```
SIRIUS_2021_04_14_4282        
Position maximum = 924
Position given by the linear interpolation = 923
Chosen position for performing a rocking curve = 924
Position given by the rocking curve = 924
Take position from rocking curve.
```

```
SIRIUS_2021_04_14_4283        
Position maximum = 923
Position given by the linear interpolation = 923
Chosen position for performing a rocking curve = 923
Position given by the rocking curve = 923
Take position from rocking curve.
```

```
SIRIUS_2021_04_14_4284        
Position maximum = 923
Position given by the linear interpolation = 923
Chosen position for performing a rocking curve = 923
Position given by the rocking curve = 923
Take position from rocking curve.
```

```
SIRIUS_2021_04_14_4285        
Position maximum = 923
Position given by the linear interpolation = 923
Chosen position for performing a rocking curve = 923
Position given by the rocking curve = 923
Take position from rocking curve.
```

```
SIRIUS_2021_04_14_4286        
Position maximum = 923
Position given by the linear interpolation = 923
Chosen position for performing a rocking curve = 923
Position given by the rocking curve = 923
Take position from rocking curve.
```

```
SIRIUS_2021_04_14_4287        
Position maximum = 923
Position given by the linear interpolation = 923
Chosen position for performing a rocking curve = 923
Position given by the rocking curve = 923
Take position from rocking curve.
```

```
SIRIUS_2021_04_14_4288        
Position maximum = 923
Position given by the linear interpolation = 923
Chosen position for performing a rocking curve = 923
Position given by the rocking curve = 923
Take position from rocking curve.
```

```
SIRIUS_2021_04_14_4289        
Position maximum = 923
Position given by the linear interpolation = 922
Chosen position for performing a rocking curve = 923
Position given by the rocking curve = 923
Take position from rocking curve.
```

```
SIRIUS_2021_04_14_4290        
Position maximum = 923
Position given by the linear interpolation = 922
Chosen position for performing a rocking curve = 923
Position given by the rocking curve = 923
Take position from rocking curve.
```

```
SIRIUS_2021_04_14_4291        
Position maximum = 922
Position given by the linear interpolation = 922
Chosen position for performing a rocking curve = 922
Position given by the rocking curve = 922
Take position from rocking curve.
```

```
SIRIUS_2021_04_14_4292        
Position maximum = 922
Position given by the linear interpolation = 922
Chosen position for performing a rocking curve = 922
Position given by the rocking curve = 922
Take position from rocking curve.
```

```
SIRIUS_2021_04_14_4293        
Position maximum = 922
Position given by the linear interpolation = 922
Chosen position for performing a rocking curve = 922
Position given by the rocking curve = 922
Take position from rocking curve.
```

```
SIRIUS_2021_04_14_4294        
Position maximum = 922
Position given by the linear interpolation = 922
Chosen position for performing a rocking curve = 922
Position given by the rocking curve = 922
Take position from rocking curve.
```

```
SIRIUS_2021_04_14_4295        
Position maximum = 922
Position given by the linear interpolation = 922
Chosen position for performing a rocking curve = 922
Position given by the rocking curve = 922
Take position from rocking curve.
```

```

```

### Control on one scan¶

We check that the beam is within the summation ROI.

In [7]:

```
# We now choose the scan to plot by refering directly to the file_list
i = 15

nxs_filename = file_list[i]+'.nxs'

print('Check summation ROI on scan %s'%nxs_filename)

_, image, _, _, _, _, _, _ = DetectorSum.Extract(nxs_filename, recording_dir,
                                                 show_data_stamps=False, verbose=False)

# Replace the intensity of the dead zones with a value of 0
image=np.where(image<0., 0., image)
    
# Apply the full scan ROI
image_ROI = image[ROI[1]:ROI[1]+ROI[3], ROI[0]:ROI[0]+ROI[2]]

# Vertical position of the beam
pos_y = int(pos_y_list[i])

print('Vertical position beam = %s'%pos_y)

summation_ROI = image[pos_y-dy//2 : pos_y+dy//2+1,
                      ROI[0] : ROI[0]+ROI[2]]

print('Summation ROI = (%s, %s); (%s, %s)'%(pos_y-dy//2,ROI[0],pos_y+dy//2,ROI[0]+dx-1))

fig, ax0  = plt.subplots(figsize=(15,4))
im = ax0.pcolormesh(summation_ROI, cmap = 'jet', shading = 'auto', rasterized=True)
fig.colorbar(im, ax=ax0)
ax0.set_title('Lin scale', fontsize=16)
ax0.set(xlabel = 'horizontal pixel (x)', ylabel ='vertical pixel (y)')
ax0.invert_yaxis()
plt.show()

fig, ax0  = plt.subplots(figsize=(15,4))
im = ax0.pcolormesh(summation_ROI, norm = colors.LogNorm(), cmap = 'jet', shading = 'auto', rasterized=True)
fig.colorbar(im, ax=ax0)
ax0.set_title('Log scale', fontsize=16)
ax0.set(xlabel = 'horizontal pixel (x)', ylabel ='vertical pixel (y)')
ax0.invert_yaxis()
plt.show()
```

```
Check summation ROI on scan SIRIUS_2021_04_14_4269.nxs
Vertical position beam = 925  
Summation ROI = (922, 560); (928, 580)
```

We plot the extracted raw intensities, which looks weird because the attenuators were adjusted from one scan to another to avoid beam damage on the detector. This will be adjusted by the normalization with the incident beam.  
We also plot the position of the reflected beam. If everything went well, this should be a straight line with jumps of maximum two pixels.

In [8]:

```
fig = plt.figure(figsize=(12,5))
ax=fig.add_subplot(111)
ax.set_title('Raw intensity', fontsize=16)
plt.yscale('log')
plt.plot(I_raw_refl, 'x-k')
ax.set_xlabel('Scan index in the list', fontsize=16)
ax.set_ylabel('I_raw_refl', fontsize=16)
fig.subplots_adjust(top=0.9)
ax.tick_params(labelsize=16)
ax.yaxis.offsetText.set_fontsize(16)
plt.show()


fig = plt.figure(figsize=(12,5))
ax=fig.add_subplot(111)
ax.set_title('Position of the reflected beam', fontsize=16)
plt.plot(pos_y_list, 'x-k')
ax.set_xlabel('Scan index in the list', fontsize=16)
ax.set_ylabel('Pixel', fontsize=16)
fig.subplots_adjust(top=0.9)
ax.tick_params(labelsize=16)
ax.yaxis.offsetText.set_fontsize(16)
plt.show()
```

# Background subtraction¶

The background is defined by taking a ROI of the same size as the summation ROI, eather immediately above and below it, or on its left and right.  
**The user chooses which background is taken:**

- up & down, the most general case.
- up or down, e.g. in case of strong asymmetry or trace of the direct beam.
- left & right, e.g. if there is a strong off-specular scattering.

In [9]:

```
# Choose which background to take
is_bckg_up = True
is_bckg_down = True
is_bckg_left = False
is_bckg_right = False
```

### Control on one scan¶

We check the background ROIs in linear and log scales.

In [10]:

```
import matplotlib.patches as patches

i = 13

nxs_filename = file_list[i]+'.nxs'

print('Check background ROIs on scan %s'%nxs_filename)

_, image, _, _, _, _, _, _ = DetectorSum.Extract(nxs_filename, recording_dir,
                                                 show_data_stamps=False, verbose=False)

# Replace the intensity of the dead zones with a value of 0
image=np.where(image<0., 0., image)  
    
# Apply the full scan ROI
image_ROI = image[ROI[1]:ROI[1]+ROI[3], ROI[0]:ROI[0]+ROI[2]]

# Vertical position of the beam
pos_y = int(pos_y_list[i])

# height of the summation ROI
dy = summation_ROIsizey

# width of the summation ROI
dx = ROIsizex

print('Vertical position beam = %s'%pos_y)

print('Positions ROIs: up left corner, included, (y, x); bottom right corner, included, (y, x)')

# Summation ROI
summation_ROI = image[pos_y-dy//2 : pos_y+dy//2+1,
                      ROI[0] : ROI[0]+dx]
print('Summation ROI = (%s, %s); (%s, %s)'%(pos_y-dy//2,ROI[0],pos_y+dy//2,ROI[0]+dx-1))

# Up & down background ROIs
bckg_ROI_up = image[pos_y-dy-dy//2 : pos_y-dy+dy//2+1,
                    ROI[0] : ROI[0]+dx]
print('Up bckg ROI = (%s, %s); (%s, %s)'%(pos_y-dy-dy//2,ROI[0],pos_y-dy+dy//2,ROI[0]+dx-1))


bckg_ROI_down = image[pos_y+dy-dy//2 : pos_y+dy+dy//2+1,
                      ROI[0] : ROI[0]+dx]

print('Down bckg ROI = (%s, %s); (%s, %s)'%(pos_y+dy-dy//2,ROI[0],pos_y+dy+dy//2,ROI[0]+dx-1))

# Left & right background ROIs
bckg_ROI_left = image[pos_y-dy//2 : pos_y+dy//2+1,
                      ROI[0]-dx : ROI[0]]
print('Left bckg ROI = (%s, %s); (%s, %s)'%(pos_y-dy//2,ROI[0]-dx,pos_y+dy//2,ROI[0]-1))

bckg_ROI_right = image[pos_y-dy//2 : pos_y+dy//2+1,
                      ROI[0]+dx : ROI[0]+2*dx]
print('Right bckg ROI = (%s, %s); (%s, %s)'%(pos_y-dy//2,ROI[0]+dx,pos_y+dy//2,ROI[0]+2*dx-1))


# Draw a larger ROI for display of the background
image_ROI_display = image[pos_y-dy-dy//2 : pos_y+dy+dy//2+1,
                          ROI[0]-dx : ROI[0]+2*dx]    


fig, ax0  = plt.subplots(figsize=(15,4))

rect_up = patches.Rectangle((dx,0), dx, dy, linewidth=2, edgecolor='r', facecolor='none')
rect_down = patches.Rectangle((dx,2*dy), dx, dy, linewidth=2, edgecolor='r', facecolor='none')
rect_left = patches.Rectangle((0,dy), dx, dy, linewidth=2, edgecolor='w', facecolor='none')
rect_right = patches.Rectangle((2*dx,dy), dx, dy, linewidth=2, edgecolor='w', facecolor='none')
im = ax0.pcolormesh(image_ROI_display, cmap = 'jet', shading = 'auto', rasterized=True)
ax0.add_patch(rect_up)
ax0.add_patch(rect_down)
ax0.add_patch(rect_left)
ax0.add_patch(rect_right)
ax0.set(xlabel = 'horizontal pixel (x)', ylabel ='vertical pixel (y)')
ax0.invert_yaxis()
ax0.set_title('Linear scale', fontsize=16)
plt.show()


fig, ax1  = plt.subplots(figsize=(15,4))
rect_up = patches.Rectangle((dx,0), dx, dy, linewidth=2, edgecolor='r', facecolor='none')
rect_down = patches.Rectangle((dx,2*dy), dx, dy, linewidth=2, edgecolor='r', facecolor='none')
rect_left = patches.Rectangle((0,dy), dx, dy, linewidth=2, edgecolor='w', facecolor='none')
rect_right = patches.Rectangle((2*dx,dy), dx, dy, linewidth=2, edgecolor='w', facecolor='none')
im = ax1.pcolormesh(image_ROI_display, norm = colors.LogNorm(), cmap = 'jet', shading = 'auto', rasterized=True)
ax1.add_patch(rect_up)
ax1.add_patch(rect_down)
ax1.add_patch(rect_left)
ax1.add_patch(rect_right)
ax1.set(xlabel = 'horizontal pixel (x)', ylabel ='vertical pixel (y)')
ax1.invert_yaxis()
ax1.set_title('Log scale', fontsize=16)
plt.show()
```

```
Check background ROIs on scan SIRIUS_2021_04_14_4267.nxs
Vertical position beam = 925  
Positions ROIs: up left corner, included, (y, x); bottom right corner, included, (y, x)
Summation ROI = (922, 560); (928, 580)
Up bckg ROI = (915, 560); (921, 580)
Down bckg ROI = (929, 560); (935, 580)
Left bckg ROI = (922, 539); (928, 559)
Right bckg ROI = (922, 581); (928, 601)
```

### Subtract background for all the scans & define errors¶

We extract the background for each scan and subtract it from the intensity of the reflected beam.

We also define the error for each point as the square root of the intensity. We use a worst-case scenario for the error definition: the sum of the error on the reflected beam and of the average error of the background ROIs.

In [11]:

```
I_bckg_up = np.array([])
I_bckg_down = np.array([])
I_bckg_left = np.array([])
I_bckg_right = np.array([])

for i in range(len(file_list)):
    
    file = file_list[i]
    
    nxs_filename = file+'.nxs'

    _, image, _, _, _, _, _, _ = DetectorSum.Extract(nxs_filename, recording_dir,
                                                     show_data_stamps=False, verbose=False)
    
    # Replace the intensity of the dead zones with a value of 0
    image=np.where(image<0., 0., image)
    
    # Vertical position of the beam
    pos_y = int(pos_y_list[i])

    # height of the summation ROI
    dy = summation_ROIsizey

    # width of the summation ROI
    dx = ROIsizex    
        
    # Definitions of bckg ROIs
    bckg_ROI_up = image[pos_y-dy-dy//2 : pos_y-dy+dy//2+1,
                        ROI[0] : ROI[0]+dx]

    bckg_ROI_down = image[pos_y+dy-dy//2 : pos_y+dy+dy//2+1,
                          ROI[0] : ROI[0]+dx]

    bckg_ROI_left = image[pos_y-dy//2 : pos_y+dy//2+1,
                          ROI[0]-dx : ROI[0]]

    bckg_ROI_right = image[pos_y-dy//2 : pos_y+dy//2+1,
                          ROI[0]+dx : ROI[0]+2*dx]

    # Extract info from nexus file
    nexus = PN.PyNexusFile(recording_dir+file+'.nxs', fast=True)
    stamps0D, data0D = nexus.extractData("0D")
    nbpts=int(nexus.get_nbpts())
    sensor_list = [stamps0D[i][0] if stamps0D[i][1]== None else stamps0D[i][1] for i in range(len(stamps0D))]

    integration_timeArg = sensor_list.index('integration_time')
    integration_time = np.mean(data0D[integration_timeArg])

    # Sum the ROI and normalize with the integration time and the number of images
    I_bckg_up = np.append(I_bckg_up, bckg_ROI_up.sum()/integration_time/nbpts)
    I_bckg_down = np.append(I_bckg_down, bckg_ROI_down.sum()/integration_time/nbpts)
    I_bckg_left = np.append(I_bckg_left, bckg_ROI_left.sum()/integration_time/nbpts)
    I_bckg_right = np.append(I_bckg_right, bckg_ROI_right.sum()/integration_time/nbpts)
    
if any([is_bckg_up, is_bckg_down, is_bckg_left, is_bckg_right]):
    # Take the average of the chosen backgrounds ROIs
    print('Background taken: '+is_bckg_up*'up '+is_bckg_down*'down '+is_bckg_left*'left '+is_bckg_right*'right ')
    I_bckg_refl = (is_bckg_up*I_bckg_up+is_bckg_down*I_bckg_down+is_bckg_left*I_bckg_left+is_bckg_right*I_bckg_right)/(is_bckg_up*1.+is_bckg_down*1.+is_bckg_left*1.+is_bckg_right*1.)
    err_I_sub_refl = np.sqrt(I_raw_refl)+(is_bckg_up*np.sqrt(I_bckg_up)+is_bckg_down*np.sqrt(I_bckg_down)+is_bckg_left*np.sqrt(I_bckg_left)+is_bckg_right*np.sqrt(I_bckg_right))/(is_bckg_up*1.+is_bckg_down*1.+is_bckg_left*1.+is_bckg_right*1.)
else:    
    print('No subtraction done.')
    I_bckg_refl = 0.*I_bckg_up    
    err_I_sub_refl = np.sqrt(I_raw_refl)

I_sub_refl = I_raw_refl - I_bckg_refl
```

```
Background taken: up down
```

We plot the background intensity, which looks weird for the same reasons as for the reflected beams. We see that the subtraction is important for large values of theta.

In [12]:

```
fig = plt.figure(figsize=(12,5))
ax=fig.add_subplot(111)
plt.yscale('log')
plt.plot(I_raw_refl, 'b-',  label = 'Reflected beam before background subtraction')
plt.errorbar(np.arange(np.shape(I_sub_refl)[0]), I_sub_refl, err_I_sub_refl,
             fmt = 'k.-', label = 'Reflected beam after background subtraction')
plt.plot(I_bckg_refl, 'r-', label = 'Background')
if is_bckg_up: plt.plot(I_bckg_up, 'g--', label = 'Background up')
if is_bckg_down: plt.plot(I_bckg_down, 'm--', label = 'Background down')
if is_bckg_left: plt.plot(I_bckg_left, 'g-.', label = 'Background left')
if is_bckg_right: plt.plot(I_bckg_right, 'm-.', label = 'Background right')
ax.set_xlabel('Scan index in the list', fontsize=16)
ax.set_ylabel('I', fontsize=16)
plt.legend()
ax.tick_params(labelsize=16)
ax.yaxis.offsetText.set_fontsize(16)
plt.show()
```

# Normalization by the corrected voltage of the ionization chamber¶

For each scan of the XRR, the voltage of the ionization chamber is measured for each of its 6 gains. The values are stored in the companion file `_XRR_gains.dat`.  
An increase of 1 in the gain corresponds to a factor 10 in the measured voltage, with a saturation at 10 V.  
**We assume that the voltage is proportional to the intensity of the incident beam**, provided that the gain is high enough. Thus, the voltage is used to normalize the intensity of the reflected and direct beams.

The following step consists in finding for each point the maximum gain for which the voltage returned by the ionization chamber is not saturated.

## Extraction of the voltage value¶

### Details and control for a single scan¶

We detail the code for one point of the XRR here.

In [13]:

```
# Extract the intensity of the ionization chamber for the different gains

i = 3

file = file_list[i]

file += '_XRR_gains.dat'

# Extraction of the voltage for each gain
gain1 =  np.genfromtxt(recording_dir+file)[1]
gain2 =  np.genfromtxt(recording_dir+file)[2]
gain3 =  np.genfromtxt(recording_dir+file)[3]
gain4 =  np.genfromtxt(recording_dir+file)[4]
gain5 =  np.genfromtxt(recording_dir+file)[5]
gain6 =  np.genfromtxt(recording_dir+file)[6]

gains = [gain1, gain2, gain3, gain4, gain5, gain6]

# Identify saturated values
g1s = gain1 if gain1<9.9 else -1
g2s = gain2 if gain2<9.9 else -1
g3s = gain3 if gain3<9.9 else -1
g4s = gain4 if gain4<9.9 else -1
g5s = gain5 if gain5<9.9 else -1
g6s = gain6 if gain6<9.9 else -1

# Extract the maximum non-saturated voltage value, normalized by the corresponding gain
g_temp = g5s/1e4 if g6s<0 else g6s/1e5
g_temp = g4s/1e3 if g_temp<0 else g_temp
g_temp = g3s/1e2 if g_temp<0 else g_temp
g_temp = g2s/1e1 if g_temp<0 else g_temp
g_temp = g1s if g_temp<0 else g_temp

V_refl = g_temp

print('V = %g for file %s'%(V_refl, file))
```

```
V = 3.77106e-07 for file SIRIUS_2021_04_14_4257_XRR_gains.dat
```

### Extract V for all the scans¶

The procedure is the same as for the example.

In [14]:

```
# Extract the intensity of the ionization chamber for the different gains

gain1 = np.array([])
gain2 = np.array([])
gain3 = np.array([])
gain4 = np.array([])
gain5 = np.array([])
gain6 = np.array([])

for file in file_list:

    file += '_XRR_gains.dat'

    # Extraction of the voltage for each gain
    
    gain1_temp =  np.genfromtxt(recording_dir+file)[1]
    gain2_temp =  np.genfromtxt(recording_dir+file)[2]
    gain3_temp =  np.genfromtxt(recording_dir+file)[3]
    gain4_temp =  np.genfromtxt(recording_dir+file)[4]
    gain5_temp =  np.genfromtxt(recording_dir+file)[5]
    gain6_temp =  np.genfromtxt(recording_dir+file)[6]

    gain1 = np.append(gain1, gain1_temp)
    gain2 = np.append(gain2, gain2_temp)
    gain3 = np.append(gain3, gain3_temp)
    gain4 = np.append(gain4, gain4_temp)
    gain5 = np.append(gain5, gain5_temp)
    gain6 = np.append(gain6, gain6_temp)

gains = [gain1, gain2, gain3, gain4, gain5, gain6]

# Identify saturated values
g1s = np.where(gain1<9.9, gain1, -1) 
g2s = np.where(gain2<9.9, gain2, -1) 
g3s = np.where(gain3<9.9, gain3, -1) 
g4s = np.where(gain4<9.9, gain4, -1) 
g5s = np.where(gain5<9.9, gain5, -1) 
g6s = np.where(gain6<9.9, gain6, -1) 

# Construct the final curve
g_temp = np.where(g6s<0, g5s/1e4, g6s/1e5)
g_temp = np.where(g_temp<0, g4s/1e3, g_temp)
g_temp = np.where(g_temp<0, g3s/1e2, g_temp)
g_temp = np.where(g_temp<0, g2s/1e1, g_temp)
V_refl = np.where(g_temp<0, g1s, g_temp)
```

We plot V, which is non-monotonic because the attenuators are changed in the course of the XRR curve.

In [15]:

```
fig = plt.figure(figsize=(12,5))
ax=fig.add_subplot(111)
plt.plot(V_refl, 'r-')
ax.set_xlabel('Scan index in the list', fontsize=16)
ax.set_ylabel('V', fontsize=16)
ax.tick_params(labelsize=16)
ax.yaxis.offsetText.set_fontsize(16)
plt.show()
```

## Normalize the reflected beam by the voltage of the ionization chamber¶

The intensity of the reflected beam is simply divided by the corresponding voltage of the ionization chamber V, and so are the errors and the background.

We also extract the value of theta, the incident angle on the water surface, for each data point:  
$$\theta = 2 |m4\_{pitch}−m4\_{pitch0}|$$  
$m4\_{pitch}$ is the angle of the deflection mirror, and $m4\_{pitch0}$ the value for which it is parallel to the beam.  
**$m4\_{pitch0}$ is provided by the user in JupyLabBook.**

In [16]:

```
# User-defined value of m4pitch0
m4pitch0 = -0.0375

# Normalize by V
I_sub_over_V_refl = I_sub_refl/V_refl
I_bckg_over_V_refl = I_bckg_refl/V_refl
err_I_sub_over_V_refl = err_I_sub_refl/V_refl

m4pitch = np.array([])

for file in file_list:

    nxs_filename = file+'.nxs'

    # Extract info from nexus file
    nexus = PN.PyNexusFile(recording_dir+nxs_filename, fast=True)
    stamps0D, data0D = nexus.extractData("0D")
    sensor_list = [stamps0D[i][0] if stamps0D[i][1]== None else stamps0D[i][1] for i in range(len(stamps0D))]

    if 'm4pitch' in sensor_list:
        m4pitchArg = sensor_list.index('m4pitch')

    m4pitch = np.append(m4pitch, np.mean(data0D[m4pitchArg]))
    
    # Define theta (in rad)
    theta = 2*np.abs(m4pitch-m4pitch0)*np.pi/180.
```

We plot the normalized intensity as a function of 2\*theta, now this looks like a XRR curve. The last missing thing is the normalization by the direct beam.

In [17]:

```
fig = plt.figure(figsize=(12,5))
ax=fig.add_subplot(111)
plt.yscale('log')
plt.errorbar(2*theta*180./np.pi, I_sub_over_V_refl, err_I_sub_over_V_refl, fmt = 'k.-',
             label = 'Reflected beam after subtraction')
plt.plot(2*theta*180./np.pi, I_bckg_over_V_refl, 'r-', label = 'Background')
plt.plot(2*theta*180./np.pi, I_sub_over_V_refl+I_bckg_over_V_refl,
         'b-', label = 'Reflected beam before subtraction')
ax.set_xlabel('2*theta (deg)', fontsize=16)
ax.set_ylabel('I', fontsize=16)
ax.tick_params(labelsize=16)
plt.legend()
ax.yaxis.offsetText.set_fontsize(16)
plt.show()
```

# Normalization by the direct beam¶

Every steps made above should be performed as well on the direct beam.

## Control the direct¶

We check that the direct is in the Pilatus.

In [18]:

```
# Here we focus on the direct scan only
nxs_filename = 'SIRIUS_2021_04_14_4253.nxs'

# Extract the sum of all images in the time scan
_, image_direct, _, _, _, _, _, _ = DetectorSum.Extract(nxs_filename, recording_dir,
                                                        show_data_stamps=False, verbose=False)

# Replace the intensity of the dead zones with a value of 0
image_direct = np.where(image_direct<0., 0., image_direct)

# Show the ROI image (summed over the time scan)
fig, ax0  = plt.subplots(figsize=(15,4))
im = ax0.pcolormesh(image_direct, cmap = 'jet', norm = colors.LogNorm(), shading = 'auto', rasterized=True)
fig.colorbar(im, ax=ax0)
ax0.set(xlabel = 'horizontal pixel (x)', ylabel ='vertical pixel (y)')
ax0.invert_yaxis()
plt.show()
```

```

```

## Finding the position of the direct beam¶

The direct beam is easily found by taking the position of maximum on the whole detector.

In [19]:

```
# Integrate along the horizontal axis and find the maximum
integrated_x_direct = image_direct.sum(axis=1)

pos_y_direct = np.argmax(integrated_x_direct)

# height of the summation ROI
# Always use an odd height for the summation_ROI!
dy = summation_ROIsizey

print('Position maximum = %s'%pos_y_direct)

summation_ROI_direct = image_direct[pos_y_direct-dy//2 : pos_y_direct+dy//2+1,
                                    ROI[0] : ROI[0]+ROI[2]]

print('Top left corner of summation ROI = %s, %s'%(pos_y_direct-dy//2,
                                                   pos_y_direct+dy//2))

fig, ax0  = plt.subplots(figsize=(15,4))
im = ax0.pcolormesh(summation_ROI_direct, cmap = 'jet', shading = 'auto', rasterized=True)
fig.colorbar(im, ax=ax0)
ax0.set(xlabel = 'horizontal pixel (x)', ylabel ='vertical pixel (y)')
ax0.invert_yaxis()
plt.show()
```

```
Position maximum = 939
Top left corner of summation ROI = 936, 942
```

## Extract the raw intensity of the direct beam¶

We sum over the summation ROI and normalize by the integration time and the number of points in the scan.

In [20]:

```
# Extract info from nexus file
nexus = PN.PyNexusFile(recording_dir+nxs_filename, fast=True)
stamps0D, data0D = nexus.extractData("0D")
nbpts=int(nexus.get_nbpts())
sensor_list = [stamps0D[i][0] if stamps0D[i][1]== None else stamps0D[i][1] for i in range(len(stamps0D))]

integration_timeArg = sensor_list.index('integration_time')
integration_time = np.mean(data0D[integration_timeArg])

# Sum the ROI and normalize with the integration time and the number of images
I_raw_direct = summation_ROI_direct.sum()/integration_time/nbpts

print('Raw value of the direct beam (before subtraction & normalization) = %g'%I_raw_direct)
```

```
Raw value of the direct beam (before subtraction & normalization) = 884100
```

## Background subtraction¶

The background is defined the same way as for the reflected beam.

In [21]:

```
summation_ROI_direct = image_direct[pos_y_direct-dy//2 : pos_y_direct+dy//2+1,
                                    ROI[0] : ROI[0]+dx]
print('Summation ROI = (%s, %s); (%s, %s)'%(pos_y_direct-dy//2,ROI[0],pos_y_direct+dy//2,ROI[0]+dx-1))

bckg_ROI_up_direct = image_direct[pos_y_direct-dy-dy//2 : pos_y_direct-dy+dy//2+1,
                                  ROI[0] : ROI[0]+dx]
print('Up bckg ROI = (%s, %s); (%s, %s)'%(pos_y_direct-dy-dy//2,ROI[0],pos_y_direct-dy+dy//2,ROI[0]+dx-1))


bckg_ROI_down_direct = image_direct[pos_y_direct+dy-dy//2 : pos_y_direct+dy+dy//2+1,
                                    ROI[0] : ROI[0]+dx]

print('Down bckg ROI = (%s, %s); (%s, %s)'%(pos_y_direct+dy-dy//2,ROI[0],pos_y_direct+dy+dy//2,ROI[0]+dx-1))

bckg_ROI_left_direct = image_direct[pos_y_direct-dy//2 : pos_y_direct+dy//2+1,
                                    ROI[0]-dx : ROI[0]]
print('Left bckg ROI = (%s, %s); (%s, %s)'%(pos_y_direct-dy//2,ROI[0]-dx,pos_y_direct+dy//2,ROI[0]-1))

bckg_ROI_right_direct = image_direct[pos_y_direct-dy//2 : pos_y_direct+dy//2+1,
                                     ROI[0]+dx : ROI[0]+2*dx]
print('Right bckg ROI = (%s, %s); (%s, %s)'%(pos_y_direct-dy//2,ROI[0]+dx,pos_y_direct+dy//2,ROI[0]+2*dx-1))

# Draw a larger ROI for display of the background
image_ROI_display_direct = image_direct[pos_y_direct-dy-dy//2 : pos_y_direct+dy+dy//2+1,
                                        ROI[0]-dx : ROI[0]+2*dx]    

 
I_bckg_up_direct = bckg_ROI_up_direct.sum()/integration_time/nbpts
I_bckg_down_direct = bckg_ROI_down_direct.sum()/integration_time/nbpts
I_bckg_left_direct = bckg_ROI_left_direct.sum()/integration_time/nbpts
I_bckg_right_direct = bckg_ROI_right_direct.sum()/integration_time/nbpts

    
if any([is_bckg_up, is_bckg_down, is_bckg_left, is_bckg_right]):
    # Take the average of the chosen backgrounds ROIs
    print('Background taken: '+is_bckg_up*'up '+is_bckg_down*'down '+is_bckg_left*'left '+is_bckg_right*'right ')
    I_bckg_direct = (is_bckg_up*I_bckg_up_direct+is_bckg_down*I_bckg_down_direct+is_bckg_left*I_bckg_left_direct+is_bckg_right*I_bckg_right_direct)/(is_bckg_up*1.+is_bckg_down*1.+is_bckg_left*1.+is_bckg_right*1.)
else:    
    print('No subtraction done.')
    I_bckg_direct = 0.
   
I_sub_direct = I_raw_direct - I_bckg_direct

print('Value of the upper background = %g'%I_bckg_up_direct)
print('Value of the lower background = %g'%I_bckg_down_direct)
print('Value of the right background = %g'%I_bckg_left_direct)
print('Value of the left background = %g'%I_bckg_right_direct)
print('Value of the chosen background = %g'%I_bckg_direct)
print('Value of the direct beam after subtraction of the background = %g'%I_sub_direct)

fig, ax0  = plt.subplots(figsize=(15,4))

rect_up = patches.Rectangle((dx,0), dx, dy, linewidth=2, edgecolor='r', facecolor='none')
rect_down = patches.Rectangle((dx,2*dy), dx, dy, linewidth=2, edgecolor='r', facecolor='none')
rect_left = patches.Rectangle((0,dy), dx, dy, linewidth=2, edgecolor='w', facecolor='none')
rect_right = patches.Rectangle((2*dx,dy), dx, dy, linewidth=2, edgecolor='w', facecolor='none')
im = ax0.pcolormesh(image_ROI_display_direct, cmap = 'jet', shading = 'auto', rasterized=True)
ax0.add_patch(rect_up)
ax0.add_patch(rect_down)
ax0.add_patch(rect_left)
ax0.add_patch(rect_right)
ax0.set(xlabel = 'horizontal pixel (x)', ylabel ='vertical pixel (y)')
ax0.invert_yaxis()
ax0.set_title('Linear scale', fontsize=16)
plt.show()


fig, ax1  = plt.subplots(figsize=(15,4))
rect_up = patches.Rectangle((dx,0), dx, dy, linewidth=2, edgecolor='r', facecolor='none')
rect_down = patches.Rectangle((dx,2*dy), dx, dy, linewidth=2, edgecolor='r', facecolor='none')
rect_left = patches.Rectangle((0,dy), dx, dy, linewidth=2, edgecolor='w', facecolor='none')
rect_right = patches.Rectangle((2*dx,dy), dx, dy, linewidth=2, edgecolor='w', facecolor='none')
im = ax1.pcolormesh(image_ROI_display_direct, norm = colors.LogNorm(), cmap = 'jet', shading = 'auto', rasterized=True)
ax1.add_patch(rect_up)
ax1.add_patch(rect_down)
ax1.add_patch(rect_left)
ax1.add_patch(rect_right)
ax1.set(xlabel = 'horizontal pixel (x)', ylabel ='vertical pixel (y)')
ax1.invert_yaxis()
ax1.set_title('Log scale', fontsize=16)
plt.show()
```

```
Summation ROI = (936, 560); (942, 580)
Up bckg ROI = (929, 560); (935, 580)
Down bckg ROI = (943, 560); (949, 580)
Left bckg ROI = (936, 539); (942, 559)
Right bckg ROI = (936, 581); (942, 601)
Background taken: up down 
Value of the upper background = 1499.91
Value of the lower background = 1175.91
Value of the right background = 146.455
Value of the left background = 121
Value of the chosen background = 1337.91
Value of the direct beam after subtraction of the background = 882762
```

## Normalization by the incident beam¶

V is defined the same way as for the reflected beam, except that there is no m4pitch value recorded in the companion file `_direct_gains.dat`.

In [22]:

```
# Extract the intensity of the ionization chamber for the different gains

file = nxs_filename[:-4]

file += '_direct_gains.dat'

# Extraction of the voltage for each gain
gain1 =  np.genfromtxt(recording_dir+file)[0]
gain2 =  np.genfromtxt(recording_dir+file)[1]
gain3 =  np.genfromtxt(recording_dir+file)[2]
gain4 =  np.genfromtxt(recording_dir+file)[3]
gain5 =  np.genfromtxt(recording_dir+file)[4]
gain6 =  np.genfromtxt(recording_dir+file)[5]

gains = [gain1, gain2, gain3, gain4, gain5, gain6]

# Identify saturated values
g1s = gain1 if gain1<9.9 else -1
g2s = gain2 if gain2<9.9 else -1
g3s = gain3 if gain3<9.9 else -1
g4s = gain4 if gain4<9.9 else -1
g5s = gain5 if gain5<9.9 else -1
g6s = gain6 if gain6<9.9 else -1

# Extract the maximum non-saturated voltage value, normalized by its corresponding gain
g_temp = g5s/1e4 if g6s<0 else g6s/1e5
g_temp = g4s/1e3 if g_temp<0 else g_temp
g_temp = g3s/1e2 if g_temp<0 else g_temp
g_temp = g2s/1e1 if g_temp<0 else g_temp
g_temp = g1s if g_temp<0 else g_temp

V_direct = g_temp

print('V = %g for the direct beam.'%(V_direct))

I_sub_over_V_direct = I_sub_direct/V_direct

print('Intensity of the direct beam = %g after background subtraction & normalization by V.'%(I_sub_over_V_direct))
```

```
V = 3.65021e-07 for the direct beam.
Intensity of the direct beam = 2.41839e+12 after background subtraction & normalization by V.
```

# Normalization by the direct beam : XRR curve¶

Finally, we normalize the intensity of the reflected beam with the direct beam and obtain the XRR curve.

In [23]:

```
R = I_sub_over_V_refl/I_sub_over_V_direct
err_R = err_I_sub_over_V_refl/I_sub_over_V_direct
bckg_R = I_bckg_over_V_refl/I_sub_over_V_direct
```

We plot the results for $q\_z= 4\pi/\lambda\sin(\theta)$ as well, where **$\lambda$ is the wavelength provided by the user in JupyLabBook**, and for $Iq\_z^4$ to normalize by the Fresnel reflectivity.

In [24]:

```
# lambda in nm
wavelength = 0.155
qz = 4*np.pi/wavelength*np.sin(theta)

fig = plt.figure(figsize=(12,5))
ax=fig.add_subplot(111)
plt.yscale('log')
plt.errorbar(2*theta*180./np.pi, R, err_R, fmt = 'k.-', label = 'XRR after subtraction')
plt.plot(2*theta*180./np.pi, R+bckg_R, 'b-', label = 'XRR before subtraction')
plt.plot(2*theta*180./np.pi, bckg_R, 'r-', label = 'Background')
plt.legend()
ax.set_xlabel('2*theta (deg)', fontsize=16)
ax.set_ylabel('R', fontsize=16)
ax.tick_params(labelsize=16)
ax.yaxis.offsetText.set_fontsize(16)
plt.show()

fig = plt.figure(figsize=(12,5))
ax=fig.add_subplot(111)
plt.yscale('log')
plt.errorbar(qz, R, err_R, fmt = 'k.-')
ax.set_xlabel('qz (nm-1)', fontsize=16)
ax.set_ylabel('R', fontsize=16)
ax.tick_params(labelsize=16)
ax.yaxis.offsetText.set_fontsize(16)
plt.show()

fig = plt.figure(figsize=(12,5))
ax=fig.add_subplot(111)
plt.yscale('log')
plt.errorbar(qz, R*qz**4, err_R*qz**4, fmt = 'k.-')
ax.set_xlabel('qz (nm-1)', fontsize=16)
ax.set_ylabel('R*qz^4 (nm-4)', fontsize=16)
ax.tick_params(labelsize=16)
ax.yaxis.offsetText.set_fontsize(16)
plt.show()
```

# Summary of the useful variables¶

- `I_raw_refl` & `I_raw_direct`: the intensity obtained by summing all the pixels in the summation ROI, which is centered on the reflected/direct beams.
- `I_bckg_refl` & `I_bckg_direct`: the intensity obtained by summing and averaging the background ROIs chosen by the user.
- `I_sub_refl` & `I_sub_direct`: `I_raw`-`I_bckg`.
- `V_refl` & `V_direct`: voltage of the ionization chamber normalized by its gain, proportional to the intensity of the incident beam.
- `R`: the reflectivity, with all the proper normalizations done.
- `err_R`: the error bars on the reflectivity.

# Summary of the parameters¶

In [25]:

```
print('XRR file: %s'%original_nxs_filename)
print('Full scan ROI (ROIx0, ROIy0, ROIsizex, ROIsizey): (%g,%g,%g,%g)'%(ROIx0, ROIy0, ROIsizex, ROIsizey))
print('Vertical size of the summation ROI: %g'%summation_ROIsizey)
print('Background taken: '+is_bckg_up*'up '+is_bckg_down*'down '+is_bckg_left*'left '+is_bckg_right*'right ')
print('m4pitch0: %g'%m4pitch0)
print('wavelength: %g'%wavelength)
```

```
XRR file: SIRIUS_2021_04_14_4254.nxs
Full scan ROI (ROIx0, ROIy0, ROIsizex, ROIsizey): (560,905,21,40)
Vertical size of the summation ROI: 7
Background taken: up down 
m4pitch0: -0.0375
wavelength: 0.155
```
