# Supplementary material for: Opportunities and new developments for the study of surfaces and interfaces in soft condensed matter at the SIRIUS beamline of Synchrotron SOLEIL
Source: J Synchrotron Radiat. 2024 Jan 1;31(Pt 1):162–76. doi: 10.1107/S1600577523008810 (PMC10833424; doi:10.1107/S1600577523008810)
Supplement: Supplementary file 1 [file s-31-00162-sup1.zip › JupyLabBook-v3.0.2/docs/XRR_solid/Howto_batch_XRR_solid.html]

Howto\_batch\_XRR\_solid


# Batch XRR solid¶

We show here how to batch a series of XRR using the library `XRR_solid.py` (JupyLabBook >= v2.10.5).

Each step is described in details in the notebook `Howto_data_reduction_XRR_solid.ipynb`.

**Careful, in this work (Rosilio2022), a factor has been introduced on gain 8 in XRR\_solid.py to account for a non-linearity of the ionization chamber.**

## User inputs¶

In [1]:

```
# Put the path to the folder containing /lib
# Here we start from JupyLabBook/docs/XRR/ and we want to go back to JupyLabBook/
import os
import numpy as np
os.chdir("../../")

# Import the library
from lib.extraction import XRR_solid as XRR_solid

# Directory where the nexus files are
recording_dir = '/home/experiences/sirius/hemmerle/ruche/sirius-soleil/com-sirius/users/2022/rosilio/'

# Directory where the data will be saved (the directory must exist)
working_dir = 'working/'

# List of XRR to treat (list of list)
base_name = 'SIRIUS_2022_02_23_'
list_XRR = [
['4066', '4067', '4068', '4069', '4070', '4073'],
['4081', '4082', '4083', '4084', '4085', '4087'],
['4096', '4097', '4098', '4099', '4101', '4103'],
['4111', '4112', '4113', '4114', '4116'],
]

# List of corresponding direct scans
list_direct = [
'4065',
'4080',
'4095',
'4110',
]

# User defined summation ROI
# Always use an odd number for ROIsizey!
ROIx0=566
ROIy0=947
ROIsizex=14
ROIsizey=5

# lambda (in nm)
wavelength = 0.12398

# Define ROIs for background
is_bckg_up = True
is_bckg_down = True
is_bckg_left = False
is_bckg_right = False
```

## Loop over the files¶

In [2]:

```
for i, nxs_filenames in enumerate(list_XRR):
    nxs_filenames = [base_name + nxs_filename + '.nxs' for nxs_filename in nxs_filenames]
    direct_nxs_filename = base_name + list_direct[i] + '.nxs'
       
    print('Treating XRR: %s-%s'%(nxs_filenames[0],nxs_filenames[-1]))

    XRR_solid.Treat(nxs_filenames, recording_dir, direct_nxs_filename,
          ROIx0, ROIy0, ROIsizex, ROIsizey,
          wavelength, force_direct=False,  fdirect=1.,
          is_bckg_up=True, is_bckg_down=True, is_bckg_left=False, is_bckg_right=False,
          working_dir=working_dir, plot_XRR_gamma=False, plot_XRR_qz=False,
          save=True, verbose=False)

    print('')
```

```
Treating XRR: SIRIUS_2022_02_23_4066.nxs-SIRIUS_2022_02_23_4073.nxs
Background taken: up down     
Direct extracted from SIRIUS_2022_02_23_4065.nxs: direct=6.07374e+13
                              
Treating XRR: SIRIUS_2022_02_23_4081.nxs-SIRIUS_2022_02_23_4087.nxs
Background taken: up down     
Direct extracted from SIRIUS_2022_02_23_4080.nxs: direct=5.82397e+13
                              
Treating XRR: SIRIUS_2022_02_23_4096.nxs-SIRIUS_2022_02_23_4103.nxs
Background taken: up down     
Direct extracted from SIRIUS_2022_02_23_4095.nxs: direct=5.80651e+13
                              
Treating XRR: SIRIUS_2022_02_23_4111.nxs-SIRIUS_2022_02_23_4116.nxs
Background taken: up down     
Direct extracted from SIRIUS_2022_02_23_4110.nxs: direct=5.91508e+13
```

## Plot a specific result¶

In [3]:

```
import numpy as np

for nxs_filenames in list_XRR:

    nxs_filenames = [base_name + nxs_filename + '.nxs' for nxs_filename in nxs_filenames]
    
    first_nxs = nxs_filenames[0][:nxs_filenames[0].rfind('.nxs')]
    last_nxs = nxs_filenames[-1][:nxs_filenames[-1].rfind('.nxs')]

    savename=working_dir+first_nxs.split('.nxs')[0]+\
            '-'+last_nxs.split('.nxs')[0].split('_')[-1]
    
    data_filename = savename+'_XRR.dat'
    
    [gamma, qz, bckg_R_up, bckg_R_down, bckg_R_left, bckg_R_right, bckg_R, err_R, R] =\
    np.loadtxt(data_filename, delimiter = '\t').transpose()

    XRR_solid.Plot(gamma, qz, bckg_R_up, bckg_R_down, bckg_R_left, bckg_R_right, bckg_R,\
                   err_R, R, nxs_filenames, plot_XRR_gamma=True, plot_XRR_qz=True)
```

In [ ]:

```

```
