# Supplementary material for: Opportunities and new developments for the study of surfaces and interfaces in soft condensed matter at the SIRIUS beamline of Synchrotron SOLEIL
Source: J Synchrotron Radiat. 2024 Jan 1;31(Pt 1):162–76. doi: 10.1107/S1600577523008810 (PMC10833424; doi:10.1107/S1600577523008810)
Supplement: Supplementary file 1 [file s-31-00162-sup1.zip › JupyLabBook-v3.0.2/docs/XRR_solid/Howto_data_reduction_XRR_solid.html]

Howto\_data\_reduction\_XRR\_solid


# Description of the data reduction process for XRR on a solid surface¶

This notebook explains the different steps performed to extract the X-ray reflectivity in JupyLabBook >= v2.10.5 with the library `XRR_solid.py`.

# Conditions¶

The conditions for the example treated here are:

- A 2D detector (Pilatus or UFXC) is on the delta-gamma arm.
- A height scan or a time scan of the direct beam has been performed.
- The ionization chamber has been mounted before the sample.

# Extraction of the intensity of the reflected beam¶

We describe here how the raw intensity of the reflected beam is extracted.

## User-defined summation ROI¶

The user defines the summation ROI, **which will be summed to extract the intensity of the reflected beam**. Careful, the larger the ROI, the more background you add to the signal. **Also, the height has to be an odd number, to have the beam centered.**

In [1]:

```
# User defined summation ROI
# Always use an odd number for ROIsizey!
ROIx0=566
ROIy0=947
ROIsizex=14
ROIsizey=5
```

### Control on one data point¶

We check that the ROI is right for one scan of the XRR curve.  
We use the library `DetectorSum.py` to extract the 2D images from the nexus file. All the images within a same scan are summed (i.e. summation over all the angles in the scan).

In [2]:

```
# Put the path to the folder containing /lib
# Here we start from JupyLabBook/docs/XRR/ and we want to go back to JupyLabBook/
import os
os.chdir("../../")

from lib.extraction import DetectorSum as DetectorSum
import numpy as np
import matplotlib.pyplot as plt
import matplotlib.colors as colors

recording_dir = 'not_in_git/recording/'
nxs_filename = 'SIRIUS_2022_02_23_4082.nxs'


# Extract the sum of all images in the scan
_, image, _, _, _, _, _, _  =DetectorSum.Extract(nxs_filename, recording_dir,
                                                 show_data_stamps=False, verbose=False)

# Replace the intensity of the dead zones with a value of 0
image=np.where(image<0., 0., image)


# Extract the summation ROI
# Full image: ROI = [0, 0, 981, 1043]
# Here: ROI = [ROIx0, ROIy0, ROIsizex, ROIsizey]

#Apply the ROI
summation_ROI = image[ROIy0:ROIy0+ROIsizey, ROIx0:ROIx0+ROIsizex]

print('Summation ROI = (%s, %s); (%s, %s)'%(ROIy0, ROIx0, ROIy0+ROIsizey-1, ROIx0+ROIsizex-1))

fig, ax0  = plt.subplots(figsize=(15,4))
im = ax0.pcolormesh(summation_ROI, cmap = 'jet', shading = 'auto', rasterized=True)
fig.colorbar(im, ax=ax0)
ax0.set_title('Lin scale', fontsize=16)
ax0.set(xlabel = 'horizontal pixel (x)', ylabel ='vertical pixel (y)')
ax0.invert_yaxis()
plt.show()

fig, ax0  = plt.subplots(figsize=(15,4))
im = ax0.pcolormesh(summation_ROI, norm = colors.LogNorm(), cmap = 'jet', shading = 'auto', rasterized=True)
fig.colorbar(im, ax=ax0)
ax0.set_title('Log scale', fontsize=16)
ax0.set(xlabel = 'horizontal pixel (x)', ylabel ='vertical pixel (y)')
ax0.invert_yaxis()
plt.show()
```

```
Summation ROI = (947, 566); (951, 579)
```

### Control on all the scans¶

We check on every scan of the XRR that the reflected beam falls in the summation ROI.

In [3]:

```
nxs_filenames = \
['SIRIUS_2022_02_23_4081.nxs',
 'SIRIUS_2022_02_23_4082.nxs',
 'SIRIUS_2022_02_23_4083.nxs',
 'SIRIUS_2022_02_23_4084.nxs',
 'SIRIUS_2022_02_23_4085.nxs',
 'SIRIUS_2022_02_23_4087.nxs',
]

for nxs_filename in nxs_filenames:
    
    print(nxs_filename)
    
    _, image, _, _, _, _, _, _  =DetectorSum.Extract(nxs_filename, recording_dir,
                                                     show_data_stamps=False, verbose=False)
  
    # Replace the intensity of the dead zones with a value of 0
    image=np.where(image<0., 0., image)

    # Apply the ROI
    summation_ROI = image[ROIy0:ROIy0+ROIsizey, ROIx0:ROIx0+ROIsizex]

    # Show the full image integrated over the scan
    fig, ax0  = plt.subplots(figsize=(15,3))
    #fig, ax0  = plt.subplots(figsize=(15,15))
    im = ax0.pcolormesh(summation_ROI, norm = colors.LogNorm(),
                        cmap = 'jet', shading = 'auto', rasterized=True)

    ax0.invert_yaxis()
    plt.show()
```

```
SIRIUS_2022_02_23_4081.nxs
```

```
SIRIUS_2022_02_23_4082.nxs
```

```
SIRIUS_2022_02_23_4083.nxs
```

```
SIRIUS_2022_02_23_4084.nxs
```

```
SIRIUS_2022_02_23_4085.nxs
```

```
SIRIUS_2022_02_23_4087.nxs
```

### Extract the raw intensity of the reflected beam¶

For each scan of the XRR we then extract the raw intensity of the reflected beam by summing over the summation ROI.  
We normalize each point by the integration time.

In [4]:

```
from lib.extraction.common import PyNexus as PN

I_raw_refl = np.array([])
gamma = np.array([])

for nxs_filename in nxs_filenames:    

    # Extract info from nexus file
    nexus = PN.PyNexusFile(recording_dir+nxs_filename, fast=True)
    stamps0D, data0D = nexus.extractData("0D")
    sensor_list = [stamps0D[i][0] if stamps0D[i][1]== None else stamps0D[i][1] for i in range(len(stamps0D))]
    
    # Get the integration time
    integration_timeArg = sensor_list.index('integration_time')
    integration_time = np.mean(data0D[integration_timeArg])

    # Get the two-theta angle
    if 'gamma' in sensor_list:
        gammaArg = sensor_list.index('gamma')

    gamma = np.append(gamma, data0D[gammaArg])
    
    # Extract individual images
    images, _, _, _, _, _, _, _  =DetectorSum.Extract(nxs_filename, recording_dir,
                                                      show_data_stamps=False, verbose=False)
 
    for image in images:

        # Replace the intensity of the dead zones with a value of 0
        image = np.where(image<0., 0., image)

        # Apply the full scan ROI
        summation_ROI = image[ROIy0:ROIy0+ROIsizey, ROIx0:ROIx0+ROIsizex]
  
        # Sum the ROI and normalize with the integration time
        I_raw_refl = np.append(I_raw_refl,summation_ROI.sum(axis=0).sum(axis=0)/integration_time)
```

```

```

We plot the extracted raw intensities, which looks weird because the attenuators were adjusted from one scan to another to avoid beam damage on the detector. This will be adjusted with the normalization by the incident beam.

In [5]:

```
fig = plt.figure(figsize=(12,5))
ax=fig.add_subplot(111)
plt.yscale('log')
plt.plot(gamma, I_raw_refl, 'x-k')
ax.set_xlabel('2*theta (deg)', fontsize=16)
ax.set_ylabel('I_raw_refl', fontsize=16)
fig.subplots_adjust(top=0.9)
ax.tick_params(labelsize=16)
ax.yaxis.offsetText.set_fontsize(16)
plt.show()
```

# Background subtraction¶

The background is defined by taking a ROI of the same size as the summation ROI, eather immediately above and below it, or on its left and right.  
**The user chooses which background is taken:**

- up & down, the most general case.
- up or down, e.g. in case of strong asymmetry or trace of the direct beam.
- left & right, e.g. if there is a strong off-specular scattering.

In [6]:

```
# Choose which background to take
is_bckg_up = True
is_bckg_down = True
is_bckg_left = False
is_bckg_right = False
```

### Control on one scan¶

We check the background ROIs in linear and log scales.

In [7]:

```
import matplotlib.patches as patches

i = 1

nxs_filename = nxs_filenames[i]

print('Check background ROIs on scan %s (integrated)'%nxs_filename)


# Extract the sum of all images in the time scan
_, image, _, _, _, _, _, _  =DetectorSum.Extract(nxs_filename, recording_dir,
                                                 show_data_stamps=False, verbose=False)

# Replace the intensity of the dead zones with a value of 0
image=np.where(image<0., 0., image)


# Extract the summation ROI
# Full image: ROI = [0, 0, 981, 1043]
# Here: ROI = [ROIx0, ROIy0, ROIsizex, ROIsizey]

#Apply the ROI
summation_ROI = image[ROIy0 : ROIy0+ROIsizey,
                      ROIx0 : ROIx0+ROIsizex]

print('Summation ROI = (%s, %s); (%s, %s)'%(ROIy0, ROIx0, ROIy0+ROIsizey-1, ROIx0+ROIsizex-1))

# Up & down background ROIs
bckg_ROI_up = image[ROIy0-ROIsizey : ROIy0,
                    ROIx0 : ROIx0+ROIsizex]
print('Up bckg ROI = (%s, %s); (%s, %s)'%(ROIy0-ROIsizey, ROIx0,
                                          ROIy0-1, ROIx0+ROIsizex-1))


bckg_ROI_down = image[ROIy0+ROIsizey : ROIy0+2*ROIsizey,
                      ROIx0 : ROIx0+ROIsizex]
print('Down bckg ROI = (%s, %s); (%s, %s)'%(ROIy0+ROIsizey, ROIx0,
                                            ROIy0+2*ROIsizey-1, ROIx0+ROIsizex-1))

# Left & right background ROIs
bckg_ROI_left = image[ROIy0 : ROIy0+ROIsizey,
                      ROIx0-ROIsizex : ROIx0]
print('Left bckg ROI = (%s, %s); (%s, %s)'%(ROIy0, ROIx0-ROIsizex,
                                            ROIy0+ROIsizey-1,ROIx0-1))

bckg_ROI_right = image[ROIy0 : ROIy0+ROIsizey,
                       ROIx0+ROIsizex : ROIx0+2*ROIsizex]
print('Right bckg ROI = (%s, %s); (%s, %s)'%(ROIy0,ROIx0+ROIsizex,
                                             ROIy0+ROIsizey-1,ROIx0+2*ROIsizex-1))

# Draw a larger ROI for display of the background
image_ROI_display = image[ROIy0-ROIsizey : ROIy0+2*ROIsizey,
                          ROIx0-ROIsizex : ROIx0+2*ROIsizex]    


fig, ax0  = plt.subplots(figsize=(15,4))

rect_up = patches.Rectangle((ROIsizex,0), ROIsizex, ROIsizey, linewidth=2, edgecolor='r', facecolor='none')
rect_down = patches.Rectangle((ROIsizex,2*ROIsizey), ROIsizex, ROIsizey, linewidth=2, edgecolor='r', facecolor='none')
rect_left = patches.Rectangle((0,ROIsizey), ROIsizex, ROIsizey, linewidth=2, edgecolor='w', facecolor='none')
rect_right = patches.Rectangle((2*ROIsizex,ROIsizey), ROIsizex, ROIsizey, linewidth=2, edgecolor='w', facecolor='none')
im = ax0.pcolormesh(image_ROI_display, cmap = 'jet', shading = 'auto', rasterized=True)
ax0.add_patch(rect_up)
ax0.add_patch(rect_down)
ax0.add_patch(rect_left)
ax0.add_patch(rect_right)
ax0.set(xlabel = 'horizontal pixel (x)', ylabel ='vertical pixel (y)')
ax0.invert_yaxis()
ax0.set_title('Linear scale', fontsize=16)
plt.show()


fig, ax1  = plt.subplots(figsize=(15,4))
rect_up = patches.Rectangle((ROIsizex,0), ROIsizex, ROIsizey, linewidth=2, edgecolor='r', facecolor='none')
rect_down = patches.Rectangle((ROIsizex,2*ROIsizey), ROIsizex, ROIsizey, linewidth=2, edgecolor='r', facecolor='none')
rect_left = patches.Rectangle((0,ROIsizey), ROIsizex, ROIsizey, linewidth=2, edgecolor='w', facecolor='none')
rect_right = patches.Rectangle((2*ROIsizex,ROIsizey), ROIsizex, ROIsizey, linewidth=2, edgecolor='w', facecolor='none')
im = ax1.pcolormesh(image_ROI_display, norm = colors.LogNorm(), cmap = 'jet', shading = 'auto', rasterized=True)
ax1.add_patch(rect_up)
ax1.add_patch(rect_down)
ax1.add_patch(rect_left)
ax1.add_patch(rect_right)
ax1.set(xlabel = 'horizontal pixel (x)', ylabel ='vertical pixel (y)')
ax1.invert_yaxis()
ax1.set_title('Log scale', fontsize=16)
plt.show()
```

```
Check background ROIs on scan SIRIUS_2022_02_23_4082.nxs (integrated)
Summation ROI = (947, 566); (951, 579)
Up bckg ROI = (942, 566); (946, 579)
Down bckg ROI = (952, 566); (956, 579)
Left bckg ROI = (947, 552); (951, 565)
Right bckg ROI = (947, 580); (951, 593)
```

### Subtract background for all the points & define errors¶

We extract the background for each scan and subtract it from the intensity of the reflected beam.

We also define the error for each point as the square root of the intensity. We use a worst-case scenario for the error definition: the sum of the error on the reflected beam and of the average error of the background ROIs.

In [8]:

```
I_bckg_up = np.array([])
I_bckg_down = np.array([])
I_bckg_left = np.array([])
I_bckg_right = np.array([])

for nxs_filename in nxs_filenames:  
   
    # Extract individual images
    images, _, _, _, _, _, _, _  =DetectorSum.Extract(nxs_filename, recording_dir,
                                                      show_data_stamps=False, verbose=False)
 
    # Extract info from nexus file
    nexus = PN.PyNexusFile(recording_dir+nxs_filename, fast=True)
    stamps0D, data0D = nexus.extractData("0D")
    sensor_list = [stamps0D[i][0] if stamps0D[i][1]== None else stamps0D[i][1] for i in range(len(stamps0D))]
    
    # Get the integration time
    integration_timeArg = sensor_list.index('integration_time')
    integration_time = np.mean(data0D[integration_timeArg])

    for image in images:

        # Replace the intensity of the dead zones with a value of 0
        image = np.where(image<0., 0., image)

        bckg_ROI_up = image[ROIy0-ROIsizey : ROIy0,
                            ROIx0 : ROIx0+ROIsizex]

        bckg_ROI_down = image[ROIy0+ROIsizey : ROIy0+2*ROIsizey,
                              ROIx0 : ROIx0+ROIsizex]

        bckg_ROI_left = image[ROIy0 : ROIy0+ROIsizey,
                              ROIx0-ROIsizex : ROIx0]
        
        bckg_ROI_right = image[ROIy0 : ROIy0+ROIsizey,
                               ROIx0+ROIsizex : ROIx0+2*ROIsizex]

        # Sum the ROI and normalize with the integration time
        I_bckg_up = np.append(I_bckg_up, bckg_ROI_up.sum()/integration_time)
        I_bckg_down = np.append(I_bckg_down, bckg_ROI_down.sum()/integration_time)
        I_bckg_left = np.append(I_bckg_left, bckg_ROI_left.sum()/integration_time)
        I_bckg_right = np.append(I_bckg_right, bckg_ROI_right.sum()/integration_time)

if any([is_bckg_up, is_bckg_down, is_bckg_left, is_bckg_right]):
    # Take the average of the chosen backgrounds ROIs
    print('Background taken: '+is_bckg_up*'up '+is_bckg_down*'down '+is_bckg_left*'left '+is_bckg_right*'right ')
    I_bckg_refl = (is_bckg_up*I_bckg_up+is_bckg_down*I_bckg_down+is_bckg_left*I_bckg_left+is_bckg_right*I_bckg_right)/(is_bckg_up*1.+is_bckg_down*1.+is_bckg_left*1.+is_bckg_right*1.)
    err_I_sub_refl = np.sqrt(I_raw_refl)+(is_bckg_up*np.sqrt(I_bckg_up)+is_bckg_down*np.sqrt(I_bckg_down)+is_bckg_left*np.sqrt(I_bckg_left)+is_bckg_right*np.sqrt(I_bckg_right))/(is_bckg_up*1.+is_bckg_down*1.+is_bckg_left*1.+is_bckg_right*1.)
else:    
    print('No subtraction done.')
    I_bckg_refl = 0.*I_bckg_up    
    err_I_sub_refl = np.sqrt(I_raw_refl)

I_sub_refl = I_raw_refl - I_bckg_refl
```

```
Background taken: up down
```

We plot the background intensity, which looks weird for the same reasons as for the reflected beams. We see that the subtraction is important for large values of theta.

In [9]:

```
fig = plt.figure(figsize=(12,5))
ax=fig.add_subplot(111)
plt.yscale('log')
plt.plot(gamma, I_raw_refl, 'b-',  label = 'Reflected beam before background subtraction')
plt.errorbar(gamma, I_sub_refl, err_I_sub_refl,
             fmt = 'k.-', label = 'Reflected beam after background subtraction')
plt.plot(gamma, I_bckg_refl, 'r-', label = 'Background')
if is_bckg_up: plt.plot(gamma, I_bckg_up, 'g--', label = 'Background up')
if is_bckg_down: plt.plot(gamma, I_bckg_down, 'm--', label = 'Background down')
if is_bckg_left: plt.plot(gamma, I_bckg_left, 'g-.', label = 'Background left')
if is_bckg_right: plt.plot(gamma, I_bckg_right, 'm-.', label = 'Background right')
ax.set_xlabel('2*theta (deg)', fontsize=16)
ax.set_ylabel('I', fontsize=16)
plt.legend()
ax.tick_params(labelsize=16)
ax.yaxis.offsetText.set_fontsize(16)
plt.show()
```

# Normalization by the corrected voltage of the ionization chamber¶

We extract the voltage measured by the ionization chamber and its gain.  
An increase of 1 in the gain corresponds to a factor 10 in the measured voltage, with a saturation at 10 V.  
**We assume that the voltage is proportional to the intensity of the incident beam**, provided that the gain is high enough. Note that a gain higher than 7 may induce non-linearities, as it is the case in this example.

The intensity of the reflected beam is simply divided by the corresponding voltage of the ionization chamber V, corrected by its gain, and so are the errors and the background.

In [10]:

```
V_refl = np.array([])

for nxs_filename in nxs_filenames:  
   
    # Extract info from nexus file
    nexus = PN.PyNexusFile(recording_dir+nxs_filename, fast=True)
    stamps0D, data0D = nexus.extractData("0D")
    sensor_list = [stamps0D[i][0] if stamps0D[i][1]== None else stamps0D[i][1] for i in range(len(stamps0D))]
    
    # Get the voltage of the ionization chamber
    ionchamberArg = sensor_list.index('ionchamber')
    ionchamber = data0D[ionchamberArg]
    
    # Get the gain of the ionization chamber
    gainArg = sensor_list.index('commandfemtoionchamber')
    gain = data0D[gainArg]
    
    # In this example, there was a non-linearity between gain 8 and the previous ones
    # The factor introduced here has been measured elsewhere
    if gain[0] == 8:
        ionchamber = ionchamber/1.4025
    
    # Conversion in float
    gain = gain.astype(float)
    
    V_refl = np.append(V_refl, ionchamber*10**(-gain))

# Normalize by V
I_sub_over_V_refl = I_sub_refl/V_refl
I_bckg_over_V_refl = I_bckg_refl/V_refl
err_I_sub_over_V_refl = err_I_sub_refl/V_refl
```

We plot the normalized intensity as a function of 2\*theta, now this looks like a XRR curve. The last missing thing is the normalization by the direct beam.

In [11]:

```
fig = plt.figure(figsize=(12,5))
ax=fig.add_subplot(111)
plt.yscale('log')
plt.errorbar(gamma, I_sub_over_V_refl, err_I_sub_over_V_refl, fmt = 'k.-',
             label = 'Reflected beam after subtraction')
plt.plot(gamma, I_bckg_over_V_refl, 'r-', label = 'Background')
plt.plot(gamma, I_sub_over_V_refl+I_bckg_over_V_refl,
         'b-', label = 'Reflected beam before subtraction')
ax.set_xlabel('2*theta (deg)', fontsize=16)
ax.set_ylabel('I', fontsize=16)
ax.tick_params(labelsize=16)
plt.legend()
ax.yaxis.offsetText.set_fontsize(16)
plt.show()
```

# Normalization by the direct beam¶

Every steps made above should be performed as well on the direct beam.

We can have two types of direct scans:

- a height scan. In this case, we extract the intensity of the direct beam from the first 5 points of the scan.
- a time scan. In this case, we average over the whole scan.

## Finding the position of the direct beam¶

The direct beam is easily found by taking the position of maximum on the whole detector.

In [12]:

```
nxs_filename = 'SIRIUS_2022_02_23_4080.nxs'

# Extract info from nexus file
nexus = PN.PyNexusFile(recording_dir+nxs_filename, fast=True)
stamps0D, data0D = nexus.extractData("0D")
sensor_list = [stamps0D[i][0] if stamps0D[i][1]== None else stamps0D[i][1] for i in range(len(stamps0D))]

# Check if the direct scan is a height scan or a time scan
if sensor_list[0] == 'zs':
    is_direct_height_scan = True
    print('Direct scan is a height scan.')
    print('Taking the average over the 5 first points.')
    
    # Extract all the images in the scan
    images_direct, _, _, _, _, _, _, _  =DetectorSum.Extract(nxs_filename, recording_dir,
                                                     show_data_stamps=False, verbose=False)

    image_direct = np.mean(images_direct[0:5], axis = 0)
else:
    is_direct_height_scan = False
    print('Direct scan is not a height scan (probably a time scan).')
    print('Taking the average over the whole scan.')

    # Extract all the images in the scan
    _, image_direct, _, _, _, _, _, _  =DetectorSum.Extract(nxs_filename, recording_dir,
                                                     show_data_stamps=False, verbose=False)

    image_direct = np.mean(images_direct, axis = 0)

# Replace the intensity of the dead zones with a value of 0
image_direct=np.where(image_direct<0., 0., image_direct)

# Integrate along the horizontal axis and find the maximum
integrated_x_direct = image_direct.sum(axis=1)
pos_y_direct = np.argmax(integrated_x_direct)

print('Position maximum = %s'%pos_y_direct)

# ROI_direct = [ROIx0, pos_y_direct-ROIsizey//2,  ROIsizex, ROIsizey]
ROIy0_direct = pos_y_direct-ROIsizey//2

summation_ROI_direct = image_direct[ROIy0_direct : ROIy0_direct+ROIsizey,
                                    ROIx0 : ROIx0+ROIsizex]

print('Summation ROI = (%s, %s); (%s, %s)'%(ROIy0_direct, ROIx0,
                                            ROIy0_direct+ROIsizey-1, ROIx0+ROIsizex-1))

fig, ax0  = plt.subplots(figsize=(15,4))
im = ax0.pcolormesh(summation_ROI_direct, cmap = 'jet', shading = 'auto', rasterized=True)
fig.colorbar(im, ax=ax0)
ax0.set(xlabel = 'horizontal pixel (x)', ylabel ='vertical pixel (y)')
ax0.invert_yaxis()
plt.show()
```

```
Direct scan is a height scan.
Taking the average over the 5 first points.
Position maximum = 951        
Summation ROI = (949, 566); (953, 579)
```

## Extract the intensity of the direct beam¶

In [13]:

```
# Extract info from nexus file
nexus = PN.PyNexusFile(recording_dir+nxs_filename, fast=True)
stamps0D, data0D = nexus.extractData("0D")
sensor_list = [stamps0D[i][0] if stamps0D[i][1]== None else stamps0D[i][1] for i in range(len(stamps0D))]

integration_timeArg = sensor_list.index('integration_time')
integration_time = np.mean(data0D[integration_timeArg])

# Sum the ROI and normalize with the integration time
I_raw_direct = summation_ROI_direct.sum()/integration_time

print('Raw value of the direct beam (before subtraction & normalization) = %g'%I_raw_direct)
```

```
Raw value of the direct beam (before subtraction & normalization) = 1.01427e+06
```

## Background subtraction¶

The background is defined the same way as for the reflected beam.

In [14]:

```
bckg_ROI_up_direct = image_direct[ROIy0_direct-ROIsizey : ROIy0_direct,
                                  ROIx0 : ROIx0+ROIsizex]

bckg_ROI_down_direct = image_direct[ROIy0_direct+ROIsizey : ROIy0_direct+2*ROIsizey,
                                    ROIx0 : ROIx0+ROIsizex]

bckg_ROI_left_direct = image_direct[ROIy0_direct : ROIy0_direct+ROIsizey,
                                    ROIx0-ROIsizex : ROIx0]

bckg_ROI_right_direct = image_direct[ROIy0_direct : ROIy0_direct+ROIsizey,
                                     ROIx0+ROIsizex : ROIx0+2*ROIsizex]

# Sum the ROI and normalize with the integration time
I_bckg_up_direct = bckg_ROI_up_direct.sum()/integration_time
I_bckg_down_direct = bckg_ROI_down_direct.sum()/integration_time
I_bckg_left_direct = bckg_ROI_left_direct.sum()/integration_time
I_bckg_right_direct = bckg_ROI_right_direct.sum()/integration_time

if any([is_bckg_up, is_bckg_down, is_bckg_left, is_bckg_right]):
    # Take the average of the chosen backgrounds ROIs
    print('Background taken: '+is_bckg_up*'up '+is_bckg_down*'down '+is_bckg_left*'left '+is_bckg_right*'right ')
    I_bckg_direct = (is_bckg_up*I_bckg_up_direct+is_bckg_down*I_bckg_down_direct+is_bckg_left*I_bckg_left_direct+is_bckg_right*I_bckg_right_direct)/(is_bckg_up*1.+is_bckg_down*1.+is_bckg_left*1.+is_bckg_right*1.)
else:    
    print('No subtraction done.')
    I_bckg_direct = 0.
   
I_sub_direct = I_raw_direct - I_bckg_direct

# Draw a larger ROI for display of the background
image_ROI_display_direct = image_direct[ROIy0_direct-ROIsizey : ROIy0_direct+2*ROIsizey,
                                        ROIx0-ROIsizex : ROIx0+2*ROIsizex]  
  

fig, ax0  = plt.subplots(figsize=(15,4))

rect_up = patches.Rectangle((ROIsizex,0), ROIsizex, ROIsizey, linewidth=2, edgecolor='r', facecolor='none')
rect_down = patches.Rectangle((ROIsizex,2*ROIsizey), ROIsizex, ROIsizey, linewidth=2, edgecolor='r', facecolor='none')
rect_left = patches.Rectangle((0,ROIsizey), ROIsizex, ROIsizey, linewidth=2, edgecolor='w', facecolor='none')
rect_right = patches.Rectangle((2*ROIsizex,ROIsizey), ROIsizex, ROIsizey, linewidth=2, edgecolor='w', facecolor='none')
im = ax0.pcolormesh(image_ROI_display_direct, cmap = 'jet', shading = 'auto', rasterized=True)
ax0.add_patch(rect_up)
ax0.add_patch(rect_down)
ax0.add_patch(rect_left)
ax0.add_patch(rect_right)
ax0.set(xlabel = 'horizontal pixel (x)', ylabel ='vertical pixel (y)')
ax0.invert_yaxis()
ax0.set_title('Linear scale', fontsize=16)
plt.show()


fig, ax1  = plt.subplots(figsize=(15,4))
rect_up = patches.Rectangle((ROIsizex,0), ROIsizex, ROIsizey, linewidth=2, edgecolor='r', facecolor='none')
rect_down = patches.Rectangle((ROIsizex,2*ROIsizey), ROIsizex, ROIsizey, linewidth=2, edgecolor='r', facecolor='none')
rect_left = patches.Rectangle((0,ROIsizey), ROIsizex, ROIsizey, linewidth=2, edgecolor='w', facecolor='none')
rect_right = patches.Rectangle((2*ROIsizex,ROIsizey), ROIsizex, ROIsizey, linewidth=2, edgecolor='w', facecolor='none')
im = ax1.pcolormesh(image_ROI_display_direct, norm = colors.LogNorm(), cmap = 'jet', shading = 'auto', rasterized=True)
ax1.add_patch(rect_up)
ax1.add_patch(rect_down)
ax1.add_patch(rect_left)
ax1.add_patch(rect_right)
ax1.set(xlabel = 'horizontal pixel (x)', ylabel ='vertical pixel (y)')
ax1.invert_yaxis()
ax1.set_title('Log scale', fontsize=16)
plt.show()
```

```
Background taken: up down
```

## Normalization by the corrected voltage of the ionization chamber¶

In [15]:

```
# Extract info from nexus file
nexus = PN.PyNexusFile(recording_dir+nxs_filename, fast=True)
stamps0D, data0D = nexus.extractData("0D")
sensor_list = [stamps0D[i][0] if stamps0D[i][1]== None else stamps0D[i][1] for i in range(len(stamps0D))]

# Get the voltage of the ionization chamber
ionchamberArg = sensor_list.index('ionchamber')
# Get the gain of the ionization chamber
gainArg = sensor_list.index('commandfemtoionchamber')

if is_direct_height_scan:
    ionchamber = np.mean(data0D[ionchamberArg][0:5])
    gain = np.mean(data0D[gainArg][0:5])
else:
    ionchamber = np.mean(data0D[ionchamberArg])
    gain = np.mean(data0D[gainArg])
    
# In this example, there was a non-linearity between gain 8 and the previous ones
# The factor introduced here has been measured elsewhere
if np.isclose(gain,8):
    ionchamber = ionchamber/1.4025
    
# Conversion in float
gain = gain.astype(float)

V_direct = ionchamber*10**(-gain)

# Normalize by V
I_sub_over_V_direct = I_sub_direct/V_direct

print('Intensity of the direct beam = %g after background subtraction & normalization by V.'%(I_sub_over_V_direct))
```

```
Intensity of the direct beam = 5.85002e+13 after background subtraction & normalization by V.
```

# Normalization by the direct beam : XRR curve¶

Finally, we normalize the intensity of the reflected beam with the direct beam and obtain the XRR curve.

In [16]:

```
R = I_sub_over_V_refl/I_sub_over_V_direct
err_R = err_I_sub_over_V_refl/I_sub_over_V_direct
bckg_R = I_bckg_over_V_refl/I_sub_over_V_direct
```

We plot the results for $q\_z= 4\pi/\lambda\sin(\theta)$ as well, where **$\lambda$ is the wavelength provided by the user in JupyLabBook**, and for $Iq\_z^4$ to normalize by the Fresnel reflectivity.

In [17]:

```
# lambda in nm
wavelength = 0.12398 
qz = 4*np.pi/wavelength*np.sin(gamma*np.pi/180./2.)

fig = plt.figure(figsize=(12,5))
ax=fig.add_subplot(111)
plt.yscale('log')
plt.errorbar(gamma, R, err_R, fmt = 'k.-', label = 'XRR after subtraction')
plt.plot(gamma, R+bckg_R, 'b-', label = 'XRR before subtraction')
plt.plot(gamma, bckg_R, 'r-', label = 'Background')
plt.legend()
ax.set_xlabel('2*theta (deg)', fontsize=16)
ax.set_ylabel('R', fontsize=16)
ax.tick_params(labelsize=16)
ax.yaxis.offsetText.set_fontsize(16)
plt.show()

fig = plt.figure(figsize=(12,5))
ax=fig.add_subplot(111)
plt.yscale('log')
plt.errorbar(qz, R, err_R, fmt = 'k.-')
ax.set_xlabel('qz (nm-1)', fontsize=16)
ax.set_ylabel('R', fontsize=16)
ax.tick_params(labelsize=16)
ax.yaxis.offsetText.set_fontsize(16)
plt.show()

fig = plt.figure(figsize=(12,5))
ax=fig.add_subplot(111)
plt.yscale('log')
plt.ylim(1e-6, 1e-2)
plt.errorbar(qz, R*qz**4, err_R*qz**4, fmt = 'k.-')
ax.set_xlabel('qz (nm-1)', fontsize=16)
ax.set_ylabel('R*qz^4 (nm-4)', fontsize=16)
ax.tick_params(labelsize=16)
ax.yaxis.offsetText.set_fontsize(16)
plt.show()
```

# Summary of the useful variables¶

- `I_raw_refl` & `I_raw_direct`: the intensity obtained by summing all the pixels in the summation ROI, which is centered on the reflected/direct beams.
- `I_bckg_refl` & `I_bckg_direct`: the same for the direct beam.
- `I_sub_refl` & `I_sub_direct`: `I_raw`-`I_bckg`.
- `V_refl` & `V_direct`: voltage of the ionization chamber normalized by its gain, proportional to the intensity of the incident beam.
- `R`: the reflectivity, with all the proper normalizations done.
- `err_R`: the error bars on the reflectivity.
