# Supplementary material for: Opportunities and new developments for the study of surfaces and interfaces in soft condensed matter at the SIRIUS beamline of Synchrotron SOLEIL
Source: J Synchrotron Radiat. 2024 Jan 1;31(Pt 1):162–76. doi: 10.1107/S1600577523008810 (PMC10833424; doi:10.1107/S1600577523008810)
Supplement: Supplementary file 1 [file s-31-00162-sup1.zip › JupyLabBook-v3.0.2/docs/sphinx/build/html/genindex.html]

Index — JupyLabBook v3.0 documentation

### Navigation

- index
- modules |
- JupyLabBook v3.0 documentation »
- Index

# Index

**B**
| **C**
| **D**
| **E**
| **F**
| **G**
| **I**
| **L**
| **M**
| **N**
| **P**
| **R**
| **S**
| **U**

## B

|  |
| --- |
| - bin\_matrix\_vertical() (in module lib.backend.gixd) |

## C

|  |  |
| --- | --- |
| - calib\_thetaz() (in module lib.backend.gixd) - check\_and\_set\_paths() (lib.frontend.experiment.Experiment method) - check\_sdd\_elems() (in module lib.backend.xrf) - close() (lib.backend.PyNexus.PyNexusFile method) | - command (lib.frontend.scan.Scan attribute) - compute\_bragg\_new() (in module lib.backend.data\_1d) - create\_cell() (in module lib.frontend.notebook) - create\_cell\_action\_widgets() (in module lib.frontend.notebook) |

## D

|  |  |
| --- | --- |
| - default (lib.frontend.experiment.Experiment attribute) - default\_log (lib.frontend.experiment.Experiment attribute) - delete\_cell() (in module lib.frontend.notebook) - display\_action() (in module lib.jupylabbook) - display\_interactive\_1d\_plot() (in module lib.frontend.process\_widgets.wdata\_1d) - display\_process() (in module lib.jupylabbook) - display\_widgets\_1d\_fit() (in module lib.frontend.process\_widgets.wdata\_1d) - display\_widgets\_action() (in module lib.frontend.action) - display\_widgets\_area\_detector() (in module lib.frontend.process\_widgets.warea\_detector) - display\_widgets\_calib\_thetaz() (in module lib.frontend.process\_widgets.wgixd) - display\_widgets\_calib\_xrr\_liquid() (in module lib.frontend.process\_widgets.wxrr) - display\_widgets\_data\_1d() (in module lib.frontend.process\_widgets.wdata\_1d) - display\_widgets\_energy\_calib() (in module lib.frontend.process\_widgets.wdata\_1d) - display\_widgets\_form() (in module lib.frontend.form) | - display\_widgets\_gixd() (in module lib.frontend.process\_widgets.wgixd) - display\_widgets\_gixs() (in module lib.frontend.process\_widgets.wgixs) - display\_widgets\_identify\_peaks() (in module lib.frontend.process\_widgets.wxrf) - display\_widgets\_insert\_from\_log() (in module lib.frontend.jlb\_io) - display\_widgets\_insert\_image() (in module lib.frontend.jlb\_io) - display\_widgets\_insert\_script() (in module lib.frontend.jlb\_io) - display\_widgets\_insert\_text() (in module lib.frontend.jlb\_io) - display\_widgets\_isotherm() (in module lib.frontend.process\_widgets.wisotherm) - display\_widgets\_load\_params\_from\_json() (in module lib.frontend.jlb\_io) - display\_widgets\_process() (in module lib.frontend.process) - display\_widgets\_save\_params\_in\_json() (in module lib.frontend.jlb\_io) - display\_widgets\_vineyard() (in module lib.frontend.process\_widgets.wgixd) - display\_widgets\_xrf() (in module lib.frontend.process\_widgets.wxrf) - display\_widgets\_xrr\_liquid() (in module lib.frontend.process\_widgets.wxrr) - display\_widgets\_xrr\_solid() (in module lib.frontend.process\_widgets.wxrr) |

## E

|  |  |
| --- | --- |
| - erf\_function() (in module lib.backend.data\_1d) - Experiment (class in lib.frontend.experiment) - export\_logs\_to\_rlogs() (in module lib.frontend.jlb\_io) - export\_nb\_to\_pdf() (in module lib.frontend.jlb\_io) - extract\_absorbers\_from\_log() (in module lib.frontend.jlb\_io) - extract\_area\_detector\_scan() (in module lib.backend.area\_detector) - extract\_commands\_from\_log() (in module lib.frontend.jlb\_io) - extract\_data\_1d() (in module lib.backend.data\_1d) - extract\_dead\_pixels() (in module lib.frontend.jlb\_io) - extract\_direct\_xrr\_liquid() (in module lib.backend.xrr) - extract\_direct\_xrr\_solid() (in module lib.backend.xrr) - extract\_gixd\_scan() (in module lib.backend.gixd) | - extract\_gixs\_scan() (in module lib.backend.gixs) - extract\_isotherm\_scan() (in module lib.backend.isotherm) - extract\_one\_data\_point() (lib.backend.PyNexus.PyNexusFile method) - extract\_positions\_from\_log() (in module lib.frontend.jlb\_io) - extract\_scan\_data() (lib.backend.PyNexus.PyNexusFile method) - extract\_volt\_ion\_chamber\_liquid() (in module lib.backend.xrr) - extract\_xrf\_scan() (in module lib.backend.xrf) - extract\_xrr\_liquid\_scan() (in module lib.backend.xrr) - extract\_xrr\_solid\_scan() (in module lib.backend.xrr) - extractAndSave2DData() (lib.backend.PyNexus.PyNexusFile method) - extractData() (lib.backend.PyNexus.PyNexusFile method) - extractDataStamp() (lib.backend.PyNexus.PyNexusFile method) - extractStamps() (lib.backend.PyNexus.PyNexusFile method) |

## F

|  |  |
| --- | --- |
| - fit\_with\_erf() (in module lib.backend.data\_1d) | - fit\_with\_gaussian() (in module lib.backend.data\_1d) |

## G

|  |  |
| --- | --- |
| - gaussian\_function() (in module lib.backend.data\_1d) - get\_aliases() (in module lib.backend.PyNexus) | - get\_default\_param\_value() (lib.frontend.experiment.Experiment method) - get\_nbpts() (lib.backend.PyNexus.PyNexusFile method) |

## I

|  |
| --- |
| - is\_paths\_ok (lib.frontend.experiment.Experiment attribute) |

## L

|  |  |
| --- | --- |
| - lib   - module - lib.backend   - module - lib.backend.area\_detector   - module - lib.backend.data\_1d   - module - lib.backend.gixd   - module - lib.backend.gixs   - module - lib.backend.isotherm   - module - lib.backend.PyNexus   - module - lib.backend.xrf   - module - lib.backend.xrr   - module - lib.frontend   - module - lib.frontend.action   - module - lib.frontend.experiment   - module - lib.frontend.form   - module - lib.frontend.jlb\_io   - module | - lib.frontend.notebook   - module - lib.frontend.process   - module - lib.frontend.process\_widgets   - module - lib.frontend.process\_widgets.warea\_detector   - module - lib.frontend.process\_widgets.wdata\_1d   - module - lib.frontend.process\_widgets.wgixd   - module - lib.frontend.process\_widgets.wgixs   - module - lib.frontend.process\_widgets.wisotherm   - module - lib.frontend.process\_widgets.wxrf   - module - lib.frontend.process\_widgets.wxrr   - module - lib.frontend.scan   - module - lib.jupylabbook   - module - list\_logs (lib.frontend.experiment.Experiment attribute) - list\_nxs (lib.frontend.experiment.Experiment attribute) - list\_params\_files (lib.frontend.experiment.Experiment attribute) - list\_scans (lib.frontend.experiment.Experiment attribute) - list\_scripts (lib.frontend.experiment.Experiment attribute) - load\_params\_from\_json() (lib.frontend.experiment.Experiment method) |

## M

|  |
| --- |
| - module   - lib   - lib.backend   - lib.backend.area\_detector   - lib.backend.data\_1d   - lib.backend.gixd   - lib.backend.gixs   - lib.backend.isotherm   - lib.backend.PyNexus   - lib.backend.xrf   - lib.backend.xrr   - lib.frontend   - lib.frontend.action   - lib.frontend.experiment   - lib.frontend.form   - lib.frontend.jlb\_io   - lib.frontend.notebook   - lib.frontend.process   - lib.frontend.process\_widgets   - lib.frontend.process\_widgets.warea\_detector   - lib.frontend.process\_widgets.wdata\_1d   - lib.frontend.process\_widgets.wgixd   - lib.frontend.process\_widgets.wgixs   - lib.frontend.process\_widgets.wisotherm   - lib.frontend.process\_widgets.wxrf   - lib.frontend.process\_widgets.wxrr   - lib.frontend.scan   - lib.jupylabbook |

## N

|  |  |
| --- | --- |
| - name (lib.frontend.scan.Scan attribute) | - nxs\_name (lib.frontend.scan.Scan attribute) |

## P

|  |  |
| --- | --- |
| - params (lib.frontend.experiment.Experiment attribute) - path\_to\_nxs (lib.frontend.scan.Scan attribute) - paths (lib.frontend.experiment.Experiment attribute) - plot\_area\_detector\_scan() (in module lib.backend.area\_detector) - plot\_calib\_xrr\_liquid() (in module lib.backend.xrr) - plot\_data\_1d() (in module lib.backend.data\_1d) - plot\_energy\_calib() (in module lib.backend.data\_1d) - plot\_erf\_fit() (in module lib.backend.data\_1d) - plot\_gaussian\_fit() (in module lib.backend.data\_1d) - plot\_gixd\_scan() (in module lib.backend.gixd) - plot\_gixs\_scan() (in module lib.backend.gixs) - plot\_isotherm\_scan() (in module lib.backend.isotherm) - plot\_vineyard() (in module lib.backend.gixd) - plot\_xrf\_first\_last() (in module lib.backend.xrf) - plot\_xrf\_spectrogram() (in module lib.backend.xrf) - plot\_xrf\_sum() (in module lib.backend.xrf) - plot\_xrr\_m4pitch() (in module lib.backend.xrr) - plot\_xrr\_pos\_y\_beam() (in module lib.backend.xrr) | - plot\_xrr\_qz() (in module lib.backend.xrr) - plot\_xrr\_twotheta() (in module lib.backend.xrr) - preview\_direct\_xrr() (in module lib.frontend.process\_widgets.wxrr) - preview\_indiv\_scan\_xrr() (in module lib.frontend.process\_widgets.wxrr) - print\_version() (in module lib.jupylabbook) - process\_area\_detector\_scan() (in module lib.backend.area\_detector) - process\_data\_1d() (in module lib.backend.data\_1d) - process\_energy\_calib() (in module lib.backend.data\_1d) - process\_erf\_fit() (in module lib.backend.data\_1d) - process\_gaussian\_fit() (in module lib.backend.data\_1d) - process\_gixd\_scan() (in module lib.backend.gixd) - process\_gixs\_scan() (in module lib.backend.gixs) - process\_isotherm\_scan() (in module lib.backend.isotherm) - process\_vineyard() (in module lib.backend.gixd) - process\_xrf\_scan() (in module lib.backend.xrf) - process\_xrr\_liquid\_scan() (in module lib.backend.xrr) - process\_xrr\_solid\_scan() (in module lib.backend.xrr) - PyNexusFile (class in lib.backend.PyNexus) |

## R

|  |  |
| --- | --- |
| - refresh\_cell() (in module lib.frontend.notebook) | - residuals\_erf\_function() (in module lib.backend.data\_1d) - residuals\_gaussian\_function() (in module lib.backend.data\_1d) |

## S

|  |  |
| --- | --- |
| - save\_area\_detector\_scan() (in module lib.backend.area\_detector) - save\_data\_1d() (in module lib.backend.data\_1d) - save\_fit\_result() (in module lib.backend.data\_1d) - save\_gixd\_scan() (in module lib.backend.gixd) - save\_gixs\_scan() (in module lib.backend.gixs) - save\_isotherm\_scan() (in module lib.backend.isotherm) - save\_nb() (in module lib.frontend.notebook) - save\_params\_in\_json() (lib.frontend.experiment.Experiment method) - save\_xrf\_scan() (in module lib.backend.xrf) - save\_xrr\_liquid\_scan() (in module lib.backend.xrr) - save\_xrr\_solid\_scan() (in module lib.backend.xrr) - saveExtractedData() (lib.backend.PyNexus.PyNexusFile method) | - saveOneDExtractedData() (lib.backend.PyNexus.PyNexusFile method) - savePointExtractedData() (lib.backend.PyNexus.PyNexusFile method) - saveTwoDExtractedData() (lib.backend.PyNexus.PyNexusFile method) - Scan (class in lib.frontend.scan) - set\_command() (lib.frontend.scan.Scan method) - set\_identifiers() (lib.frontend.scan.Scan method) - set\_list\_images() (lib.frontend.experiment.Experiment method) - set\_list\_logs() (lib.frontend.experiment.Experiment method) - set\_list\_nxs() (lib.frontend.experiment.Experiment method) - set\_list\_params\_files() (lib.frontend.experiment.Experiment method) - set\_list\_scripts() (lib.frontend.experiment.Experiment method) - set\_scans() (lib.frontend.experiment.Experiment method) - start() (in module lib.jupylabbook) |

## U

|  |
| --- |
| - update\_param\_from\_widget() (lib.frontend.experiment.Experiment method) |

### Quick search

### Navigation

- index
- modules |
- JupyLabBook v3.0 documentation »
- Index

© Copyright 2022, Hemmerle Arnaud.
Created using Sphinx 5.0.2.
