# Supplementary material for: Opportunities and new developments for the study of surfaces and interfaces in soft condensed matter at the SIRIUS beamline of Synchrotron SOLEIL
Source: J Synchrotron Radiat. 2024 Jan 1;31(Pt 1):162–76. doi: 10.1107/S1600577523008810 (PMC10833424; doi:10.1107/S1600577523008810)
Supplement: Supplementary file 1 [file s-31-00162-sup1.zip › JupyLabBook-v3.0.2/docs/sphinx/build/html/index.html]

Welcome to jupylabbook’s documentation! — JupyLabBook v3.0 documentation

### Navigation

- index
- modules |
- JupyLabBook v3.0 documentation »
- Welcome to jupylabbook’s documentation!

# Welcome to jupylabbook’s documentation!¶

# Indices and tables¶

- Index
- Module Index
- Search Page

### Table of Contents

- Welcome to jupylabbook’s documentation!
- Indices and tables

### This Page

- Show Source

### Quick search

### Navigation

- index
- modules |
- JupyLabBook v3.0 documentation »
- Welcome to jupylabbook’s documentation!

© Copyright 2022, Hemmerle Arnaud.
Created using Sphinx 5.0.2.
