# Supplementary material for: Opportunities and new developments for the study of surfaces and interfaces in soft condensed matter at the SIRIUS beamline of Synchrotron SOLEIL
Source: J Synchrotron Radiat. 2024 Jan 1;31(Pt 1):162–76. doi: 10.1107/S1600577523008810 (PMC10833424; doi:10.1107/S1600577523008810)
Supplement: Supplementary file 1 [file s-31-00162-sup1.zip › JupyLabBook-v3.0.2/docs/sphinx/build/html/lib.backend.html]

lib.backend package — JupyLabBook v3.0 documentation

### Navigation

- index
- modules |
- JupyLabBook v3.0 documentation »
- lib.backend package

# lib.backend package¶

## Submodules¶

## lib.backend.PyNexus module¶

*class* lib.backend.PyNexus.PyNexusFile(*filename*, *aliases=None*, *\*\*keywords*)¶
:   Bases: `object`

    Class for a SOLEIL Nexus file

    close()¶
    :   Close the Nexus File

    extractAndSave2DData()¶
    :   extract and save the 2D data from scan\_data
        output:

        > nb\_data\_saved : numer of data point saved

    extractData(*which='all'*)¶
    :   extract the the data from scan\_data
        Input :

        > which : value = ‘all’, ‘0D’, ‘1D’, ‘2D’
        > SPECIFY IF ALL data, or only respectively point (0D), spectrum (1D)
        > or images (2D) have to be extracted.

        output:
        :   tuple with 2 elements :
            :   stamps : longname of the data
                data : data itself

    extractDataStamp(*which=None*)¶
    :   extract the the data from scan\_data
        Input :

        > which : the stamp of the data to extract

        output:
        :   data : data itself

    extractStamps()¶
    :   extract only the stamps from scan\_data
        Input :
        output:

        > stamps : list of the longname of the data

    extract\_one\_data\_point(*dataset*, *num*, *verbose=True*)¶
    :   extract one data point from scan\_data
        input : number of the point
        output:

        > the stamp
        > the data point (could be an array)

    extract\_scan\_data()¶
    :   return all the scan data extracted from the nexus file
        Input : None
        Output : a tuple with 2 elements

        > - stamps : the name of the registered quantities
        > - the data itself as a numpy array

    get\_nbpts()¶
    :   return the number of points recorded in the nexus file

    saveExtractedData(*result*)¶
    :   Save all the previously extracted data to files
        Input : result : a tuple with stamp (name) and data coming from the extract functions
        Output : the file on the disk, .dat for 0D point data, .mat for spectrum data, and .imag for image data

    saveOneDExtractedData(*result*)¶
    :   Save only the 1D spectrum previously extracted data to a .mat file
        Input : result : a tuple with stamp (name) and data coming from the extract functions
        Output : the file on the disk

    savePointExtractedData(*result*)¶
    :   Save only the 0D, point, scalar, previously extracted data to a .dat file
        Input : result : a tuple with stamp (name) and data coming from the extract functions
        Output : the file on the disk

    saveTwoDExtractedData(*result*)¶
    :   Save only the 2D Image previously extracted data to a .imag file
        Input : result : a tuple with stamp (name) and data coming from the extract functions
        Output : the file on the disk

lib.backend.PyNexus.get\_aliases(*filename*)¶
:   build a dictionnary of the aliases and tango adresses from the elements
    in file filename
    Input :

    > filename : the config file

    Output:
    :   a dictionnary which keywords are the aliases

## lib.backend.area\_detector module¶

Library for area detector.

lib.backend.area\_detector.extract\_area\_detector\_scan(*nxs\_name*, *path\_to\_nxs\_dir*, *is\_print\_stamps*, *is\_print\_info*)¶
:   Extract the nexus file and return useful quantities.

    Parameters
    :   - **nxs\_name** (*str*) – Nexus name, e.g. SIRIUS\_2020\_03\_12\_0756.nxs.
        - **path\_to\_nxs\_dir** (*str*) – Path to the nexus files directory, e.g. user/.
        - **is\_print\_stamps** (*bool**,* *optional*) – Print the list of sensors contained in the nexus file.
        - **is\_print\_info** (*bool**,* *optional*) – Verbose mode.

    Returns
    :   - **images** (*array*) – Array of individual detector images.
        - **images\_sum** (*array*) – Detector images integrated over the scan.
        - **integ\_x** (*array*) – Profile integrated along the horizontal axis.
        - **integ\_y** (*array*) – Profile integrated along the vertical axis.
        - **alias\_detector** (*str*) – The alias of the 2D detector, e.g. ‘pilatus’ or ‘ufxc’.
        - **time\_str** (*str*) – Starting/ending dates of the scan.
        - **stamps\_0d** (*array*) – Aliases of each 0D sensor in the scan.
        - **data\_0d** (*array*) – Values of each 0D sensor in the scan.

    Raises
    :   - **FileNotFoundError** – If a file or a folder is missing.
        - **SystemExit****(****'Could not open Nexus file.'****)****)** – If the Nexus file cannot be accessed.
        - **Exception****(****'Area detector not found.'****)** – If no area detector is found in the sensor list.

lib.backend.area\_detector.plot\_area\_detector\_scan(*images\_sum*, *integ\_x*, *integ\_y*, *nxs\_name*, *absorbers*, *is\_area\_detector\_logz*, *map\_area\_detector*, *x\_min*, *x\_max*, *y\_min*, *y\_max*, *time\_str*)¶
:   Plot area detector data.

    Parameters
    :   - **images\_sum** (*array*) – Detector images integrated over the scan.
        - **integ\_x** (*array*) – Profile integrated along the horizontal axis.
        - **integ\_y** (*array*) – Profile integrated along the vertical axis.
        - **nxs\_name** (*str*) – Nexus name, e.g. SIRIUS\_2020\_03\_12\_0756.nxs.
        - **absorbers** (*str**,* *optional*) – Text to display which absorber was used.
        - **is\_area\_detector\_logz** (*bool**,* *optional*) – Log scale on the color scale of the image.
        - **map\_area\_detector** (*str**,* *optional*) – Colormap of the image.
        - **x\_min** (*float**,* *optional*) – Min limit of the vertical profile plot (integrated over the horizontal axis).
        - **x\_max** (*float**,* *optional*) – Max limit of the vertical profile plot (integrated over the horizontal axis).
        - **y\_min** (*float**,* *optional*) – Min limit of the horizontal profile plot (integrated over the vertical axis).
        - **y\_max** (*float**,* *optional*) – Max limit of the horizontal profile plot (integrated over the vertical axis).
        - **time\_str** (*str*) – Starting/ending dates of the scan.

    Returns
    :   **fig** – The figure to be saved in pdf.

    Return type
    :   matplotlib figure

lib.backend.area\_detector.process\_area\_detector\_scan(*nxs\_name*, *path\_to\_nxs\_dir*, *x\_min=0*, *x\_max=980*, *y\_min=0*, *y\_max=1042*, *absorbers=''*, *is\_area\_detector\_logz=True*, *map\_area\_detector='viridis'*, *path\_to\_save\_dir=''*, *is\_print\_stamps=False*, *is\_plot=False*, *is\_save\_sum=False*, *is\_save\_each=False*, *is\_print\_info=False*)¶
:   Call functions for extracting, plotting, and saving an output of a scan on an area detector.

    Parameters
    :   - **nxs\_name** (*str*) – Nexus name, e.g. SIRIUS\_2020\_03\_12\_0756.nxs.
        - **path\_to\_nxs\_dir** (*str*) – Path to the nexus files directory, e.g. user/.
        - **x\_min** (*float**,* *optional*) – Min limit of the vertical profile plot (integrated over the horizontal axis).
        - **x\_max** (*float**,* *optional*) – Max limit of the vertical profile plot (integrated over the horizontal axis).
        - **y\_min** (*float**,* *optional*) – Min limit of the horizontal profile plot (integrated over the vertical axis).
        - **y\_max** (*float**,* *optional*) – Max limit of the horizontal profile plot (integrated over the vertical axis).
        - **absorbers** (*str**,* *optional*) – Text to display which absorber was used.
        - **is\_area\_detector\_logz** (*bool**,* *optional*) – Log scale on the color scale of the image.
        - **map\_area\_detector** (*str**,* *optional*) – Colormap of the image.
        - **path\_to\_save\_dir** (*str**,* *optional*) – Path to the directory where the treated files will be saved.
        - **is\_print\_stamps** (*bool**,* *optional*) – Print the list of sensors contained in the nexus file.
        - **is\_plot** (*bool**,* *optional*) – Plot the 2D GIXD image and the integrated profiles.
        - **is\_save\_sum** (*bool**,* *optional*) – Save the sum of the images.
        - **is\_save\_each** (*bool**,* *optional*) – Save each individual image and the sum.
        - **is\_print\_info** (*bool**,* *optional*) – Verbose mode.

    Returns
    :   - **images\_sum** (*array*) – Detector images integrated over the scan.
        - **integ\_x** (*array*) – Profile integrated along the horizontal axis.
        - **integ\_y** (*array*) – Profile integrated along the vertical axis.

lib.backend.area\_detector.save\_area\_detector\_scan(*images*, *images\_sum*, *integ\_x*, *integ\_y*, *alias\_detector*, *nxs\_name*, *stamps\_0d*, *data\_0d*, *fig*, *path\_to\_save\_dir*, *is\_print\_info*)¶
:   Save area detector data.

    XXX\_DETECTOR\_sum.mat: the matrix corresponding to the image displayed, in ascii.
    XXX\_DETECTOR\_sum.tiff: the matrix corresponding to the image displayed, in tiff.
    XXX\_integrated\_x.dat: the horizontal integration of the whole detector as a function of y.
    XXX\_integrated\_y.dat: the vertical integration of the whole detector as a function of x.
    XXX\_images/XXX\_DETECTOR\_N.tiff : each image of the scan, in tiff.
    XXX.dat : the value of each sensor at each point of the scan.
    XXX.pdf : the figure in pdf.

    Parameters
    :   - **images** (*array*) – Array of individual detector images.
        - **images\_sum** (*array*) – Detector images integrated over the scan.
        - **integ\_x** (*array*) – Profile integrated along the horizontal axis.
        - **integ\_y** (*array*) – Profile integrated along the vertical axis.
        - **alias\_detector** (*str*) – The alias of the 2D detector, e.g. ‘pilatus’ or ‘ufxc’.
        - **nxs\_name** (*str*) – Nexus name, e.g. SIRIUS\_2020\_03\_12\_0756.nxs.
        - **stamps\_0d** (*array*) – Aliases of each 0D sensor in the scan.
        - **data\_0d** (*array*) – Values of each 0D sensor in the scan.
        - **fig** (*None* *or* *matplotlib figure*) – The figure to be saved in pdf. Pass None if not wanted.
        - **path\_to\_save\_dir** (*str*) – Path to the directory where the treated files will be saved.
        - **is\_print\_info** (*bool*) – Verbose mode.

## lib.backend.data\_1d module¶

Library for data 1D.

lib.backend.data\_1d.compute\_bragg\_new(*stamps\_0d*, *data\_0d*, *xas\_energy\_shift*, *xas\_signal\_label*, *xas\_norm\_label*, *xas\_standard\_name*, *energy\_current*, *bragg\_current*, *path\_to\_xas\_dir*)¶
:   Compute the new bragg value based on the energy shift with the standard.

    Parameters
    :   - **stamps\_0d** (*array*) – Aliases of each 0D sensor in the scan.
        - **data\_0d** (*array*) – Values of each 0D sensor in the scan.
        - **xas\_energy\_shift** (*float*) – Energy shift between the measurement and the standard in keV.
        - **xas\_signal\_label** (*str*) – Exact name of the sensor used for signal, as it appears in the stamps.
        - **xas\_norm\_label** (*str*) – Exact name of the sensor used for normalization, as it appears in the stamps.
        - **xas\_standard\_name** (*str*) – Chosen xas standard file, e.g. ‘XAS\_ref\_Ni.txt’.
        - **energy\_current** (*float*) – Current value of the DCM energy in keV.
        - **bragg\_current** (*float*) – Current value of the bragg angle in deg.
        - **path\_to\_xas\_dir** (*str*) – Path to the folder containing the standards.

    Returns
    :   - **bragg\_new** (*float*) – Value of the new bragg in deg.
        - **xas\_energy\_standard** (*array*) – List of energies from the standard in keV.
        - **xas\_transmission\_standard** (*array*) – List of transmissions from the standard.
        - **xas\_energy\_meas** (*array*) – List of energies measured in keV.
        - **xas\_transmission\_meas** (*array*) – List of transmissions measured in keV.
        - **str\_to\_display** (*str or None*) – String to display after the plot.

lib.backend.data\_1d.erf\_function(*x*, *mu*, *sigma*, *amplitude*, *cst*, *sign*)¶
:   Return an error function with a constant background.

lib.backend.data\_1d.extract\_data\_1d(*nxs\_name*, *path\_to\_nxs\_dir*, *x\_label*, *y\_label*, *is\_print\_stamps*, *is\_print\_info*)¶
:   Extract the nexus file and return useful quantities.

    Parameters
    :   - **nxs\_name** (*str*) – Nexus name, e.g. SIRIUS\_2020\_03\_12\_0756.nxs.
        - **path\_to\_nxs\_dir** (*str*) – Path to the nexus files directory, e.g. user/.
        - **x\_label** (*str*) – Exact name of the x sensor, as it appears in the stamps.
        - **y\_label** (*str*) – Exact name of the y sensor, as it appears in the stamps.
        - **is\_print\_stamps** (*bool**,* *optional*) – Print the list of sensors contained in the nexus file.
        - **is\_print\_info** (*bool**,* *optional*) – Verbose mode.

    Returns
    :   - **x** (*array*) – List of x values.
        - **y** (*array*) – List of y values.
        - **time\_str** (*str*) – Starting/ending dates of the scan.
        - **stamps\_0d** (*array*) – Aliases of each 0D sensor in the scan.
        - **data\_0d** (*array*) – Values of each 0D sensor in the scan.

    Raises
    :   - **FileNotFoundError** – If a file or a folder is missing.
        - **Exception****(****'Could not open Nexus file.'****)****)** – If the Nexus file cannot be accessed.
        - **Exception****(****'Sensor not found.'****)** – If the sensor is not found in the sensor list.

lib.backend.data\_1d.fit\_with\_erf(*x*, *y*, *params\_init*, *is\_print\_info*)¶
:   Fit with an error function using LMFIT.

    Parameters
    :   - **x** (*array*) – List of x values.
        - **y** (*array*) – List of y values.
        - **params\_init** (*dict* *or* *None*) – Dictionnary of parameters for initial guesses and limits.
          All the parameters should be given. For example:
          params\_init = {‘cst’:{‘init’:0, ‘min’:-1, ‘max’:1},
          ‘mu’:{‘init’:0, ‘min’:-1, ‘max’:1},
          ‘sigma’:{‘init’:0.2, ‘min’:0., ‘max’:1},
          ‘amplitude’:{‘init’:10., ‘min’:-10, ‘max’:20},
          ‘sign’:{‘value’:-1}}
        - **is\_print\_info** (*bool*) – Verbose mode.

    Returns
    :   - **y\_fit** (*array*) – List of y values from the fit.
        - **lm\_result** (*object MinimizerResult*) – Results of lm minimization. Includes data such as status and error messages, fit statistics,
          and the updated (i.e., best-fit) parameters themselves in the params attribute.

lib.backend.data\_1d.fit\_with\_gaussian(*x*, *y*, *params\_init*, *is\_print\_info*)¶
:   Fit with a gaussian using LMFIT.

    Parameters
    :   - **x** (*array*) – List of x values.
        - **y** (*array*) – List of y values.
        - **params\_init** (*dict* *or* *None*) – Dictionnary of parameters for initial guesses and limits.
          All the parameters should be given. For example:
          params\_init = {‘cst’:{‘init’:0, ‘min’:-1, ‘max’:1},
          ‘linear\_coeff’:{‘init’:0, ‘min’:-1, ‘max’:1},
          ‘mu’:{‘init’:0, ‘min’:-1, ‘max’:1},
          ‘sigma’:{‘init’:0.2, ‘min’:0., ‘max’:1},
          ‘amplitude’:{‘init’:10., ‘min’:-10, ‘max’:20}}
        - **is\_print\_info** (*bool*) – Verbose mode.

    Returns
    :   - **y\_fit** (*array*) – List of y values from the fit.
        - **lm\_result** (*object MinimizerResult*) – Results of lm minimization. Includes data such as status and error messages, fit statistics,
          and the updated (i.e., best-fit) parameters themselves in the params attribute.

lib.backend.data\_1d.gaussian\_function(*x*, *mu*, *sigma*, *cst*, *linear\_coeff*, *amplitude*)¶
:   Return a gaussian with a linear background.

lib.backend.data\_1d.plot\_data\_1d(*x*, *y*, *x\_label*, *y\_label*, *time\_str*, *nxs\_name*, *absorbers*, *is\_logx*, *is\_logy*)¶
:   Plot 1d data.

    Parameters
    :   - **x** (*array*) – List of x values.
        - **y** (*array*) – List of y values.
        - **x\_label** (*str*) – Exact name of the x sensor, as it appears in the stamps.
        - **y\_label** (*str*) – Exact name of the y sensor, as it appears in the stamps.
        - **time\_str** (*str*) – Starting/ending dates of the scan.
        - **nxs\_name** (*str*) – Nexus name, e.g. SIRIUS\_2020\_03\_12\_0756.nxs.
        - **absorbers** (*str**,* *optional*) – Text to display which absorber was used.
        - **is\_logx** (*bool**,* *optional*) – Log scale on the x axis.
        - **is\_logy** (*bool**,* *optional*) – Log scale on the y axis.

    Returns
    :   **fig** – The figure to be saved in pdf.

    Return type
    :   matplotlib figure

lib.backend.data\_1d.plot\_energy\_calib(*xas\_standard\_name*, *xas\_energy\_min*, *xas\_energy\_max*, *xas\_energy\_shift*, *xas\_energy\_meas*, *xas\_transmission\_meas*, *xas\_energy\_standard*, *xas\_transmission\_standard*, *str\_to\_display*)¶
:   Plot the comparison with the xas standard and print the new bragg.

    Parameters
    :   - **xas\_standard\_name** (*str*) – Chosen xas standard file, e.g. ‘XAS\_ref\_Ni.txt’.
        - **xas\_energy\_min** (*float*) – Energy minimum for the plot in keV.
        - **xas\_energy\_max** (*float*) – Energy maximum for the plot in keV.
        - **xas\_energy\_shift** (*float*) – Energy shift between the measurement and the standard in keV.
        - **xas\_energy\_meas** (*array*) – List of energies measured in keV.
        - **xas\_transmission\_meas** (*array*) – List of transmissions measured in keV.
        - **xas\_energy\_standard** (*array*) – List of energies from the standard in keV.
        - **xas\_transmission\_standard** (*array*) – List of transmissions from the standard.
        - **str\_to\_display** (*str* *or* *None*) – String to display after the plot.

    Returns
    :   **fig** – The figure to be saved in pdf.

    Return type
    :   matplotlib figure

lib.backend.data\_1d.plot\_erf\_fit(*x*, *y*, *y\_fit*, *lm\_result*, *x\_label*, *y\_label*, *time\_str*, *nxs\_name*, *absorbers*, *is\_print\_info*)¶
:   Plot fit of 1d data.

    Parameters
    :   - **x** (*array*) – List of x values.
        - **y** (*array*) – List of y values.
        - **y\_fit** (*array*) – List of y values from the fit.
        - **lm\_result** (*object MinimizerResult*) – Results of lm minimization. Includes data such as status and error messages, fit statistics,
          and the updated (i.e., best-fit) parameters themselves in the params attribute.
        - **x\_label** (*str*) – Exact name of the x sensor, as it appears in the stamps.
        - **y\_label** (*str*) – Exact name of the y sensor, as it appears in the stamps.
        - **time\_str** (*str*) – Starting/ending dates of the scan.
        - **nxs\_name** (*str*) – Nexus name, e.g. SIRIUS\_2020\_03\_12\_0756.nxs.
        - **absorbers** (*str*) – Text to display which absorber was used.
        - **is\_print\_info** (*bool*) – Verbose mode.

    Returns
    :   **fig** – The figure to be saved in pdf.

    Return type
    :   matplotlib figure

lib.backend.data\_1d.plot\_gaussian\_fit(*x*, *y*, *y\_fit*, *lm\_result*, *x\_label*, *y\_label*, *time\_str*, *nxs\_name*, *absorbers*, *is\_print\_info*)¶
:   Plot fit of 1d data.

    Parameters
    :   - **x** (*array*) – List of x values.
        - **y** (*array*) – List of y values.
        - **y\_fit** (*array*) – List of y values from the fit.
        - **lm\_result** (*object MinimizerResult*) – Results of lm minimization. Includes data such as status and error messages, fit statistics,
          and the updated (i.e., best-fit) parameters themselves in the params attribute.
        - **x\_label** (*str*) – Exact name of the x sensor, as it appears in the stamps.
        - **y\_label** (*str*) – Exact name of the y sensor, as it appears in the stamps.
        - **time\_str** (*str*) – Starting/ending dates of the scan.
        - **nxs\_name** (*str*) – Nexus name, e.g. SIRIUS\_2020\_03\_12\_0756.nxs.
        - **absorbers** (*str*) – Text to display which absorber was used.
        - **is\_print\_info** (*bool*) – Verbose mode.

    Returns
    :   **fig** – The figure to be saved in pdf.

    Return type
    :   matplotlib figure

lib.backend.data\_1d.process\_data\_1d(*nxs\_name*, *path\_to\_nxs\_dir*, *x\_label*, *y\_label*, *is\_logx=False*, *is\_logy=False*, *absorbers=''*, *path\_to\_save\_dir=''*, *is\_print\_stamps=False*, *is\_plot=False*, *is\_save=False*, *is\_print\_info=False*)¶
:   Call functions for extracting, plotting, and saving 1d data.

    Parameters
    :   - **nxs\_name** (*str*) – Nexus name, e.g. SIRIUS\_2020\_03\_12\_0756.nxs.
        - **path\_to\_nxs\_dir** (*str*) – Path to the nexus files directory, e.g. user/.
        - **x\_label** (*str*) – Exact name of the x sensor, as it appears in the stamps.
        - **y\_label** (*str*) – Exact name of the y sensor, as it appears in the stamps.
        - **is\_logx** (*bool**,* *optional*) – Log scale on the x axis.
        - **is\_logy** (*bool**,* *optional*) – Log scale on the y axis.
        - **absorbers** (*str**,* *optional*) – Text to display which absorber was used.
        - **path\_to\_save\_dir** (*str*) – Path to the directory where the treated files will be saved.
        - **is\_print\_stamps** (*bool**,* *optional*) – Print the list of sensors contained in the nexus file.
        - **is\_plot** (*bool**,* *optional*) – Plot the data.
        - **is\_save** (*bool**,* *optional*) – Save the results.
        - **is\_print\_info** (*bool**,* *optional*) – Verbose mode.

    Returns
    :   - **x** (*array*) – List of x values.
        - **y** (*array*) – List of y values.

lib.backend.data\_1d.process\_energy\_calib(*nxs\_name*, *path\_to\_nxs\_dir*, *xas\_energy\_min*, *xas\_energy\_max*, *xas\_energy\_shift*, *xas\_signal\_label*, *xas\_norm\_label*, *xas\_standard\_name*, *energy\_current*, *bragg\_current*, *path\_to\_xas\_dir*, *path\_to\_save\_dir=''*, *is\_save=False*)¶
:   Call functions for extracting, plotting, and saving energy calibration
    with XAS standards.

    Parameters
    :   - **nxs\_name** (*str*) – Nexus name, e.g. SIRIUS\_2020\_03\_12\_0756.nxs.
        - **path\_to\_nxs\_dir** (*str*) – Path to the nexus files directory, e.g. user/.
        - **xas\_energy\_min** (*float*) – Energy minimum for the plot in keV.
        - **xas\_energy\_max** (*float*) – Energy maximum for the plot in keV.
        - **xas\_energy\_shift** (*float*) – Energy shift between the measurement and the standard in keV.
        - **xas\_signal\_label** (*str*) – Exact name of the sensor used for signal, as it appears in the stamps.
        - **xas\_norm\_label** (*str*) – Exact name of the sensor used for normalization, as it appears in the stamps.
        - **xas\_standard\_name** (*str*) – Chosen xas standard file, e.g. ‘XAS\_ref\_Ni.txt’.
        - **energy\_current** (*float*) – Current value of the DCM energy in keV.
        - **bragg\_current** (*float*) – Current value of the bragg angle in deg.
        - **path\_to\_xas\_dir** (*str*) – Path to the folder containing the standards.
        - **path\_to\_save\_dir** (*str*) – Path to the directory where the treated files will be saved.
        - **is\_save** (*bool**,* *optional*) – Save the results.

lib.backend.data\_1d.process\_erf\_fit(*nxs\_name*, *path\_to\_nxs\_dir*, *x\_label*, *y\_label*, *params\_init=None*, *absorbers=''*, *path\_to\_save\_dir=''*, *is\_print\_stamps=False*, *is\_plot=False*, *is\_save=False*, *is\_print\_info=False*)¶
:   Call functions for extracting, fitting with an error function, plotting, and saving 1d data.

    Parameters
    :   - **nxs\_name** (*str*) – Nexus name, e.g. SIRIUS\_2020\_03\_12\_0756.nxs.
        - **path\_to\_nxs\_dir** (*str*) – Path to the nexus files directory, e.g. user/.
        - **x\_label** (*str*) – Exact name of the x sensor, as it appears in the stamps.
        - **y\_label** (*str*) – Exact name of the y sensor, as it appears in the stamps.
        - **params\_init** (*dict* *or* *None**,* *optional*) – Dictionnary of parameters for initial guesses and limits.
          All the parameters should be given. For example:
          params\_init = {‘cst’:{‘init’:0, ‘min’:-1, ‘max’:1},
          ‘mu’:{‘init’:0, ‘min’:-1, ‘max’:1},
          ‘sigma’:{‘init’:0.2, ‘min’:0., ‘max’:1},
          ‘amplitude’:{‘init’:10., ‘min’:-10, ‘max’:20},
          ‘sign’:{‘value’:-1}}
        - **absorbers** (*str**,* *optional*) – Text to display which absorber was used.
        - **path\_to\_save\_dir** (*str*) – Path to the directory where the treated files will be saved.
        - **is\_print\_stamps** (*bool**,* *optional*) – Print the list of sensors contained in the nexus file.
        - **is\_plot** (*bool**,* *optional*) – Plot the data.
        - **is\_save** (*bool**,* *optional*) – Save the results.
        - **is\_print\_info** (*bool**,* *optional*) – Verbose mode.

    Returns
    :   - **x** (*array*) – List of x values.
        - **y** (*array*) – List of y values.
        - **y\_fit** (*array*) – List of y values from the fit.
        - **lm\_result** (*object MinimizerResult*) – Results of lm minimization. Includes data such as status and error messages, fit statistics,
          and the updated (i.e., best-fit) parameters themselves in the params attribute.

lib.backend.data\_1d.process\_gaussian\_fit(*nxs\_name*, *path\_to\_nxs\_dir*, *x\_label*, *y\_label*, *params\_init=None*, *absorbers=''*, *path\_to\_save\_dir=''*, *is\_print\_stamps=False*, *is\_plot=False*, *is\_save=False*, *is\_print\_info=False*)¶
:   Call functions for extracting, fitting with a gaussian, plotting, and saving 1d data.

    Parameters
    :   - **nxs\_name** (*str*) – Nexus name, e.g. SIRIUS\_2020\_03\_12\_0756.nxs.
        - **path\_to\_nxs\_dir** (*str*) – Path to the nexus files directory, e.g. user/.
        - **x\_label** (*str*) – Exact name of the x sensor, as it appears in the stamps.
        - **y\_label** (*str*) – Exact name of the y sensor, as it appears in the stamps.
        - **params\_init** (*dict* *or* *None**,* *optional*) – Dictionnary of parameters for initial guesses and limits.
          All the parameters should be given. For example:
          params\_init = {‘cst’:{‘init’:0, ‘min’:-1, ‘max’:1},
          ‘linear\_coeff’:{‘init’:0, ‘min’:-1, ‘max’:1},
          ‘mu’:{‘init’:0, ‘min’:-1, ‘max’:1},
          ‘sigma’:{‘init’:0.2, ‘min’:0., ‘max’:1},
          ‘amplitude’:{‘init’:10., ‘min’:-10, ‘max’:20}}
        - **absorbers** (*str**,* *optional*) – Text to display which absorber was used.
        - **path\_to\_save\_dir** (*str*) – Path to the directory where the treated files will be saved.
        - **is\_print\_stamps** (*bool**,* *optional*) – Print the list of sensors contained in the nexus file.
        - **is\_plot** (*bool**,* *optional*) – Plot the data.
        - **is\_save** (*bool**,* *optional*) – Save the results.
        - **is\_print\_info** (*bool**,* *optional*) – Verbose mode.

    Returns
    :   - **x** (*array*) – List of x values.
        - **y** (*array*) – List of y values.
        - **y\_fit** (*array*) – List of y values from the fit.
        - **lm\_result** (*object MinimizerResult*) – Results of lm minimization. Includes data such as status and error messages, fit statistics,
          and the updated (i.e., best-fit) parameters themselves in the params attribute.

lib.backend.data\_1d.residuals\_erf\_function(*params*, *x*, *y*)¶
:   Return the residuals of the error function.

lib.backend.data\_1d.residuals\_gaussian\_function(*params*, *x*, *y*)¶
:   Return the residuals of the gaussian function.

lib.backend.data\_1d.save\_data\_1d(*nxs\_name*, *stamps\_0d*, *data\_0d*, *fig*, *path\_to\_save\_dir*, *is\_print\_info*)¶
:   Save 1D data.

    XXX.dat : the value of each sensor at each point of the scan.
    XXX.pdf : the figure in pdf.

    Parameters
    :   - **nxs\_name** (*str*) – Nexus name, e.g. SIRIUS\_2020\_03\_12\_0756.nxs.
        - **stamps\_0d** (*array*) – Aliases of each 0D sensor in the scan.
        - **data\_0d** (*array*) – Values of each 0D sensor in the scan.
        - **fig** (*None* *or* *matplotlib figure*) – The figure to be saved in pdf. Pass None if not wanted.
        - **path\_to\_save\_dir** (*str*) – Path to the directory where the treated files will be saved.
        - **is\_print\_info** (*bool*) – Verbose mode.

lib.backend.data\_1d.save\_fit\_result(*nxs\_name*, *x*, *y*, *y\_fit*, *lm\_result*, *x\_label*, *y\_label*, *fig*, *path\_to\_save\_dir*, *is\_print\_info*)¶
:   Save 1D data and fit results.

    XXX.dat : the value of each sensor at each point of the scan.
    XXX.pdf : the figure in pdf.
    XXX\_fit\_FUNCTION\_report.dat : the lmfit report.
    XXX\_fit\_FUNCTION\_result.dat : the fitted data x, y, y\_fit.

    Parameters
    :   - **nxs\_name** (*str*) – Nexus name, e.g. SIRIUS\_2020\_03\_12\_0756.nxs.
        - **x** (*array*) – List of x values.
        - **y** (*array*) – List of y values.
        - **y\_fit** (*array*) – List of y values from the fit.
        - **lm\_result** (*object MinimizerResult*) – Results of lm minimization. Includes data such as status and error messages, fit statistics,
          and the updated (i.e., best-fit) parameters themselves in the params attribute.
        - **x\_label** (*str*) – Exact name of the x sensor, as it appears in the stamps.
        - **y\_label** (*str*) – Exact name of the y sensor, as it appears in the stamps.
        - **fig** (*None* *or* *matplotlib figure*) – The figure to be saved in pdf. Pass None if not wanted.
        - **path\_to\_save\_dir** (*str*) – Path to the directory where the treated files will be saved.
        - **is\_print\_info** (*bool*) – Verbose mode.

## lib.backend.gixd module¶

Library for GIXD.

lib.backend.gixd.bin\_matrix\_vertical(*mat*, *bin\_size=10*)¶
:   Bin a matrix along the vertical axis.

    Parameters
    :   - **mat** (*array*) – Numpy array with the matrix to bin.
        - **bin\_size** (*int**,* *optional*) – Size in pixels of the vertical binning.

    Returns
    :   **(channels\_binned, mat\_binned)** – Channels and matrix after binning.

    Return type
    :   tupple of arrays

lib.backend.gixd.calib\_thetaz(*gamma\_channel*, *is\_plot=True*)¶
:   Fit and plot gamma vs channel for the calibration of thetaz.

    Parameters
    :   - **gamma\_channel** (*array*) – Numpy array containing the values of each gamma and corresponding channel.
          For example :
          np.array([
          [0, 970],
          [-1, 899],
          [-2, 827]])
        - **is\_plot** (*bool**,* *optional*) – Plot the fit.

    Returns
    :   **thetaz\_factor** – Factor for conversion from channel to radian in the vertical direction (rad/channel).

    Return type
    :   float

lib.backend.gixd.extract\_gixd\_scan(*nxs\_name*, *path\_to\_nxs\_dir*, *channel0*, *thetaz\_factor*, *wavelength*, *thetac*, *bin\_size*, *is\_compute\_qz*, *is\_force\_gamma*, *fgamma*, *roi\_soller*, *is\_print\_stamps*, *is\_print\_info*)¶
:   Extract the nexus file and return useful quantities for GIXD.

    Parameters
    :   - **nxs\_name** (*str*) – Nexus name, e.g. SIRIUS\_2020\_03\_12\_0756.nxs.
        - **path\_to\_nxs\_dir** (*str*) – Path to the nexus files directory, e.g. user/.
        - **channel0** (*int*) – Vertical channel corresponding to the Vineyard’s peak.
        - **thetaz\_factor** (*float*) – Factor for conversion from channel to radian in the vertical direction (rad/channel).
        - **wavelength** (*float*) – Wavelength in nm.
        - **thetac** (*float*) – Critical angle in rad.
        - **bin\_size** (*int*) – Size in pixels of the vertical binning (along qz).
        - **is\_compute\_qz** (*bool*) – Convert pixels to qz in the vertical direction.
        - **is\_force\_gamma** (*bool*) – Force gamma to be equal to the value of fgamma.
        - **fgamma** (*float*) – Value of gamma (deg) to be used if is\_force\_gamma is set to True
          or if gamma is absent from the sensor list.
        - **roi\_soller** (*list of int*) – ROI of the Soller’s slits.
        - **is\_print\_stamps** (*bool*) – Print the list of sensors contained in the nexus file.
        - **is\_print\_info** (*bool*) – Verbose mode.

    Returns
    :   - **x** (*array*) – Either qxy (nm^-1) values, if qxy is available in the list of sensors,
          or delta (deg) if not.
        - **y** (*array*) – Either qz (nm^-1) values, if is\_compute\_qz is True,
          or vertical channels if not.
        - **x\_label** (*str*) – ‘qxy’ or ‘delta’ (useful for plot).
        - **y\_label** (*str*) – ‘qz’ or ‘channels’ (useful for plot).
        - **time\_str** (*str*) – Starting/ending dates of the scan.
        - *column\_x* – Column corresponding to the x values in stamps\_0d (useful for save).
        - **integ\_rod** (*array*) – Rods integrated over the whole vertical axis of the detector.
        - **integ\_rod\_top** (*array*) – Rods integrated over the top half vertical axis of the detector.
        - **integ\_rod\_bottom** (*array*) – Rods integrated over the bottom half vertical axis of the detector.
        - **integ\_rod\_first\_quarter** (*array*) – Rods integrated over the bottom quarter vertical axis of the detector.
        - **mat** (*array*) – Matrix with each line corresponding to a position of delta.
        - **mat\_binned** (*array*) – Matrix after vertical binning.
        - **channels\_binned** (*array*) – Channels after vertical binning.
        - **mean\_pi** (*float or None*) – Average of the surface pressure (mN/m) over the scan (None if pressure not found).
        - **mean\_area** (*float or None*) – Average of the area per molecule (nm^2) over the scan (None if area not found).
        - **mean\_gamma** (*float or None*) – Average of gamma (deg) over the scan (None if gamma not found).
        - **stamps\_0d** (*array*) – Aliases of each 0D sensor in the scan.
        - **data\_0d** (*array*) – Values of each 0D sensor in the scan.

    Raises
    :   - **FileNotFoundError** – If a file or a folder is missing.
        - **SystemExit****(****'Could not open Nexus file.'****)****)** – If the Nexus file cannot be accessed.
        - **SystemExit****(****'delta** **or** **qxy not found.'****)** – If delta and qxy are not found in the sensor list.
        - **SystemExit****(****'gamma not found.'****)** – If gamma is not found in the sensor list and is\_compute\_qz is True.
        - **SystemExit****(****'pilatus not found.'****)** – If pilatus is not found in the sensor list.

lib.backend.gixd.plot\_gixd\_scan(*x*, *y*, *x\_label*, *y\_label*, *time\_str*, *integ\_rod*, *integ\_rod\_top*, *integ\_rod\_bottom*, *integ\_rod\_first\_quarter*, *mat\_binned*, *mean\_pi*, *mean\_gamma*, *nxs\_name*, *absorbers*, *is\_gixd\_logx*, *is\_gixd\_logy*, *is\_gixd\_logz*, *nb\_levels*, *map\_gixd*)¶
:   Plot GIXD data.

    Parameters
    :   - **x** (*array*) – Either qxy (nm^-1) values, if qxy is available in the list of sensors,
          or delta (deg) if not.
        - **y** (*array*) – Either qz (nm^-1) values, if is\_compute\_qz is True,
          or vertical channels if not.
        - **x\_label** (*str*) – ‘qxy’ or ‘delta’ (useful for plot).
        - **y\_label** (*str*) – ‘qz’ or ‘channels’ (useful for plot).
        - **time\_str** (*str*) – Starting/ending dates of the scan.
        - **column\_x** – Column corresponding to the x values in stamps\_0d (useful for save).
        - **integ\_rod** (*array*) – Rods integrated over the whole vertical axis of the detector.
        - **integ\_rod\_top** (*array*) – Rods integrated over the top half vertical axis of the detector.
        - **integ\_rod\_bottom** (*array*) – Rods integrated over the bottom half vertical axis of the detector.
        - **integ\_rod\_first\_quarter** (*array*) – Rods integrated over the bottom quarter vertical axis of the detector.
        - **mat\_binned** (*array*) – Matrix after vertical binning.
        - **mean\_pi** (*float* *or* *None*) – Average of the surface pressure (mN/m) over the scan (None if pressure not found).
        - **mean\_gamma** (*float* *or* *None*) – Average of gamma (deg) over the scan (None if gamma not found).
        - **nxs\_name** (*str*) – Nexus name, e.g. SIRIUS\_2020\_03\_12\_0756.nxs.
        - **absorbers** (*str*) – Text to display which absorber was used.
        - **is\_gixd\_logx** (*bool*) – Log scale on the x axis of the integrated profile.
        - **is\_gixd\_logy** (*bool*) – Log scale on the y axis of the integrated profile.
        - **is\_gixd\_logz** (*bool*) – Log scale on the color scale of the image.
        - **nb\_levels** (*int*) – Number of color levels for the image display.
        - **map\_gixd** (*str*) – Colormap of the image.

    Returns
    :   **fig** – The figure to be saved in pdf.

    Return type
    :   matplotlib figure

lib.backend.gixd.plot\_vineyard(*integ\_over\_qxy*, *channel0*)¶
:   Plot the profile integrated over qxy that was used to find the Vineyard’s peak.

    Parameters
    :   - **integ\_over\_qxy** (*array*) – GIXD image integrated over the qxy axis.
        - **channel0** (*int*) – Vertical channel corresponding to the Vineyard’s peak.

lib.backend.gixd.process\_gixd\_scan(*nxs\_name*, *path\_to\_nxs\_dir*, *channel0*, *thetaz\_factor*, *wavelength*, *thetac*, *bin\_size*, *is\_compute\_qz*, *is\_force\_gamma=False*, *fgamma=0.0*, *absorbers=''*, *is\_gixd\_logx=False*, *is\_gixd\_logy=False*, *is\_gixd\_logz=False*, *nb\_levels=50*, *map\_gixd='jet'*, *path\_to\_save\_dir=''*, *list\_moy\_to\_create=[10, 20, 40]*, *roi\_soller=[510, 350, 130, 692]*, *is\_print\_stamps=False*, *is\_plot=False*, *is\_save=False*, *is\_print\_info=False*)¶
:   Call functions for extracting, plotting, and saving a GIXD scan.

    Parameters
    :   - **nxs\_name** (*str*) – Nexus name, e.g. SIRIUS\_2020\_03\_12\_0756.nxs.
        - **path\_to\_nxs\_dir** (*str*) – Path to the nexus files directory, e.g. user/.
        - **channel0** (*int*) – Vertical channel corresponding to the Vineyard’s peak.
        - **thetaz\_factor** (*float*) – Factor for conversion from channel to radian in the vertical direction (rad/channel).
        - **wavelength** (*float*) – Wavelength in nm.
        - **thetac** (*float*) – Critical angle in rad.
        - **bin\_size** (*int*) – Size in pixels of the vertical binning (along qz).
        - **is\_compute\_qz** (*bool*) – Convert pixels to qz in the vertical direction.
        - **is\_force\_gamma** (*bool**,* *optional*) – Force gamma to be equal to the value of fgamma.
        - **fgamma** (*float**,* *optional*) – Value of gamma (deg) to be used if is\_force\_gamma is set to True
          or if gamma is absent from the sensor list.
        - **absorbers** (*str**,* *optional*) – Text to display which absorber was used.
        - **is\_gixd\_logx** (*bool**,* *optional*) – Log scale on the x axis of the integrated profile.
        - **is\_gixd\_logy** (*bool**,* *optional*) – Log scale on the y axis of the integrated profile.
        - **is\_gixd\_logz** (*bool**,* *optional*) – Log scale on the color scale of the image.
        - **nb\_levels** (*int**,* *optional*) – Number of color levels for the image display.
        - **map\_gixd** (*str**,* *optional*) – Colormap of the image.
        - **path\_to\_save\_dir** (*str**,* *optional*) – Path to the directory where the treated files will be saved.
        - **list\_moy\_to\_create** (*list of int**,* *optional*) – Bin sizes to be used, e.g. [10, 20, 40].
        - **roi\_soller** (*list of int**,* *optional*) – ROI of the Soller’s slits.
        - **is\_print\_stamps** (*bool**,* *optional*) – Print the list of sensors contained in the nexus file.
        - **is\_plot** (*bool**,* *optional*) – Plot the 2D GIXD image and the integrated profiles.
        - **is\_save** (*bool**,* *optional*) – Save the results.
        - **is\_print\_info** (*bool**,* *optional*) – Verbose mode.

    Returns
    :   - **x** (*array*) – Either qxy (nm^-1) values, if qxy is available in the list of sensors,
          or delta (deg) if not.
        - **y** (*array*) – Either qz (nm^-1) values, if is\_compute\_qz is True,
          or vertical channels if not.
        - **integ\_rod** (*array*) – Rods integrated over the whole vertical axis of the detector.
        - **integ\_rod\_top** (*array*) – Rods integrated over the top half vertical axis of the detector.
        - **integ\_rod\_bottom** (*array*) – Rods integrated over the bottom half vertical axis of the detector.
        - **integ\_rod\_first\_quarter** (*array*) – Rods integrated over the bottom quarter vertical axis of the detector.
        - **mat** (*array*) – Each line corresponds to a position of delta.
          It is the matrix corresponding to the image displayed in plot.
        - **mat\_binned** (*array*) – Matrix after vertical binning.
        - **channels\_binned** (*array*) – Channels after vertical binning.
        - **mean\_pi** (*float or None*) – Average of the surface pressure (mN/m) over the scan (None if pressure not found).
        - **mean\_area** (*float or None*) – Average of the area per molecule (nm^2) over the scan (None if area not found).
        - **mean\_gamma** (*float or None*) – Average of gamma (deg) over the scan (None if gamma not found).

lib.backend.gixd.process\_vineyard(*nxs\_name*, *path\_to\_nxs\_dir*, *bin\_size=10*, *is\_force\_gamma=False*, *fgamma=0.0*, *is\_gixd\_logx=False*, *is\_gixd\_logy=False*, *is\_gixd\_logz=False*, *nb\_levels=50*, *map\_gixd='jet'*, *roi\_soller=[510, 350, 130, 692]*, *is\_print\_stamps=False*, *is\_plot=False*, *is\_print\_info=False*)¶
:   Extract the nexus file, plot and return the channel of the Vineyard’s peak.

    Parameters
    :   - **nxs\_name** (*str*) – Nexus name, e.g. SIRIUS\_2020\_03\_12\_0756.nxs.
        - **path\_to\_nxs\_dir** (*str*) – Path to the nexus files directory, e.g. user/.
        - **bin\_size** (*int**,* *optional*) – Size in pixels of the vertical binning (along qz).
        - **is\_force\_gamma** (*bool**,* *optional*) – Force gamma to be equal to the value of fgamma.
        - **fgamma** (*float**,* *optional*) – Value of gamma (deg) to be used if is\_force\_gamma is set to True
          or if gamma is absent from the sensor list.
        - **is\_gixd\_logx** (*bool**,* *optional*) – Log scale on the x axis of the integrated profile.
        - **is\_gixd\_logy** (*bool**,* *optional*) – Log scale on the y axis of the integrated profile.
        - **is\_gixd\_logz** (*bool**,* *optional*) – Log scale on the color scale of the image.
        - **nb\_levels** (*int**,* *optional*) – Number of color levels for the image display.
        - **map\_gixd** (*str**,* *optional*) – Colormap of the image.
        - **roi\_soller** (*list of int**,* *optional*) – ROI of the Soller’s slits.
        - **is\_print\_stamps** (*bool**,* *optional*) – Print the list of sensors contained in the nexus file.
        - **is\_plot** (*bool**,* *optional*) – Plot the 2D GIXD image and the integrated profiles.
        - **is\_print\_info** (*bool**,* *optional*) – Verbose mode.

    Returns
    :   **channel0** – Vertical channel corresponding to the Vineyard’s peak.

    Return type
    :   int

lib.backend.gixd.save\_gixd\_scan(*x*, *integ\_rod*, *integ\_rod\_top*, *integ\_rod\_bottom*, *integ\_rod\_first\_quarter*, *mat*, *list\_moy\_to\_create*, *mean\_gamma*, *column\_x*, *channel0*, *thetaz\_factor*, *wavelength*, *thetac*, *nxs\_name*, *stamps\_0d*, *data\_0d*, *fig*, *path\_to\_save\_dir*, *is\_compute\_qz*, *is\_print\_info*)¶
:   Save GIXD data.

    XXX\_1D.mat: the matrix corresponding to the image displayed.
    Each line corresponds to a position of delta.

    XXX\_1D.dat: the value of each sensor at each point of the scan.
    It contains also integration along qz:

    > - QzIntegrated : over the whole detector,
    > - QzIntegratedTop : over its top half,
    > - QzIntegratedBottom : over its bottom half,
    > - QzIntegratedBottomQuarter : over its bottom quarter.

    XXX.pdf : the figure in pdf.

    Binned data:
    :   - XXX\_1D.matNN : binning of the matrix, with NN the number of points per bin.
        - XXX\_1D\_qz.datNN : to convert bin number to qz in XXX\_1D.matNN.
        - XXX\_1D.moyNN : a more convenient way to represent the binned matrices with a 3 columns (qxy, qz, intensity) display.

    Parameters
    :   - **x** (*array*) – Either qxy (nm^-1) values, if qxy is available in the list of sensors,
          or delta (deg) if not.
        - **integ\_rod** (*array*) – Rods integrated over the whole vertical axis of the detector.
        - **integ\_rod\_top** (*array*) – Rods integrated over the top half vertical axis of the detector.
        - **integ\_rod\_bottom** (*array*) – Rods integrated over the bottom half vertical axis of the detector.
        - **integ\_rod\_first\_quarter** (*array*) – Rods integrated over the bottom quarter vertical axis of the detector.
        - **mat** (*array*) – Matrix with each line corresponding to a position of delta.
        - **list\_moy\_to\_create** (*list of int**,* *optional*) – Bin sizes to be used, e.g. [10, 20, 40].
        - **mean\_gamma** (*float* *or* *None*) – Average of gamma (deg) over the scan (None if gamma not found).
        - **column\_x** (*int*) – Column corresponding to the x values in stamps\_0d (useful for save).
        - **channel0** (*int*) – Vertical channel corresponding to the Vineyard’s peak.
        - **thetaz\_factor** (*float*) – Factor for conversion from channel to radian in the vertical direction (rad/channel).
        - **wavelength** (*float*) – Wavelength in nm.
        - **thetac** (*float*) – Critical angle in rad.
        - **nxs\_name** (*str*) – Nexus name, e.g. SIRIUS\_2020\_03\_12\_0756.nxs.
        - **stamps\_0d** (*array*) – Aliases of each 0D sensor in the scan.
        - **data\_0d** (*array*) – Values of each 0D sensor in the scan.
        - **fig** (*None* *or* *matplotlib figure*) – The figure to be saved in pdf. Pass None if not wanted.
        - **path\_to\_save\_dir** (*str*) – Path to the directory where the treated files will be saved.
        - **is\_compute\_qz** (*bool*) – Convert pixels to qz in the vertical direction.
        - **is\_print\_info** (*bool*) – Verbose mode.

## lib.backend.gixs module¶

Library for GIXS.

lib.backend.gixs.extract\_gixs\_scan(*nxs\_name*, *path\_to\_nxs\_dir*, *wavelength*, *distance\_detec*, *pixel\_poni\_x*, *pixel\_poni\_y*, *pixel\_size*, *is\_force\_gamma*, *is\_force\_delta*, *is\_force\_thetai*, *fgamma*, *fdelta*, *fthetai*, *is\_print\_stamps*, *is\_print\_info*)¶
:   Extract the nexus file and return useful quantities for GIXS.

    Parameters
    :   - **nxs\_name** (*str*) – Nexus name, e.g. SIRIUS\_2020\_03\_12\_0756.nxs.
        - **path\_to\_nxs\_dir** (*str*) – Path to the nexus files directory, e.g. user/.
        - **wavelength** (*float*) – Wavelength in nm.
        - **distance\_detec** (*float*) – Distance between the detector and the center of the sample in mm.
        - **pixel\_poni\_x** (*float*) – horizontal coordinate of the Point Of Normal Incidence in pixels. Measured on the
          direct beam at delta=0 and gamma=0.
        - **pixel\_poni\_y** (*float*) – vertical coordinate of the Point Of Normal Incidence in pixels. Measured on the
          direct beam at delta=0 and gamma=0.
        - **pixel\_size** (*float*) – pixel size in mm.
        - **is\_force\_gamma** (*bool*) – Force gamma to be equal to the value of fgamma.
        - **is\_force\_delta** (*bool*) – Force delta to be equal to the value of fdelta.
        - **is\_force\_thetai** (*bool*) – Force thetai to be equal to the value of fthetai.
        - **fgamma** (*float*) – Value of gamma (deg) to be used if is\_force\_gamma is set to True
          or if gamma is absent from the sensor list.
        - **fdelta** (*float*) – Value of delta (deg) to be used if is\_force\_delta is set to True
          or if delta is absent from the sensor list.
        - **fthetai** (*float*) – Value of thetai (deg) to be used if is\_force\_thetai is set to True
          or if alphax is absent from the sensor list.
        - **is\_print\_stamps** (*bool*) – Print the list of sensors contained in the nexus file.
        - **is\_print\_info** (*bool*) – Verbose mode.

    Returns
    :   - **images** (*array*) – Array of individual Pilatus images.
        - **images\_sum** (*array*) – Pilatus images integrated over the scan.
        - **qxy\_grid** (*array*) – 2D grid of qxy for 2D plots.
        - **qz\_grid** (*array*) – 2D grid of qz for 2D plots.
        - **integ\_qxy** (*array*) – Profile integrated along the horizontal axis.
        - **integ\_qz** (*array*) – Profile integrated along the vertical axis.
        - **qxy\_list** (*array*) – List of qxy in the profile.
        - **qz\_list** (*array*) – List of qz in the profile.
        - **gamma\_str** (*str*) – Label with value of gamma.
        - **delta\_str** (*str*) – Label with value of delta.
        - **thetai\_str** (*str*) – Label with value of thetai.
        - **time\_str** (*str*) – Starting/ending dates of the scan.
        - **stamps\_0d** (*array*) – Aliases of each 0D sensor in the scan.
        - **data\_0d** (*array*) – Values of each 0D sensor in the scan.

    Raises
    :   - **FileNotFoundError** – If a file or a folder is missing.
        - **SystemExit****(****'Could not open Nexus file.'****)****)** – If the Nexus file cannot be accessed.
        - **SystemExit****(****'pilatus not found.'****)** – If pilatus is not found in the sensor list.

lib.backend.gixs.plot\_gixs\_scan(*images\_sum*, *qxy\_grid*, *qz\_grid*, *integ\_qxy*, *integ\_qz*, *qxy\_list*, *qz\_list*, *nxs\_name*, *absorbers*, *is\_gixs\_logz*, *map\_gixs*, *qxy\_min*, *qxy\_max*, *qz\_min*, *qz\_max*, *gamma\_str*, *delta\_str*, *thetai\_str*, *time\_str*)¶
:   Plot GIXS data.

    Parameters
    :   - **images\_sum** (*array*) – Pilatus images integrated over the scan.
        - **qxy\_grid** (*array*) – 2D grid of qxy for 2D plots.
        - **qz\_grid** (*array*) – 2D grid of qz for 2D plots.
        - **integ\_qxy** (*array*) – Profile integrated along the horizontal axis.
        - **integ\_qz** (*array*) – Profile integrated along the vertical axis.
        - **qxy\_list** (*array*) – List of qxy in the profile.
        - **qz\_list** (*array*) – List of qz in the profile.
        - **nxs\_name** (*str*) – Nexus name, e.g. SIRIUS\_2020\_03\_12\_0756.nxs.
        - **absorbers** (*str*) – Text to display which absorber was used.
        - **is\_gixs\_logz** (*bool*) – Log scale on the color scale of the image.
        - **map\_gixs** (*str*) – Colormap of the image.
        - **qxy\_min** (*float*) – Min limit of the vertical profile plot (integrated over the horizontal axis).
        - **qxy\_max** (*float*) – Max limit of the vertical profile plot (integrated over the horizontal axis).
        - **qz\_min** (*float*) – Min limit of the horizontal profile plot (integrated over the vertical axis).
        - **qz\_max** (*float*) – Max limit of the horizontal profile plot (integrated over the vertical axis).
        - **gamma\_str** (*str*) – Label with value of gamma.
        - **delta\_str** (*str*) – Label with value of delta.
        - **thetai\_str** (*str*) – Label with value of thetai.
        - **time\_str** (*str*) – Starting/ending dates of the scan.

    Returns
    :   **fig** – The figure to be saved in pdf.

    Return type
    :   matplotlib figure

lib.backend.gixs.process\_gixs\_scan(*nxs\_name*, *path\_to\_nxs\_dir*, *wavelength*, *distance\_detec*, *pixel\_poni\_x*, *pixel\_poni\_y*, *pixel\_size*, *is\_force\_gamma=False*, *is\_force\_delta=False*, *is\_force\_thetai=False*, *fgamma=0.0*, *fdelta=0.0*, *fthetai=0.0*, *qxy\_min=0.0*, *qxy\_max=1.0*, *qz\_min=0.0*, *qz\_max=1.0*, *absorbers=''*, *is\_gixs\_logz=True*, *map\_gixs='viridis'*, *path\_to\_save\_dir=''*, *is\_print\_stamps=False*, *is\_plot=False*, *is\_save\_sum=False*, *is\_save\_each=False*, *is\_print\_info=False*)¶
:   Call functions for extracting, plotting, and saving a GIXS scan.

    Parameters
    :   - **nxs\_name** (*str*) – Nexus name, e.g. SIRIUS\_2020\_03\_12\_0756.nxs.
        - **path\_to\_nxs\_dir** (*str*) – Path to the nexus files directory, e.g. user/.
        - **wavelength** (*float*) – Wavelength in nm.
        - **distance\_detec** (*float*) – Distance between the detector and the center of the sample in mm.
        - **pixel\_poni\_x** (*float*) – horizontal coordinate of the Point Of Normal Incidence in pixels. Measured on the
          direct beam at delta=0 and gamma=0.
        - **pixel\_poni\_y** (*float*) – vertical coordinate of the Point Of Normal Incidence in pixels. Measured on the
          direct beam at delta=0 and gamma=0.
        - **pixel\_size** (*float*) – pixel size in mm.
        - **is\_force\_gamma** (*bool**,* *optional*) – Force gamma to be equal to the value of fgamma.
        - **is\_force\_delta** (*bool**,* *optional*) – Force delta to be equal to the value of fdelta.
        - **is\_force\_thetai** (*bool**,* *optional*) – Force thetai to be equal to the value of fthetai.
        - **fgamma** (*float**,* *optional*) – Value of gamma (deg) to be used if is\_force\_gamma is set to True
          or if gamma is absent from the sensor list.
        - **fdelta** (*float**,* *optional*) – Value of delta (deg) to be used if is\_force\_delta is set to True
          or if delta is absent from the sensor list.
        - **fthetai** (*float**,* *optional*) – Value of thetai (deg) to be used if is\_force\_thetai is set to True
          or if alphax is absent from the sensor list.
        - **qxy\_min** (*float**,* *optional*) – Min limit of the vertical profile plot (integrated over the horizontal axis).
        - **qxy\_max** (*float**,* *optional*) – Max limit of the vertical profile plot (integrated over the horizontal axis).
        - **qz\_min** (*float**,* *optional*) – Min limit of the horizontal profile plot (integrated over the vertical axis).
        - **qz\_max** (*float**,* *optional*) – Max limit of the horizontal profile plot (integrated over the vertical axis).
        - **absorbers** (*str**,* *optional*) – Text to display which absorber was used.
        - **is\_gixs\_logz** (*bool**,* *optional*) – Log scale on the color scale of the image.
        - **map\_gixs** (*str**,* *optional*) – Colormap of the image.
        - **path\_to\_save\_dir** (*str**,* *optional*) – Path to the directory where the treated files will be saved.
        - **is\_print\_stamps** (*bool**,* *optional*) – Print the list of sensors contained in the nexus file.
        - **is\_plot** (*bool**,* *optional*) – Plot the 2D GIXS image and the integrated profiles.
        - **is\_save\_sum** (*bool**,* *optional*) – Save the sum of the images.
        - **is\_save\_each** (*bool**,* *optional*) – Save each individual image and the sum.
        - **is\_print\_info** (*bool**,* *optional*) – Verbose mode.

    Returns
    :   - **images\_sum** (*array*) – Pilatus images integrated over the scan.
        - **integ\_qxy** (*array*) – Profile integrated along the horizontal axis.
        - **integ\_qz** (*array*) – Profile integrated along the vertical axis.
        - **qxy\_list** (*array*) – List of qxy in the profile.
        - **qz\_list** (*array*) – List of qz in the profile.

lib.backend.gixs.save\_gixs\_scan(*images*, *images\_sum*, *integ\_qxy*, *integ\_qz*, *qxy\_list*, *qz\_list*, *nxs\_name*, *stamps\_0d*, *data\_0d*, *fig*, *path\_to\_save\_dir*, *is\_print\_info*)¶
:   Save GIXS data.

    XXX\_pilatus\_sum.mat: the matrix corresponding to the image displayed, in ascii.
    XXX\_pilatus\_sum.tiff: the matrix corresponding to the image displayed, in tiff.
    XXX\_integrated\_qxy.dat: the horizontal integration of the whole detector as a function of qz (nm-1).
    XXX\_integrated\_qz.dat: the vertical integration of the whole detector as a function of qxy (nm-1).
    XXX\_images/XXX\_pliatus\_N.tiff : each image of the scan, in tiff.
    XXX.dat : the value of each sensor at each point of the scan.
    XXX.pdf : the figure in pdf.

    Parameters
    :   - **images** (*array*) – Array of individual Pilatus images.
        - **images\_sum** (*array*) – Pilatus images integrated over the scan.
        - **integ\_qxy** (*array*) – Profile integrated along the horizontal axis.
        - **integ\_qz** (*array*) – Profile integrated along the vertical axis.
        - **qxy\_list** (*array*) – List of qxy in the profile.
        - **qz\_list** (*array*) – List of qz in the profile.
        - **nxs\_name** (*str*) – Nexus name, e.g. SIRIUS\_2020\_03\_12\_0756.nxs.
        - **stamps\_0d** (*array*) – Aliases of each 0D sensor in the scan.
        - **data\_0d** (*array*) – Values of each 0D sensor in the scan.
        - **fig** (*None* *or* *matplotlib figure*) – The figure to be saved in pdf. Pass None if not wanted.
        - **path\_to\_save\_dir** (*str*) – Path to the directory where the treated files will be saved.
        - **is\_print\_info** (*bool*) – Verbose mode.

## lib.backend.isotherm module¶

Library for isotherms.

lib.backend.isotherm.extract\_isotherm\_scan(*nxs\_name*, *path\_to\_nxs\_dir*, *is\_print\_stamps*, *is\_print\_info*)¶
:   Extract the nexus file and return useful quantities.

    Parameters
    :   - **nxs\_name** (*str*) – Nexus name, e.g. SIRIUS\_2020\_03\_12\_0756.nxs.
        - **path\_to\_nxs\_dir** (*str*) – Path to the nexus files directory, e.g. user/.
        - **is\_print\_stamps** (*bool*) – Print the list of sensors contained in the nexus file.
        - **is\_print\_info** (*bool*) – Verbose mode.

    Returns
    :   - **area** (*array*) – List of area values.
        - **pressure** (*array*) – List of pressure values.
        - **time** (*array*) – List of time values.
        - **time\_str** (*str*) – Starting/ending dates of the scan.
        - **stamps\_0d** (*array*) – Aliases of each 0D sensor in the scan.
        - **data\_0d** (*array*) – Values of each 0D sensor in the scan.

    Raises
    :   - **FileNotFoundError** – If a file or a folder is missing.
        - **SystemExit****(****'Could not open Nexus file.'****)****)** – If the Nexus file cannot be accessed.
        - **SystemExit****(****'Required sensor not found.'****)** – If a required sensor is not found in the sensor list.

lib.backend.isotherm.plot\_isotherm\_scan(*area*, *pressure*, *time*, *first\_point\_plot*, *last\_point\_plot*, *nxs\_name*, *time\_str*)¶
:   Plot the isotherm.

    Parameters
    :   - **area** (*array*) – List of area values.
        - **pressure** (*array*) – List of pressure values.
        - **time** (*array*) – List of time values.
        - **first\_point\_plot** (*int*) – First point of the plots.
        - **last\_point\_plot** (*int*) – Last point of the plots.
        - **nxs\_name** (*str*) – Nexus name, e.g. SIRIUS\_2020\_03\_12\_0756.nxs.
        - **time\_str** (*str*) – Starting/ending dates of the scan.

    Returns
    :   **fig** – The figure to be saved in pdf.

    Return type
    :   matplotlib figure

lib.backend.isotherm.process\_isotherm\_scan(*nxs\_name*, *path\_to\_nxs\_dir*, *first\_point\_plot=0*, *last\_point\_plot=- 1*, *path\_to\_save\_dir=''*, *is\_print\_stamps=False*, *is\_plot=False*, *is\_save=False*, *is\_print\_info=False*)¶
:   Call functions for extracting, plotting, and saving an isotherm.

    Parameters
    :   - **nxs\_name** (*str*) – Nexus name, e.g. SIRIUS\_2020\_03\_12\_0756.nxs.
        - **path\_to\_nxs\_dir** (*str*) – Path to the nexus files directory, e.g. user/.
        - **first\_point\_plot** (*int**,* *optional*) – First point of the plots.
        - **last\_point\_plot** (*int**,* *optional*) – Last point of the plots.
        - **path\_to\_save\_dir** (*str**,* *optional*) – Path to the directory where the treated files will be saved.
        - **is\_print\_stamps** (*bool**,* *optional*) – Print the list of sensors contained in the nexus file.
        - **is\_plot** (*bool**,* *optional*) – Plot the 2D GIXD image and the integrated profiles.
        - **is\_save** (*bool**,* *optional*) – Save the results.
        - **is\_print\_info** (*bool**,* *optional*) – Verbose mode.

    Returns
    :   - **area** (*array*) – List of area values.
        - **pressure** (*array*) – List of pressure values.
        - **time** (*array*) – List of time values.

lib.backend.isotherm.save\_isotherm\_scan(*nxs\_name*, *stamps\_0d*, *data\_0d*, *fig*, *path\_to\_save\_dir*, *is\_print\_info*)¶
:   Save isotherm data.

    XXX.dat : the value of each sensor at each point of the scan.
    XXX.pdf : the figure in pdf.

    Parameters
    :   - **nxs\_name** (*str*) – Nexus name, e.g. SIRIUS\_2020\_03\_12\_0756.nxs.
        - **stamps\_0d** (*array*) – Aliases of each 0D sensor in the scan.
        - **data\_0d** (*array*) – Values of each 0D sensor in the scan.
        - **fig** (*None* *or* *matplotlib figure*) – The figure to be saved in pdf. Pass None if not wanted.
        - **path\_to\_save\_dir** (*str*) – Path to the directory where the treated files will be saved.
        - **is\_print\_info** (*bool*) – Verbose mode.

## lib.backend.xrf module¶

Library for XRF.

lib.backend.xrf.check\_sdd\_elems(*nxs\_name*, *path\_to\_nxs\_dir*, *list\_sdd\_elems*)¶
:   Check is all the SDD elems are in the Nexus file (ICR, OCR, spectrum).

    Parameters
    :   - **nxs\_name** (*str*) – Nexus name, e.g. SIRIUS\_2020\_03\_12\_0756.nxs.
        - **path\_to\_nxs\_dir** (*str*) – Path to the nexus files directory, e.g. user/.
        - **list\_sdd\_elems** (*list of int*) – List of SDD elements, e.g. [0, 1, 2].

    Returns
    :   - **is\_icr\_found** (*bool*) – True if all the required ICR are present in the Nexus file.
        - **is\_ocr\_found** (*bool*) – True if all the required OCR are present in the Nexus file.
        - **is\_spectrum\_found** (*bool*) – True if all the required spectrums are present in the Nexus file.

    Raises
    :   **SystemExit****(****'Could not open Nexus file.'****)****)** – If the Nexus file cannot be accessed.

lib.backend.xrf.extract\_xrf\_scan(*nxs\_name*, *path\_to\_nxs\_dir*, *list\_sdd\_elems*, *channel\_xrf\_first*, *channel\_xrf\_last*, *sdd\_gain*, *sdd\_ev0*, *is\_print\_stamps*, *is\_print\_info*)¶
:   Extract the nexus file and return useful quantities.

    Parameters
    :   - **nxs\_name** (*str*) – Nexus name, e.g. SIRIUS\_2020\_03\_12\_0756.nxs.
        - **path\_to\_nxs\_dir** (*str*) – Path to the nexus files directory, e.g. user/.
        - **list\_sdd\_elems** (*list of int*) – List of SDD elements, e.g. [0, 1, 2].
        - **channel\_xrf\_first** (*int*) – Spectrums are extracted between channel\_xrf\_first and channel\_xrf\_last.
        - **channel\_xrf\_last** (*int*) – Spectrums are extracted between channel\_xrf\_first and channel\_xrf\_last.
        - **sdd\_gain** (*float*) – Channels are converted to eVs following eVs = sdd\_gain\*channel+sdd\_ev0
        - **sdd\_ev0** (*float*) – Channels are converted to eVs following eVs = sdd\_gain\*channel+sdd\_ev0
        - **is\_print\_stamps** (*bool*) – Print the list of sensors contained in the nexus file.
        - **is\_print\_info** (*bool*) – Verbose mode.

    Returns
    :   - **channels** (*array*) – The extracted channels.
        - **energies** (*array*) – The extracted channels converted to eVs.
        - **spectrums** (*array*) – The extracted spectrums (between point 0 and last point with signal).
        - **first\_non\_zero\_spectrum** (*int*) – Argument of the first spectrum with signal.
        - **last\_non\_zero\_spectrum** (*int*) – Argument of the last spectrum with signal.
        - **time\_str** (*str*) – Starting/ending dates of the scan.
        - **stamps\_0d** (*array*) – Aliases of each 0D sensor in the scan.
        - **data\_0d** (*array*) – Values of each 0D sensor in the scan.

    Raises
    :   - **FileNotFoundError** – If a file or a folder is missing.
        - **SystemExit****(****'Could not open Nexus file.'****)****)** – If the Nexus file cannot be accessed.
        - **SystemExit****(****'Required sensor not found.'****)** – If a required sensor is not found in the sensor list.

lib.backend.xrf.plot\_xrf\_first\_last(*channels*, *energies*, *spectrums*, *arr\_peaks*, *first\_non\_zero\_spectrum*, *time\_str*, *is\_use\_ev*, *is\_first\_plot*, *is\_xrf\_log*, *absorbers*, *nxs\_name*)¶
:   Plot first and last spectrums of a XRF scan.

    Parameters
    :   - **channels** (*array*) – The extracted channels.
        - **energies** (*array*) – The extracted channels converted to eVs.
        - **spectrums** (*array*) – The extracted spectrums (between point 0 and last point with signal).
        - **arr\_peaks** (*array of tuples*) – Peaks to display, for ex. arr\_peaks = [(‘Elastic’, ‘12000.’), (‘Compton’, ‘11670.’)]
        - **first\_non\_zero\_spectrum** (*int*) – Argument of the first spectrum with signal.
        - **time\_str** (*str*) – Starting/ending dates of the scan.
        - **is\_use\_ev** (*bool*) – Convert the channels to eVs.
        - **is\_first\_plot** (*bool*) – True if this is the first plot of the series (to print title and text).
        - **is\_xrf\_log** (*bool*) – Log on the intensity in the plots.
        - **absorbers** (*str*) – Text to display which absorber was used.
        - **nxs\_name** (*str*) – Nexus name, e.g. SIRIUS\_2020\_03\_12\_0756.nxs.

    Returns
    :   **fig\_first\_last** – The figure of the first and last spectrums to be saved in pdf.

    Return type
    :   matplotlib figure

lib.backend.xrf.plot\_xrf\_spectrogram(*channels*, *energies*, *spectrums*, *last\_non\_zero\_spectrum*, *time\_str*, *is\_use\_ev*, *is\_first\_plot*, *is\_xrf\_log*, *absorbers*, *nxs\_name*)¶
:   Plot spectrogram of a XRF scan.

    Parameters
    :   - **channels** (*array*) – The extracted channels.
        - **energies** (*array*) – The extracted channels converted to eVs.
        - **spectrums** (*array*) – The extracted spectrums (between point 0 and last point with signal).
        - **last\_non\_zero\_spectrum** (*int*) – Argument of the last spectrum with signal.
        - **time\_str** (*str*) – Starting/ending dates of the scan.
        - **is\_use\_ev** (*bool*) – Convert the channels to eVs.
        - **is\_first\_plot** (*bool*) – True if this is the first plot of the series (to print title and text).
        - **is\_xrf\_log** (*bool*) – Log on the intensity in the plots.
        - **absorbers** (*str*) – Text to display which absorber was used.
        - **nxs\_name** (*str*) – Nexus name, e.g. SIRIUS\_2020\_03\_12\_0756.nxs.

    Returns
    :   **fig\_spectrogram** – The figure of the spectrogram to be saved in pdf.

    Return type
    :   matplotlib figure

lib.backend.xrf.plot\_xrf\_sum(*channels*, *energies*, *spectrums*, *arr\_peaks*, *time\_str*, *is\_use\_ev*, *is\_first\_plot*, *is\_xrf\_log*, *absorbers*, *nxs\_name*)¶
:   Plot sum over all the spectrums of a XRF scan.

    Parameters
    :   - **channels** (*array*) – The extracted channels.
        - **energies** (*array*) – The extracted channels converted to eVs.
        - **spectrums** (*array*) – The extracted spectrums (between point 0 and last point with signal).
        - **arr\_peaks** (*array of tuples*) – Peaks to display, for ex. arr\_peaks = [(‘Elastic’, ‘12000.’), (‘Compton’, ‘11670.’)]
        - **time\_str** (*str*) – Starting/ending dates of the scan.
        - **is\_use\_ev** (*bool*) – Convert the channels to eVs.
        - **is\_first\_plot** (*bool*) – True if this is the first plot of the series (to print title and text).
        - **is\_xrf\_log** (*bool*) – Log on the intensity in the plots.
        - **absorbers** (*str*) – Text to display which absorber was used.
        - **nxs\_name** (*str*) – Nexus name, e.g. SIRIUS\_2020\_03\_12\_0756.nxs.

    Returns
    :   **fig\_sum** – The figure of the sum to be saved in pdf.

    Return type
    :   matplotlib figure

lib.backend.xrf.process\_xrf\_scan(*nxs\_name*, *path\_to\_nxs\_dir*, *list\_sdd\_elems*, *channel\_xrf\_first=0*, *channel\_xrf\_last=2048*, *sdd\_gain=10.0*, *sdd\_ev0=0.0*, *arr\_peaks=[(None, None)]*, *is\_use\_ev=False*, *is\_xrf\_log=True*, *absorbers=''*, *path\_to\_save\_dir=''*, *is\_print\_stamps=False*, *is\_plot\_spectrogram=False*, *is\_plot\_sum=False*, *is\_plot\_first\_last=False*, *is\_save=False*, *is\_print\_info=False*)¶
:   Call functions for extracting, plotting, and saving a GIXD scan.

    Parameters
    :   - **nxs\_name** (*str*) – Nexus name, e.g. SIRIUS\_2020\_03\_12\_0756.nxs.
        - **path\_to\_nxs\_dir** (*str*) – Path to the nexus files directory, e.g. user/.
        - **list\_sdd\_elems** (*list of int*) – List of SDD elements, e.g. [0, 1, 2]
        - **channel\_xrf\_first** (*int**,* *optional*) – Spectrums are extracted between channel\_xrf\_first and channel\_xrf\_last.
        - **channel\_xrf\_last** (*int**,* *optional*) – Spectrums are extracted between channel\_xrf\_first and channel\_xrf\_last.
        - **sdd\_gain** (*float**,* *optional*) – Channels are converted to eVs following eVs = sdd\_gain\*channel+sdd\_ev0
        - **sdd\_ev0** (*float**,* *optional*) – Channels are converted to eVs following eVs = sdd\_gain\*channel+sdd\_ev0
        - **arr\_peaks** (*array of tuples**,* *optional*) – Peaks to display, for ex. arr\_peaks = [(‘Elastic’, ‘12000.’), (‘Compton’, ‘11670.’)]
        - **is\_use\_ev** (*bool**,* *optional*) – Convert the channels to eVs.
        - **is\_xrf\_log** (*bool**,* *optional*) – Log on the intensity in the plots.
        - **absorbers** (*str**,* *optional*) – Text to display which absorber was used.
        - **path\_to\_save\_dir** (*str**,* *optional*) – Path to the directory where the treated files will be saved.
        - **is\_print\_stamps** (*bool**,* *optional*) – Print the list of sensors contained in the nexus file.
        - **is\_plot\_spectrogram** (*bool**,* *optional*) – Plot the spectrogram.
        - **is\_plot\_sum** (*bool**,* *optional*) – Plot the sum of all the spectrums present in the scan.
        - **is\_plot\_first\_last** (*bool**,* *optional*) – Plot the first and last spectrums.
        - **is\_save** (*bool**,* *optional*) – Save the results.
        - **is\_print\_info** (*bool**,* *optional*) – Verbose mode.

    Returns
    :   - **channels** (*array*) – The extracted channels.
        - **energies** (*array*) – The extracted channels converted to eVs.
        - **spectrums** (*array*) – The extracted spectrums (between point 0 and last point with signal).

lib.backend.xrf.save\_xrf\_scan(*nxs\_name*, *path\_to\_nxs\_dir*, *stamps\_0d*, *data\_0d*, *fig\_spectrogram*, *fig\_sum*, *fig\_first\_last*, *path\_to\_save\_dir*, *is\_print\_info*)¶
:   Save XRF data.

    To avoid passing large variables such as each spectrum (not summed),
    we redo the extraction with PyNexus directly within this function.

    XXX\_fluospectrumNN.mat: the matrix corresponding to the spectrums of SDD elements
    NN, in ascii.
    XXX.dat : the value of each sensor at each point of the scan.
    XXX\_spectrogram.pdf : the figure of the spectrogram in pdf.
    XXX\_sum.pdf : the figure of the sum in pdf.
    XXX\_first\_last.pdf : the figure of the first and last spectrums in pdf.

    Parameters
    :   - **nxs\_name** (*str*) – Nexus name, e.g. SIRIUS\_2020\_03\_12\_0756.nxs.
        - **path\_to\_nxs\_dir** (*str*) – Path to the nexus files directory, e.g. user/.
        - **stamps\_0d** (*array*) – Aliases of each 0D sensor in the scan.
        - **data\_0d** (*array*) – Values of each 0D sensor in the scan.
        - **fig\_spectrogram** (*matplotlib None* *or* *matplotlib figure*) – The figure of the spectrogram to be saved in pdf.
        - **fig\_sum** (*matplotlib None* *or* *matplotlib figure*) – The figure of the sum to be saved in pdf.
        - **fig\_first\_last** (*matplotlib None* *or* *matplotlib figure*) – The figure of the first and last spectrums to be saved in pdf.
        - **path\_to\_save\_dir** (*str*) – Path to the directory where the treated files will be saved.
        - **is\_print\_info** (*bool*) – Verbose mode.

    Raises
    :   **SystemExit****(****'Could not open Nexus file.'****)****)** – If the Nexus file cannot be accessed.

## lib.backend.xrr module¶

Module for processing XRR (on solids and liquids).

lib.backend.xrr.extract\_direct\_xrr\_liquid(*direct\_scan\_name*, *roi\_x0*, *roi\_size\_x*, *roi\_size\_y*, *summation\_roi\_size\_y*, *is\_bckg\_up*, *is\_bckg\_down*, *is\_bckg\_left*, *is\_bckg\_right*, *is\_track\_beam*, *path\_to\_nxs\_dir*, *is\_print\_info*)¶
:   Extract the value of the direct beam.

    Parameters
    :   - **direct\_scan\_name** (*str*) – Nexus filename of the direct scan.
        - **roi\_x0** (*int*) – x0 of the full-scan ROI.
        - **roi\_size\_x** (*int*) – Size x of the full-scan ROI.
        - **roi\_size\_y** (*int*) – Size y of the full-scan ROI.
        - **summation\_roi\_size\_y** (*int*) – Size y of the summation ROI (centered on the beam).
          It has to be an odd number.
        - **is\_bckg\_up** (*bool*) – Take into account the upper ROI for background subtraction.
        - **is\_bckg\_down** (*bool*) – Take into account the lower ROI for background subtraction.
        - **is\_bckg\_left** (*bool*) – Take into account the left ROI for background subtraction.
        - **is\_bckg\_right** (*bool*) – Take into account the right ROI for background subtraction.
        - **is\_track\_beam** (*bool*) – Track the vertical position of the reflected beam.
        - **path\_to\_nxs\_dir** (*str*) – Path to the nexus files directory, e.g. user/.
        - **is\_print\_info** (*bool*) – Verbose mode.

    Returns
    :   **direct\_value** – Direct after background subtraction and normalization.

    Return type
    :   float

    Raises
    :   - **FileNotFoundError** – If a file or a folder is missing.
        - **SystemExit****(****'Could not open Nexus file.'****)****)** – If the Nexus file cannot be accessed.
        - **SystemExit****(****'Required sensor not found.'****)** – If a required sensor is not found in the sensor list.

lib.backend.xrr.extract\_direct\_xrr\_solid(*direct\_scan\_name*, *roi\_x0*, *roi\_size\_x*, *roi\_size\_y*, *is\_bckg\_up*, *is\_bckg\_down*, *is\_bckg\_left*, *is\_bckg\_right*, *path\_to\_nxs\_dir*, *is\_print\_info*)¶
:   Extract the value of the direct beam.

    Parameters
    :   - **direct\_scan\_name** (*str*) – Nexus filename of the direct scan.
        - **roi\_x0** (*int*) – x0 of the full-scan ROI.
        - **roi\_size\_x** (*int*) – Size x of the full-scan ROI.
        - **roi\_size\_y** (*int*) – Size y of the full-scan ROI.
          It has to be an odd number.
        - **is\_bckg\_up** (*bool*) – Take into account the upper ROI for background subtraction.
        - **is\_bckg\_down** (*bool*) – Take into account the lower ROI for background subtraction.
        - **is\_bckg\_left** (*bool*) – Take into account the left ROI for background subtraction.
        - **is\_bckg\_right** (*bool*) – Take into account the right ROI for background subtraction.
        - **path\_to\_nxs\_dir** (*str*) – Path to the nexus files directory, e.g. user/.
        - **is\_print\_info** (*bool*) – Verbose mode.

    Returns
    :   **direct\_value** – Direct after background subtraction and normalization.

    Return type
    :   float

    Raises
    :   - **FileNotFoundError** – If a file or a folder is missing.
        - **SystemExit****(****'Could not open Nexus file.'****)****)** – If the Nexus file cannot be accessed.
        - **SystemExit****(****'Required sensor not found.'****)** – If a required sensor is not found in the sensor list.

lib.backend.xrr.extract\_volt\_ion\_chamber\_liquid(*list\_xrr\_files*, *scan\_type*, *path\_to\_nxs\_dir*)¶
:   Extract the voltages of the ionization chamber in the case of an XRR on a liquid.

    Parameters
    :   - **list\_xrr\_files** (*list of str*) – List of XRR file names without the ‘.nxs’, e.g. [‘SIRIUS\_2021\_06\_18\_0568’].
        - **scan\_type** (*str*) – ‘refl’ or ‘direct’.
        - **path\_to\_nxs\_dir** (*str*) – directory where the nexus file is stored.

    Returns
    :   **voltages** – List of voltages of the ionization chamber (normalized by its gain).

    Return type
    :   array

lib.backend.xrr.extract\_xrr\_liquid\_scan(*list\_xrr\_files*, *path\_to\_nxs\_dir*, *roi\_x0*, *roi\_y0*, *roi\_size\_x*, *roi\_size\_y*, *summation\_roi\_size\_y*, *m4pitch0*, *wavelength*, *direct\_value*, *is\_bckg\_up*, *is\_bckg\_down*, *is\_bckg\_left*, *is\_bckg\_right*, *is\_track\_beam*, *is\_print\_info*)¶
:   Extract the XRR from a series of scans.

    Parameters
    :   - **list\_xrr\_files** (*list of str*) – List of XRR file names without the ‘.nxs’, e.g. [‘SIRIUS\_2021\_06\_18\_0568’].
        - **path\_to\_nxs\_dir** (*str*) – Path to the nexus files directory, e.g. user/.
        - **roi\_x0** (*int*) – x0 of the full-scan ROI.
        - **roi\_y0** (*int*) – y0 of the full-scan ROI.
        - **roi\_size\_x** (*int*) – Size x of the full-scan ROI.
        - **roi\_size\_y** (*int*) – Size y of the full-scan ROI.
        - **summation\_roi\_size\_y** (*int*) – Size y of the summation ROI (centered on the beam).
          It has to be an odd number.
        - **m4pitch0** (*float*) – Value of m4pitch0 (m4pitch aligned with the beam) in deg.
        - **wavelength** (*float*) – Wavelength in nm.
        - **direct\_value** (*float*) – Direct after background subtraction and normalization.
        - **is\_bckg\_up** (*bool*) – Take into account the upper ROI for background subtraction.
        - **is\_bckg\_down** (*bool*) – Take into account the lower ROI for background subtraction.
        - **is\_bckg\_left** (*bool*) – Take into account the left ROI for background subtraction.
        - **is\_bckg\_right** (*bool*) – Take into account the right ROI for background subtraction.
        - **is\_track\_beam** (*bool*) – Track the vertical position of the reflected beam.
        - **is\_print\_info** (*bool*) – Verbose mode.

    Returns
    :   - **m4pitch** (*array*) – List of m4pitch (deg).
        - **theta** (*array*) – List of theta (rad).
        - **qz** (*array*) – List of qz (nm-1).
        - **pos\_y\_beam** (*array*) – List of vertical positions of the reflected beam (pix).
        - **bckg\_refl\_up** (*array*) – List of integrated intensities of the bckg up.
        - **bckg\_refl\_down** (*array*) – List of integrated intensities of the bckg down.
        - **bckg\_refl\_left** (*array*) – List of integrated intensities of the bckg left.
        - **bckg\_refl\_right** (*array*) – List of integrated intensities of the bckg right.
        - **bckg\_refl** (*array*) – List of averaged chosen backgrounds.
        - **err\_refl** (*array*) – List of error bars of the XRR.
        - **refl** (*array*) – List of values of the normalized XRR.
        - **time\_str** (*str*) – Starting/ending dates of the whole XRR scan.

    Raises
    :   - **FileNotFoundError** – If a file or a folder is missing.
        - **SystemExit****(****'Could not open Nexus file.'****)****)** – If the Nexus file cannot be accessed.
        - **SystemExit****(****'Required sensor not found.'****)** – If a required sensor is not found in the sensor list.

lib.backend.xrr.extract\_xrr\_solid\_scan(*list\_xrr\_files*, *path\_to\_nxs\_dir*, *roi\_x0*, *roi\_y0*, *roi\_size\_x*, *roi\_size\_y*, *wavelength*, *direct\_value*, *is\_bckg\_up*, *is\_bckg\_down*, *is\_bckg\_left*, *is\_bckg\_right*, *is\_print\_info*)¶
:   Extract the XRR from a series of scans.

    Parameters
    :   - **list\_xrr\_files** (*list of str*) – List of XRR file names without the ‘.nxs’, e.g. [‘SIRIUS\_2021\_06\_18\_0568’].
        - **path\_to\_nxs\_dir** (*str*) – Path to the nexus files directory, e.g. user/.
        - **roi\_x0** (*int*) – x0 of the full-scan ROI.
        - **roi\_y0** (*int*) – y0 of the full-scan ROI.
        - **roi\_size\_x** (*int*) – Size x of the full-scan ROI.
        - **roi\_size\_y** (*int*) – Size y of the full-scan ROI.
          It has to be an odd number.
        - **wavelength** (*float*) – Wavelength in nm.
        - **direct\_value** (*float*) – Direct after background subtraction and normalization.
        - **is\_bckg\_up** (*bool*) – Take into account the upper ROI for background subtraction.
        - **is\_bckg\_down** (*bool*) – Take into account the lower ROI for background subtraction.
        - **is\_bckg\_left** (*bool*) – Take into account the left ROI for background subtraction.
        - **is\_bckg\_right** (*bool*) – Take into account the right ROI for background subtraction.
        - **is\_print\_info** (*bool*) – Verbose mode.

    Returns
    :   - **theta** (*array*) – List of theta (rad).
        - **qz** (*array*) – List of qz (nm-1).
        - **bckg\_refl\_up** (*array*) – List of integrated intensities of the bckg up.
        - **bckg\_refl\_down** (*array*) – List of integrated intensities of the bckg down.
        - **bckg\_refl\_left** (*array*) – List of integrated intensities of the bckg left.
        - **bckg\_refl\_right** (*array*) – List of integrated intensities of the bckg right.
        - **bckg\_refl** (*array*) – List of averaged chosen backgrounds.
        - **err\_refl** (*array*) – List of error bars of the XRR.
        - **refl** (*array*) – List of values of the normalized XRR.
        - **time\_str** (*str*) – Starting/ending dates of the whole XRR scan.

    Raises
    :   - **FileNotFoundError** – If a file or a folder is missing.
        - **SystemExit****(****'Could not open Nexus file.'****)****)** – If the Nexus file cannot be accessed.
        - **SystemExit****(****'Required sensor not found.'****)** – If a required sensor is not found in the sensor list.

lib.backend.xrr.plot\_calib\_xrr\_liquid(*calib\_xrr\_data*, *distance\_detec*)¶
:   Fit and plot the values from the calibration,
    to give the user the coefficient to be used in the XRR scripts (on liquids).

    Parameters
    :   - **calib\_xrr\_data** (*array*) – Numpy array containing the values of m4pitch, c10tablepitch, gamma and zs for the calibration.
        - **distance\_detec** (*float*) – Distance between the detector and the center of the sample in mm.

lib.backend.xrr.plot\_xrr\_m4pitch(*m4pitch*, *bckg\_refl*, *err\_refl*, *refl*, *list\_xrr\_files*, *is\_first\_plot*, *time\_str*)¶
:   Plot XRR as a function of m4pitch.

    Parameters
    :   - **m4pitch** (*array*) – List of m4pitch (deg).
        - **bckg\_refl** (*array*) – List of averaged chosen backgrounds.
        - **err\_refl** (*array*) – List of error bars of the XRR.
        - **refl** (*array*) – List of values of the normalized XRR.
        - **list\_xrr\_files** (*list of str*) – List of XRR file names without the ‘.nxs’, e.g. [‘SIRIUS\_2021\_06\_18\_0568’].
        - **is\_first\_plot** (*bool*) – True if this is the first plot of the series (to print title and text).
        - **time\_str** (*str*) – Starting/ending dates of the whole XRR scan.

    Returns
    :   **fig\_m4pitch** – The figure to be saved in pdf.

    Return type
    :   matplotlib figure

lib.backend.xrr.plot\_xrr\_pos\_y\_beam(*m4pitch*, *pos\_y\_beam*, *list\_xrr\_files*, *is\_first\_plot*, *time\_str*)¶
:   Plot the vertical position of the reflected beam
    as a function of m4pitch.

    Parameters
    :   - **m4pitch** (*array*) – List of m4pitch (deg).
        - **pos\_y\_beam** (*array*) – List of vertical positions of the reflected beam (pix).
        - **list\_xrr\_files** (*list of str*) – List of XRR file names without the ‘.nxs’, e.g. [‘SIRIUS\_2021\_06\_18\_0568’].
        - **is\_first\_plot** (*bool*) – True if this is the first plot of the series (to print title and text).
        - **time\_str** (*str*) – Starting/ending dates of the whole XRR scan.

    Returns
    :   **fig\_pos\_y\_beam** – The figure to be saved in pdf.

    Return type
    :   matplotlib figure

lib.backend.xrr.plot\_xrr\_qz(*qz*, *bckg\_refl*, *err\_refl*, *refl*, *list\_xrr\_files*, *is\_first\_plot*, *time\_str*)¶
:   Plot XRR as a function of qz.

    Parameters
    :   - **qz** (*array*) – List of qz (nm-1).
        - **bckg\_refl** (*array*) – List of averaged chosen backgrounds.
        - **err\_refl** (*array*) – List of error bars of the XRR.
        - **refl** (*array*) – List of values of the normalized XRR.
        - **list\_xrr\_files** (*list of str*) – List of XRR file names without the ‘.nxs’, e.g. [‘SIRIUS\_2021\_06\_18\_0568’].
        - **is\_first\_plot** (*bool*) – True if this is the first plot of the series (to print title and text).
        - **time\_str** (*str*) – Starting/ending dates of the whole XRR scan.

    Returns
    :   **fig\_qz** – The figure to be saved in pdf.

    Return type
    :   matplotlib figure

lib.backend.xrr.plot\_xrr\_twotheta(*theta*, *bckg\_refl*, *err\_refl*, *refl*, *list\_xrr\_files*, *is\_first\_plot*, *time\_str*)¶
:   Plot XRR as a function of 2\*theta.

    Parameters
    :   - **theta** (*array*) – List of theta (rad).
        - **bckg\_refl** (*array*) – List of averaged chosen backgrounds.
        - **err\_refl** (*array*) – List of error bars of the XRR.
        - **refl** (*array*) – List of values of the normalized XRR.
        - **list\_xrr\_files** (*list of str*) – List of XRR file names without the ‘.nxs’, e.g. [‘SIRIUS\_2021\_06\_18\_0568’].
        - **is\_first\_plot** (*bool*) – True if this is the first plot of the series (to print title and text).
        - **time\_str** (*str*) – Starting/ending dates of the whole XRR scan.

    Returns
    :   **fig\_twotheta** – The figure to be saved in pdf.

    Return type
    :   matplotlib figure

lib.backend.xrr.process\_xrr\_liquid\_scan(*list\_xrr\_files*, *path\_to\_nxs\_dir*, *direct\_scan\_name*, *roi\_x0*, *roi\_y0*, *roi\_size\_x*, *roi\_size\_y*, *summation\_roi\_size\_y*, *m4pitch0*, *wavelength*, *fdirect=1.0*, *is\_bckg\_up=True*, *is\_bckg\_down=True*, *is\_bckg\_left=False*, *is\_bckg\_right=False*, *is\_force\_direct=True*, *is\_track\_beam=True*, *path\_to\_save\_dir=''*, *is\_plot\_m4pitch=False*, *is\_plot\_twotheta=False*, *is\_plot\_qz=False*, *is\_plot\_pos\_y\_beam=False*, *is\_save=False*, *is\_print\_info=False*)¶
:   Call functions for extracting, plotting, and saving a XRR scan on a liquid.

    Parameters
    :   - **list\_xrr\_files** (*list of str*) – List of XRR file names without the ‘.nxs’, e.g. [‘SIRIUS\_2021\_06\_18\_0568’].
        - **path\_to\_nxs\_dir** (*str*) – Path to the nexus files directory, e.g. user/.
        - **direct\_scan\_name** (*str*) – Nexus filename of the direct scan.
        - **roi\_x0** (*int*) – x0 of the full-scan ROI.
        - **roi\_y0** (*int*) – y0 of the full-scan ROI.
        - **roi\_size\_x** (*int*) – Size x of the full-scan ROI.
        - **roi\_size\_y** (*int*) – Size y of the full-scan ROI.
        - **summation\_roi\_size\_y** (*int*) – Size y of the summation ROI (centered on the beam).
          It has to be an odd number.
        - **m4pitch0** (*float*) – Value of m4pitch0 (m4pitch aligned with the beam) in deg.
        - **wavelength** (*float*) – Wavelength in nm.
        - **fdirect** (*float**,* *optional*) – Value of the normalisation to be used if is\_force\_direct is True
        - **is\_bckg\_up** (*bool**,* *optional*) – Take into account the upper ROI for background subtraction.
        - **is\_bckg\_down** (*bool**,* *optional*) – Take into account the lower ROI for background subtraction.
        - **is\_bckg\_left** (*bool**,* *optional*) – Take into account the left ROI for background subtraction.
        - **is\_bckg\_right** (*bool**,* *optional*) – Take into account the right ROI for background subtraction.
        - **is\_force\_direct** (*bool**,* *optional*) – Force the normalization to be equal to the value of fdirect.
        - **is\_track\_beam** (*bool**,* *optional*) – Track the vertical position of the reflected beam.
        - **path\_to\_save\_dir** (*str**,* *optional*) – Path to the directory where the treated files will be saved.
        - **is\_plot\_m4pitch** (*bool**,* *optional*) – Plot the XRR as a function of m4pitch.
        - **is\_plot\_twotheta** (*bool**,* *optional*) – Plot the XRR as a function of 2\*theta.
        - **is\_plot\_qz** (*bool**,* *optional*) – Plot the XRR as a function of qz.
        - **is\_plot\_pos\_y\_beam** (*bool**,* *optional*) – Plot the vertical position of the reflected beam as a function of m4pitch.
        - **is\_save** (*bool**,* *optional*) – Save the results.
        - **is\_print\_info** (*bool**,* *optional*) – Verbose mode.

    Returns
    :   - **m4pitch** (*array*) – List of m4pitch (deg).
        - **theta** (*array*) – List of theta (rad).
        - **qz** (*array*) – List of qz (nm-1).
        - **bckg\_refl\_up** (*array*) – List of integrated intensities of the bckg up.
        - **bckg\_refl\_down** (*array*) – List of integrated intensities of the bckg down.
        - **bckg\_refl\_left** (*array*) – List of integrated intensities of the bckg left.
        - **bckg\_refl\_right** (*array*) – List of integrated intensities of the bckg right.
        - **bckg\_refl** (*array*) – List of averaged chosen backgrounds.
        - **err\_refl** (*array*) – List of error bars of the XRR.
        - **refl** (*array*) – List of values of the normalized XRR.

    Raises
    :   - **FileNotFoundError** – If a file or a folder is missing.
        - **SystemExit****(****'Could not open Nexus file.'****)****)** – If the Nexus file cannot be accessed.
        - **SystemExit****(****'Required sensor not found.'****)** – If a required sensor is not found in the sensor list.

lib.backend.xrr.process\_xrr\_solid\_scan(*list\_xrr\_files*, *path\_to\_nxs\_dir*, *direct\_scan\_name*, *roi\_x0*, *roi\_y0*, *roi\_size\_x*, *roi\_size\_y*, *wavelength*, *fdirect=1.0*, *is\_bckg\_up=True*, *is\_bckg\_down=True*, *is\_bckg\_left=False*, *is\_bckg\_right=False*, *is\_force\_direct=True*, *path\_to\_save\_dir=''*, *is\_plot\_twotheta=False*, *is\_plot\_qz=False*, *is\_save=False*, *is\_print\_info=False*)¶
:   Call functions for extracting, plotting, and saving a XRR scan on a solid.

    Parameters
    :   - **list\_xrr\_files** (*list of str*) – List of XRR file names without the ‘.nxs’, e.g. [‘SIRIUS\_2021\_06\_18\_0568’].
        - **path\_to\_nxs\_dir** (*str*) – Path to the nexus files directory, e.g. user/.
        - **direct\_scan\_name** (*str*) – Nexus filename of the direct scan.
        - **roi\_x0** (*int*) – x0 of the full-scan ROI.
        - **roi\_y0** (*int*) – y0 of the full-scan ROI.
        - **roi\_size\_x** (*int*) – Size x of the full-scan ROI.
        - **roi\_size\_y** (*int*) – Size y of the full-scan ROI.
          It has to be an odd number.
        - **wavelength** (*float*) – Wavelength in nm.
        - **fdirect** (*float**,* *optional*) – Value of the normalisation to be used if is\_force\_direct is True
        - **is\_bckg\_up** (*bool**,* *optional*) – Take into account the upper ROI for background subtraction.
        - **is\_bckg\_down** (*bool**,* *optional*) – Take into account the lower ROI for background subtraction.
        - **is\_bckg\_left** (*bool**,* *optional*) – Take into account the left ROI for background subtraction.
        - **is\_bckg\_right** (*bool**,* *optional*) – Take into account the right ROI for background subtraction.
        - **is\_force\_direct** (*bool**,* *optional*) – Force the normalization to be equal to the value of fdirect.
        - **path\_to\_save\_dir** (*str**,* *optional*) – Path to the directory where the treated files will be saved.
        - **is\_plot\_twotheta** (*bool**,* *optional*) – Plot the XRR as a function of 2\*theta.
        - **is\_plot\_qz** (*bool**,* *optional*) – Plot the XRR as a function of qz.
        - **is\_save** (*bool**,* *optional*) – Save the results.
        - **is\_print\_info** (*bool**,* *optional*) – Verbose mode.

    Returns
    :   - **theta** (*array*) – List of theta (rad).
        - **qz** (*array*) – List of qz (nm-1).
        - **bckg\_refl\_up** (*array*) – List of integrated intensities of the bckg up.
        - **bckg\_refl\_down** (*array*) – List of integrated intensities of the bckg down.
        - **bckg\_refl\_left** (*array*) – List of integrated intensities of the bckg left.
        - **bckg\_refl\_right** (*array*) – List of integrated intensities of the bckg right.
        - **bckg\_refl** (*array*) – List of averaged chosen backgrounds.
        - **err\_refl** (*array*) – List of error bars of the XRR.
        - **refl** (*array*) – List of values of the normalized XRR.

    Raises
    :   - **FileNotFoundError** – If a file or a folder is missing.
        - **SystemExit****(****'Could not open Nexus file.'****)****)** – If the Nexus file cannot be accessed.
        - **SystemExit****(****'Required sensor not found.'****)** – If a required sensor is not found in the sensor list.

lib.backend.xrr.save\_xrr\_liquid\_scan(*m4pitch*, *theta*, *qz*, *pos\_y\_beam*, *bckg\_refl\_up*, *bckg\_refl\_down*, *bckg\_refl\_left*, *bckg\_refl\_right*, *bckg\_refl*, *err\_refl*, *refl*, *list\_xrr\_files*, *fig\_m4pitch*, *fig\_twotheta*, *fig\_qz*, *fig\_pos\_y\_beam*, *path\_to\_nxs\_dir*, *path\_to\_save\_dir*, *is\_print\_info*)¶
:   Save data of a XRR scan on a liquid.

    To save the .dat of each individual file,
    we redo the extraction with PyNexus directly within this function.

    XXX\_XRR.dat: different parameters relevant for XRR for each point of m4pitch taken.
    XXX.dat: the value of each sensor at each point of each scan in the list of XRR files.
    XXX\_XRR\_m4pitch.pdf, XXX\_XRR\_qz.pdf,
    XXX\_XRR\_twotheta.pdf, XXX\_XRR\_pos\_y\_beam.pdf: the figures in pdf.

    Parameters
    :   - **m4pitch** (*array*) – List of m4pitch (deg).
        - **theta** (*array*) – List of theta (rad).
        - **qz** (*array*) – List of qz (nm-1).
        - **pos\_y\_beam** (*array*) – List of vertical positions of the reflected beam (pix).
        - **bckg\_refl\_up** (*array*) – List of integrated intensities of the bckg up.
        - **bckg\_refl\_down** (*array*) – List of integrated intensities of the bckg down.
        - **bckg\_refl\_left** (*array*) – List of integrated intensities of the bckg left.
        - **bckg\_refl\_right** (*array*) – List of integrated intensities of the bckg right.
        - **bckg\_refl** (*array*) – List of averaged chosen backgrounds.
        - **err\_refl** (*array*) – List of error bars of the XRR.
        - **refl** (*array*) – List of values of the normalized XRR.
        - **list\_xrr\_files** (*list of str*) – List of XRR file names without the ‘.nxs’, e.g. [‘SIRIUS\_2021\_06\_18\_0568’].
        - **fig\_m4pitch** (*matplotlib figure*) – The figure to be saved in pdf.
        - **fig\_twotheta** (*matplotlib figure*) – The figure to be saved in pdf.
        - **fig\_qz** (*matplotlib figure*) – The figure to be saved in pdf.
        - **fig\_pos\_y\_beam** (*matplotlib figure*) – The figure to be saved in pdf.
        - **path\_to\_nxs\_dir** (*str*) – Path to the nexus files directory, e.g. user/.
        - **path\_to\_save\_dir** (*str*) – Path to the directory where the treated files will be saved.
        - **is\_print\_info** (*bool*) – Verbose mode.

    Raises
    :   **SystemExit****(****'Could not open Nexus file.'****)****)** – If the Nexus file cannot be accessed.

lib.backend.xrr.save\_xrr\_solid\_scan(*theta*, *qz*, *bckg\_refl\_up*, *bckg\_refl\_down*, *bckg\_refl\_left*, *bckg\_refl\_right*, *bckg\_refl*, *err\_refl*, *refl*, *list\_xrr\_files*, *fig\_twotheta*, *fig\_qz*, *path\_to\_nxs\_dir*, *path\_to\_save\_dir*, *is\_print\_info*)¶
:   Save data of a XRR scan on a solid.

    To save the .dat of each individual file,
    we redo the extraction with PyNexus directly within this function.

    XXX\_XRR.dat: different parameters relevant for XRR for each point of theta taken.
    XXX.dat: the value of each sensor at each point of each scan in the list of XRR files.
    XXX\_XRR\_qz.pdf, XXX\_XRR\_twotheta.pdf: the figures in pdf.

    Parameters
    :   - **theta** (*array*) – List of theta (rad).
        - **qz** (*array*) – List of qz (nm-1).
        - **bckg\_refl\_up** (*array*) – List of integrated intensities of the bckg up.
        - **bckg\_refl\_down** (*array*) – List of integrated intensities of the bckg down.
        - **bckg\_refl\_left** (*array*) – List of integrated intensities of the bckg left.
        - **bckg\_refl\_right** (*array*) – List of integrated intensities of the bckg right.
        - **bckg\_refl** (*array*) – List of averaged chosen backgrounds.
        - **err\_refl** (*array*) – List of error bars of the XRR.
        - **refl** (*array*) – List of values of the normalized XRR.
        - **list\_xrr\_files** (*list of str*) – List of XRR file names without the ‘.nxs’, e.g. [‘SIRIUS\_2021\_06\_18\_0568’].
        - **fig\_twotheta** (*matplotlib figure*) – The figure to be saved in pdf.
        - **fig\_qz** (*matplotlib figure*) – The figure to be saved in pdf.
        - **path\_to\_nxs\_dir** (*str*) – Path to the nexus files directory, e.g. user/.
        - **path\_to\_save\_dir** (*str*) – Path to the directory where the treated files will be saved.
        - **is\_print\_info** (*bool*) – Verbose mode.

    Raises
    :   **SystemExit****(****'Could not open Nexus file.'****)****)** – If the Nexus file cannot be accessed.

## Module contents¶

### Table of Contents

- lib.backend package
  - Submodules
  - lib.backend.PyNexus module
  - lib.backend.area\_detector module
  - lib.backend.data\_1d module
  - lib.backend.gixd module
  - lib.backend.gixs module
  - lib.backend.isotherm module
  - lib.backend.xrf module
  - lib.backend.xrr module
  - Module contents

### This Page

- Show Source

### Quick search

### Navigation

- index
- modules |
- JupyLabBook v3.0 documentation »
- lib.backend package

© Copyright 2022, Hemmerle Arnaud.
Created using Sphinx 5.0.2.
