# Supplementary material for: Opportunities and new developments for the study of surfaces and interfaces in soft condensed matter at the SIRIUS beamline of Synchrotron SOLEIL
Source: J Synchrotron Radiat. 2024 Jan 1;31(Pt 1):162–76. doi: 10.1107/S1600577523008810 (PMC10833424; doi:10.1107/S1600577523008810)
Supplement: Supplementary file 1 [file s-31-00162-sup1.zip › JupyLabBook-v3.0.2/docs/sphinx/build/html/lib.frontend.html]

lib.frontend package — JupyLabBook v3.0 documentation

### Navigation

- index
- modules |
- JupyLabBook v3.0 documentation »
- lib.frontend package

# lib.frontend package¶

## Subpackages¶

- lib.frontend.process\_widgets package
  - Submodules
  - lib.frontend.process\_widgets.warea\_detector module
  - lib.frontend.process\_widgets.wdata\_1d module
  - lib.frontend.process\_widgets.wgixd module
  - lib.frontend.process\_widgets.wgixs module
  - lib.frontend.process\_widgets.wisotherm module
  - lib.frontend.process\_widgets.wxrf module
  - lib.frontend.process\_widgets.wxrr module
  - Module contents

## Submodules¶

## lib.frontend.action module¶

Module for action widgets.

lib.frontend.action.display\_widgets\_action(*expt*)¶
:   Set and display the widgets for choosing an action.

    Parameters
    :   **expt** (*object*) – Object of the class Experiment.

## lib.frontend.experiment module¶

Module defining the class Experiment.

*class* lib.frontend.experiment.Experiment¶
:   Bases: `object`

    Class defining an Experiment.

    paths¶
    :   Dictionary of paths to files and folders.

        Type
        :   dict

    params¶
    :   Dictionary of parameters.

        Type
        :   dict

    default¶
    :   Dictionary of default values.

        Type
        :   dict

    is\_paths\_ok¶
    :   True if all the paths are correct.

        Type
        :   bool

    list\_nxs¶
    :   List of nexus filenames in the nxs folder.

        Type
        :   list of str

    list\_logs¶
    :   List of log filenames in the logs folder.

        Type
        :   list of str

    list\_scripts¶
    :   List of script filenames in the scripts folder.

        Type
        :   list of str

    list\_scans¶
    :   List of scans selected in the interactive widget.

        Type
        :   list of objects Scan

    list\_params\_files¶
    :   List of json files in the params folder.

        Type
        :   list of str

    default\_log¶
    :   List of commands from a log file.

        Type
        :   str

    check\_and\_set\_paths(*paths*)¶
    :   Check if the paths exist and set the attribute paths.

        Parameters
        :   **paths** (*dict*) – Dictionary of paths to files and folders.

    get\_default\_param\_value(*key*, *alt*)¶
    :   Return the value associated to the key if it already exists,
        else return the alternative value.

        Parameters
        :   - **key** (*str*) – Key in the dictionnary params.
            - **alt** – Alternative value to return if the key is not present in params.

        Returns
        :   Value associated to the key or alternative value if not present in params.

        Return type
        :   value

    load\_params\_from\_json(*path\_to\_params*)¶
    :   Import a json file of params and set the attribute params.

        Parameters
        :   **path\_to\_params** (*str*) – Path to the json file.

    save\_params\_in\_json(*path\_to\_params*)¶
    :   Save the attribute params in a json file.

        Parameters
        :   **path\_to\_params** (*str*) – Path to the json file.

    set\_list\_images()¶
    :   Refresh the list of files in the images folder and set the attribute list\_images.

    set\_list\_logs()¶
    :   Refresh the list of log files and set the attribute list\_logs.

    set\_list\_nxs()¶
    :   Refresh the list of nxs files and set the attribute list\_nxs.

    set\_list\_params\_files()¶
    :   Refresh the list of params files and set the attribute list\_params\_files.

    set\_list\_scripts()¶
    :   Refresh the list of script files and set the attribute list\_scripts.

    set\_scans(*list\_nxs*)¶
    :   Create a list of object Scans.

        Parameters
        :   **list\_nxs** (*list of str*) – List of nexus filenames in the nxs folder.

    update\_param\_from\_widget(*dict\_widgets*, *list\_keys*)¶
    :   Update the default value of the keys in params with the value of the
        corresponding widget.

        Parameters
        :   - **dict\_widgets** (*dict*) – Dictionnary with the widgets.
            - **list\_keys** (*list of str*) – List of keys in params to update.

## lib.frontend.form module¶

Module for inserting a form (description of the experiment) in the notebook.

lib.frontend.form.display\_widgets\_form()¶
:   Set and display the widgets for filling and inserting a form in
    the notebook.

## lib.frontend.jlb\_io module¶

Module dealing with the various imports and exports (pdf, logs, state …)
and insertions in the notebook.

lib.frontend.jlb\_io.display\_widgets\_insert\_from\_log(*expt*)¶
:   Set and display the widgets for inserting commands or positions
    from a log file.

    Parameters
    :   **expt** (*object*) – Object of the class Experiment.

lib.frontend.jlb\_io.display\_widgets\_insert\_image(*path\_to\_images\_dir*, *list\_images*)¶
:   Set and display the widgets for inserting an image.

    Parameters
    :   - **path\_to\_images\_dir** (*str*) – Path to the directory with the images.
        - **list\_images** (*list of str*) – List of images filenames in the images folder.

lib.frontend.jlb\_io.display\_widgets\_insert\_script(*path\_to\_scripts\_dir*, *list\_scripts*)¶
:   Set and display the widgets for inserting a script.

    Parameters
    :   - **path\_to\_scripts\_dir** (*str*) – Path to the directory with the scripts.
        - **list\_scripts** (*list of str*) – List of script filenames in the scripts folder.

lib.frontend.jlb\_io.display\_widgets\_insert\_text()¶
:   Set and display the widgets for inserting text.

lib.frontend.jlb\_io.display\_widgets\_load\_params\_from\_json(*expt*)¶
:   Set and display the widgets for loading params from
    a json file.

    Parameters
    :   **expt** (*object*) – Object of the class Experiment.

lib.frontend.jlb\_io.display\_widgets\_save\_params\_in\_json(*expt*)¶
:   Set and display the widgets for saving params in
    a json file.

    Parameters
    :   **expt** (*object*) – Object of the class Experiment.

lib.frontend.jlb\_io.export\_logs\_to\_rlogs(*path\_to\_logs\_dir*, *list\_logs*, *verbose=True*, *output\_dir='readable\_logs/'*)¶
:   Export all the logs to a human-readable version with date and commands only.

    Parameters
    :   - **path\_to\_logs\_dir** (*str*) – Path to the logs files directory, e.g. user/logs/.
        - **list\_logs** (*list of str*) – List of log filenames in the logs folder.
        - **verbose** (*bool**,* *optional*) – Verbose mode.
        - **output\_dir** (*str**,* *optional*) – Output directory.

lib.frontend.jlb\_io.export\_nb\_to\_pdf(*path\_to\_nb*, *timeout=100.0*, *verbose=True*, *output\_dir='.'*)¶
:   Export the notebook to pdf.

    Parameters
    :   - **path\_to\_nb** (*str*) – Path to the notebook. PDF is saved in the same folder.
        - **timeout** (*float**,* *optional*) – Time in s before timeout.
        - **verbose** (*bool**,* *optional*) – Verbose mode.
        - **output\_dir** (*str**,* *optional*) – Output directory.

lib.frontend.jlb\_io.extract\_absorbers\_from\_log(*path\_to\_logs\_dir*, *list\_logs*, *scan\_name*)¶
:   Extract from a log file the absorbers used for a specific scan.

    Parameters
    :   - **path\_to\_logs\_dir** (*str*) – Path to the logs files directory, e.g. user/logs/.
        - **list\_logs** (*list of str*) – List of log filenames in the logs folder.
        - **scan\_name** (*str*) – Name of the scan, e.g. ‘SIRIUS\_2020\_03\_12\_0756’.

    Returns
    :   **absorbers** – Absorbers used for the scan.

    Return type
    :   str

lib.frontend.jlb\_io.extract\_commands\_from\_log(*path\_to\_log\_file*)¶
:   Extract all commands from a log file.

    Parameters
    :   **path\_to\_log\_file** (*str*) – Path to the log file.

    Returns
    :   **arr\_all\_commands** – Extracted commands.

    Return type
    :   array of str

lib.frontend.jlb\_io.extract\_dead\_pixels(*file\_dead\_pixels*)¶
:   Extract the dead pixels file.

    Parameters
    :   **file\_dead\_pixels** (*str*) – Path to the dead pixels file, e.g. ‘lib/params/dead\_pixels/dead\_pixels\_pilatus.dat’

    Returns
    :   **arr\_dead\_pixels** – (y,x) positions of the dead pixels.

    Return type
    :   array

lib.frontend.jlb\_io.extract\_positions\_from\_log(*path\_to\_log\_file*)¶
:   Extract all positions from a log file.

    Parameters
    :   **path\_to\_log\_file** (*str*) – Path to the log file.

    Returns
    :   **list\_positions** – Extracted positions.

    Return type
    :   array of str

## lib.frontend.notebook module¶

Module dealing with actions on the notebook itself (create cell, save nb, …).
The code requires use of Javascript to modify the structure of the notebook
and is therefore not usable in Jupyter Lab, which forbids Javascript.

lib.frontend.notebook.create\_cell(*code*, *position='below'*, *celltype='markdown'*, *is\_print=True*, *is\_execute=True*)¶
:   Create a cell in the Jupyter Notebook.

    Parameters
    :   - **code** (*str*) – Code to display in the cell
        - **position** (*str**,* *optional*) – Where to put the cell: ‘below’ or ‘at\_bottom’
        - **celltype** (*str**,* *optional*) – Type of cell: ‘code’ or ‘markdown’
        - **is\_print** (*bool**,* *optional*) – Print the cell in the pdf report
        - **is\_execute** (*bool**,* *optional*) – Execute the cell after its creation

lib.frontend.notebook.create\_cell\_action\_widgets(*is\_delete\_current\_cell*)¶
:   Delete the current cell if asked, and create a cell to call the action widgets.

    Parameters
    :   **is\_delete\_current\_cell** (*bool*) – Delete the current cell if True.

lib.frontend.notebook.delete\_cell()¶
:   Delete the cell in which this function was called in the Jupyter Notebook.

lib.frontend.notebook.refresh\_cell()¶
:   Refresh the current cell.

lib.frontend.notebook.save\_nb()¶
:   Save the current state of the notebook (including the widgets).

## lib.frontend.process module¶

Module for process widgets.

lib.frontend.process.display\_widgets\_process(*expt*)¶
:   Set and display the widgets for choosing a process on a selected scan.

    Parameters
    :   **expt** (*object*) – Object of the class Experiment.

## lib.frontend.scan module¶

Module defining the class Scan.

*class* lib.frontend.scan.Scan¶
:   Bases: `object`

    Class defining a Scan.

    path\_to\_nxs¶
    :   Path to the nexus file, e.g. user/SIRIUS\_2020\_03\_12\_0756.nxs.

        Type
        :   str

    nxs\_name¶
    :   Nexus name, e.g. SIRIUS\_2020\_03\_12\_0756.nxs.

        Type
        :   str

    name¶
    :   Name of the scan, as is in the logs, e.g. SIRIUS\_2020\_03\_12\_0756.

        Type
        :   str

    command¶
    :   Command corresponding to the scan, if found in the logs.

        Type
        :   str or None

    set\_command(*path\_to\_logs\_dir*, *list\_logs*)¶
    :   Find the command corresponding to the scan in the logs,
        and set it to the attribute command.

        Parameters
        :   - **path\_to\_logs\_dir** (*str*) – Path to the logs files directory, e.g. user/logs/.
            - **list\_logs** (*list of str*) – List of log filenames in the logs folder.

    set\_identifiers(*path\_to\_nxs\_dir*, *nxs\_name*)¶
    :   Set identifiers to the scan (path, name …).

        Parameters
        :   - **path\_to\_nxs\_dir** (*str*) – Path to the nexus files directory, e.g. user/.
            - **nxs\_name** (*str*) – Nexus name, e.g. SIRIUS\_2020\_03\_12\_0756.nxs.

## Module contents¶

### Table of Contents

- lib.frontend package
  - Subpackages
  - Submodules
  - lib.frontend.action module
  - lib.frontend.experiment module
  - lib.frontend.form module
  - lib.frontend.jlb\_io module
  - lib.frontend.notebook module
  - lib.frontend.process module
  - lib.frontend.scan module
  - Module contents

### This Page

- Show Source

### Quick search

### Navigation

- index
- modules |
- JupyLabBook v3.0 documentation »
- lib.frontend package

© Copyright 2022, Hemmerle Arnaud.
Created using Sphinx 5.0.2.
