# Supplementary material for: Opportunities and new developments for the study of surfaces and interfaces in soft condensed matter at the SIRIUS beamline of Synchrotron SOLEIL
Source: J Synchrotron Radiat. 2024 Jan 1;31(Pt 1):162–76. doi: 10.1107/S1600577523008810 (PMC10833424; doi:10.1107/S1600577523008810)
Supplement: Supplementary file 1 [file s-31-00162-sup1.zip › JupyLabBook-v3.0.2/docs/sphinx/build/html/lib.frontend.process_widgets.html]

lib.frontend.process\_widgets package — JupyLabBook v3.0 documentation

### Navigation

- index
- modules |
- JupyLabBook v3.0 documentation »
- lib.frontend.process\_widgets package

# lib.frontend.process\_widgets package¶

## Submodules¶

## lib.frontend.process\_widgets.warea\_detector module¶

Module for area detector widgets.

lib.frontend.process\_widgets.warea\_detector.display\_widgets\_area\_detector(*expt*)¶
:   Set and display the widgets for processing area detector.

    Parameters
    :   **expt** (*object*) – Object of the class Experiment.

## lib.frontend.process\_widgets.wdata\_1d module¶

Module for data 1D widgets.

lib.frontend.process\_widgets.wdata\_1d.display\_interactive\_1d\_plot(*scan*)¶
:   Extract the 0D sensors from the nxs file and display an interactive 1D plot.

    Parameters
    :   **scan** (*object*) – Object of the class Scan.

    Raises
    :   **SystemExit****(****'Could not open Nexus file.'****)****)** – If the Nexus file cannot be accessed.

lib.frontend.process\_widgets.wdata\_1d.display\_widgets\_1d\_fit(*expt*, *function\_name*)¶
:   Set and display the widgets for fitting 1d data with a predefined function.
    So far, no widget is required and this function directly creates the cell.
    This function leaves the possibility to add future intermediate widgets.

    Parameters
    :   - **expt** (*object*) – Object of the class Experiment.
        - **function\_name** (*str*) – Predefined function to be used for the fit, e.g. ‘gaussian’ or ‘erf’.

lib.frontend.process\_widgets.wdata\_1d.display\_widgets\_data\_1d(*expt*)¶
:   Set and display the widgets for processing 1d data.
    So far, no widget is required and this function directly creates the cell.
    This function leaves the possibility to add future intermediate widgets.

    Parameters
    :   **expt** (*object*) – Object of the class Experiment.

lib.frontend.process\_widgets.wdata\_1d.display\_widgets\_energy\_calib(*expt*)¶
:   Set and display the widgets for energy calibration with standards.

    Parameters
    :   **expt** (*object*) – Object of the class Experiment.

    Raises
    :   **SystemExit****(****'Could not open Nexus file.'****)****)** – If the Nexus file cannot be accessed.

## lib.frontend.process\_widgets.wgixd module¶

Module for GIXD widgets.

lib.frontend.process\_widgets.wgixd.display\_widgets\_calib\_thetaz()¶
:   Set and display the widgets for calibration of theta z for GIXD.
    So far, no widget is required and this function directly creates the cell.
    This function leaves the possibility to add future intermediate widgets.

lib.frontend.process\_widgets.wgixd.display\_widgets\_gixd(*expt*)¶
:   Set and display the widgets for processing GIXD.

    Parameters
    :   **expt** (*object*) – Object of the class Experiment.

lib.frontend.process\_widgets.wgixd.display\_widgets\_vineyard(*expt*)¶
:   Set and display the widgets for extracting the Vineyard peak.
    So far, no widget is required and this function directly creates the cell.
    This function leaves the possibility to add future intermediate widgets.

    Parameters
    :   **expt** (*object*) – Object of the class Experiment.

## lib.frontend.process\_widgets.wgixs module¶

Module for GIXS widgets.

lib.frontend.process\_widgets.wgixs.display\_widgets\_gixs(*expt*)¶
:   Set and display the widgets for processing GIXS.

    Parameters
    :   **expt** (*object*) – Object of the class Experiment.

## lib.frontend.process\_widgets.wisotherm module¶

Module for isotherm widgets.

lib.frontend.process\_widgets.wisotherm.display\_widgets\_isotherm(*expt*)¶
:   Set and display the widgets for processing isotherms.

    Parameters
    :   **expt** (*object*) – Object of the class Experiment.

## lib.frontend.process\_widgets.wxrf module¶

Module for XRF widgets.

lib.frontend.process\_widgets.wxrf.display\_widgets\_identify\_peaks(*expt*, *dw*)¶
:   Prepare and display the widgets for identification of peaks.

    Parameters
    :   - **expt** (*object*) – Object of the class Experiment.
        - **dw** (*dict*) – Dictionnary of widgets.

lib.frontend.process\_widgets.wxrf.display\_widgets\_xrf(*expt*)¶
:   Set and display the widgets for processing XRF.

    Parameters
    :   **expt** (*object*) – Object of the class Experiment.

## lib.frontend.process\_widgets.wxrr module¶

Module for XRR widgets (on solids and liquids).

lib.frontend.process\_widgets.wxrr.display\_widgets\_calib\_xrr\_liquid(*expt*)¶
:   Set and display the widgets for calibration of XRR on liquid.

    Parameters
    :   **expt** (*object*) – Object of the class Experiment.

lib.frontend.process\_widgets.wxrr.display\_widgets\_xrr\_liquid(*expt*)¶
:   Set and display the widgets for processing XRR on a liquid.

    Parameters
    :   **expt** (*object*) – Object of the class Experiment.

lib.frontend.process\_widgets.wxrr.display\_widgets\_xrr\_solid(*expt*)¶
:   Set and display the widgets for processing XRR on a solid.

    Parameters
    :   **expt** (*object*) – Object of the class Experiment.

lib.frontend.process\_widgets.wxrr.preview\_direct\_xrr(*expt*)¶
:   Extract and display the ROI of the direct beam.

    Parameters
    :   **expt** (*object*) – Object of the class Experiment.

lib.frontend.process\_widgets.wxrr.preview\_indiv\_scan\_xrr(*nxs\_name*, *expt*)¶
:   Extract and display the ROIs, for interactive preview.

    Parameters
    :   - **nxs\_name** (*str*) – Nexus name, e.g. SIRIUS\_2020\_03\_12\_0756.nxs.
        - **expt** (*object*) – Object of the class Experiment.

## Module contents¶

### Table of Contents

- lib.frontend.process\_widgets package
  - Submodules
  - lib.frontend.process\_widgets.warea\_detector module
  - lib.frontend.process\_widgets.wdata\_1d module
  - lib.frontend.process\_widgets.wgixd module
  - lib.frontend.process\_widgets.wgixs module
  - lib.frontend.process\_widgets.wisotherm module
  - lib.frontend.process\_widgets.wxrf module
  - lib.frontend.process\_widgets.wxrr module
  - Module contents

### This Page

- Show Source

### Quick search

### Navigation

- index
- modules |
- JupyLabBook v3.0 documentation »
- lib.frontend.process\_widgets package

© Copyright 2022, Hemmerle Arnaud.
Created using Sphinx 5.0.2.
