# Supplementary material for: Opportunities and new developments for the study of surfaces and interfaces in soft condensed matter at the SIRIUS beamline of Synchrotron SOLEIL
Source: J Synchrotron Radiat. 2024 Jan 1;31(Pt 1):162–76. doi: 10.1107/S1600577523008810 (PMC10833424; doi:10.1107/S1600577523008810)
Supplement: Supplementary file 1 [file s-31-00162-sup1.zip › JupyLabBook-v3.0.2/docs/sphinx/build/html/lib.html]

lib package — JupyLabBook v3.0 documentation

### Navigation

- index
- modules |
- JupyLabBook v3.0 documentation »
- lib package

# lib package¶

## Subpackages¶

- lib.backend package
  - Submodules
  - lib.backend.PyNexus module
  - lib.backend.area\_detector module
  - lib.backend.data\_1d module
  - lib.backend.gixd module
  - lib.backend.gixs module
  - lib.backend.isotherm module
  - lib.backend.xrf module
  - lib.backend.xrr module
  - Module contents
- lib.frontend package
  - Subpackages
    - lib.frontend.process\_widgets package
      - Submodules
      - lib.frontend.process\_widgets.warea\_detector module
      - lib.frontend.process\_widgets.wdata\_1d module
      - lib.frontend.process\_widgets.wgixd module
      - lib.frontend.process\_widgets.wgixs module
      - lib.frontend.process\_widgets.wisotherm module
      - lib.frontend.process\_widgets.wxrf module
      - lib.frontend.process\_widgets.wxrr module
      - Module contents
  - Submodules
  - lib.frontend.action module
  - lib.frontend.experiment module
  - lib.frontend.form module
  - lib.frontend.jlb\_io module
  - lib.frontend.notebook module
  - lib.frontend.process module
  - lib.frontend.scan module
  - Module contents

## Submodules¶

## lib.jupylabbook module¶

Custom module called by JupyLabBook.ipynb.
Coordinates the different sub-modules.
The object experiment containing all the attributes relevant to the current experiment is defined here.

lib.jupylabbook.display\_action()¶
:   Prepare and display the widgets for selecting the next action.

lib.jupylabbook.display\_process()¶
:   Prepare and display the widgets for processing the selected scans.

lib.jupylabbook.print\_version(*version*)¶
:   Print the version of JupyLabBook and a link to the main repo.

    Parameters
    :   **version** (*str*) – Version of JupyLabBook vX.Y.Z with X major, Y minor, Z patch.

lib.jupylabbook.start(*paths*)¶
:   Check that the paths are ok and display the action widgets.

    Parameters
    :   **paths** (*dict*) – Dictionary of all the required paths to specific folders and files.

    Raises
    :   **FileNotFoundError** – If a file or a folder is missing.

## Module contents¶

### Table of Contents

- lib package
  - Subpackages
  - Submodules
  - lib.jupylabbook module
  - Module contents

### This Page

- Show Source

### Quick search

### Navigation

- index
- modules |
- JupyLabBook v3.0 documentation »
- lib package

© Copyright 2022, Hemmerle Arnaud.
Created using Sphinx 5.0.2.
