# Supplementary material for: Opportunities and new developments for the study of surfaces and interfaces in soft condensed matter at the SIRIUS beamline of Synchrotron SOLEIL
Source: J Synchrotron Radiat. 2024 Jan 1;31(Pt 1):162–76. doi: 10.1107/S1600577523008810 (PMC10833424; doi:10.1107/S1600577523008810)
Supplement: Supplementary file 1 [file s-31-00162-sup1.zip › JupyLabBook-v3.0.2/docs/sphinx/build/html/modules.html]

lib — JupyLabBook v3.0 documentation

### Navigation

- index
- modules |
- JupyLabBook v3.0 documentation »
- lib

# lib¶

- lib package
  - Subpackages
    - lib.backend package
      - Submodules
      - lib.backend.PyNexus module
      - lib.backend.area\_detector module
      - lib.backend.data\_1d module
      - lib.backend.gixd module
      - lib.backend.gixs module
      - lib.backend.isotherm module
      - lib.backend.xrf module
      - lib.backend.xrr module
      - Module contents
    - lib.frontend package
      - Subpackages
      - Submodules
      - lib.frontend.action module
      - lib.frontend.experiment module
      - lib.frontend.form module
      - lib.frontend.jlb\_io module
      - lib.frontend.notebook module
      - lib.frontend.process module
      - lib.frontend.scan module
      - Module contents
  - Submodules
  - lib.jupylabbook module
  - Module contents

### This Page

- Show Source

### Quick search

### Navigation

- index
- modules |
- JupyLabBook v3.0 documentation »
- lib

© Copyright 2022, Hemmerle Arnaud.
Created using Sphinx 5.0.2.
