# Supplementary material for: Opportunities and new developments for the study of surfaces and interfaces in soft condensed matter at the SIRIUS beamline of Synchrotron SOLEIL
Source: J Synchrotron Radiat. 2024 Jan 1;31(Pt 1):162–76. doi: 10.1107/S1600577523008810 (PMC10833424; doi:10.1107/S1600577523008810)
Supplement: Supplementary file 1 [file s-31-00162-sup1.zip › JupyLabBook-v3.0.2/docs/sphinx/build/html/py-modindex.html]

Python Module Index — JupyLabBook v3.0 documentation

### Navigation

- index
- modules |
- JupyLabBook v3.0 documentation »
- Python Module Index

# Python Module Index

**l**

|  |  |  |
| --- | --- | --- |
|  |  |  |
|  | **l** |  |
|  | `lib` |  |
|  | `lib.backend` |  |
|  | `lib.backend.area_detector` |  |
|  | `lib.backend.data_1d` |  |
|  | `lib.backend.gixd` |  |
|  | `lib.backend.gixs` |  |
|  | `lib.backend.isotherm` |  |
|  | `lib.backend.PyNexus` |  |
|  | `lib.backend.xrf` |  |
|  | `lib.backend.xrr` |  |
|  | `lib.frontend` |  |
|  | `lib.frontend.action` |  |
|  | `lib.frontend.experiment` |  |
|  | `lib.frontend.form` |  |
|  | `lib.frontend.jlb_io` |  |
|  | `lib.frontend.notebook` |  |
|  | `lib.frontend.process` |  |
|  | `lib.frontend.process_widgets` |  |
|  | `lib.frontend.process_widgets.warea_detector` |  |
|  | `lib.frontend.process_widgets.wdata_1d` |  |
|  | `lib.frontend.process_widgets.wgixd` |  |
|  | `lib.frontend.process_widgets.wgixs` |  |
|  | `lib.frontend.process_widgets.wisotherm` |  |
|  | `lib.frontend.process_widgets.wxrf` |  |
|  | `lib.frontend.process_widgets.wxrr` |  |
|  | `lib.frontend.scan` |  |
|  | `lib.jupylabbook` |  |

### Quick search

### Navigation

- index
- modules |
- JupyLabBook v3.0 documentation »
- Python Module Index

© Copyright 2022, Hemmerle Arnaud.
Created using Sphinx 5.0.2.
