# Supplementary material for: Opportunities and new developments for the study of surfaces and interfaces in soft condensed matter at the SIRIUS beamline of Synchrotron SOLEIL
Source: J Synchrotron Radiat. 2024 Jan 1;31(Pt 1):162–76. doi: 10.1107/S1600577523008810 (PMC10833424; doi:10.1107/S1600577523008810)
Supplement: Supplementary file 1 [file s-31-00162-sup1.zip › JupyLabBook-v3.0.2/docs/sphinx/build/html/search.html]

Search — JupyLabBook v3.0 documentation

### Navigation

- index
- modules |
- JupyLabBook v3.0 documentation »
- Search

# Search

Please activate JavaScript to enable the search
functionality.

Searching for multiple words only shows matches that contain
all words.

### Navigation

- index
- modules |
- JupyLabBook v3.0 documentation »
- Search

© Copyright 2022, Hemmerle Arnaud.
Created using Sphinx 5.0.2.
