# Supplementary material for: Opportunities and new developments for the study of surfaces and interfaces in soft condensed matter at the SIRIUS beamline of Synchrotron SOLEIL
Source: J Synchrotron Radiat. 2024 Jan 1;31(Pt 1):162–76. doi: 10.1107/S1600577523008810 (PMC10833424; doi:10.1107/S1600577523008810)
Supplement: Supplementary file 1 [file s-31-00162-sup1.zip › JupyLabBook-v3.0.2/docs/example.zip!/example/JupyLabBook_example.pdf]

JupyLabBook_example

January 27, 2023

1 JupyLabBook @ SIRIUS

Contents

1 JupyLabBook @ SIRIUS **1**

2 Experimental setup **3**

3 Beamline alignment **4**
3.1 Subsection 4
3.1.1 SIRIUS_2020_03_11_0744: dscan basez -.2 .2 50 .1 4
3.1.2 SIRIUS_2020_03_11_0752: continuous_ascan delta -.15 .15 100 1 5
3.2 Calibration thetaz 6
3.3 Calibration energy 6
3.3.1 SIRIUS_2022_03_29_0374: ascan energydcm 8.2 8.6 400 .1 6

4 GIXD **7**
4.1 Yoneda peak 7
4.1.1 SIRIUS_2020_03_12_0756: continuous_ascan delta -24 -19 100 5 7
4.2 Process GIXD 8
4.2.1 SIRIUS_2020_03_12_0756: continuous_ascan delta -24 -19 100 5 9

5 Isotherm **10**
5.0.1 SIRIUS_Isotherm_2019_02_17_01544: isotherm 1.97 46 35000 1 10

6 1D plot **11**
6.0.1 SIRIUS_2020_03_11_0744: dscan basez -.2 .2 50 .1 11

7 GIXS **12**
7.1 WAXS 12
7.1.1 SIRIUS_2021_11_26_6088: tscan 5 5 12
7.2 GIWAXS 13
7.2.1 SIRIUS_2021_11_26_6103: tscan 10 10 13
7.3 GISAXS 13
7.3.1 SIRIUS_2021_10_16_2739: run scan_0_18.ipy 13

8 Plot area detector **14**
8.1 Pilatus 14
8.1.1 SIRIUS_2021_11_26_6088: tscan 5 5 14

8.2 UFXC . . . 15
8.2.1 SIRIUS__2021__11__11__3728: tscan 2 9 . . . 15

9 XRF **16**
9.1 SDD 4-elements . . . 16
9.1.1 SIRIUS__2017__12__11__08042: run xsw7.ipy . . . 16
9.2 SDD 1-element . . . 18
9.2.1 SIRIUS__Fluo__2020__07__03__0042: tscan 500 30 . . . 18

10 XRR **20**
10.1 Calibration XRR (liquid) . . . 20
10.2 Scan XRR (liquid) . . . 21
10.2.1 SIRIUS__2021__04__14__4298 . . . 21
10.3 Scan XRR (solid) . . . 23
10.3.1 SIRIUS__2022__02__23__4081: run scan__XRR.ipy . . . 23

11 Insert script **24**
11.0.1 script__with__loop.ipy . . . 24

12 Insert positions **25**
12.0.1 wm delta gamma . . . 25
12.0.2 %wm diffracto . . . 25

13 Insert commands **26**

14 Save/load params **26**

15 Export **26**

```
<IPython.core.display.Javascript object>
```

```
JupyLabBook version: v3.0.2
More info on: https://gitlab.com/soleil-data-treatment/soleil-beamlines/soleil-
beamline-sirius/JupyLabBook

Data reduction will be saved in the folder:
	data/
The nexus files should be in the folder:
	user/
The logs should be in the folder:
	user/logs/
The params should be in the folder:
	../../lib/params/
The scripts should be in the folder:
	user/scripts/
Notebook name:
	JupyLabBook_example.ipynb
Default parameters loaded.
```

```
<IPython.core.display.Javascript object>
```

2 Experimental setup

SIRIUS Beamline : Experiment 1234

Example

- Type: Proposal
- Safety: Yellow
- Date: 11/06/21-12/06/21

- Main proposer: Hemmerle
- Local contact: Arnaud
- Users (on site): AH

- Machine:
  - Current: 450 mA
  - Mode: Top-up

- Optics:
  - DCM: Si111
  - MGM: Not used
  - M1: M1-A Pt Track
  - M2: M2 Pt Track
  - M3: No M3
  - M4: M4 Pt Track

- Beam:
  - Fixed/Variable energy: Fixed

  - Energy (keV): 8
  - Wavelength (nm): 0.155
  - Harmonic: 12
  - Polarisation: LH
  - Phase (deg): 0
  - Horizontal focalisation: False
  - Vertical focalisation: True
  - Horizontal beamsize (mm): 2
  - Vertical beamsize (mm): 0.05

- Monitors and XBPM:
  - mon1:
  - mon2: thick diamond
  - mon3:
  - mon4:
  - Detectors: Pilatus on delta/gamma.

- Remarks: This is an example.

3 Beamline alignment

Here we show functions used during beamline alignment.

LaTeX formula can be used in the text:

$$\frac{786 - 558}{2 \times 2069} \times 0.0355 = 1.9 mrad$$

3.1 Subsection

Fit with erf function.

3.1.1 SIRIUS_2020_03_11_0744: dscan basez -.2 .2 50 .1

```
Absorbers: No Absorbers
Starting time: 2020-03-11 16:52:56.910030
Ending time: 2020-03-11 16:53:42.791704
```

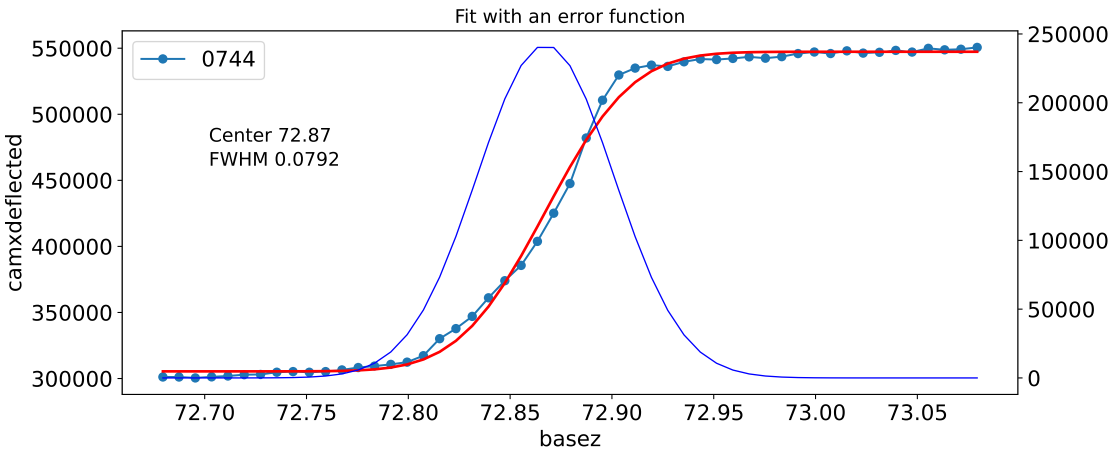

Fit with a gaussian.

3.1.2 SIRIUS_2020_03_11_0752: continuous_ascan delta -.15 .15 100 1

```
Absorbers: Al 1000micron
Starting time: 2020-03-11 18:41:13.266293
Ending time: 2020-03-11 18:44:06.972633
```

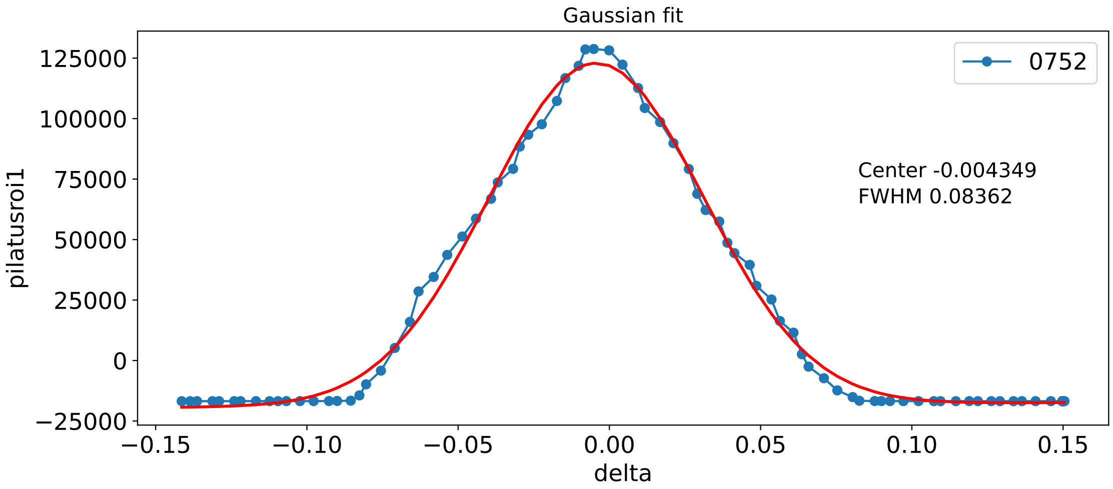

3.2 Calibration thetaz

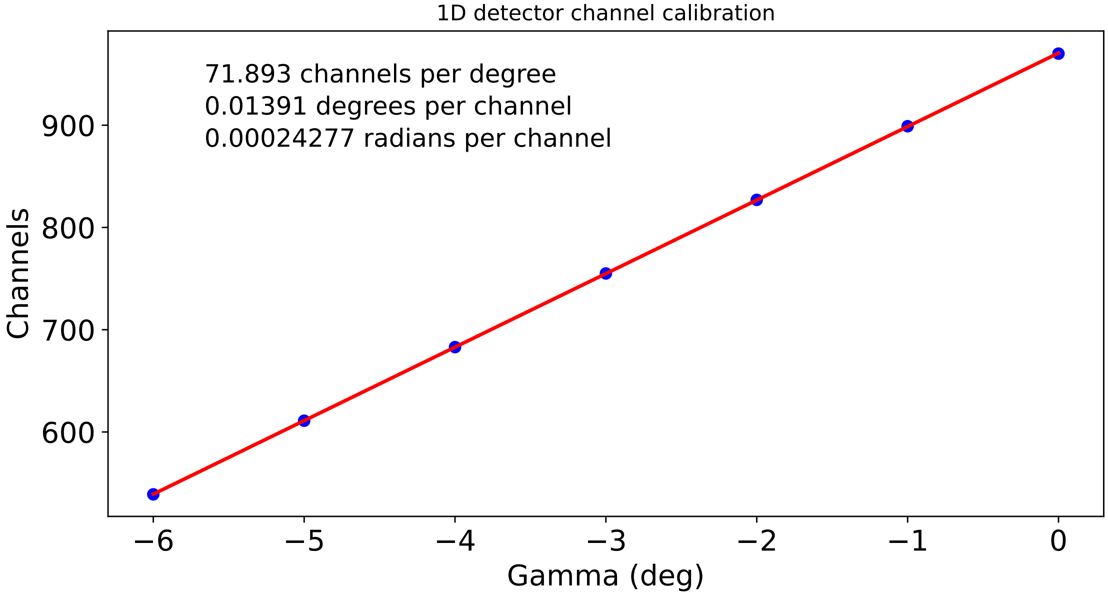

3.3 Calibration energy

3.3.1 SIRIUS_2022_03_29_0374: ascan energydcm 8.2 8.6 400 .1

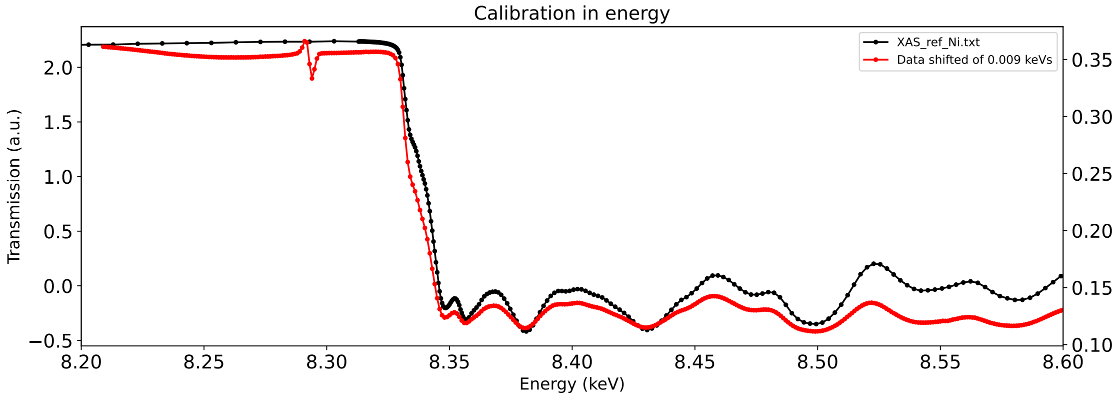


```
Current value of braggdcm = 13.7745
Current value of energydcm = 8.333
Do at the current energy: setposition braggdcm 13.7593
```

4 GIXD

4.1 Yoneda peak

Extraction of the Yoneda-Vineyard peak.

4.1.1 SIRIUS_2020_03_12_0756: continuous_ascan delta -24 -19 100 5

```
 - Open Nexus Data File :
        user/SIRIUS_2020_03_12_0756.nxs
        . Number of data points: 10
        . Available Counters:
                0  -------> delta
                1  -------> zs
                2  -------> gamma
                3  -------> hu36energy
                4  -------> xs
                5  -------> energydcm
                6  -------> current
                7  -------> mon2
                8  -------> surfacepressure
                9  -------> areapermolecule
                10  -------> qxy
                11  -------> pilatus
                12  -------> pilatusroi1
                13  -------> integration_time
                14  -------> sensorsRelTimestamps
                15  -------> sensorsTimestamps
        . Pilatus data found, (column 11, alias pilatus)
        . qxy data found, (column 10, alias qxy)
        . Valid data between points 0 and 9
        . Surface pressure data found, mean value 19.73 ± 0.005632 mN/m
        . Area per molecule data found, mean value 0.3558 ± 1.942e-05 nm2 per
molecule
        . Gamma motor data found, mean value -0.0005415 deg
Starting time: 2020-03-12 11:16:03.090248
Ending time: 2020-03-12 11:16:51.901126
```

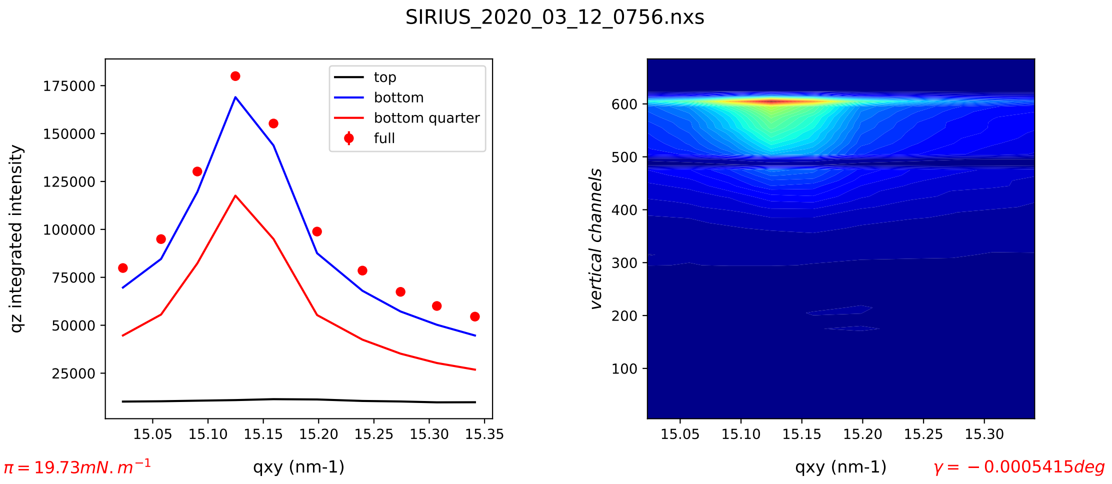

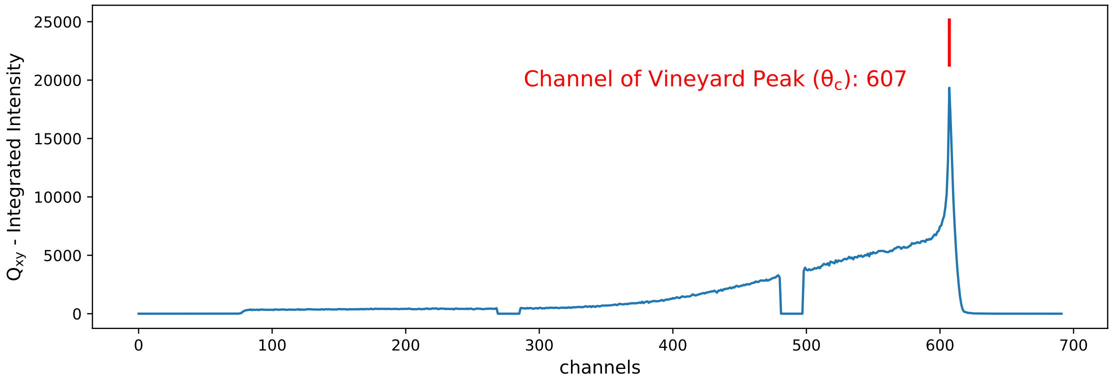


```
Data not saved. To save data, run a GIXD on the scan.
Channel0: 607
```

4.2 Process GIXD

Classic GIXD with:

$$q_{xy} = \frac{4\pi}{\lambda} \sin\left(\frac{2\theta}{2}\right)$$

Generates:
- SIRIUS_2020_03_12_0756_1D_qz.dat for each binning
- SIRIUS_2020_03_12_0756_1D.dat
- SIRIUS_2020_03_12_0756_1D.mat for each binning
- SIRIUS_2020_03_12_0756_1D.moy for each binning

4.2.1 SIRIUS_2020_03_12_0756: continuous_ascan delta -24 -19 100 5

```
 - Open Nexus Data File :
        user/SIRIUS_2020_03_12_0756.nxs
        . Number of data points: 10
        . Available Counters:
                0  -------> delta
                1  -------> zs
                2  -------> gamma
                3  -------> hu36energy
                4  -------> xs
                5  -------> energydcm
                6  -------> current
                7  -------> mon2
                8  -------> surfacepressure
                9  -------> areapermolecule
                10  -------> qxy
                11  -------> pilatus
                12  -------> pilatusroi1
                13  -------> integration_time
                14  -------> sensorsRelTimestamps
                15  -------> sensorsTimestamps
        . Pilatus data found, (column 11, alias pilatus)
        . qxy data found, (column 10, alias qxy)
        . Valid data between points 0 and 9
        . Surface pressure data found, mean value 19.73 ± 0.005632 mN/m
        . Area per molecule data found, mean value 0.3558 ± 1.942e-05 nm2 per
molecule
        . Gamma motor data found, mean value -0.0005415 deg
Absorbers: 29 - Vide
Starting time: 2020-03-12 11:16:03.090248
Ending time: 2020-03-12 11:16:51.901126
```

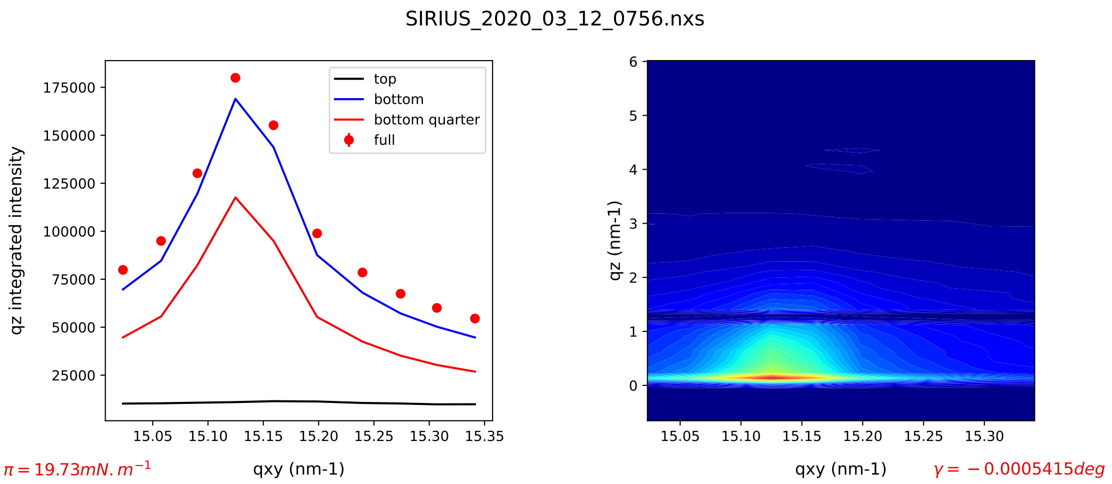

```
. Original, non binned, matrix saved in:
data/SIRIUS_2020_03_12_0756_1D.mat
. Scalar data saved in:
data/SIRIUS_2020_03_12_0756_1D.dat
. qz values saved in:
data/SIRIUS_2020_03_12_0756_1D_qz.dat10
. Binned matrix saved in:
data/SIRIUS_2020_03_12_0756_1D.mat10
. XYZ data saved in:
data/SIRIUS_2020_03_12_0756_1D.moy10
. qz values saved in:
data/SIRIUS_2020_03_12_0756_1D_qz.dat20
. Binned matrix saved in:
data/SIRIUS_2020_03_12_0756_1D.mat20
. XYZ data saved in:
data/SIRIUS_2020_03_12_0756_1D.moy20
. qz values saved in:
data/SIRIUS_2020_03_12_0756_1D_qz.dat40
. Binned matrix saved in:
data/SIRIUS_2020_03_12_0756_1D.mat40
. XYZ data saved in:
data/SIRIUS_2020_03_12_0756_1D.moy40
. Figure saved in:
data/SIRIUS_2020_03_12_0756_1D.pdf
```

5 Isotherm

5.0.1 SIRIUS_Isotherm_2019_02_17_01544: isotherm 1.97 46 35000 1

```
Starting time: 2019-02-17 16:52:00.519142
Ending time: 2019-02-17 17:43:46.021957
```

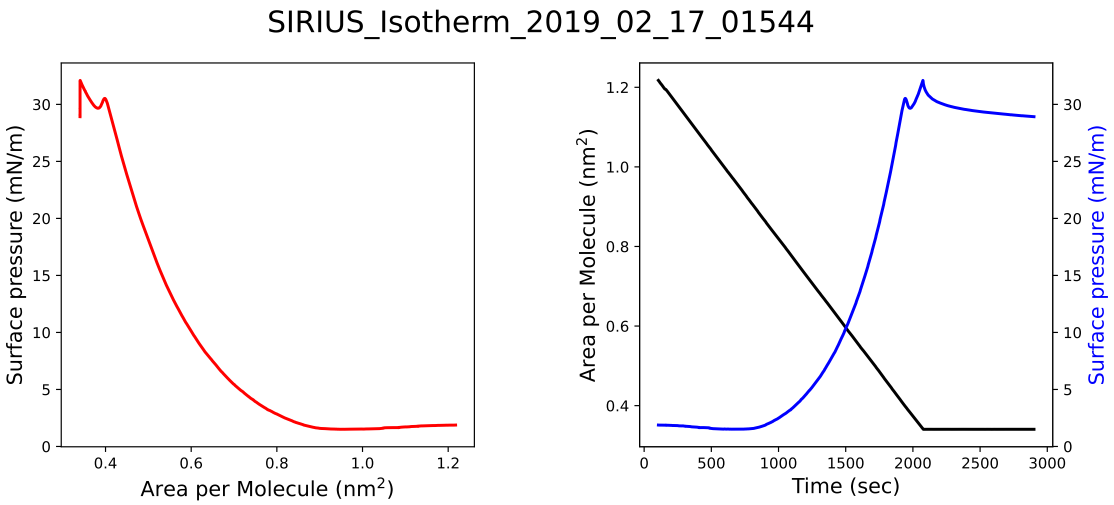

6 1D plot

Add a 1D plot by clicking on `Add plot to report`.

Generates SIRIUS_2020_03_11_0744.dat

6.0.1 SIRIUS_2020_03_11_0744: dscan basez -.2 .2 50 .1

```
Absorbers: No Absorbers
Starting time: 2020-03-11 16:52:56.910030
Ending time: 2020-03-11 16:53:42.791704
```

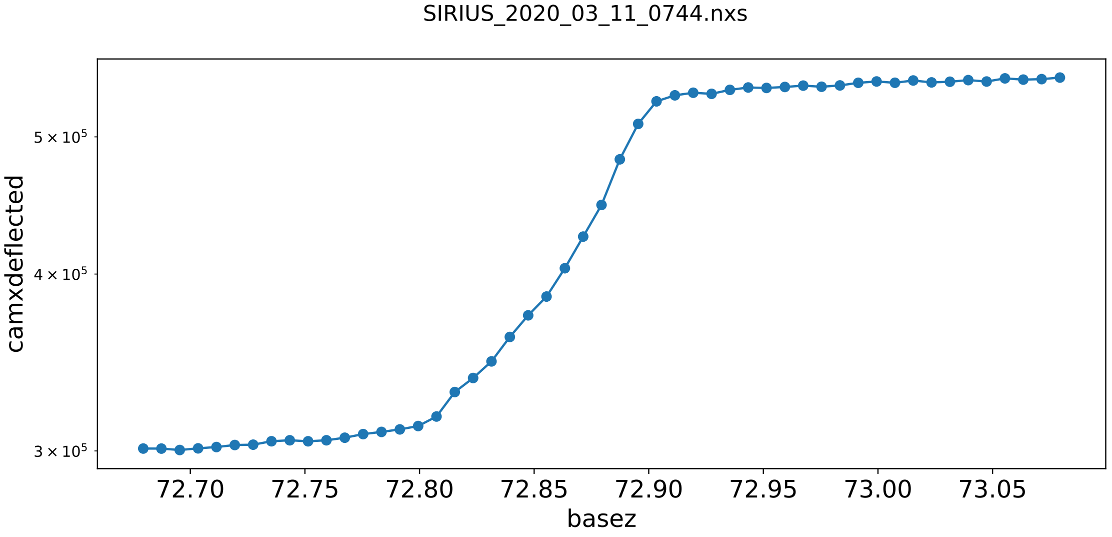

7 GIXS

GIXS: q_z vs q_{xy}.
Image and profiles with the approximation $q_{xy} = \frac{4\pi}{\lambda}\sin\left(\frac{2\theta}{2}\right)$.
Generates:
- SIRIUS_2021_11_26_6088_pilatus_sum.tiff
- SIRIUS_2021_11_26_6088_pilatus_sum.mat
- SIRIUS_2021_11_26_6088_integrated_qz.dat
- SIRIUS_2021_11_26_6088_integrated_qxy.dat

7.1 WAXS

WAXS on Ag Behenate for calibration. Use the GIXS command with thetai forced to 0.

7.1.1 SIRIUS_2021_11_26_6088: tscan 5 5

```
Starting time: 2021-11-26 10:35:05.759991
Ending time: 2021-11-26 10:35:05.759991
gamma found: gamma = -0.9997 deg
delta found: delta = -8.875 deg
thetai is forced to the value: thetai =   0 deg
```

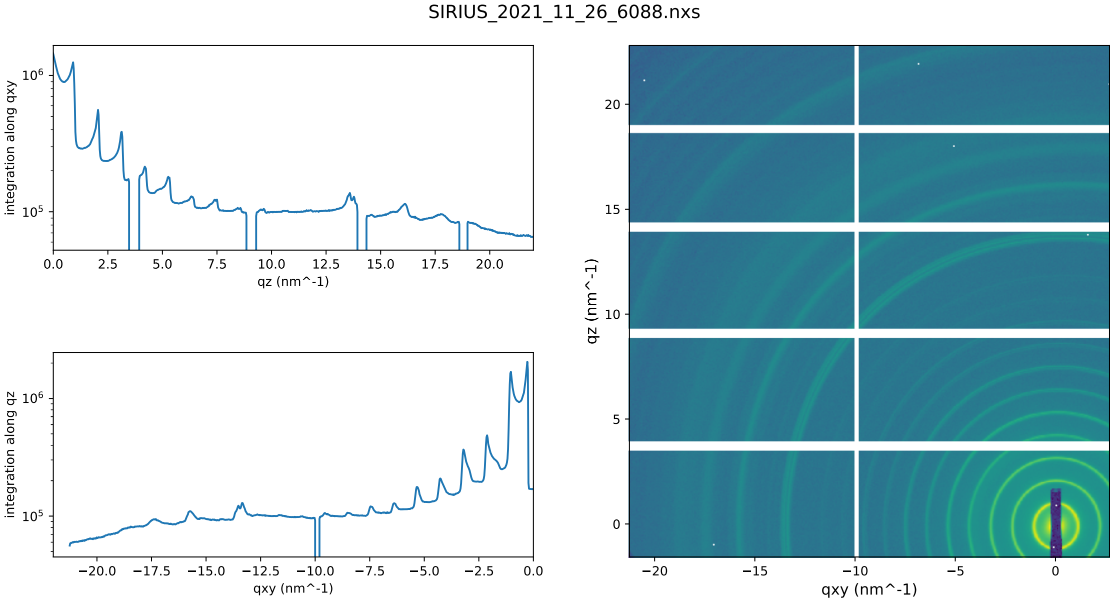

7.2 GIWAXS

GIWAXS on a P3HT film.

7.2.1 SIRIUS_2021_11_26_6103: tscan 10 10

```
Starting time: 2021-11-26 12:40:16.870662
Ending time: 2021-11-26 12:40:16.870662
gamma found: gamma =  -2 deg
delta found: delta = -8.875 deg
thetai (alphax) found: thetai = 0.1 deg
```

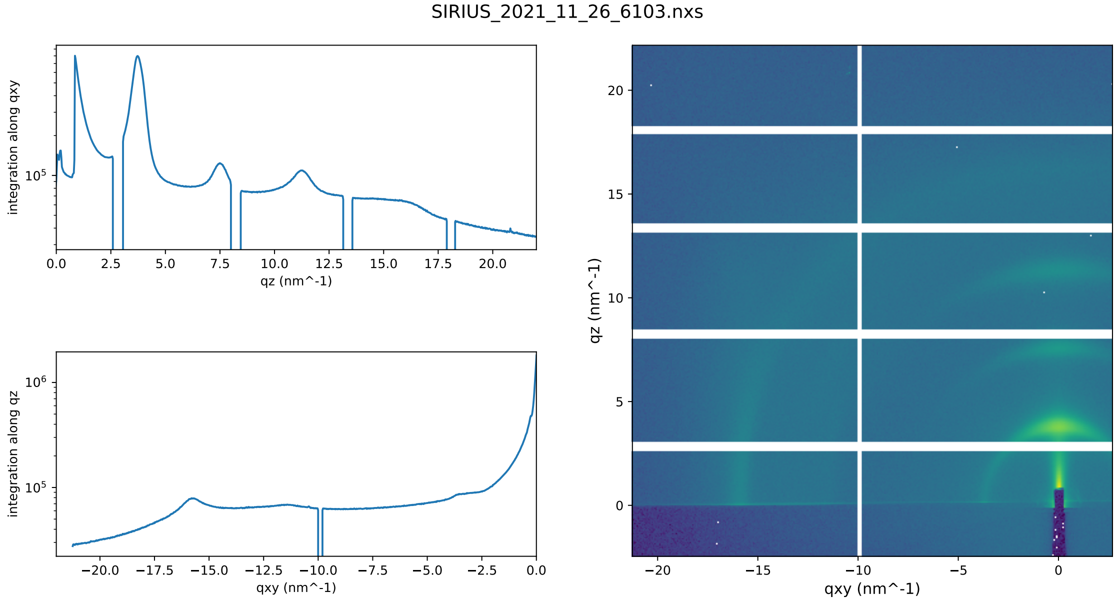

7.3 GISAXS

GISAXS image. Delta and gamma have to be forced to zero (the detector is not on the diffractometer).

7.3.1 SIRIUS_2021_10_16_2739: run scan_0_18.ipy

```
Absorbers: 29 - Vide
Starting time: 2021-10-16 18:47:21.180836
Ending time: 2021-10-16 18:47:21.180836
gamma is forced to the value: gamma =   0 deg
delta is forced to the value: delta =   0 deg
thetai (alphax) found: thetai = 0.18 deg
```

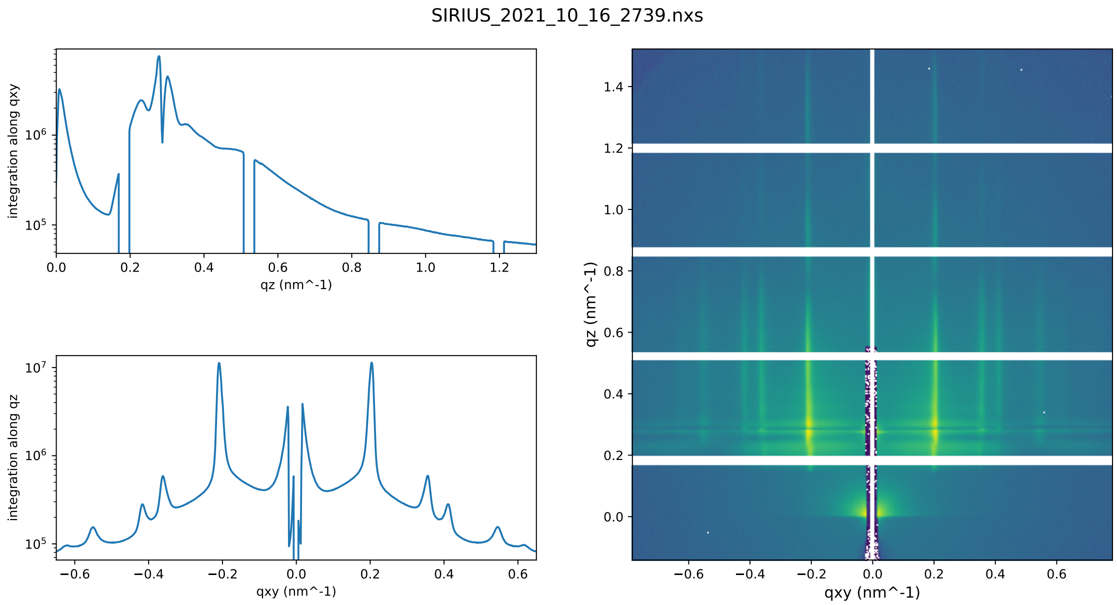

8 Plot area detector

Plot the sum of the images out of a 2D detector. Can also extract and save all the individual images if `Save each` is ticked.

8.1 Pilatus

Here with the Pilatus.
Generates:
- SIRIUS_2021_11_26_6088_pilatus_sum.tiff
- SIRIUS_2021_11_26_6088_pilatus_sum.mat
- SIRIUS_2021_11_26_6088_integrated_x.dat
- SIRIUS_2021_11_26_6088_integrated_y.dat

8.1.1 SIRIUS_2021_11_26_6088: tscan 5 5

```
Starting time: 2021-11-26 10:35:05.759991
Ending time: 2021-11-26 10:35:05.759991
```

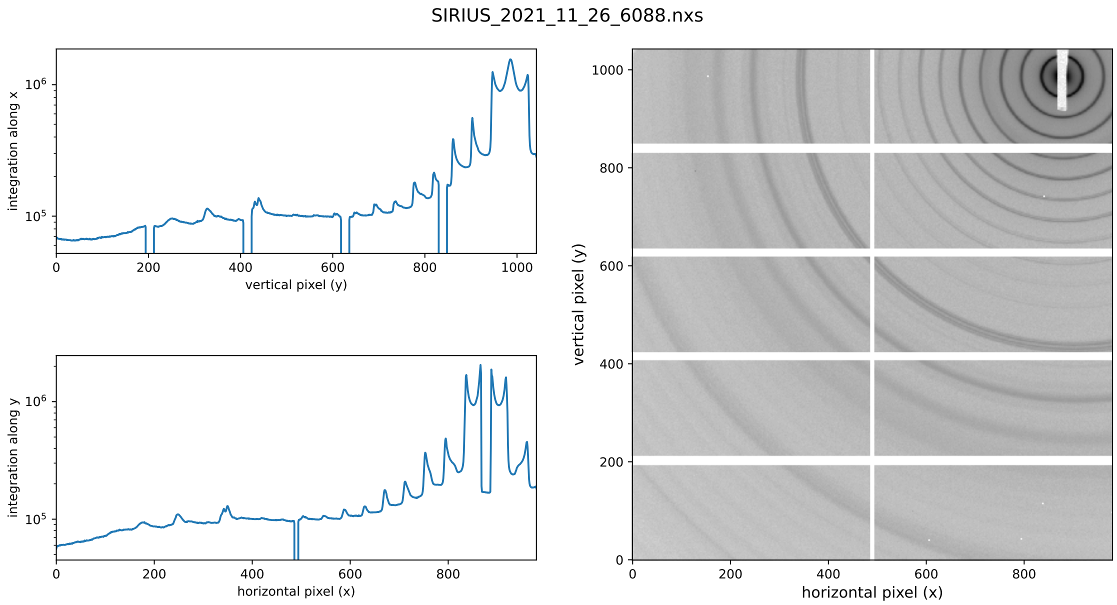

8.2 UFXC

8.2.1 SIRIUS__2021__11__11__3728: tscan 2 9

```
. Available Counters:
        0  -------> delta
        1  -------> shg
        2  -------> zs
        3  -------> alphax
        4  -------> gamma
        5  -------> xs
        6  -------> energydcm
        7  -------> svg
        8  -------> current
        9  -------> mon2
        10  -------> fluoicr00
        11  -------> fluoicr01
        12  -------> fluoicr02
        13  -------> fluoicr03
        14  -------> fluospectrum00
        15  -------> fluospectrum01
        16  -------> fluospectrum02
        17  -------> fluospectrum03
        18  -------> mon4
        19  -------> ionchamber
        20  -------> fluoocr00
```

```
                    21  -------> fluoocr01
                    22  -------> fluoocr02
                    23  -------> fluoocr03
                    24  -------> commandfemtoionchamber
                    25  -------> ufx
                    26  -------> ufxroi2
                    27  -------> integration_time
                    28  -------> sensorsRelTimestamps
                    29  -------> sensorsTimestamps
Starting time: 2021-11-11 14:37:28.024263
Ending time: 2021-11-11 14:37:46.813598
```

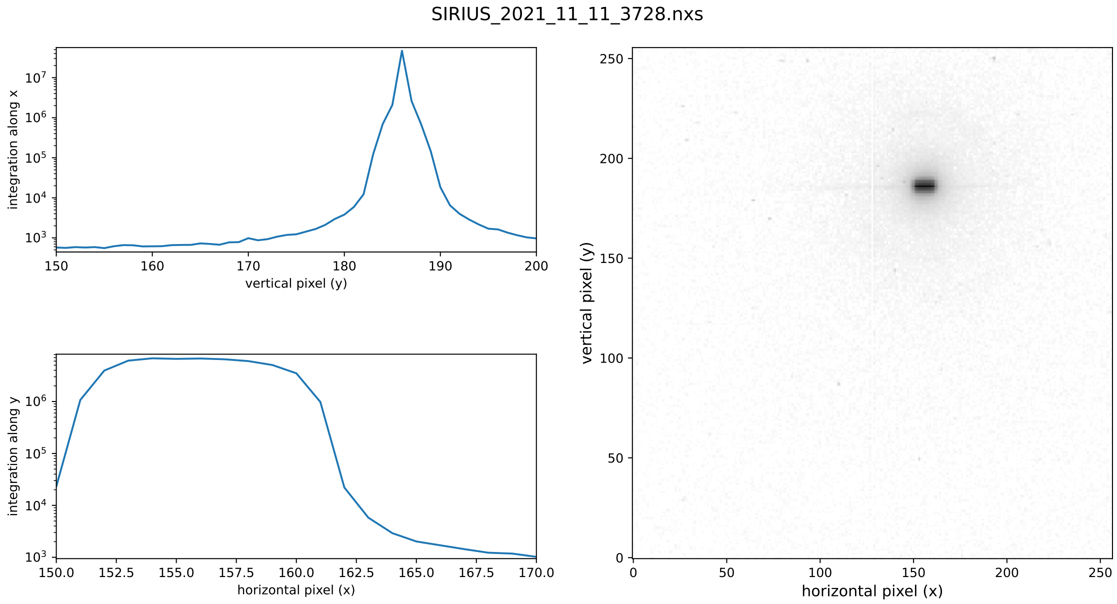

9 XRF

9.1 SDD 4-elements

Plot XRF from the 4-elements detector, in channels and without peak identification.
Generates:
- SIRIUS__2017__12__11__08042__fluospectrum.mat for each SDD element
- SIRIUS__2017__12__11__08042.dat

9.1.1 SIRIUS__2017__12__11__08042: run xsw7.ipy

```
Absorbers: Al 200micron
Starting time: 2017-12-11 06:24:58.182355
```

Ending time: 2017-12-11 06:26:26.813347

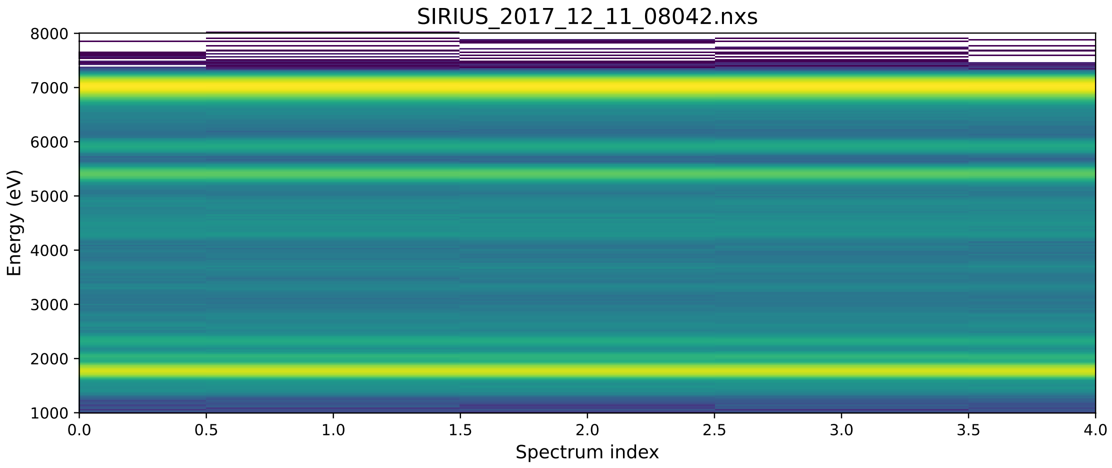

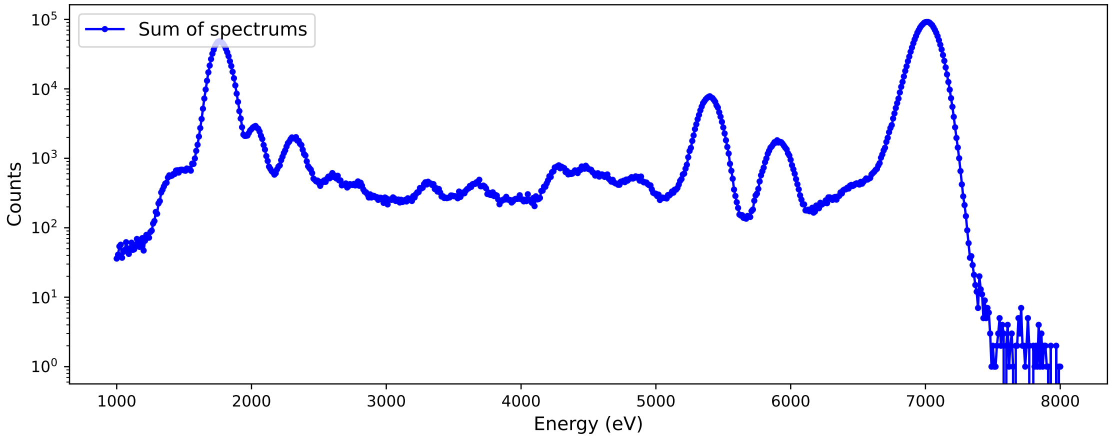

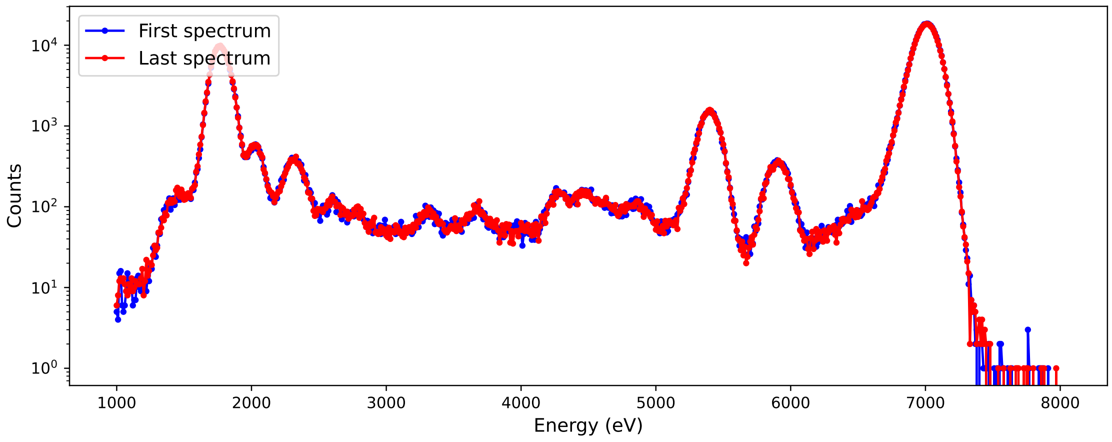

9.2 SDD 1-element

9.2.1 SIRIUS_Fluo_2020_07_03_0042: tscan 500 30

```
 - Open Nexus Data File :
        user/SIRIUS_Fluo_2020_07_03_0042.nxs
        . Number of data points: 10
        . Available Counters:
                0  -------> ys
                1  -------> zs
                2  -------> xs
                3  -------> surfacepressure
                4  -------> areapermolecule
                5  -------> fluo01
                6  -------> fluo02
                7  -------> fluo03
                8  -------> fluo04
                9  -------> fluoicr04
                10  -------> fluoocr04
                11  -------> fluospectrum04
                12  -------> qxy
                13  -------> integration_time
                14  -------> sensorsRelTimestamps
                15  -------> sensorsTimestamps
        . Valid data between points 0 and 9
Absorbers: Al 800micron
Starting time: 2020-07-03 11:01:00.235891
Ending time: 2020-07-03 11:05:34.198804
```

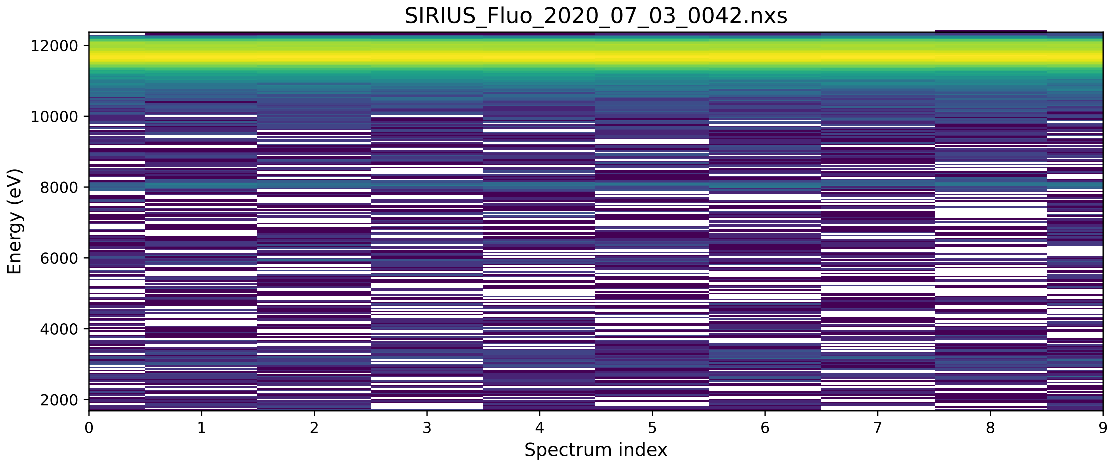

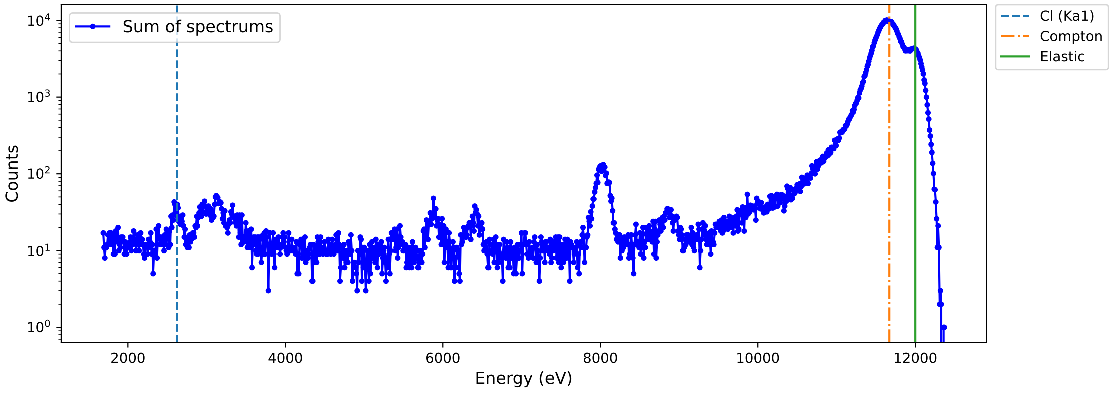

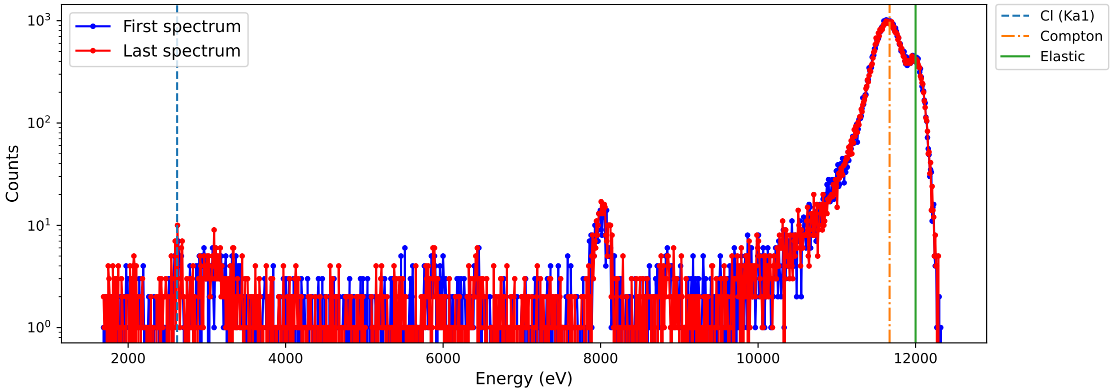


```
. Dat file saved in:
data/SIRIUS_Fluo_2020_07_03_0042.dat
. Spectrum(s) saved in:
data/SIRIUS_Fluo_2020_07_03_0042_fluospectrum*.mat
. Figure spectrogram saved in:
data/SIRIUS_Fluo_2020_07_03_0042_spectrogram.pdf
. Figure sum saved in:
data/SIRIUS_Fluo_2020_07_03_0042_sum.pdf
. Figure first/last saved in:
data/SIRIUS_Fluo_2020_07_03_0042_first_last.pdf
```

10 XRR

10.1 Calibration XRR (liquid)

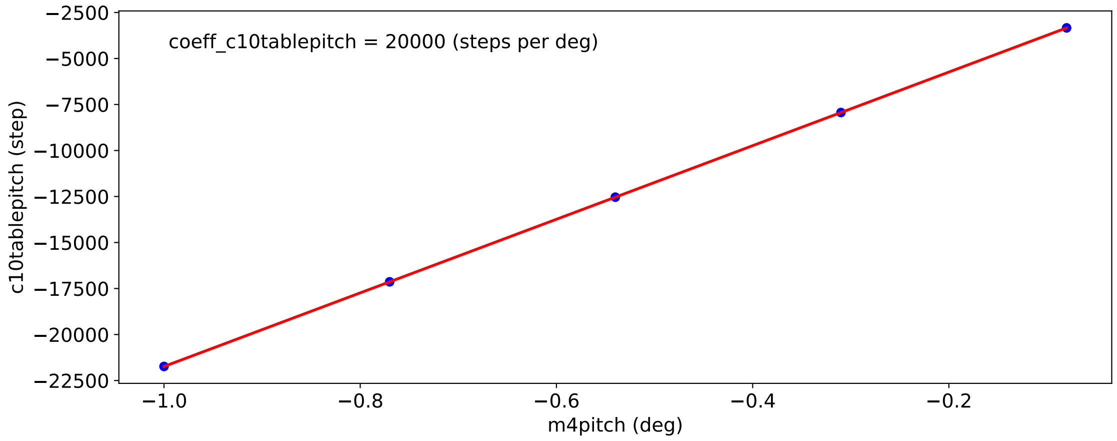

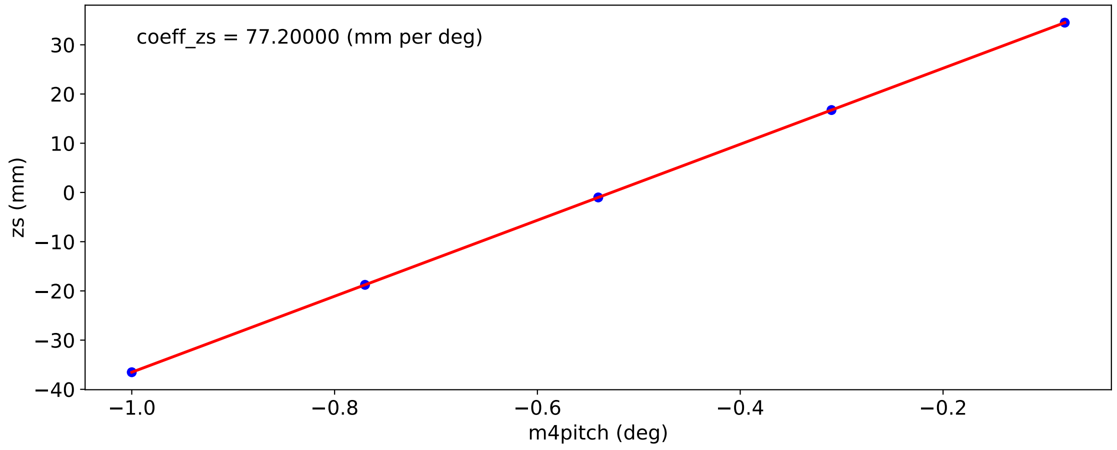

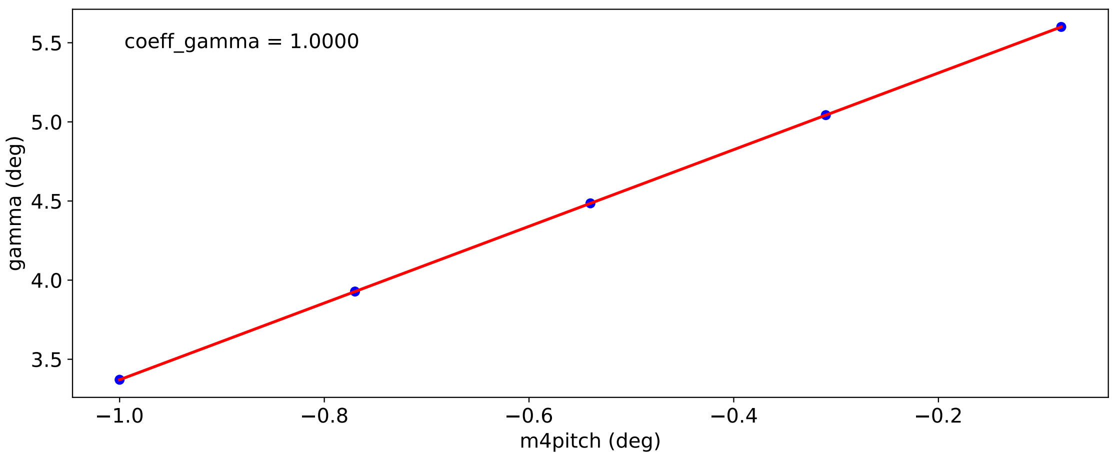

10.2 Scan XRR (liquid)

Select the first scan of the XRR series and click on `Plot XRR (liquid)`. Here we show only a few points for the example.

10.2.1 SIRIUS_2021_04_14_4298

```
Direct extracted from SIRIUS_2021_04_14_4297.nxs: direct=2.44998e+12
Starting time: 2021-04-14 14:35:30.117529
Ending time: 2021-04-14 14:43:55.901094
```

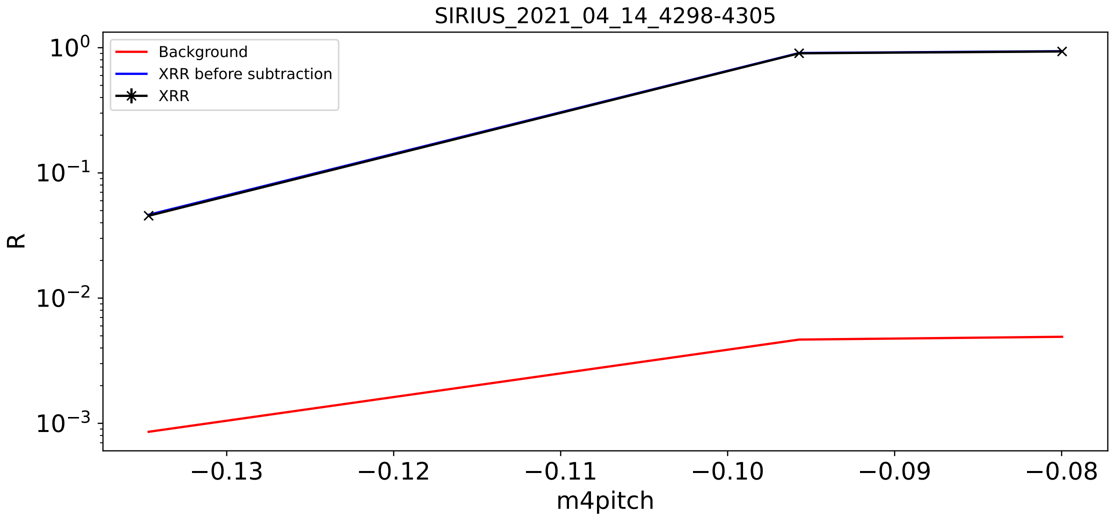

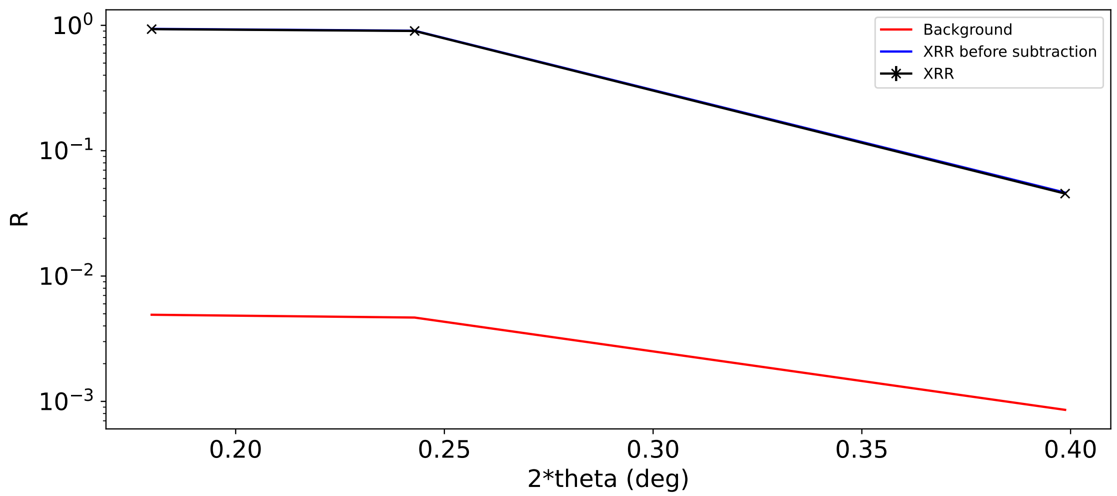
Background
XRR before subtraction
XRR
R
2*theta (deg)
0.20
0.25
0.30
0.35
0.40

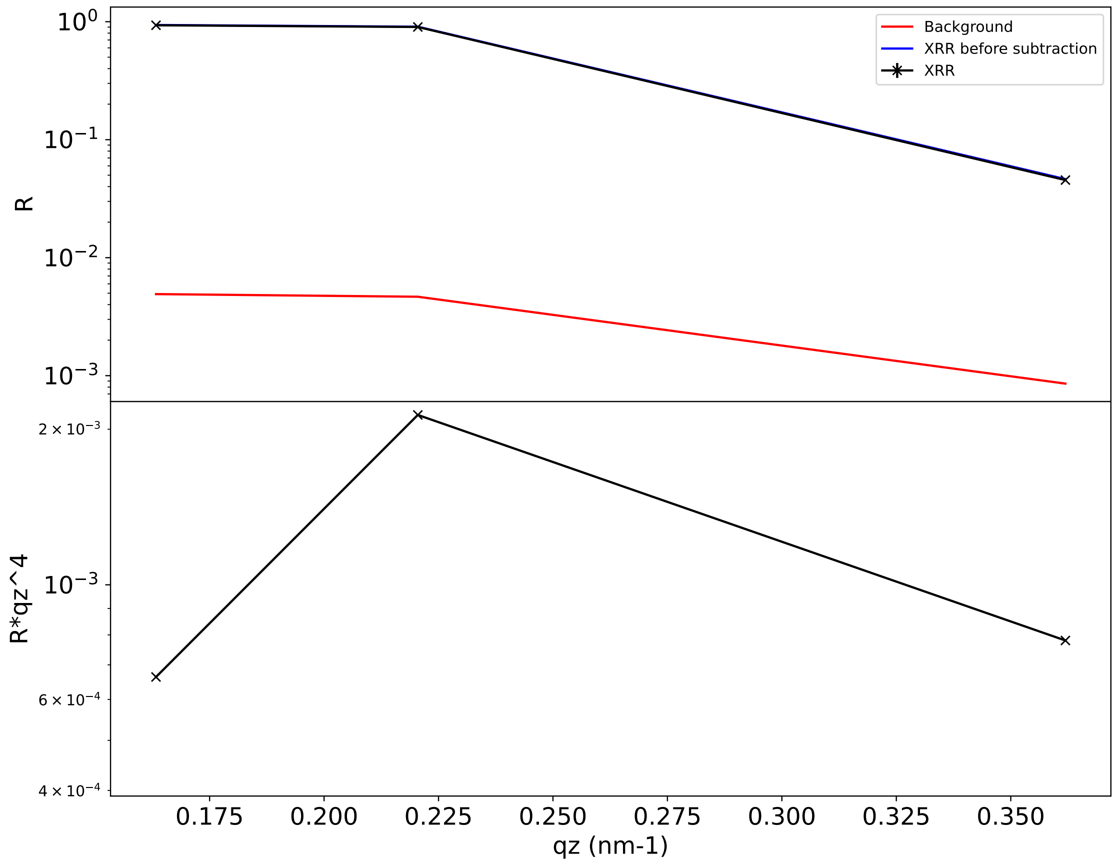
Background
XRR before subtraction
XRR
R
R*qz^4
qz (nm-1)
0.175
0.200
0.225
0.250
0.275
0.300
0.325
0.350

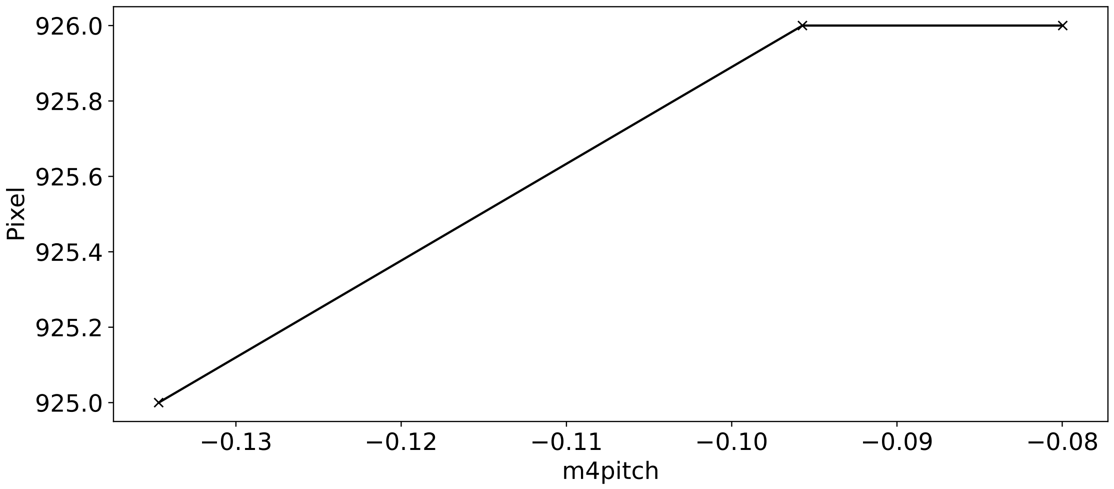


```
Save in progress…
Save done!
```

10.3 Scan XRR (solid)

Select all the scans of the XRR series and click on `Plot XRR (solid)`. Here we show only a few points for the example.

10.3.1 SIRIUS_2022_02_23_4081: run scan_XRR.ipy

```
Starting time: 2022-02-23 14:29:48.620166
Ending time: 2022-02-23 14:38:23.898086
```

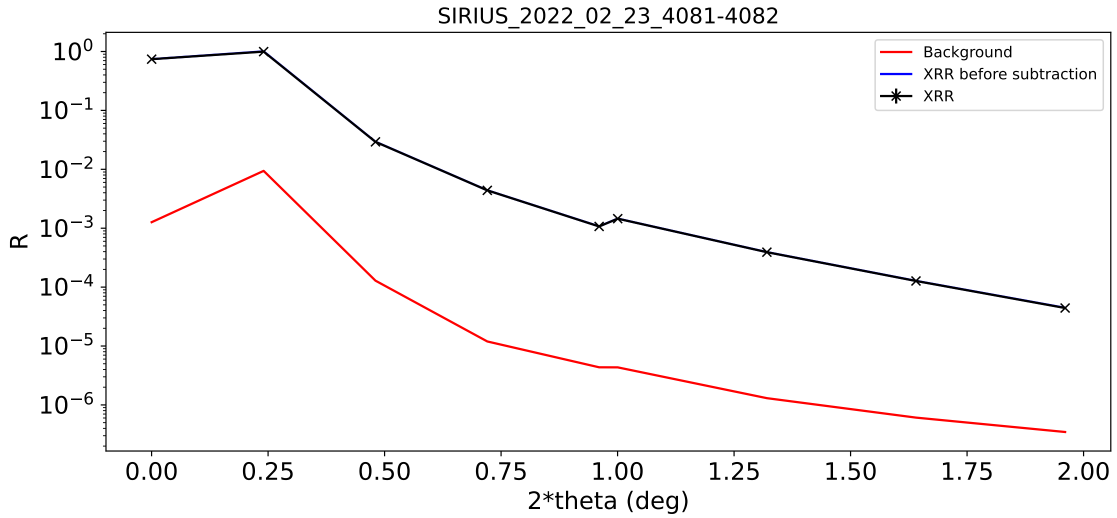

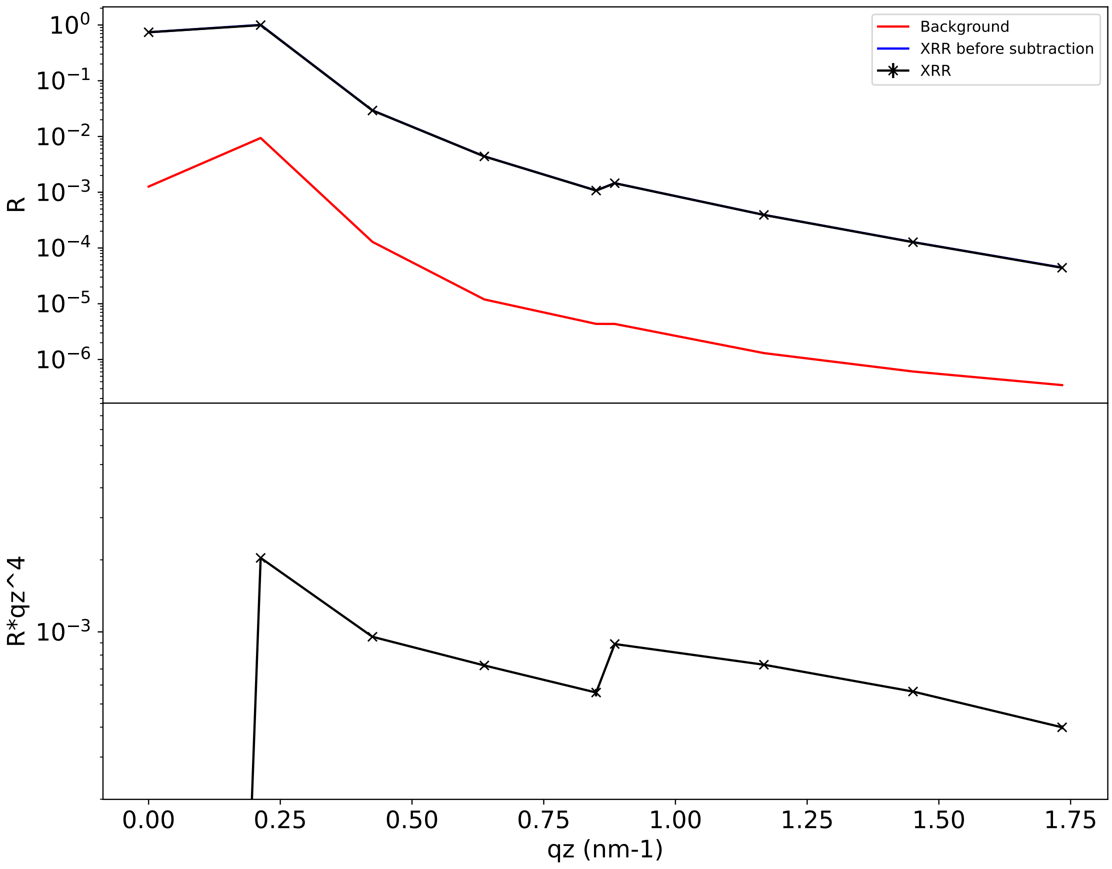


```
Save in progress…
Save done!
```

11 Insert script

Script inserted (with automatic scan numbering) using `Insert script`.

11.0.1 script_with_loop.ipy

```
%shopen
%amove delta -40
%run reset_motors.ipy
%continuous_ascan delta -35 -25 250 5 #124
%tscan 10 10 #125

for i in range(4):
```

```
    %amove delta -20
    %continuous_ascan delta -10 -3 175 5 #126 #128 #130 #132
    %run reset_motors.ipy
    %run cont_regh_abs.ipy #127 #129 #131 #133

for i in range(3):
    %amove delta -20
    %dscan delta -10 -3 175 5 #134 #136 #138
    %run reset_motors.ipy

    #%run cont_regh_abs.ipy
    %run cont_regh_abs.ipy #135 #137 #139

for i in range(50):
    %amove delta -20
    %tscan 10 100 #140 #141 #142 #143 #144 #145 #146 #147 #148 #149 #150 #151
 #152 #153 #154 #155 #156 #157 #158 #159 #160 #161 #162 #163 #164
 #165 #166 #167 #168 #169 #170 #171 #172 #173 #174 #175 #176 #177
 #178 #179 #180 #181 #182 #183 #184 #185 #186 #187 #188 #189
    %slist scan add camxdirect

%continuous_ascan delta -35 -25 250 5 #190
# %tscan 10 10
%shclose
```

12 Insert positions

12.0.1 wm delta gamma

delta	gamma
-20.0012	22.0000

12.0.2 %wm diffracto

deltacodeur	euchi	euth	euphi	kappa_h	kappa_k
0.000000 Degrees	1.00196 deg	-89.57961 deg	90.42039 deg	0.20335	1.37361

kappa_l	qxy	qxy0	qz	basexPoint	basexTrait
-0.13078	1.4125 nm-1	24.33 nm-1	-15.16 nm-1	-15.7801	-15.7801

basezPlan	basezPoint	basezTrait	basepitch	baseroll	basex
70.5895	70.5896	70.5896	0.0000 mrad	0.000 mrad	-15.780 mm

baseyaw	basez	alphax	alphay	delta	delta0
0.000 mrad	70.590 mm	0.0000	0.0000	-2.0003	-34.9995

deltaa	etaa	gamma	kappav	mu	kphi
0.0000	0.0030	22.0001	1.3080	-184.0007	0.0000

thetaa	thetah	komega	xs	ky	ys
0.0000	-0.0002	0.0000	-0.6457	-0.1000	0.0000

kz	zs	kx
0.0000	-39.9999	-0.1000

13 Insert commands

```
Wed, 30 Mar 2022 15:40:50 dmove zs -.1
Wed, 30 Mar 2022 15:40:53 ct 1
Wed, 30 Mar 2022 15:41:28 run cont_regh.ipy #456
```

14 Save/load params

Save/load the current state of the notebook (the variable jlb.expt).

15 Export

Save the notebook, export it to PDF, and convert logs to a more convenient file format.
