# Supplementary material for: Opportunities and new developments for the study of surfaces and interfaces in soft condensed matter at the SIRIUS beamline of Synchrotron SOLEIL
Source: J Synchrotron Radiat. 2024 Jan 1;31(Pt 1):162–76. doi: 10.1107/S1600577523008810 (PMC10833424; doi:10.1107/S1600577523008810)
Supplement: Supplementary file 1 [file s-31-00162-sup1.zip › JupyLabBook-v3.0.2/docs/example.zip!/example/data/SIRIUS_2017_12_11_08042_sprectogram.pdf]

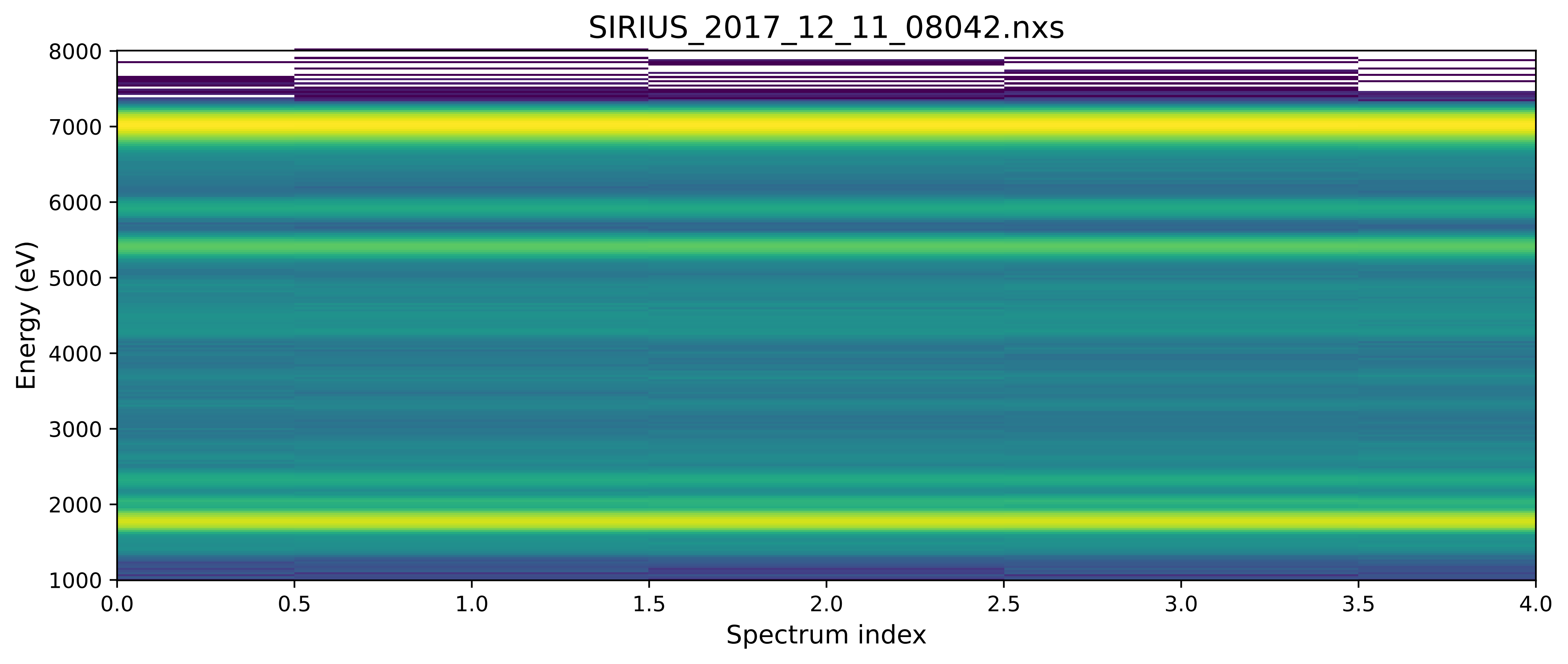
SIRIUS_2017_12_11_08042.nxs
Energy (eV)
8000
7000
6000
5000
4000
3000
2000
1000
0.0
0.5
1.0
1.5
2.0
2.5
3.0
3.5
4.0
Spectrum index
