# Supplementary material for: Opportunities and new developments for the study of surfaces and interfaces in soft condensed matter at the SIRIUS beamline of Synchrotron SOLEIL
Source: J Synchrotron Radiat. 2024 Jan 1;31(Pt 1):162–76. doi: 10.1107/S1600577523008810 (PMC10833424; doi:10.1107/S1600577523008810)
Supplement: Supplementary file 1 [file s-31-00162-sup1.zip › JupyLabBook-v3.0.2/docs/example.zip!/example/data/SIRIUS_2020_03_11_0744.pdf]

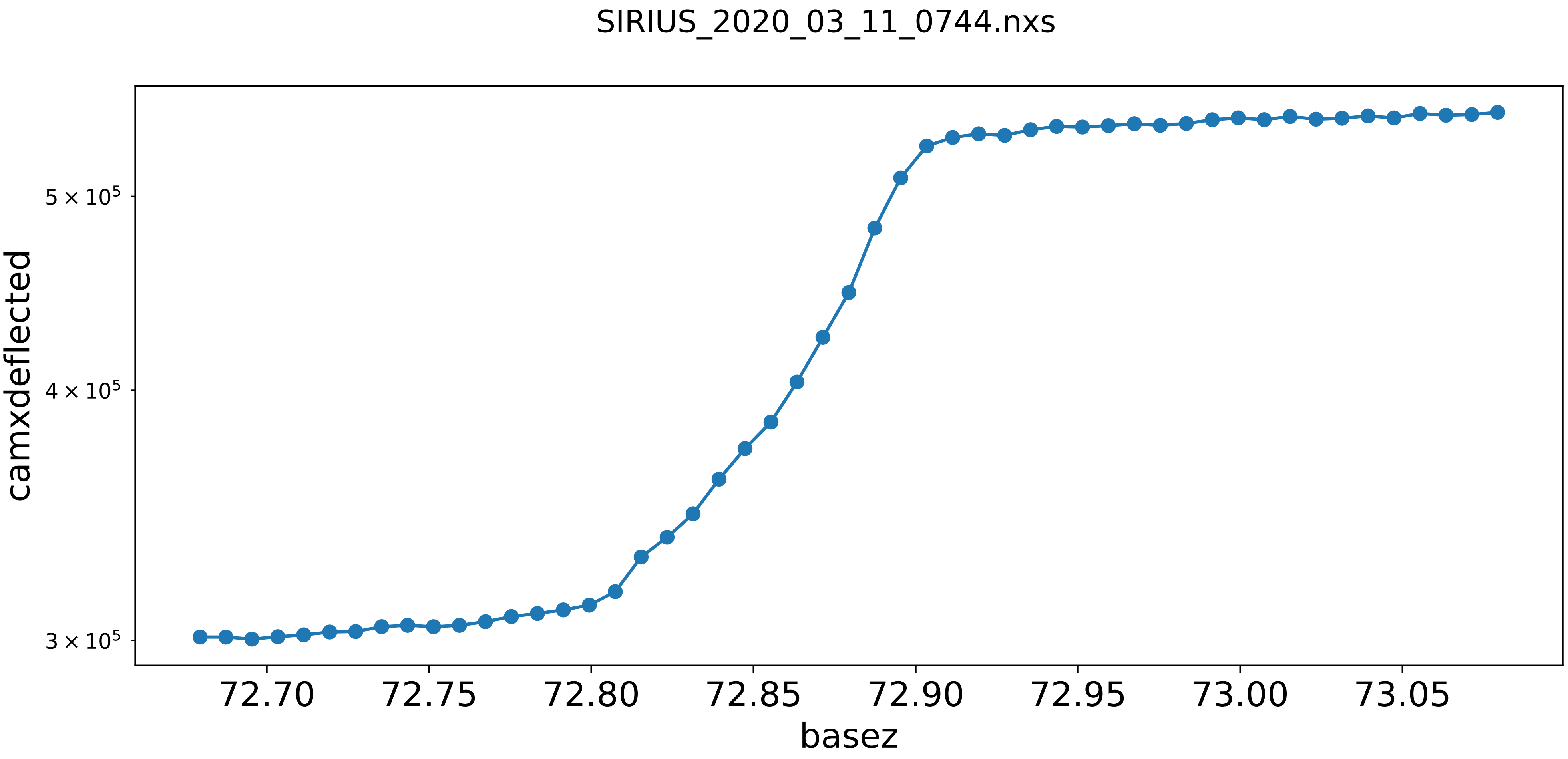
SIRIUS_2020_03_11_0744.nxs
camxdeflected
5×10^5
4×10^5
3×10^5
72.70
72.75
72.80
72.85
72.90
72.95
73.00
73.05
basez
