# Supplementary material for: Opportunities and new developments for the study of surfaces and interfaces in soft condensed matter at the SIRIUS beamline of Synchrotron SOLEIL
Source: J Synchrotron Radiat. 2024 Jan 1;31(Pt 1):162–76. doi: 10.1107/S1600577523008810 (PMC10833424; doi:10.1107/S1600577523008810)
Supplement: Supplementary file 1 [file s-31-00162-sup1.zip › JupyLabBook-v3.0.2/docs/example.zip!/example/data/SIRIUS_2020_03_11_0752.pdf]

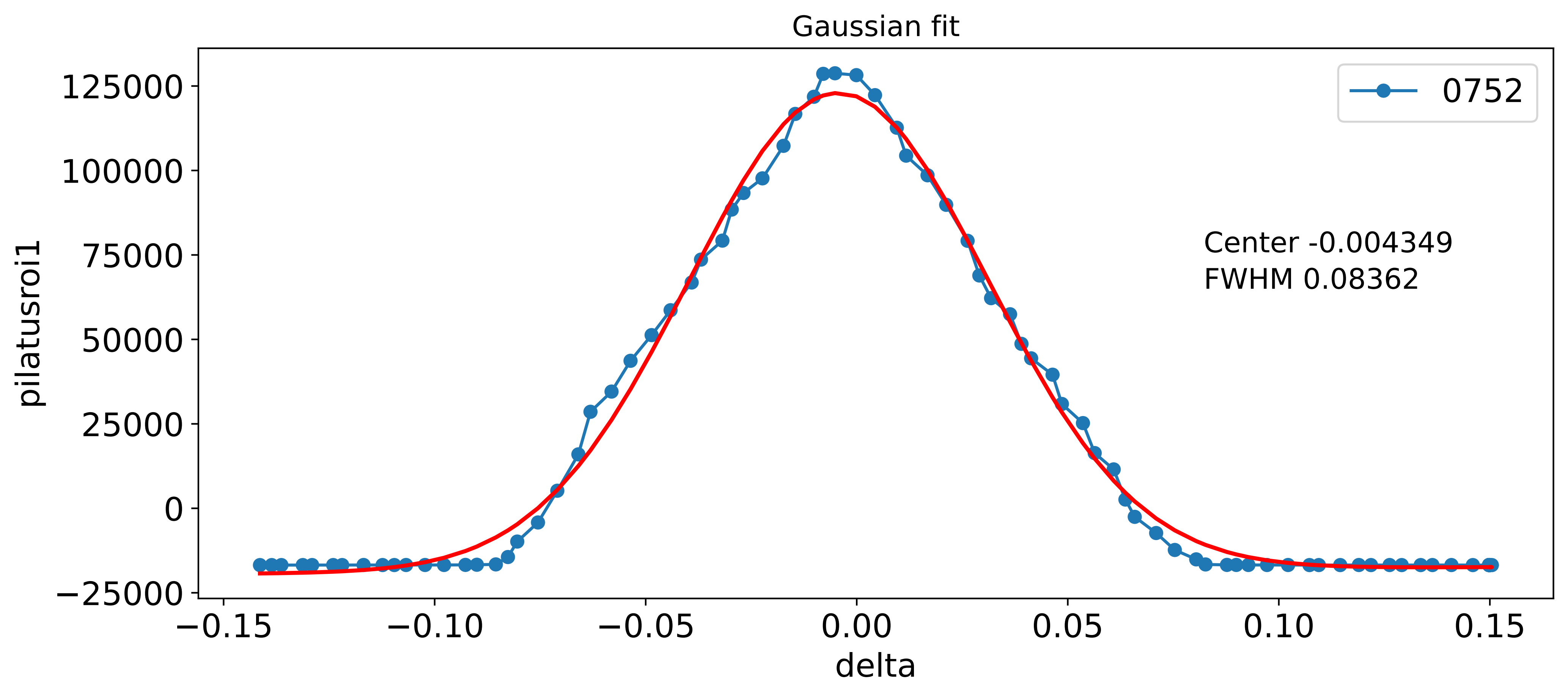

Gaussian fit
0752
Center -0.004349
FWHM 0.08362
pilatusroi1
delta
125000
100000
75000
50000
25000
0
−25000
−0.15
−0.10
−0.05
0.00
0.05
0.10
0.15
