# Supplementary material for: Opportunities and new developments for the study of surfaces and interfaces in soft condensed matter at the SIRIUS beamline of Synchrotron SOLEIL
Source: J Synchrotron Radiat. 2024 Jan 1;31(Pt 1):162–76. doi: 10.1107/S1600577523008810 (PMC10833424; doi:10.1107/S1600577523008810)
Supplement: Supplementary file 1 [file s-31-00162-sup1.zip › JupyLabBook-v3.0.2/docs/example.zip!/example/data/SIRIUS_2021_04_14_4298-4305_XRR_m4pitch.pdf]

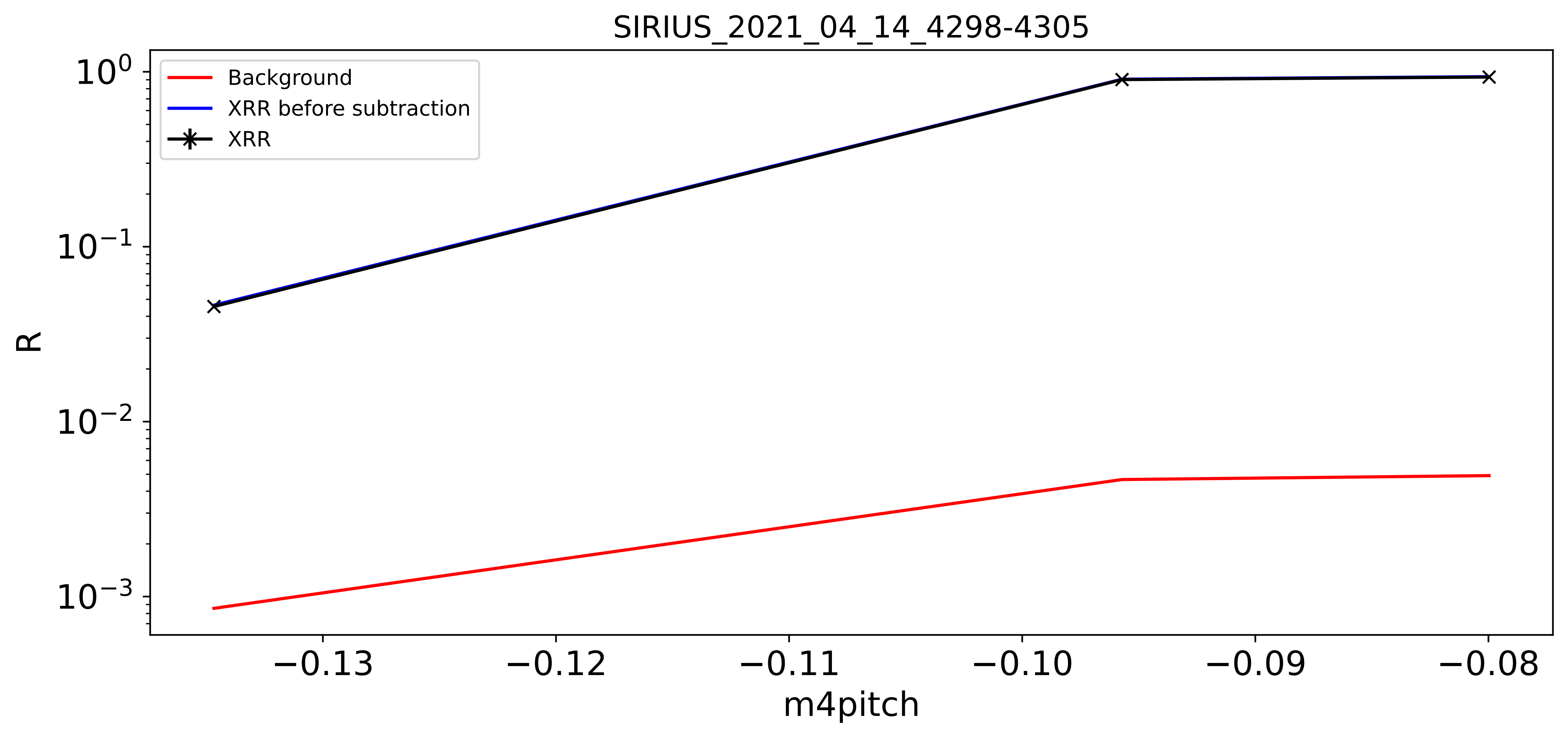

SIRIUS_2021_04_14_4298-4305
Background
XRR before subtraction
XRR
R
$10^0$
$10^{-1}$
$10^{-2}$
$10^{-3}$
−0.13
−0.12
−0.11
−0.10
−0.09
−0.08
m4pitch
