# Supplementary material for: Opportunities and new developments for the study of surfaces and interfaces in soft condensed matter at the SIRIUS beamline of Synchrotron SOLEIL
Source: J Synchrotron Radiat. 2024 Jan 1;31(Pt 1):162–76. doi: 10.1107/S1600577523008810 (PMC10833424; doi:10.1107/S1600577523008810)
Supplement: Supplementary file 1 [file s-31-00162-sup1.zip › JupyLabBook-v3.0.2/docs/example.zip!/example/data/SIRIUS_2022_03_29_0374.pdf]

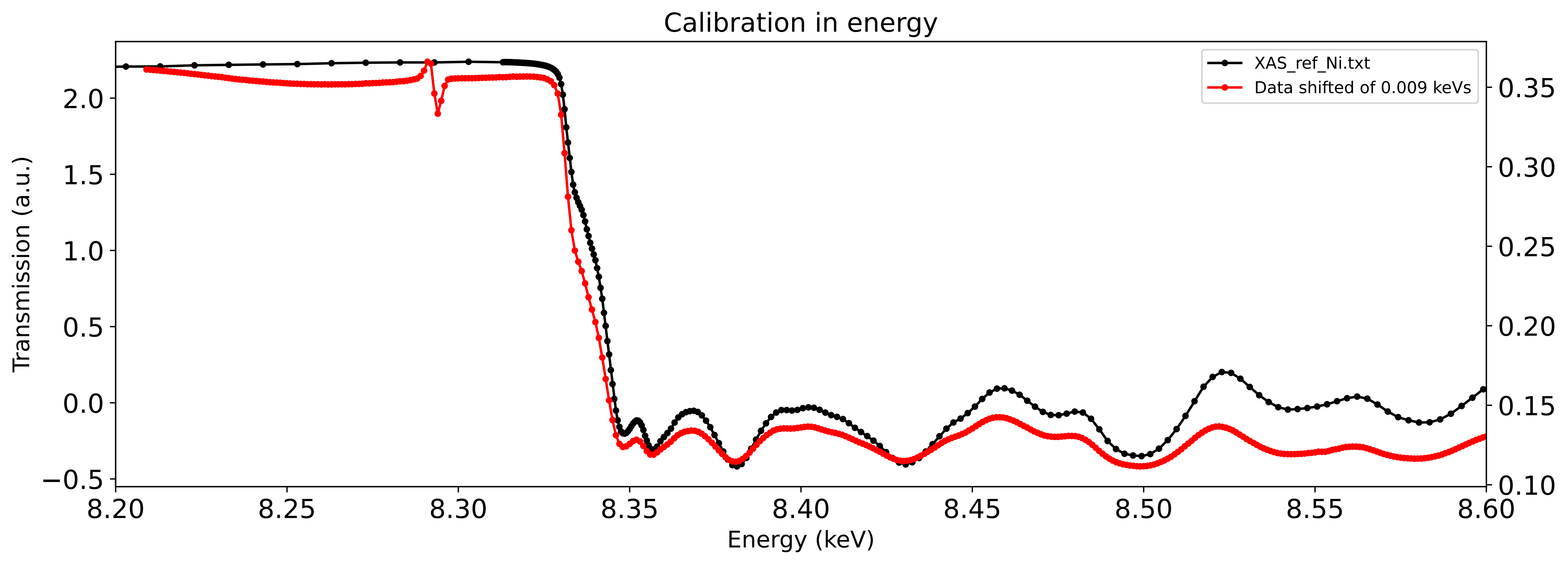

Calibration in energy
Transmission (a.u.)
Energy (keV)
XAS_ref_Ni.txt
Data shifted of 0.009 keVs
2.0
1.5
1.0
0.5
0.0
−0.5
0.35
0.30
0.25
0.20
0.15
0.10
8.20
8.25
8.30
8.35
8.40
8.45
8.50
8.55
8.60
