# Supplementary material for: Opportunities and new developments for the study of surfaces and interfaces in soft condensed matter at the SIRIUS beamline of Synchrotron SOLEIL
Source: J Synchrotron Radiat. 2024 Jan 1;31(Pt 1):162–76. doi: 10.1107/S1600577523008810 (PMC10833424; doi:10.1107/S1600577523008810)
Supplement: Supplementary file 1 [file s-31-00162-sup1.zip › JupyLabBook-v3.0.2/docs/example.zip!/example/data/SIRIUS_Fluo_2020_07_03_0042_sprectogram.pdf]

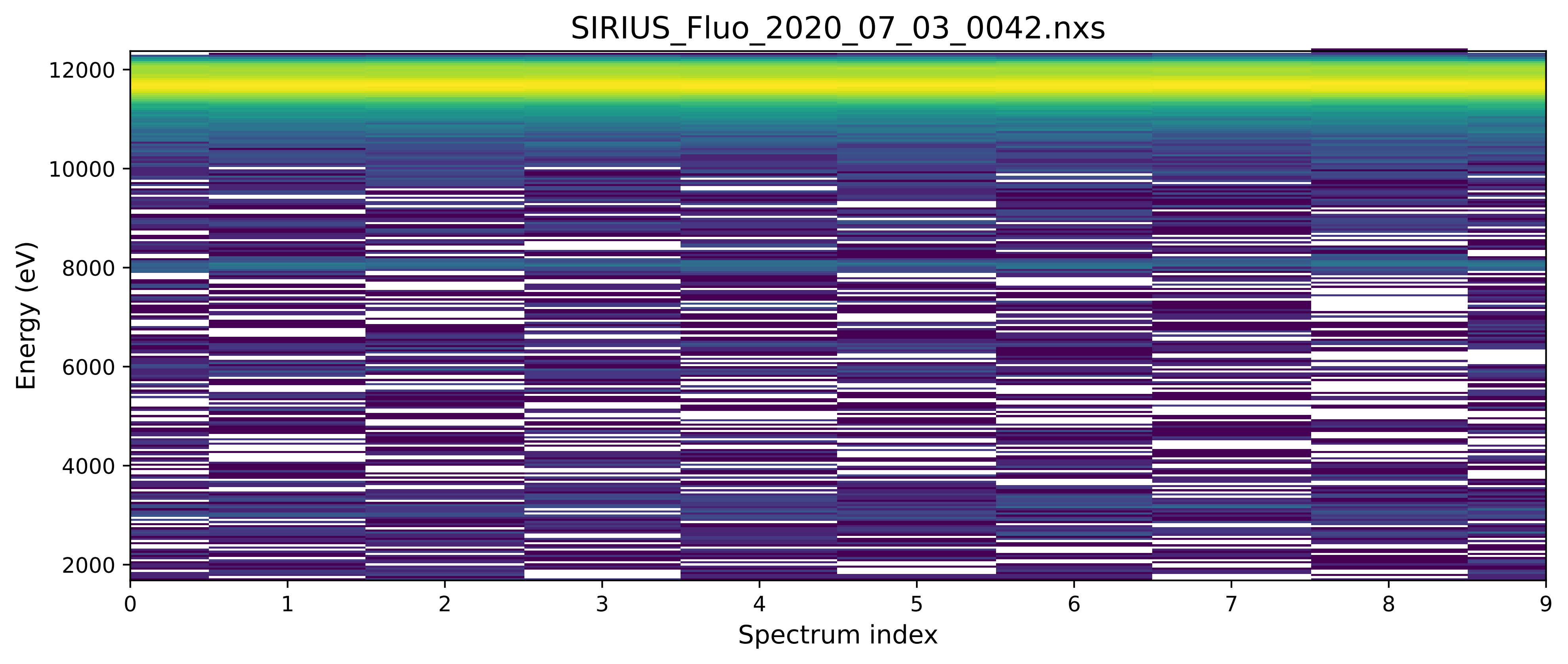
SIRIUS_Fluo_2020_07_03_0042.nxs
Energy (eV)
12000
10000
8000
6000
4000
2000
0
1
2
3
4
5
6
7
8
9
Spectrum index
