# Supplementary material for: Opportunities and new developments for the study of surfaces and interfaces in soft condensed matter at the SIRIUS beamline of Synchrotron SOLEIL
Source: J Synchrotron Radiat. 2024 Jan 1;31(Pt 1):162–76. doi: 10.1107/S1600577523008810 (PMC10833424; doi:10.1107/S1600577523008810)
Supplement: Supplementary file 1 [file s-31-00162-sup1.zip › JupyLabBook-v3.0.2/docs/example.zip!/example/data/SIRIUS_Fluo_2020_07_03_0042_first_last.pdf]

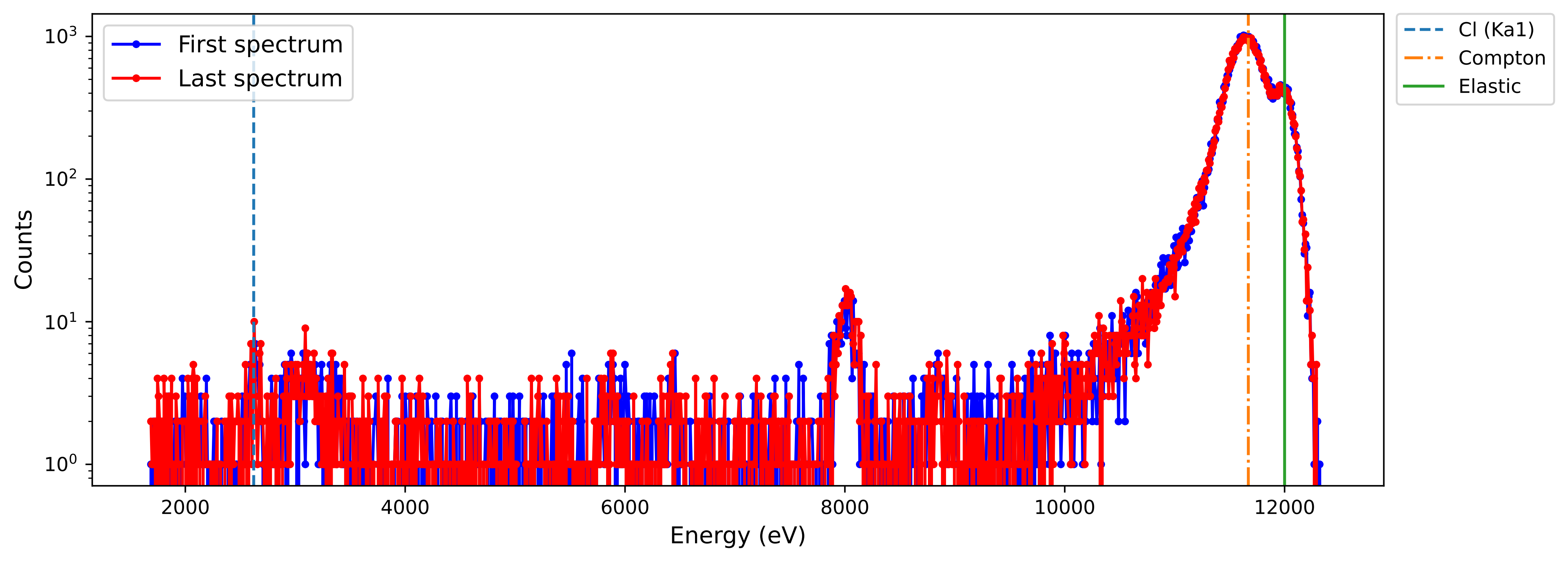
First spectrum
Last spectrum
Cl (Ka1)
Compton
Elastic
Counts
Energy (eV)
10^3
10^2
10^1
10^0
2000
4000
6000
8000
10000
12000
